# PGL-III, a Rare Intermediate of *Mycobacterium leprae* Phenolic Glycolipid Biosynthesis, Is a Potent Mincle Ligand

**DOI:** 10.1021/acscentsci.3c00040

**Published:** 2023-07-12

**Authors:** Shigenari Ishizuka, J. Hessel M. van Dijk, Tomomi Kawakita, Yuji Miyamoto, Yumi Maeda, Masamichi Goto, Guillaume Le Calvez, L. Melanie Groot, Martin D. Witte, Adriaan J. Minnaard, Gijsbert A. van der Marel, Manabu Ato, Masamichi Nagae, Jeroen D. C. Codée, Sho Yamasaki

**Affiliations:** † Department of Molecular Immunology, Research Institute for Microbial Diseases, Osaka University, 3-1 Yamadaoka, Suita, Osaka 565-0871, Japan; ‡ Laboratory of Molecular Immunology, Immunology Frontier Research Center, Osaka University, 3-1 Yamadaoka, Suita, Osaka 565-0871, Japan; § Leiden Institute of Chemistry, Leiden University, Einsteinweg 55, 2333 CC Leiden, The Netherlands; ∥ Department of Mycobacteriology, Leprosy Research Center, National Institute of Infectious Diseases, 4-2-1 Aobacho, Higashimurayama, Tokyo 189-0002, Japan; ⊥ Department of Pathology, Kagoshima University Graduate School of Medical and Dental Sciences, 8-35-1 Sakuragaoka, Kagoshima 890-8544, Japan; # Stratingh Institute for Chemistry, Nijenborgh 7, 9747 AG Groningen, The Netherlands; ∇ Center for Infectious Disease Education and Research, Osaka University (CiDER), 3-1 Yamadaoka, Suita, Osaka 565-0871, Japan

## Abstract

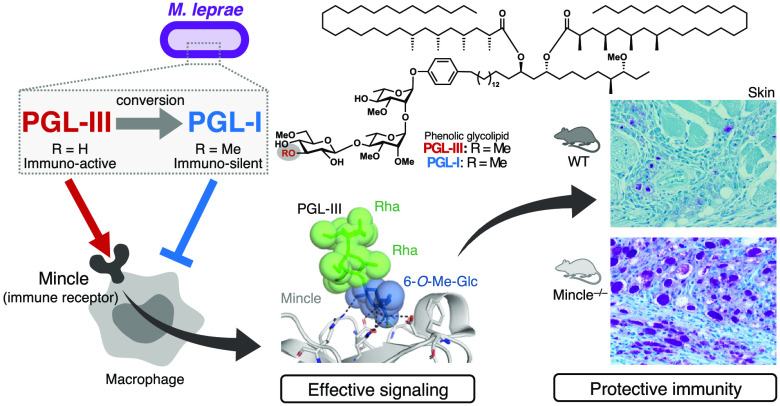

Although leprosy (Hansen’s disease) is one of the oldest known diseases, the pathogenicity of *Mycobacterium leprae* (*M. leprae*) remains enigmatic. Indeed, the cell wall components responsible for the immune response against *M. leprae* are as yet largely unidentified. We reveal here phenolic glycolipid-III (PGL-III) as an *M. leprae*-specific ligand for the immune receptor Mincle. PGL-III is a scarcely present trisaccharide intermediate in the biosynthetic pathway to PGL-I, an abundant and characteristic *M. leprae* glycolipid. Using activity-based purification, we identified PGL-III as a Mincle ligand that is more potent than the well-known *M. tuberculosis* trehalose dimycolate. The cocrystal structure of Mincle and a synthetic PGL-III analogue revealed a unique recognition mode, implying that it can engage multiple Mincle molecules. In Mincle-deficient mice infected with *M. leprae*, increased bacterial burden with gross pathologies were observed. These results show that PGL-III is a noncanonical ligand recognized by Mincle, triggering protective immunity.

## Abstract 2

PGL-III, a potent immunostimulatory glycolipid, is limited in *M. leprae* by the quick addition of a single methyl group to convert it into immunosuppressive PGL-I, which confers immune escape.

## Introduction

*Mycobacterium leprae* (*M. leprae*) is the causative pathogen of leprosy, also called Hansen’s disease. Leprosy is an ancient, chronic infectious disease that affects the skin, peripheral nerves, and eyes.^1^ Over 140 000 new cases of leprosy were detected during 2021, including more than 9000 children.^2^ Although leprosy can be cured with multidrug therapy (MDT), it can result in lifelong handicaps and irreversible deformities if left untreated.^3^ The disabilities, deformities, and morbidity of leprosy are mainly caused by the inflammatory exacerbation of skin lesions and nerve trunks, leading to motor and sensory alterations.^1^ The trigger for these characteristic leprosy symptoms is not fully understood.

The mycobacterial cell wall is characterized by a thick hydrophobic lipid layer that harbors many structurally unique glycolipids.^4^ These lipids are critical to the virulence and pathogenicity of the bacteria and play an all-important role in host–pathogen interactions.^4^ The combination of both immunostimulatory and immunomodulatory lipids in the mycobacterial cell wall provides these bacteria with a unique armamentarium to exploit the host immune system and establish long-lasting infections. Immune responses against mycobacteria are initiated when pattern recognition receptors (PRRs) sense the mycobacterial lipids. These PRRs include members of the Toll-like receptor (TLR) and C-type lectin receptor (CLR) families.^5,6^ CLRs have been shown to recognize common mycobacterial glycolipids. For example, interactions of trehalose dimycolate (TDM) with Mincle/MCL (Clec4e/Clec4d), mannose-capped lipoarabinomannan (Man-LAM) with Dectin-2 (Clec4n), and acylated phosphatidyl inositolmannosides (AcPIMs) with DCAR (Clec4b1) have been reported.^7−10^

The most prominent lipid in the cell wall of *M. leprae* is phenolic glycolipid-I (PGL-I), which constitutes up to 2% of the bacterial cell mass.^11^ Mycobacterial phenolic glycolipids are biosynthesized from a phenolphthiocerol dimycocerosate lipid that is well-conserved among various mycobacteria and is decorated with species-specific saccharides.^12^ It has been postulated that the nature of the saccharide moieties may play an important role in interactions with the host immune system. *M. leprae* PGL-I carries a 3,6-di-*O*-methyl-β-d-glucopyranosyl-(1 → 4)-2,3-di-*O*-methyl-α-l-rhamnopyranosyl-(1 → 2)-3-*O*-methyl-α-l-rhamnopyranosyl-(1 → ) trisaccharide.^11^ PGL-I has a potent immunosuppressive role,^13,14^ which may confer an immune-silent property to *M. leprae* and cause chronic infection in the host. It is also possible that immunostimulatory components are modified or suppressed in *M. leprae* to evade host immunity. However, the identity and behavior of such active components have not been well characterized, particularly in the presence of immunosuppressive lipid PGL-I. Elucidating the regulatory mechanisms of these components may lead to an understanding of an immune-escaping strategy of *M. leprae*, which may provide further therapeutic options.

We thus searched for the potential immunostimulatory components in the (glyco)lipid cell wall of *M. leprae*. We discovered, through a combination of lipid extract screening, sensitive cell-based assays, biosynthesis, and organic synthesis that a unique biosynthesis precursor to PGL-I, i.e., PGL-III, is a highly potent immunostimulating glycolipid that signals through Mincle. The Mincle–PGL-III interaction is distinct from the canonical Mincle–TDM binding as revealed by X-ray crystallography. Using an *in vivo* model, we further demonstrated the protective role of Mincle-signaling against *M. leprae* infection. In sum, the present study has identified PGL-III as a novel and potent ligand for Mincle that initiates a strong pro-inflammatory response against *M. leprae*.

## Results

### Mincle Recognition of *M. leprae*-Specific Glycolipids

**Figure 1:**
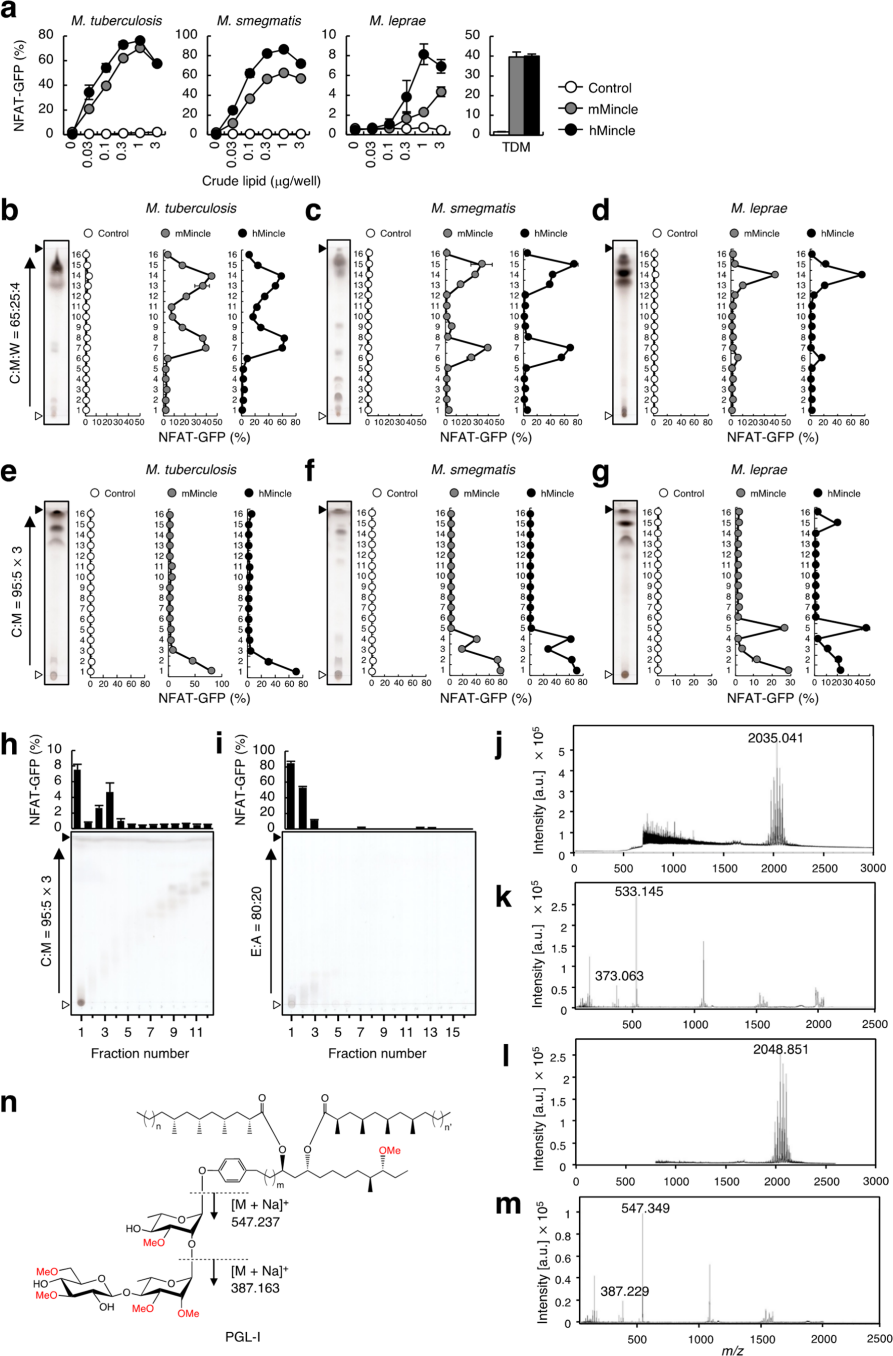
A PGL-related molecule is the Mincle ligand in *M. leprae*. (a) 2B4-NFAT-GFP reporter cells expressing mMincle + FcRγ (mMincle), hMincle + FcRγ (hMincle), or FcRγ alone (control) were stimulated with the indicated doses of crude lipid extracted from *M. tuberculosis*, *M. smegmatis*, and *M. leprae* using C:M (2:1 v/v) or TDM (0.3 μg/well) as a control. Cells were stimulated for 20 h and analyzed for GFP expression. (b–g) Crude lipids extracted from *M. tuberculosis* (b, e), *M. smegmatis* (c, f), and *M. leprae* (d, g) were developed by C:M:W (65:25:4 v/v/v) (b–d) or C:M (95:5 v/v, three runs) (e–g) and fractionated into 16 fractions. Reporter cells expressing mMincle or hMincle were stimulated with each fraction for 20 h and analyzed for GFP expression. (h) The crude lipid extract from *M. leprae* was divided into 16 fractions by HPTLC using C:M (95:5 v/v, three runs). Reporter cells expressing hMincle were stimulated with individual fractions for 20 h and analyzed for GFP expression. Each fraction was further analyzed by HPTLC using C:M (95:5 v/v, three runs) followed by staining with copper acetate reagent. (i) The reporter activity positive fraction was further subjected to HPTLC using E:A (80:20 v/v) and fractionated into 16 fractions. Reporter cells expressing hMincle were stimulated with individual fractions for 20 h and analyzed for GFP expression. Each fraction was further analyzed by HPTLC using E:A (80:20 v/v). (j) MS spectrum of the purified lipid (Figure 1i, fraction 1) in positive ion mode. The ion peak was at *m*/*z* = 2035.041 [M + Na]^+^. (k) MS/MS spectrum of *m*/*z* = 2035.041 [M + Na]^+^ shown in panel j. (l) MS spectrum of PGL-I purified from crude lipid in positive ion mode. The ion peak was at *m*/*z* = 2048.851 [M + Na]^+^. (m) MS/MS spectrum of *m*/*z* = 2048.851 [M + Na]^+^ shown in panel l. (n) The structure of PGL-I and hypothesized fragmentation mode. Indicated values correspond to *m*/*z* [M + Na]^+^ of the fragments. Open and closed arrowheads beside TLC pictures denote the origin and the solvent front, respectively. Data are presented as the mean ± SD of triplicate assays and are representative of two independent experiments with similar results (a–i).

We first examined whether *M. leprae*-derived lipids interacted with host receptors using a wide range of CLR reporter cell lines and found that reporter cells expressing Mincle were activated in the presence of crude lipid extracts from *M. leprae* as well as *M. tuberculosis* and *M. smegmatis* (Figures 1a and S1a–c). We next separated the lipid extracts using high-performance thin layer chromatography (HPTLC) to characterize the responsible active component(s) in fractionation-based assays. Ligands activating Mincle were detected in all mycobacterial species around fractions 6–8 and 13–15 (Figure 1b–d). Judging from their *R_f_* values, fractions 6–8 are expected to contain trehalose monomycolate^7^ (TMM) and fractions 13–15 are expected to contain trehalose dimycolate^7^/glucose monomycolate^15^ (TDM/GMM). However, the activity of fraction 6 from *M. leprae* was much lower than that of corresponding fractions from *M. tuberculosis* and *M. smegmatis* (Figure 1d). While many mycobacteria produce comparable amounts of TDM/GMM and TMM,^16−18^*M. leprae* generates only a limited amount of these glycolipids.^19^ We therefore suspected that fractions 13–15 from the *M. leprae* lipid extract must contain a ligand other than TDM/GMM. Indeed, fractionation using a different combination of solvents revealed an altered fraction activity profile for the *M. leprae* lipid mixture (Figure 1e–g) and a low running lipid (no. 5) from *M. leprae* selectively activated reporter cells expressing Mincle (Figure 1g). These results suggest that *M. leprae* possesses an unknown Mincle ligand distinct from TDM/GMM or TMM.

To isolate the active component(s) specific to *M. leprae*, we separated a large amount of total lipid extract into 16 fractions using HPTLC with chloroform:methanol (C:M = 95:5 v/v, three runs) (Figure 1h). In this manner, we obtained active fractions (nos. 3 and 4) that we collected and further separated using ether:acetone (E:A = 80:20 v/v). Peak activity was detected in fraction 1 (Figure 1i). An analysis of this spot using matrix-assisted laser desorption/ionization time-of-flight mass spectrometry (MALDI-TOF-MS) revealed multiple molecular ions around *m*/*z* = 2000 (Figure 1j). The difference in *m*/*z* of each peak was exactly 14.014, implying a different number of CH_2_ moieties in the structures. The low polarity of the compound and the high molecular weight (Figure 1d,j) indicated that this active component likely has long fatty acid chains. MS/MS analysis provided fragment ions *m*/*z* = 533.145 and 373.063 (Figure 1k), suggesting the presence of partially methylated tri- and disaccharides, respectively. As one of the characteristic *M. leprae* glycolipids harboring three hexoses is PGL-I,^11^ we analyzed this lipid, which we purified from *M. leprae*, using the same techniques. Similar ions around *m*/*z* = 2000 were detected (Figure 1l), and the MS/MS spectrum showed fragment ions *m*/*z* = 547.349 and 387.229 (Figure 1m), which correspond to sodium adducts of the 3,6-di-*O*-methyl-β-d-glucopyranosyl-(1 → 4)-2,3-di-*O*-methyl-α-l-rhamnopyranosyl-(1 → 2)-3-*O*-methyl-α-l-rhamnopyranosyl-(1 → ) trisaccharide and the 3,6-di-*O*-methyl-β-d-glucopyranosyl-(1 → 4)-2,3-di-*O*-methyl-α-l-rhamnopyranosyl-(1 → ) disaccharide fragments, respectively (Figure 1n).^11^ The difference in *m*/*z* of these sugar fragments with respect to the fragments analyzed from the purified lipid extract (±14) suggests that the Mincle-reactive fraction lacks one methyl group in comparison to the PGL-I glycan. Taken together, these results suggested that the active component in the *M. leprae* extract is a glycolipid with high similarity to PGL-I.

### PGL-III is a Ligand for Mincle

**Figure 2:**
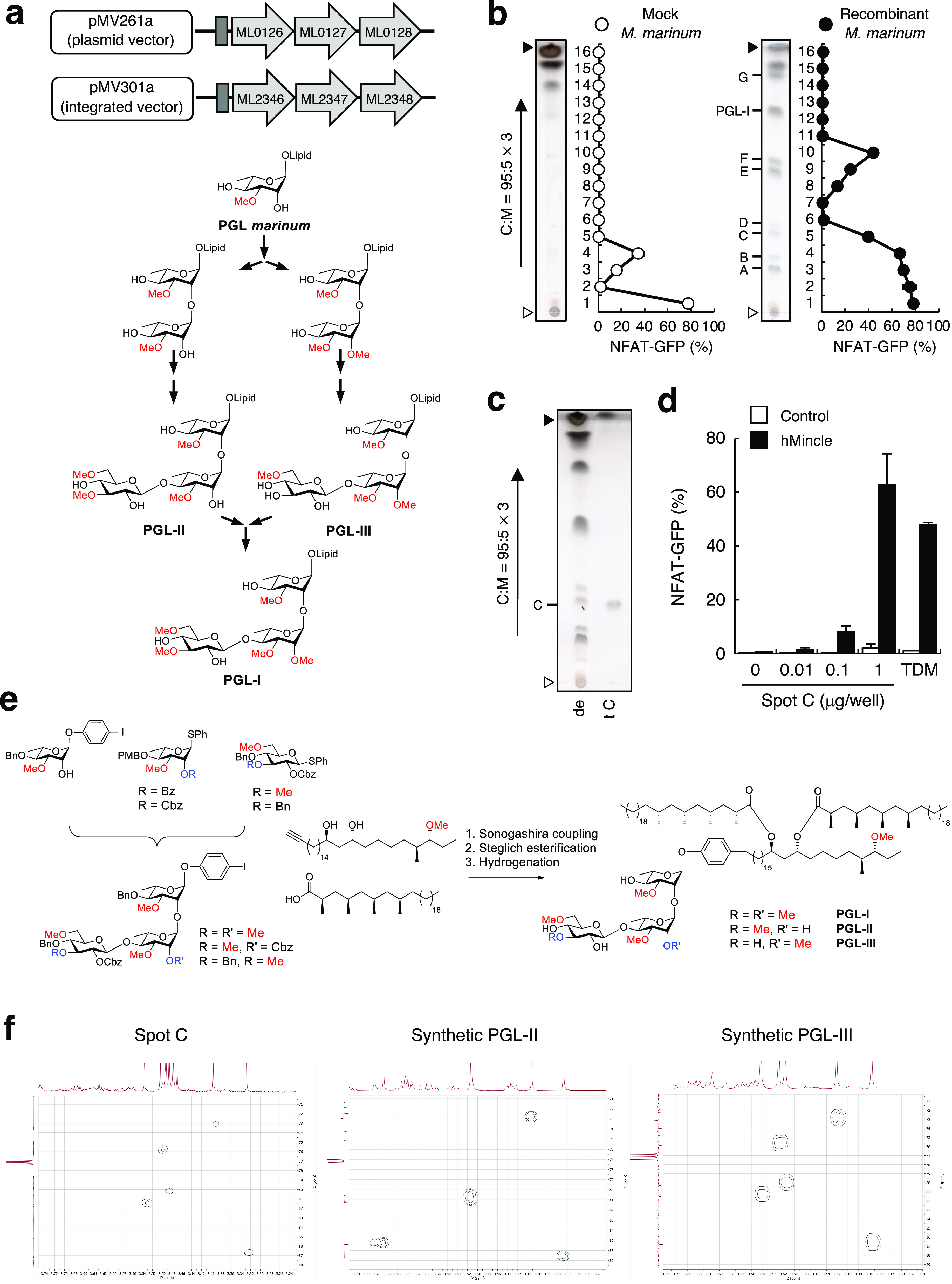
Identification of PGL-III as a ligand for Mincle. (a) Schematic diagram of *M. leprae* genes transformed into *M. marinum*. Arrows and boxes represent each enzyme and the Hsp60 promoter, respectively. ML0126, Rha-3-*O*-methyltransferase; ML0127, Rha-2-*O*-methyltransferase; ML0128, rhamnosyl transferase; ML2346 and ML2347, Glc-3- and Glc-6-*O*-methyltransferases; and ML2348, glycosyl transferase. (b) Acetone-soluble lipids were extracted from crude lipids derived from mock and recombinant *M. marinum* and were fractionated into 16 fractions by HPTLC using C:M (95:5 v/v, three runs). Reporter cells expressing hMincle were stimulated with each fraction for 20 h and analyzed for GFP expression. (c) Isolation of spot C assessed by HPTLC using C:M (95:5 v/v, three runs) and visualized by copper acetate staining. (d) Reporter cells expressing hMincle were stimulated with the indicated doses of spot C lipid or TDM (0.3 μg/well) for 20 h and analyzed for GFP expression. (e) Synthesis of PGL-I, -II, and -III. (f) Comparison of HMBC experiments using spot C lipid, synthetic PGL-II, and synthetic PGL-III. Open and closed arrowheads beside TLC pictures denote the origin and the solvent front, respectively. Data are presented as the mean ± SD of triplicate assays and are representative of two independent experiments with similar results (b, c).

Since several PGL-I-like intermediates are produced in the PGL-I biosynthetic pathway,^20^ we reasoned that the analysis of this pathway would provide further insight into the nature of this ligand. To this end, we reconstituted the PGL-I biosynthetic pathway from *M. leprae* in *M. marinum*, a mycobacterial species that does not produce PGL-I and readily grows *in vitro* (Figures 2a and S2). Upon introduction of six enzyme-encoding genes,^20^ i.e., rhamnosyl 3-*O*-methyltransferase (ML0126), rhamnosyl 2-*O*-methyltransferase (ML0127), rhamnosyl transferase (ML0128), two glucosyl methyltransferases (ML2346 and ML2347), and glucosyltransferase (ML2348), *M. marinum* produced a large amount of PGL-I, as previously reported.^21^ When developing the lipid extract of this PGL-I-producing *M. marinum* strain, several spots in addition to PGL-I were detected (Figure 2b), which are potential intermediates in the PGL-I biosynthetic pathway. Among these, we demonstrated that spot C (Figure 2c), which is more polar than PGL-I (Figure 2b), possessed substantial Mincle-activating properties (Figure 2d). Both PGL-II and -III, intermediate products of the PGL-I synthetic pathway (Figure 2a), have previously been isolated and identified from *M. leprae*,^22^ and we hypothesized that these might be candidates for the Mincle-active component in the lipid extract.

To obtain pure samples of PGL-I, -II, and -III, we generated these complex glycolipids through organic synthesis. We followed a highly convergent assembly strategy that we previously introduced for the total synthesis of PGL-tb1 in *M. tuberculosis*,^23^ building on our recent syntheses of *M. leprae* trisaccharide-BSA conjugates (Figure 2e).^24^ We masked the unmethylated PGL hydroxy groups with hydrogenolysis-labile groups (benzyl ethers and benzyloxycarbonates) to facilitate the final deprotection and safeguard the mycocerosic acid esters. The glycans were functionalized with a *p*-iodophenol at the reducing end, which allowed effective fusion to the phthiocerol alkyne derivative through Sonogashira cross coupling. The mycocerosic acids were introduced using Steglich conditions, and a final hydrogenation step led to the global deprotection and concurrent reduction of the internal alkyne, which had been formed in the Sonogashira reaction. The *M. leprae* PGLs could be assembled on a multimilligram scale, providing sufficient compounds for all subsequent studies. With the pure synthetic samples in hand, we compared their NMR spectra with that of the lipid isolated from *M. marinum* spot C (Figure 2c). Heteronuclear multiple bond correlation (HMBC) experiments were used to determine the exact positions of the methyl ethers present in the trisaccharides. As shown in Figure 2f, the HMBC spectrum of synthetic PGL-III corresponds well with the spectrum of spot C. Indeed, the MS/MS spectrum of a purified Mincle-activating fraction (Figure 1i) is consistent with the presence of the 6-*O*-methyl-β-d-glucopyranosyl-(1 → 4)-2,3-di-*O*-methyl-α-l-rhamnopyranosyl-(1 → 2)-3-*O*-methyl-α-l-rhamnopyranosyl-(1 → ) trisaccharide and the 6-*O*-methyl-β-d-glucopyranosyl-(1 → 4)-2,3-di-*O*-methyl-α-l-rhamnopyranosyl-(1 → ) disaccharide in PGL-III. Although minor differences were observed in the aliphatic region because of the difference in the phthiocerol dimycocerosate (PDIM) moiety between *M. marinum* and *M. leprae*,^12^ the mobilities of PGL-III derived from *M. leprae* and reconstituted *M. marinum* were comparable to that of synthetic PGL-III (Figure S3).

These results suggested that spot C corresponds to a glycolipid with a PGL-III saccharide moiety linked to an *M. marinum* PDIM moiety as the active component. The saccharide moiety is crucial to the Mincle ligand activity, while the requirement of the PDIM moiety is not restricted by *M. leprae*-specific lipid chains.

### Synthetic PGL-III Induces Potent Immune Responses through Mincle

**Figure 3:**
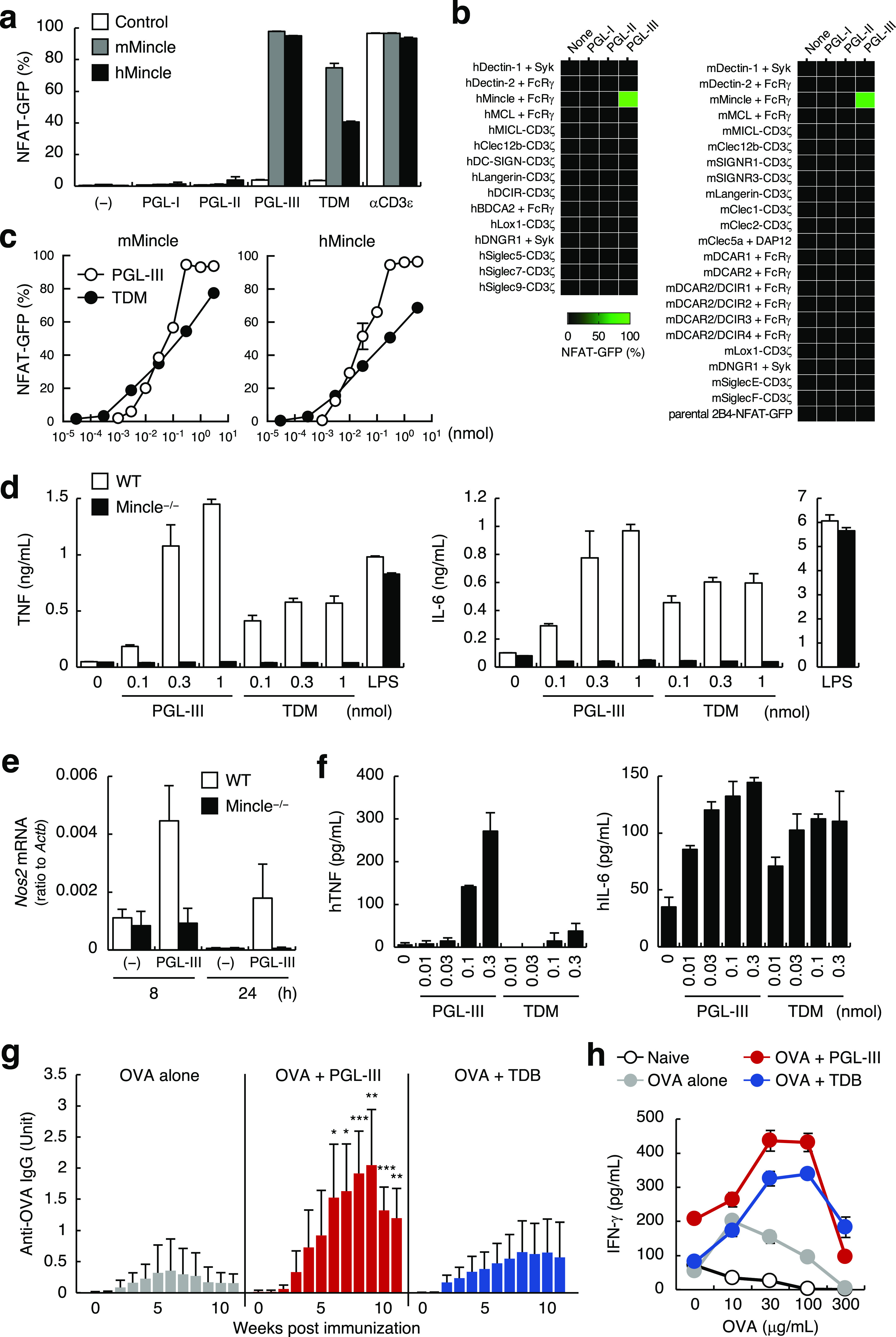
Synthetic PGL-III is recognized by Mincle with positive cooperativity and induces innate immune responses. (a) Reporter cells expressing mMincle or hMincle were stimulated with the indicated glycolipids (0.3 nmol/well) or plate-coated anti-mouse CD3ε mAb for 20 h and analyzed for GFP expression. (b) Reporter cells expressing various CLRs were stimulated with the indicated synthetic PGLs (0.3 nmol/well) for 20 h and analyzed for GFP expression. GFP expression is shown as a heat map. (c) Reporter cells expressing mMincle or hMincle were stimulated with the indicated doses of PGL-III or TDM for 20 h and analyzed for GFP expression. (d) IFN-γ/LPS-primed bone marrow-derived macrophages (BMDMs) from wild type or Mincle^–/–^ mice were stimulated with the indicated doses of PGL-III, TDM, or LPS (0.1 μg/mL) for 48 h, and the culture supernatants were analyzed for the production of pro-inflammatory cytokine production. (e) IFN-γ-primed BMDMs from wild type and Mincle^–/–^ mice were stimulated with PGL-III (5.94 nmol/well) on a 24-well plate for 8 or 24 h, and the *Nos2* mRNA expression level was determined by RT-PCR. (f) Human monocyte-derived dendritic cells (hMoDCs) were stimulated with the indicated doses of PGL-III or TDM for 24 h, and the culture supernatants were analyzed for pro-inflammatory cytokine production. (g) OVA-specific IgG production in the serum of mice immunized with OVA in the presence of PGL-III or trehalose dibehenate (TDB). Antibody production was quantified by ELISA using the sera pool from OVA/alum-treated mice as a standard. Each group includes at least five mice. *, *p* < 0.05; **, *p* < 0.01; and ***, *p* < 0.005 vs OVA alone-treated group. (h) Recall T cell response of immunized mice. IFN-γ production from lymph node cells upon stimulation with the indicated concentrations of OVA protein was determined by ELISA. Cells were stimulated on a 96-well plate unless otherwise specified. Data are presented as the mean ± SD of triplicate (a, c, d–f, h) or duplicate (b) assays and are representative of at least two independent experiments with similar results (a–h).

The activities of the synthetic PGLs were subsequently assessed using reporter cells. As expected, only PGL-III, not PGL-I or -II, showed potent ligand activity against both mouse and human Mincle (Figure 3a), and this activity was specific to Mincle (Figure 3b). Given its low abundance and relative activity (Figure 1g), the specific activity of PGL-III appears to be high. Indeed, the EC_50_ of PGL-III is lower than that of the authentic Mincle ligand TDM, particularly in the case of human Mincle, as revealed by the dose-dependent curves in Figure 3c. PGL-III showed a steeper dose–response curve for the reporter cell activity, as compared to TDM (Figure 3c), implying a unique function during infection. Synthetic PGL-III also activated primary macrophages to produce pro-inflammatory cytokines, such as TNF and IL-6, in a Mincle-dependent manner (Figure 3d) as reported for other Mincle ligands.^7^ However, the potency of PGL-III was much higher than that of TDM. Furthermore, PGL-III induced the expression of *Nos2* (Figure 3e) which synthesizes nitric oxide (NO) for controlling *M. leprae* infection.^25^ Consistent with the results of the reporter cells, PGL-III could also activate human macrophages to produce TNF and IL-6 (Figure 3f). These results show that *M. leprae* PGL-III is a strong immuno-stimulating agent, triggering the release of pro-inflammatory cytokines. PGL-III also enhanced ovalbumin (OVA)-specific IgG production (Figure 3g) and IFN-γ production from T cells (Figure 3h), suggesting that PGL-III boosts acquired immune responses as an adjuvant *in vivo*. In sum, these results indicate that PGL-III is a novel Mincle ligand with a structure and characteristics distinct from those of previously reported ligands.^26^

### Mincle-Deficient Mice Are Susceptible to *M. leprae* Infection

**Figure 4:**
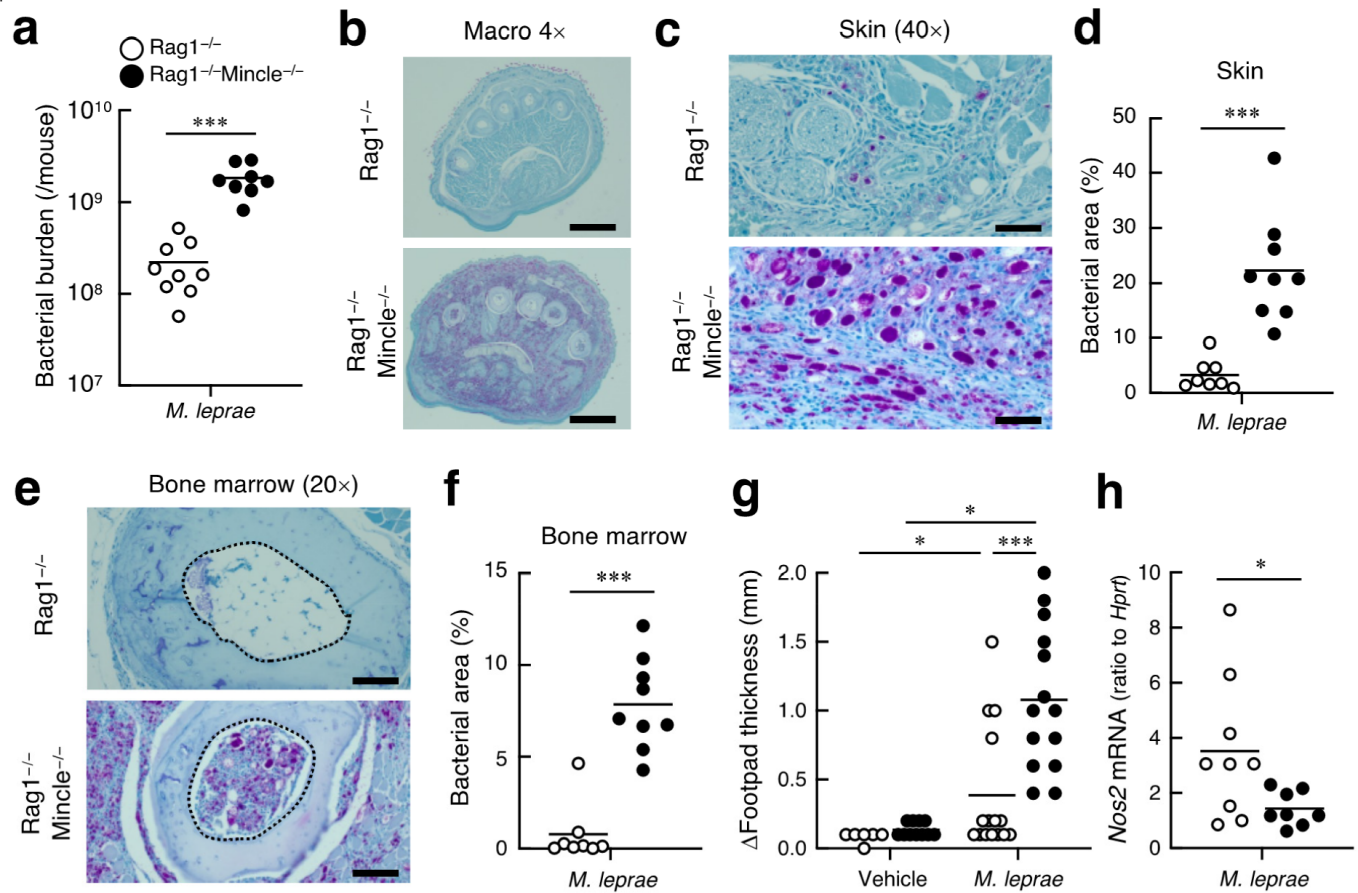
Mincle protects against *M. leprae* infection. (a) Bacterial burden of *M. leprae***-**infected mice. Rag1^–/–^ and Rag1^–/–^Mincle^–/–^ mice were subcutaneously administered 1 × 10^7^ CFU of *M. leprae* Thai53 or PBS in the footpad. Footpad samples were processed at 12 months postinfection. The number of acid-fast bacteria recovered from infected footpads was determined by RT-PCR amplifying an *M. leprae*-specific region. (b) Fite’s staining of footpad sections. Scale bar, 0.5 mm. (c) Fite’s staining of footpad skin. Scale bar, 50 μm. (d) Percentages of bacterially infected skin areas. (e) Fite’s staining of bone marrow. The area enclosed by a dotted line indicates the region of interest in the bone marrow. Scale bar, 100 μm. (f) Percentages of bacterially infected bone marrow tissue. (g) The footpad thickness was measured at 12 months postinfection, and the values were calculated as (footpad thickness after challenge) – (footpad thickness before challenge). (h) mRNA expression levels of *Nos2* of infected mice. At least eight (infected group) or three (control group) mice for one genotype were used in three independent experiments. An unpaired two-tailed Student’s *t* test was used for statistical analyses. *, *p* < 0.05; **, *p* < 0.01; and ***, *p* < 0.005.

To investigate the physiological relevance of the recognition, we performed *M. leprae* infection experiments in mice. As mice are resistant to *M. leprae*, we utilize mice lacking acquired immunity as recipient hosts.^27,28^ Mincle-deficient or -sufficient mice on an immunocompromising Rag1-deficient background were infected with *M. leprae*, and the outcome of the infection was evaluated after 12 months. The bacterial burden at the infection site was significantly higher in Mincle^–/–^ mice (Figure 4a), suggesting that Mincle plays a crucial role in protection. Histological analysis using Fite’s staining further confirmed this effect (Figure 4b–f), as the number of globi was increased in the skin of Mincle^–/–^ mice (Figure 4c and Table S1). Furthermore, *M. leprae* also infiltrated bone marrow tissues in Mincle^–/–^ mice (Figure 4e,f), suggesting that severe dissemination occurred in the absence of Mincle. As a consequence of the increased bacterial burden (Figure 4a) and cell infiltration (Table S2), the footpad thickness was significantly increased in Mincle^–/–^ mice (Figure 4g). Thus, Mincle plays a critical role in controlling *M. leprae* infection in the absence of acquired immunity in mice. We next investigated the immune reactions during infection. Most cytokines at the infected site were upregulated in Mincle^–/–^ mice, presumably due to exposure to the large number of bacteria. However, in Mincle^–/–^ mice, we observed a significant downregulation of *Nos2* (Figure 4h), a known Mincle downstream gene.^7,25^ These results suggest that Mincle plays an important role during *M. leprae* infection in mice through the induction of effector machinery such as NO-producing pathways, although we cannot exclude the contribution of other immune-stimulating *M. leprae* cell wall components in shaping the immune response.

### Structural Basis for Mincle Recognition of PGL-III

**Figure 5:**
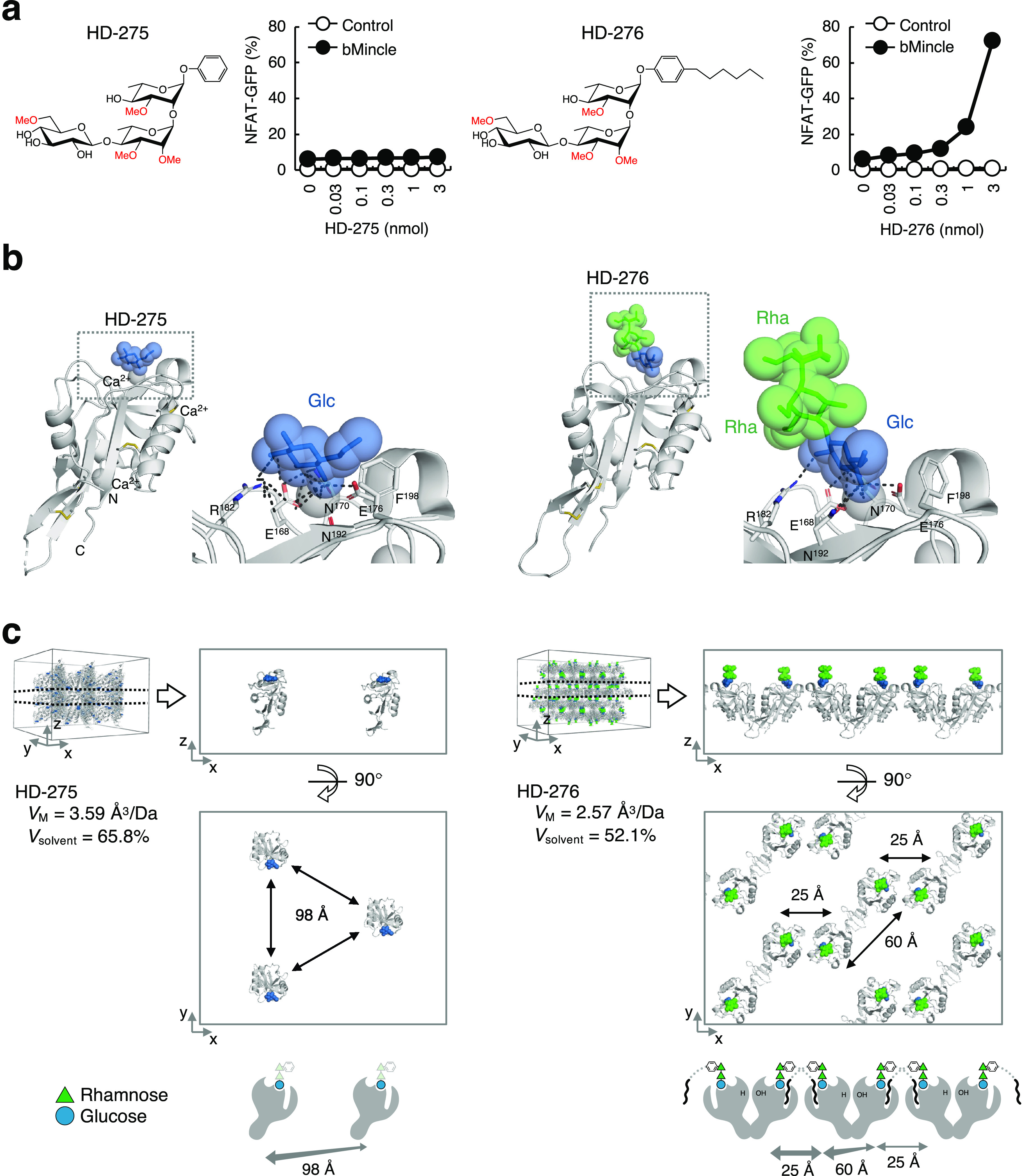
Structure of Mincle complexed with the trisaccharide moiety of PGL-III. (a) Structures of water-soluble PGL-III derivates, HD-275 and HD-276, and their reporter cell activity. Reporter cells expressing bovine Mincle + FcRγ (bMincle) or FcRγ alone (control) were stimulated with the indicated doses of PGL-III derivatives for 20 h and analyzed for GFP expression. Data are presented as the mean ± SD of triplicate assays and are representative of two independent experiments with similar results. (b) Overall structures and close-up views of ligand binding sites of Mincle CRD in complex with HD-275 (left panels) and HD-276 (right panels). Protein, carbohydrate, and calcium ions are shown in ribbon, stick, and sphere models, respectively. Blue and green spheres indicate the atoms composing glucose and rhamnose residues, respectively. Coordination and hydrogen bonds are depicted by gray dotted lines. Amino acid residues which interact with trisaccharide are shown by stick models and are labeled. (c) Crystal packings of the Mincle–HD-275 complex (left panels) and Mincle–HD-276 complex (right panels). 3D views of two crystal packings are shown in ribbon models. The Matthews coefficient (*V*_M_) and solvent content (*V*_solvent_), which reflect the “density” of crystallographic packing, are shown for each complex. Extracted layers of crystal packing of the two complexes are shown from horizontal (upper panels) and vertical (middle panels) viewpoints. Mincle–ligand complexes located in the same plane are shown. Schematic representations of HD-275 and HD-276 complexes (bottom panels). In the HD-275 complex, only the terminal glucose residues were assigned.

To further characterize the binding of PGL-III to Mincle, we synthesized water-soluble PGL-III derivatives, with or without truncated lipid tails (Figure 5a), which could be accessed via the same strategy of a late stage aglycone introduction by means of a Sonogashira reaction. Notably, the lipid-containing trisaccharide (HD-276) but not the trisaccharide without the lipid tail (HD-275) possessed ligand activity in reporter cells (Figure 5a). While structures of Mincle complexes with disaccharide-based ligands have been reported,^29,30^ those with trisaccharide ligands are unprecedented. We therefore attempted to obtain crystals using the recombinant Mincle carbohydrate recognition domain (CRD) in the presence of the trisaccharide ligands. Mincle–HD-275 and Mincle–HD-276 complexes formed differently shaped crystals under identical crystallization conditions (Figure S4a), implying that these two complexes were assembled in distinct modes. The crystals were analyzed by X-ray synchrotron radiation, and the structures were determined at 2.6 Å (Mincle–HD-275) and 2.4 Å (Mincle–HD-276) resolution (Figures 5b and S5, Table S3, and PDB 8HB5 and 8H4V) and are the first reports of Mincle structure in a complex with a trisaccharide ligand. These structures reveal that the terminal glucose of HD-275 and HD-276 interacts with the primary sugar binding site of Mincle, with the two rhamnose residues having no apparent interaction with the receptor (Figure 5b). In both the Mincle–HD-275 and Mincle–HD-276 complexes, the glucose C-2-OH group forms a hydrogen bond with R182. The C-3- and C-4-OH groups coordinate the calcium ion in a bidendate fashion and form hydrogen bonds with E168, N170, E176, and N192. *O*-Methylation of the C-3-OH (as in PGL-I) will lead to the loss of these crucial interactions, leading to a loss of ligand activity (Figure 3a). In addition to the favorable interactions of the C-3 and C-4-hydroxyls, the C-6-*O*-Me group lies along F198, which allows for a hydrophobic interaction (Figure 5b). The modes of interaction of the methylated glucose residues in HD-275 and HD-276 with Mincle were almost identical. Thus, Mincle interacts with only the single terminal sugar of the PGL-III trisaccharide moiety, in sharp contrast to the known disaccharide ligands such as trehalose (Figure S4b, PDB 4ZRW).^30^ The electron density of two rhamnose residues was clearly detected in the Mincle–HD-276 complex but not in the Mincle–HD-275 complex (Figure S4c), suggesting that the alkyl chain of HD-276 may contribute to the stabilization of the ligand–receptor complex and, presumably, a receptor–receptor interaction. Given that the Mincle–HD-276 complex is the first example of a Mincle structure bound to an agonistic ligand, we compared its crystal packing with that of Mincle–HD-275. The Mincle–HD-275 complex shows loose packing, similar to the reported structure with nonagonistic trehalose (Figure S4d, PDB 4ZRW),^30^ whereas the Mincle–HD-276 complex shows tight packing (Figure 5c). Indeed, the distances between Mincle molecules on the same layers were 98 Å and 25/60 Å for Mincle–HD-275 and Mincle–HD-276, respectively (Figure 5c). Interestingly, the alkyl chain of HD-276 appeared to bind to the hydrophobic lipid-binding groove of adjacent Mincle molecules (Figures 5c and S4e). Thus, it appears that the trisaccharide with an alkyl chain has the potential to multimerize Mincle molecules, and this can occur even under membrane-free conditions.

## Discussion

In the present study, we report that *M. leprae* produces an immunostimulatory intermediate in the PGL-I biosynthesis pathway, PGL-III, which acts as a noncanonical ligand for Mincle. The identification of this noncanonical Mincle ligand has provided a novel structure–activity relationship. The affinity of Mincle for the saccharide moiety of PGL-III has been shown in a glycan array experiment.^31^ In this experiment, the activity of the PGL-III trisaccharide having a short lipid chain was significantly lower than that of TDM, but this could be explained by the surface-bound nature of the ligand, obstructing optimal binding interactions. In contrast to previously identified Mincle ligands^26^ featuring mono- or disaccharide moieties, PGL-III is a unique ligand containing a trisaccharide. In line with known Mincle ligands,^26^ the equatorial hydroxy residues at the C-3 and C-4 of the terminal sugar are conserved in PGL-III (Figure 2a). This study thus extends and further defines the requirements of Mincle ligands and indicates that Mincle can recognize a wider variety of glycolipids than previously thought. Furthermore, this extends the spectrum of target pathogens as well as potential self-components. The mechanism of alkyl chain-mediated aggregation, which was observed in Mincle–HD-276 crystals, was implied by previous structural studies.^30^ One of the limitations of this structural study is that we used truncated glycolipids designed for crystallization, which are much shorter than natural PGL-III. Further structure–activity studies will uncover more detailed activation mechanisms for Mincle, as well as the unique mode of PGL-III binding.

Mincle induces *Nos2* expression and NO formation,^7^ which is a major effector mechanism contributing to protective immunity against *M. leprae*.^25^ Although PGL-I is abundant,^11^ the amount of PGL-III is limited in normal *M. leprae*,^22^ suggesting that persistent *M. leprae* may limit this immunoreactive intermediate such that it is “invisible” to sensors of the host immune system. As Mincle binds tightly to the C-3-OH of the terminal glucose in PGL-III, *O*-methylation of this position will block this interaction and convert it to immune-inactive PGL-I. Thus, the methyltransferase responsible for the methylation of PGL-III could be a therapeutic target against *M. leprae* infection, as inhibition should lead to the accumulation of PGL-III. A pathological role of PGL-I during infection has also been reported,^21,32^ and the reduction of this virulent factor^33^ could also be beneficial for the host. Several antileprosy drugs have been developed,^1^ but side effects^34,35^ and drug-resistant strains have been reported.^36^ Since the inhibition of PGL-I biosynthesis is a different mode of action in comparison to the currently approved drugs, this approach could provide a therapeutic option together with other therapies.

One of the limitations of the mouse model of leprosy infection is that infections cannot be established in immunocompetent mice;^28,37^ therefore, the role of Mincle was investigated in this study on a Rag1-deficient background, which lacks acquired immunity. However, *M. leprae* establishes persistent infections even in the presence of acquired immunity in some species, such as humans and nine-banded armadillos.^1,38^ We found that armadillos possess a Mincle orthologue that can recognize PGL-III with an efficiency similar to those of mouse and human Mincle (Figure S6). Now that *in vivo* infection models have been established in armadillos,^39−42^ it will be intriguing to define the role of Mincle during *M. leprae* infection in the presence of acquired immunity, for example, by using blocking antibodies. Such analyses will translate these findings to the human clinical setting.

On the other hand, the strong immunostimulatory activity of PGL-III may be involved in the characteristic pathologies observed in leprosy. The “leprosy reaction” is an acute local inflammation observed in some infected patients causing disabilities; however, its etiology is unclear. The leprosy reaction could be observed during or after treatment with antibacterial drugs.^43,44^ It is possible to speculate that these drugs may disrupt the balanced biosynthetic pathway that normally limits PGL-III, thereby triggering a potent immune reaction through the accumulation of PGL-III. This concept warrants further investigation.

Currently, the detection of serum antibodies against PGL-I is used to diagnose *M. leprae* infections.^45,46^ Anti-PGL-III antibodies have also been detected in some patients,^22^ suggesting that these patients were exposed to a certain amount of PGL-III during infection. Thus, the presence of anti-PGL-III could be a biomarker of the “leprosy reaction”. Since PGL-III is uniquely recognized by Mincle to exert its potent dose-dependent activity (Figure 3c), the concept of Mincle antagonists that interfere with PGL-III binding while not interfering with the recognition of other Mincle ligands is a promising chemical approach to suppressing the hyperimmune response observed during the leprosy reaction.

## Supporting Information Available

GFP expression of reporter cells expressing various CLRs stimulated with 16 fractions derived from high-performance thin layer chromatography separation of crude lipids from *M. tuberculosis*, *M. smegmatis*, and *M. leprae*; speculated biosynthetic pathway of PGL-I in recombinant *M. marinum*; HPTLC analysis of synthetic PGL-III and extracted Mincle-active lipids from *M. leprae* and *M. marinum*; structural studies of Mincle–HD-275, Mincle–HD-276, and Mincle–trehalose complexes; stereoviews of electron density maps of Mincle complexes with HD-275 and HD-276; reactivity of armadillo Mincle to PGL-III; histopathological findings in *M. leprae*-infected mice and in mice inoculated with *M. leprae* or PBS as controls (HE stain); and data collection and refinement of the crystallographic analysis of the complexes of Mincle with the PGL-III-derivatives (PDF)
