# Supplementary material for: PGL-III, a Rare Intermediate of Mycobacterium leprae Phenolic Glycolipid Biosynthesis, Is a Potent Mincle Ligand
Source: ACS Cent Sci. 2023 Jul 12;9(7):1388–99. doi: 10.1021/acscentsci.3c00040 (PMC10375886; doi:10.1021/acscentsci.3c00040)

Supporting info to:

PGL-III, a rare intermediate of *Mycobacterium leprae* phenolic glycolipid biosynthesis, is a potent Mincle ligand

Shigenari Ishizuka[1,2,#], J. Hessel M. van Dijk[3,#], Tomomi Kawakita[4], Yuji Miyamoto[4], Yumi Maeda[4], Masamichi Goto[5], Guillaume Le Calvez[6], L. Melanie Groot[3], Martin D. Witte[6], Adriaan J. Minnaard[6], Gijsbert A. van der Marel[3], Manabu Ato[4], Masamichi Nagae[1,2], Jeroen D. C. Codée[3,*], Sho Yamasaki[1,2,7,*]

[1] Department of Molecular Immunology, Research Institute for Microbial Diseases, Osaka University, 3-1 Yamadaoka, Suita, Osaka 565-0871, Japan

[2] Laboratory of Molecular Immunology, Immunology Frontier Research Center, Osaka University, 3-1 Yamadaoka, Suita, Osaka 565-0871, Japan

[3] Leiden Institute of Chemistry, Leiden University, Einsteinweg 55, 2333 CC Leiden, The Netherlands

[4] Department of Mycobacteriology, Leprosy Research Center, National Institute of Infectious Diseases, 4-2-1 Aobacho, Higashimurayama, Tokyo 189-0002, Japan

[5] Department of Pathology, Kagoshima University Graduate School of Medical and Dental Sciences, 8-35-1 Sakuragaoka, Kagoshima 890-8544, Japan

[6] Stratingh Institute for Chemistry, Nijenborgh 7, 9747 AG Groningen, The Netherlands

[7] Center for Infectious Disease Education and Research, Osaka University (CiDER), 3-1 Yamadaoka, Suita, Osaka 565-0871, Japan

[#]These authors contributed equally.

[*]Corresponding authors
E-mail: yamasaki@biken.osaka-u.ac.jp; jcodee@chem.leidenuniv.nl

Table of contents

1. Supplementary figures
    - Figure S1. Crude lipid CLR heatmap S3
    - Figure S2. Speculated pathway of *M. marinum* PGL-I biosynthesis S4
    - Figure S3. HPTLC analysis of Synthetic PGL-III together with Mincle active *M. leprae* lipids and recombinant *M. marinum* S5
    - Figure S4. Crystallization of HD-275 and HD-276 and comparison to trehalose S6
    - Figure S5. Stereo views of electron density maps S8
    - Figure S6. Stimulation of armadillo Mincle S9
2. Supplementary tables
    - Table S1. Histopathological findings in *M. leprae* infected mice (Fite's stain) S10
    - Table S2. Histopathological findings (HE stain) S11
    - Table S3. Data collection and refinement of the crystallographic analysis of Mincle and PGL-analogue complexes S12

3. Materials and Methods S13
4. Synthetic experimentals
    - General procedures S19
    - Building block synthesis S21
    - Oligosaccharide synthesis S26
    - Final PGL assembly S30
    - PGL-III analogues S40
5. References S42
6. NMRs of newly synthesized and isolated compounds S44

Supplementary figures

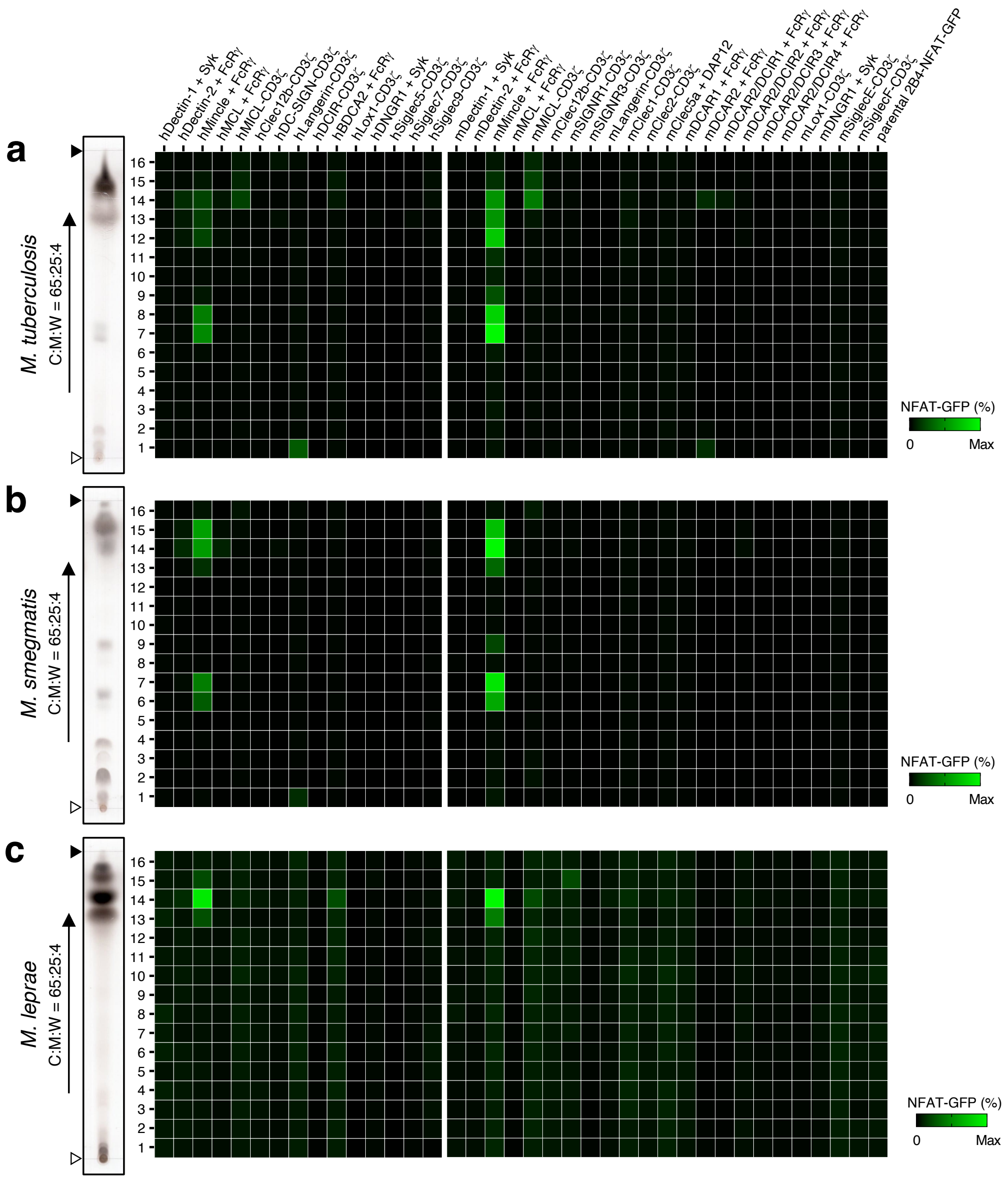

Figure S1. Mincle recognizes lipid fractions extracted from three different mycobacteria.
a–c, Crude lipids of *M. tuberculosis* (a), *M. smegmatis* (b), and *M. leprae* (c) extracted using C:M (2:1, v/v) were fractionated into 16 fractions by HPTLC using C:M:W (65:25:4, v/v/v). Open and closed arrowheads beside the TLC panels denote the origin and the solvent front, respectively. Reporter cells expressing various CLRs were stimulated with each lipid fraction for 20 hr. GFP expression is shown as a heatmap. Data are presented as the mean of duplicate assays and representative of two independent experiments with similar results.

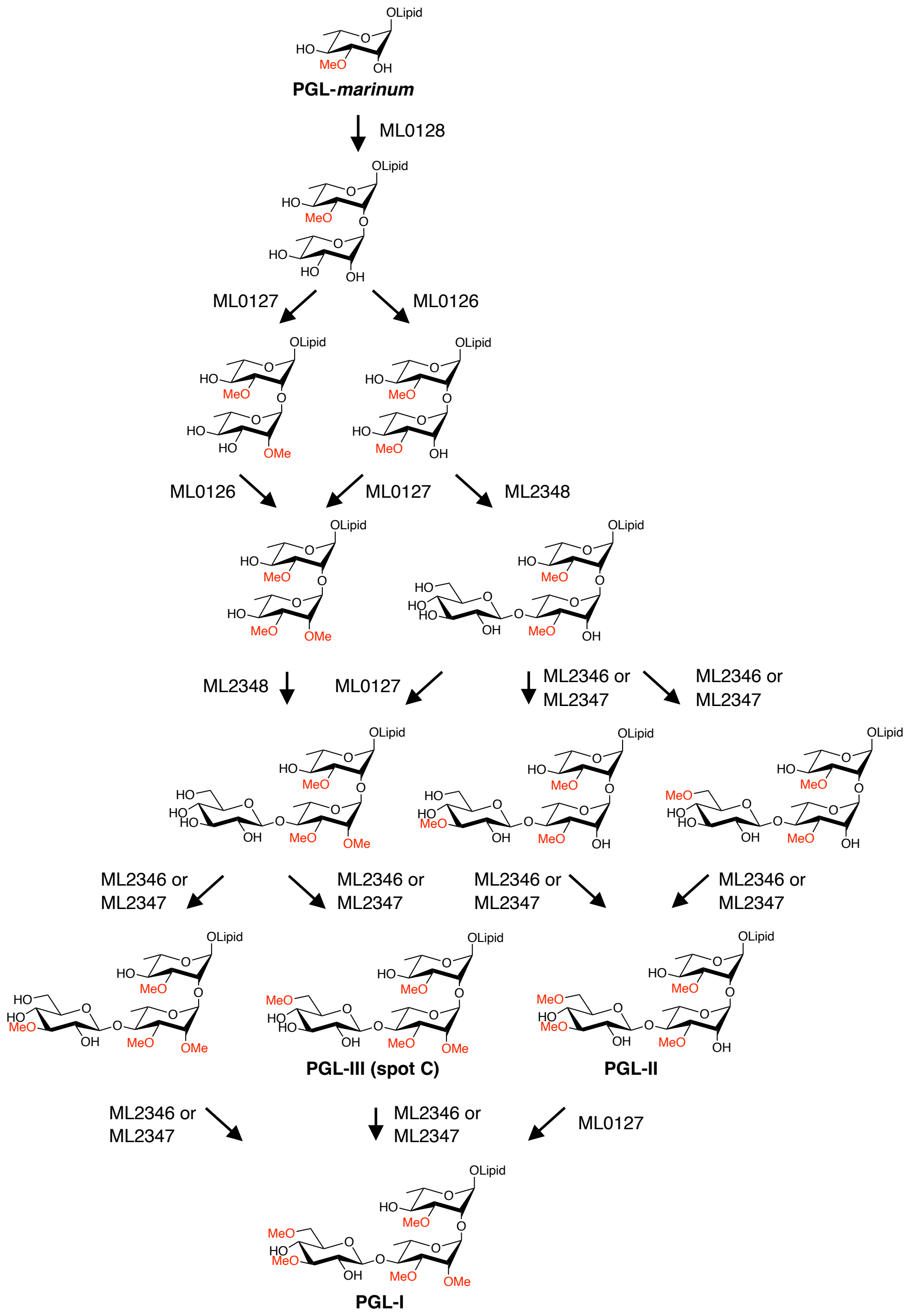

Figure S2. Hypothesized PGL-III biosynthetic pathway in recombinant *M. marinum*. Unmodified *M. marinum* possesses PGL with a single rhamnose (3-*O*-Me-Rha). Introducing a series of enzymes involved in PGL-I synthesis from *M. leprae* (ML0126, ML0127, ML0128, ML2346, ML2347, and ML2348; Figure 2a) allows production of PGL-I and its intermediate metabolites, including PGL-III, in *M. marinum*. "Lipid" means the phenolphthiocerol dimycocerosate moiety detected in *M. marinum*.

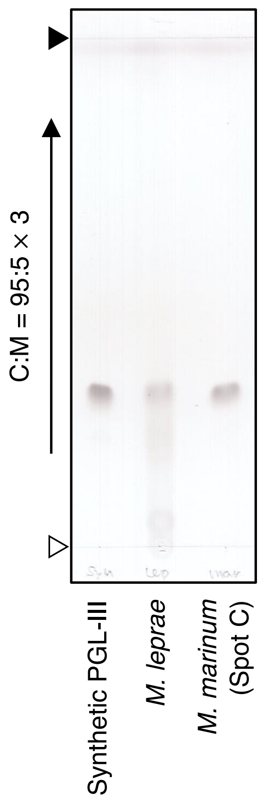

Figure S3. Mincle-activating lipid extracted from *M. leprae* is hypothesized to be PGL-III. Synthetic PGL-III, and the Mincle-activating lipids from *M. leprae* and recombinant *M. marinum* were analyzed by HPTLC using C:M (95:5, v/v, three runs). Open and closed arrowheads beside TLC panels denote the origin and solvent front, respectively.

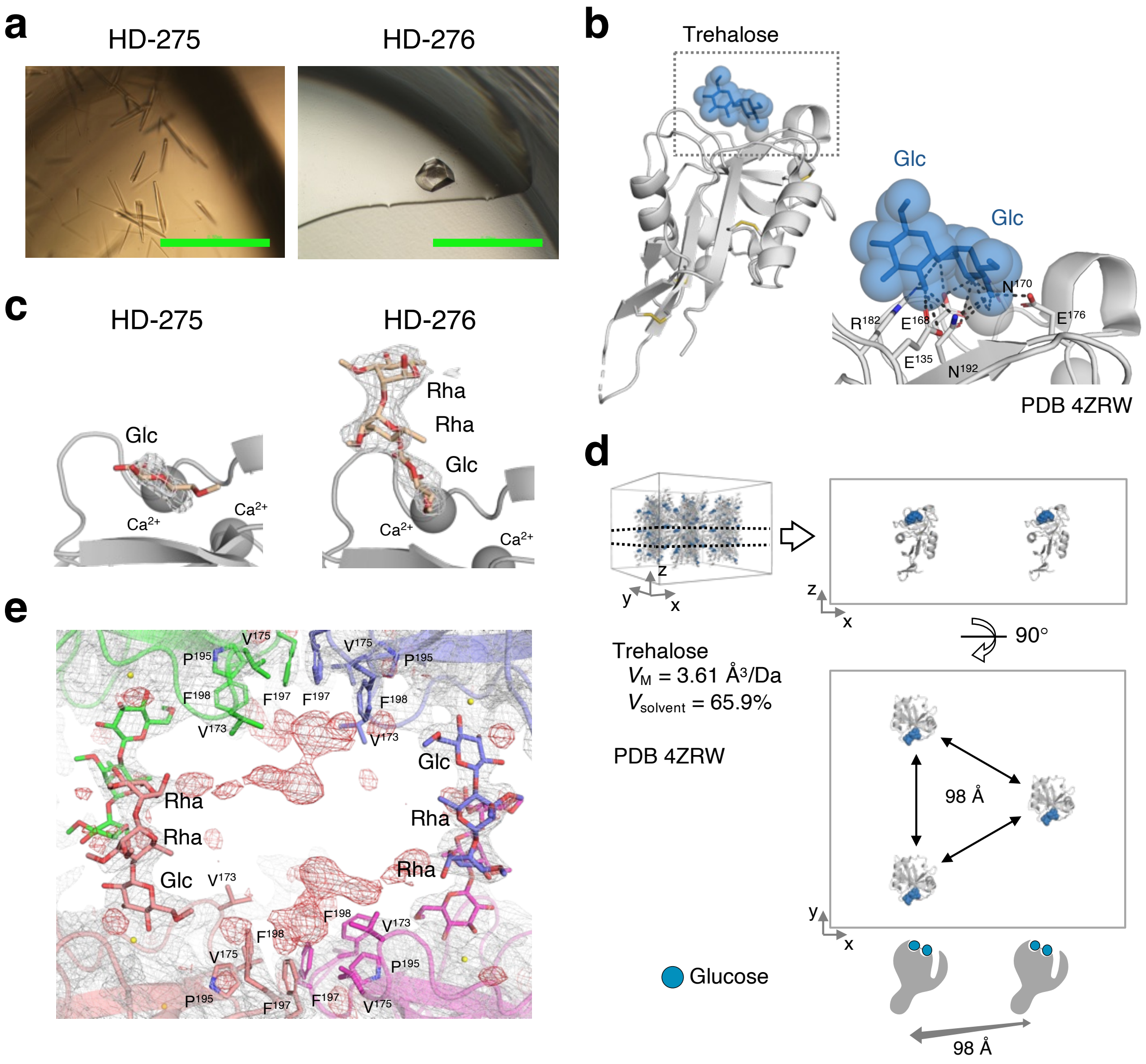

Figure S4. PGL-III is distinct from the canonical Mincle ligand, trehalose, and may induce assembly of Mincle via acyl chains.

a, Crystal(s) of Mincle–HD-275 and Mincle–HD-276 complexes. Scale bar = 0.5 mm.

b, Overall structure of Mincle CRD in complex with trehalose (PDB 4ZRW)[1]. Protein, carbohydrate, and calcium ions are shown in ribbon, stick, and sphere models, respectively (left panel). Interaction between Mincle and trehalose. Coordination and hydrogen bonds are depicted in gray dotted lines. Amino acid residues which interact with trehalose are shown in stick models and labeled (right panel).

c, Omit map contoured at 3σ levels (gray mesh) is shown around ligand binding sites of HD-275 (left panel) and HD-276 (right panel).

d, Crystal packing of the Mincle–trehalose complex. 3D views of two crystal packings are shown in ribbon models. Matthews coefficient (V_M) and solvent content ($V_{solvent}$), which reflect the "density" of crystallographic packing, are shown. Extracted layers of crystal packing of the two complexes are shown from horizontal (upper panel) and vertical (middle panel) viewpoints. Mincle–trehalose

complexes that are located in the same plane are shown. Schematic representation of Mincle–trehalose complexes (bottom panel).

e, Electron density map around hydrophobic groove of Mincle in the HD-276 complex. The $2F_{obs} - F_{calc}$ map contoured at 1.5σ levels is shown in grey mesh. The $F_{obs} - F_{calc}$ map contoured at 3σ is shown in red mesh. Unassigned electron density which may correspond to the acyl chain of HD-276 is observed near the rhamnose residue of the trisaccharide. In crystal packing, the hydrophobic groove of two Mincle molecules forms a large patch. This electron density map is also close to the hydrophobic patch of Mincle. Sugar residues and amino acid residues in the hydrophobic groove are shown in stick models and labeled.

Mincle–HD-275 complex

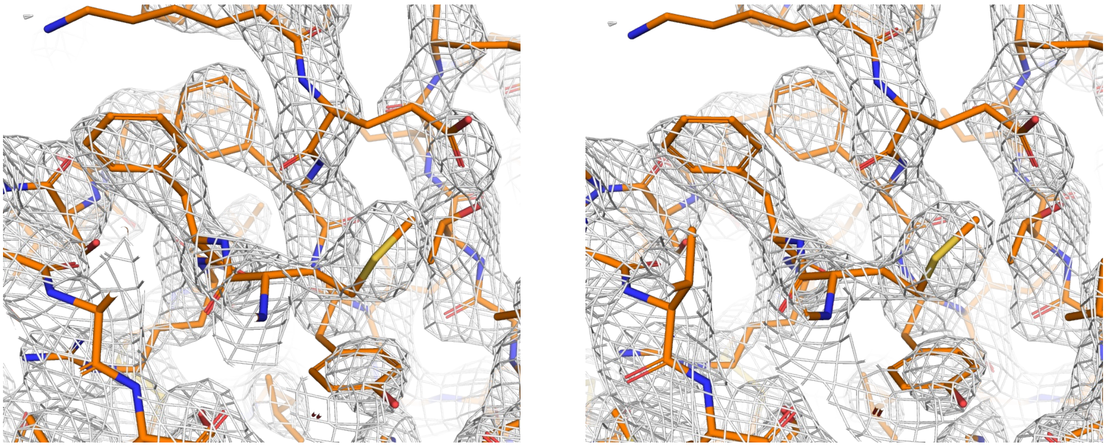

Mincle–HD-276 complex

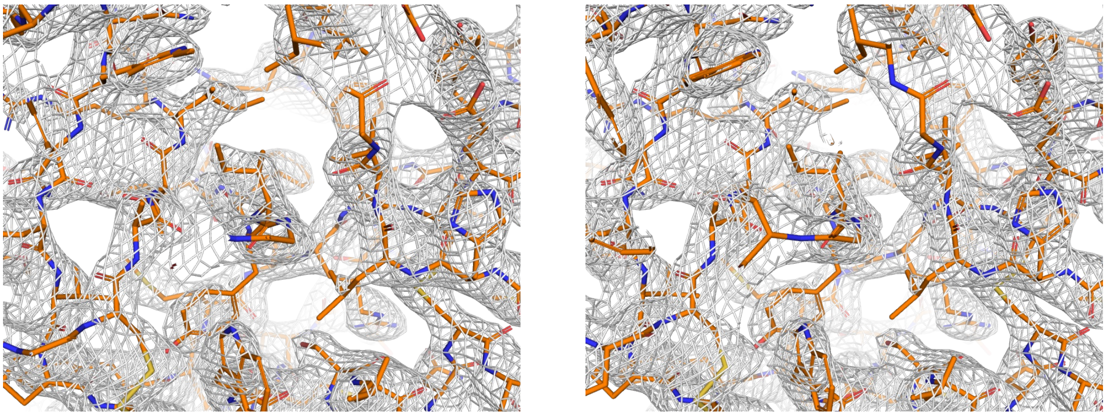

Figure S5. Stereo views of electron density maps of Mincle complexes with HD-275 and HD-276. Stereo views of $2F_{obs} - F_{calc}$ electron density map contoured at 1.5σ levels of HD-275 complex (upper panels) and HD-276 complex (lower panels) are shown.

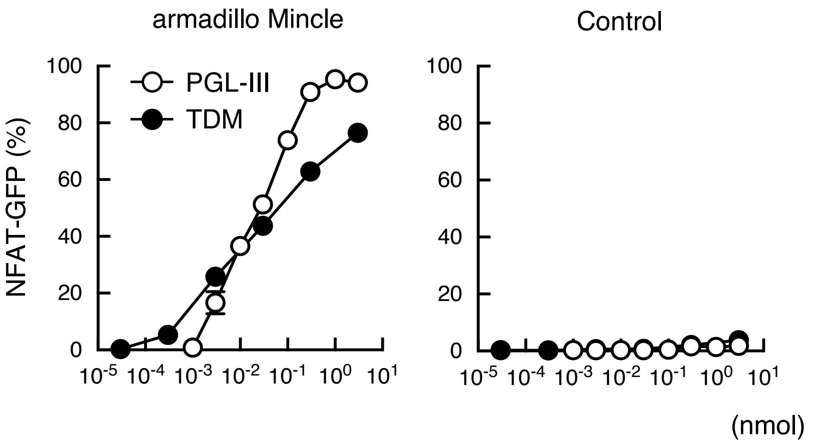

Figure S6. Armadillo Mincle recognizes PGL-III.

Reporter cells expressing armadillo Mincle + FcRγ (armadillo Mincle) or FcRγ alone (Control) were stimulated with the indicated doses of PGL-III for 20 hr and analyzed for GFP expression.

Supplementary tables

Table S1. Histopathological findings in *M. leprae*-infected mice (Fite's stain)

Stain	Inoculation	Mouse	Sex	n	Indicator (on scale 0 to 2)		
					**Globi**	**Acid-fast bacilli**	**Bone marrow infiltration**
Fite	*M. leprae*	Rag1$^{-/-}$	F	4	1	1	1
	M. leprae	Rag1$^{-/-}$	M	4	1	1	1
	M. leprae	Rag1$^{-/-}$ Mincle$^{-/-}$	F	4	2	2	2
	M. leprae	Rag1$^{-/-}$ Mincle$^{-/-}$	M	5	2	2	2

Table S2. Histopathological findings (HE stain)

					Indicator (on scale 0 to 2)		
**Stain**	**Inoculation**	**Mouse**	**Sex**	**n**	**Macrophages**	**Lymphocytes**	**Neutrophils**
HE	PBS	$Rag1^{-/-}$	F	3	0	0	0
	PBS	$Rag1^{-/-}$ $Mincle^{-/-}$	F	3	0	0	0
	PBS	$Rag1^{-/-}$ $Mincle^{-/-}$	M	3	0	0	0
	M. leprae	$Rag1^{-/-}$	F	4	1	0	1
	M. leprae	$Rag1^{-/-}$	M	4	1	1	1
	M. leprae	$Rag1^{-/-}$ $Mincle^{-/-}$	F	4	2	1	1
	M. leprae	$Rag1^{-/-}$ $Mincle^{-/-}$	M	5	2	1	1

Table S3. Data collection and refinement of the crystallographic analysis of complexes of Mincle with PGL-derivates.

	**Mincle–HD-275 complex**	**Mincle–HD-276 complex**
**Data collection**		
Space group	$P3_121$	$P3_112$
Cell dimensions		
a, b, c (Å)	97.5, 97.5, 46.5	75.3, 75.3, 111.4
α, β, γ (°)	90, 90, 120	90, 90, 120
Resolution (Å)	48.8-2.6 (2.72-2.6) *	37.7-2.4 (2.49-2.4) *
R_{merge} (%)	38.2 (373.8)	33.0 (374.6)
$I/\sigma I$	14.1 (1.8)	24.4 (1.3)
Completeness (%)	100 (100)	99.5 (100)
Redundancy	50.3 (50.5)	51.5 (53.4)
**Refinement**		
Resolution (Å)	2.6	2.4
No. reflections	8,053	14,309
R_{work} / R_{free}	29.6/33.5	25.6/28.9
No. atoms		
Protein	1,194	2,371
Ligand/ion	16	78
Water	3	0
B-factors		
Protein	50.9	95.2
Ligand/ion	58.3	107.0
Water	48.4	-
R.m.s. deviations		
Bond lengths (Å)	0.022	0.005
Bond angles (°)	0.87	0.79

*Number of xtals for each structure should be noted in footnote.

*Values in parentheses are for highest-resolution shell.

Materials and Methods

Mice

Mincle-deficient (Mincle$^{-/-}$) mice[2] were backcrossed for at least 16 generations with C57BL/6. C57BL/6 mice for immunization experiment were purchased from CLEA Japan Inc., Tokyo, Japan. These mice were maintained in a filtered-air laminar-flow enclosure and given standard laboratory food and water *ad libitum*. Four-week-old B6;129S7-Rag1tm1Mom/J (Rag1$^{-/-}$) mice were purchased from the Jackson Laboratory. Mincle$^{-/-}$ mice were back-crossed with Rag1$^{-/-}$ mice to obtain the double knockout (Rag$^{-/-}$Mincle$^{-/-}$) mice, which were validated by genotyping. These mice which produce no mature T and B cells were strictly inbred in pathogen-free individually ventilated cage housing at the animal facility of National Institute of Infectious Diseases, Leprosy Research Center. All animal protocols were reviewed and approved by the Animal Care and Use Committee of the Research Institute for Microbial Diseases, Osaka University (Biken-AP-R03-17-0) or the Animal Research Committee of Experimental Animals under the National Institute of Infectious Diseases (No.118028, No.118012), and animal experiments were conducted according to their guidelines.

Bacteria

Nude mice (BALB/cAJcl-nu/nu) for propagation of *M. leprae* Thai53 were obtained from CLEA Japan Inc., Tokyo, Japan. 1 × 10^7 *M. leprae* was inoculated into the hind footpads and nine months after inoculation, footpads were processed to obtain highly viable bacilli for the experiments[3]. *M. tuberculosis* H37Rv was obtained from National Hospital Organization Toneyama National Hospital. *M. smegmatis* mc^{2}155 was cultured as previously described[3,4].

Infection

Five to six-week-old Rag1$^{-/-}$ and Rag1$^{-/-}$Mincle$^{-/-}$ mice were inoculated with 1 × 10^7 freshly prepared *M. leprae* into the hind footpads. The thickness of the footpad was enumerated using a Vernier caliper every month. 12 months post inoculation, mice were sacrificed for histopathological examination and evaluation of *Nos2* expression.

Reagents

TDM (T3034) and LPS (L4516) were purchased from Sigma-Aldrich. TDB (890808P) was purchased from Avanti Polar Lipids. Low-endotoxin OVA (015-24731) was from Fujifilm-Wako. Anti-CD3ε (clone: 2C11) mAb was from MBL. HRP-labeled anti-OVA pAb was from GE Healthcare. The ELISA kits for TNF, IL-6, IFN-γ, hTNF, and hIL-6 were from BD Biosciences.

Cells

2B4-NFAT-GFP reporter cells expressing various CLRs were prepared as previously described[5]. Bovine Mincle (NCBI accession number: XP_010803863) and nine-banded armadillo Mincle (NCBI accession number: XP_004455316) were synthesized by Integrated DNA technologies, Inc. BMDMs and hMoDCs were prepared as previously described[6,7]. Briefly, for preparation of BMDMs, bone marrow cells from wild type (C57BL/6) and Mincle$^{-/-}$ mice were cultured in DMEM media supplemented with 10% FCS (Nichirei), 2-mercaptoethanol, antibiotics, and 10% L929 culture supernatant as a source of M-CSF for seven days. For preparation of hMoDCs, CD14^{+} monocytes purified from peripheral blood of healthy donors using LSS (Nacalai Tesque) gradient centrifugation and anti-human CD14 Microbeads (Miltenyi Biotech) were cultured in RPMI-1640 media supplemented with 10% FCS, non-essential amino acid, antibiotics, hGM-CSF (10 ng/ml) (Peprotech), and hIL-4 (10 ng/ml) (Peprotech) for seven days. The institutional review boards of Osaka University approved blood draw protocols for healthy individuals (approval number 29-4-10).

Lipid extraction and purification

The liver of *M. leprae* Thai53-infected armadillo was homogenized in Hanks' balanced salt solution (HBSS) as previously described[8,9] and resultant *M. leprae* cells were harvested by centrifugation. The recombinant *M. marinum* was cultured in 7H9 medium supplemented with OADC enrichment and 0.2% glycerol, and grown cells were harvested by centrifugation. For lipid extraction from *M. leprae* and *M. marinum*, cells were resuspended with chloroform:methanol (2:1, v/v) at room temperature for overnight, and the supernatant including the total lipid extract was evaporated. The supernatant obtained by this procedure included a minimum amount of water-soluble compounds. For enrichment of the PGL-related glycolipids, the total lipid extract was mixed with excess volume of acetone and the supernatant was obtained. For fractionation and purification, the lipid extracts were separated by HPTLC (Merck) and visualized by copper(II) acetate-phosphoric acid (180°C, 15 min) staining. Lipids-absorbing silica gel was scraped from the plate, and lipids were eluted by chloroform:methanol (2:1, v/v). The eluted lipids were filtered using Millex-LG (0.2 μm, Millipore) to remove silica gel contamination.

Generation of recombinant M. marinum

For stable expression of the *M. leprae*-derived PGL-I biosynthetic genes in heterogenous bacteria, *M. marinum*, the related gene cluster was divided into two regions and cloned into two different vectors, pMV301a (chromosomal integrative vector) and pMV261a (plasmid vector)[10]. The ML2346c–ML2348 region was amplified from *M. leprae* DNA using following primers: ML2346 sense 5'-CCGGGATCCCATGGCATTATGGAGAAGCCT-3' and anti-sense 5'-CCCAAGCTTTCAATCCAGCCGGGCGTGTAAG-3'; ML2347 sense 5'-CCCAAGCTTCGCTGAAACATTACATGGAAT-3' and ML2348 anti-sense 5'-

CGGCTTAAGCTCAACAGTCCTGATACCGAGTTC-3'. The PCR products were digested with each restriction enzyme and inserted into BamHI, HindIII, and AflII sites of pMV301a to give pMV2346–2348. ML0126–ML0128 region was amplified using following primers: ML0126 sense 5'-CCGGGATCCCATGAGAGCAGCCGAAGCTTCGAAG-3' and ML0126 antisense 5'-CCGGAATTCCAATATAAATACTGATGTTGCTTC-3'; ML0127 sense 5'-CCGGAATTCTGCTACGGACAATGTGTTCGCATTG-3' and ML0128 anti-sense 5'-CGGCTTAAGCTAGGTCGTAGTTTCGTTTTGTCTGG-3'. The PCR products were digested with each restriction enzyme and inserted into BamHI, EcoRI, and AflII sites of pMV261a to give pMV0126–0128. *M. marinum* 927 strain was transformed with pMV0126–0128 by electroporation and cultivated on 7H10 agar plate with OADC enrichment, containing 25 μg/ml kanamycin. Grown colonies were subsequently transformed with pMV2346–2348 and cultivated on 7H10 agar plate with OADC enrichment, containing 25 μg/ml kanamycin and 50 μg/ml hygromycin.

MALDI-TOF-MS analysis

MALDI-TOF-MS was performed on purified lipid fractions by using ultrafleXtreme mass spectrometer (Bruker). The matrix was 140 mg/ml 2,5-dihydroxybenzoic acid in acetonitrile-trifluoroacetic acid in 0.1% acetonitrile:trifluoroacetic acid (30:70, v/v), and analyzed in the positive mode.

NMR analysis

The isolated spot C (100 μg) was analyzed with a Bruker AV-850 using ^{1}H-NMR (ns = 256), HH-COSY (ns = 8), HSQC (ns = 32), HMBC (ns = 32), and ^{13}C-APT NMR (ns = 10000) experiments. The analysis of all spots can be found in the supporting information.

In vitro stimulation assay

To stimulate the cells, each lipid was diluted in isopropanol and the 20 μl dilutions were added into each well of the 96-well plate, followed by evaporation of the solvent. Prior to stimulation, BMDMs were treated with LPS (100 ng/ml) and IFN-γ (20 ng/ml) (Peprotech) for 24 hr for M1-polarization[11]. LPS (100 ng/ml) was used as a positive control for BMDMs stimulation. The reporter activity of 2B4-NFAT-GFP cells was analyzed by Attune NxT flow cytometer (Thermo Fisher Scientific). For real-time PCR, PGL-III was diluted in isopropanol and the 100 μl dilutions were added into each well of the 24-well plate, followed by evaporation of the solvent. Prior to stimulation, BMDMs were treated with IFN-γ (10 ng/ml) (Peprotech) for 4 hr.

Real-time PCR for BMDMs

Total RNA from BMDMs was extracted and isolated using RNA Basic Kit (Nippon Genetics). cDNA was synthesized using ReverTra Ace qPCR RT Master Mix (Toyobo), according to the manufacturer's

protocol. Quantitative real-time PCR (qPCR) was performed by QuantStudio 5 (Applied Biosystems), using THUNDERBIRD Next SYBR qPCR Mix (Toyobo). Normalized gene expression levels were indicated as the ratio between the mean value for the target gene and that of the house keeping gene *Actb*. The primers used are as follows: *Nos2*; sense 5'-GCAGGTCTTTGACGCTCGGA-3' and anti-sense 5'-GGCCGACCTGATGTTGCCAT-3'.

Immunization and recall responses

Mice were immunized by subcutaneous injection at tail base with oil-in-water emulsions (mineral oil/Tween-80/PBS, 9:1:90, v/v/v) containing OVA (200 μg) with or without synthesized PGL-III (100 nmol) or TDB (100 nmol). Sera were collected from each mouse at indicated timepoints and analyzed for OVA-specific antibody levels by ELISA using HRP-conjugated goat anti-mouse IgG and sera pool from OVA/Alum-administered mice as a standard. The concentrations of each (sub)class in this sera pool was defined as 100 Unit. After 12 weeks post inoculation, mice were sacrificed and their inguinal lymph nodes were harvested. Harvested lymph nodes were homogenized and cells (5×10^5 cells/well) were stimulated with OVA at 0, 10, 30, 100, and 300 μg/ml. After 72 hr, the supernatant was collected and IFN-γ production was measured by sandwich ELISA according to the manufacturer's instructions.

Histological analysis

12 months after infection with *M. leprae*, the feet from infected mice were fixed in 10% buffered formalin, decalcified and embedded in paraffin. Cross-sections of the distal areas of the metatarsals of the infected foot were prepared. The sections were stained with Hematoxylin-eosin (HE) and Fite's acid fast stain. Individual images were acquired using a Zeiss Axiocam 208 color CMOS camera mounted on Olympus BX53 microscope. Acquired images from tissue sections had a resolution of 3840 × 2160 pixels, corresponding to 2.00 × 3.50-mm (4×), 400 × 700-μm (20×), 200 × 350-μm (40×) areas. The positively-stained area using Fite's stain was calculated by hue analysis with ImageJ software as previously described[12]. Briefly, three images of microscopic fields with abundant bacilli for each individual were obtained and processed hue images were analyzed for Fite's stain-positive area with automatically set threshold. The values were indicated as the percentage of the number of positive pixels per total pixels for footpad or to total bone marrow pixels. Histopathological severity was scored based on the number of globi, macrophages and lymphocytes, and neutrophil infiltration.

Analysis of bacterial burden and nitric oxide modulation in vivo

For quantification of the number of *M. leprae* from the tissues, the copy number of RLEP region in DNA fraction was determined by qPCR using KOD SYBR qPCR Mix (Toyobo). The primers used are as follows: sense 5'- TGCATGTCATGGCCTTGAGG-3' and anti-sense 5'-CACCGATACCAGCGGCAGAA-3'. Total RNA and DNA were simultaneously fractionated and

extracted from the *M. leprae*-infected footpad tissue using TRIzol (Thermo Fisher Scientific). For determination of the *Nos2* expression, cDNA was synthesized from RNA fraction using ReverTra Ace qPCR RT Master Mix (Toyobo), according to the manufacturer's protocol. qPCR was performed using THUNDERBIRD Next SYBR qPCR mixture (Toyobo). Normalized gene expression levels were indicated as the ratio to the mean value of the house keeping gene *Hprt*. The primers used are as follows: *Nos2*; sense 5'-AATCTTGGAGCGAGTTGTGG-3' and anti-sense 5'-CAGGAAGTAGGTGAGGGCTTG-3'.

Protein expression and purification

Expression constructs encoding the carbohydrate recognition domain of bovine Mincle were incorporated into pCold vector including 6× His-tag and a tobacco etch virus (TEV) protease cleavage site. The plasmids were transformed into *E. coli* BL21 competent cells Champion21 (Smobio). The protein expression was induced by the addition of 1 mM isopropyl-β-D-thiogalactopyranoside (IPTG) at 18°C for overnight. Cells were suspended with lysis buffer (50 mM Tris-HCl [pH 8.0] and 0.5 M NaCl) and disrupted with sonication. The insoluble fraction of the disrupted cells was resuspended with lysozyme-working solution (50 mM Tris-HCl [pH 8.0], 150 mM NaCl, and 0.4 mg/ml lysozyme) and incubated at room temperature for 30 min, followed by treatment with 25 μg/ml DNase I at 37°C for 1 hr. The insoluble fraction was washed twice with Triton wash buffer (50 mM Tris-HCl [pH 8.0], 0.1 M NaCl, 10 mM EDTA, and 0.5% [v/v] Triton X-100), and once with resuspension buffer (50 mM Tris-HCl [pH 8.0], 0.1 M NaCl, and 10 mM EDTA). The inclusion body was collected by centrifugation and was then solubilized by denature buffer (0.2 M Tris-HCl [pH 8.0], 6 M guanidine HCl, 10 mM EDTA, and 5 mM dithiothreitol) at 37°C. 70 mg of the solubilized protein was rapidly diluted with 1 litter of refolding buffer (0.1 M Tris-HCl [pH 8.0] and 0.4 M L-arginine, 1 mM calcium chloride, 5 mM reduced glutathione, and 0.5 mM oxidized glutathione) at 4°C. The diluted solution was further dialyzed with dialysis buffer (20 mM Tris-HCl [pH 8.0], 50 mM NaCl, and 1 mM calcium chloride) for 2 d. The dialyzed solution was applied onto 5 ml of nickel-nitrilotriacetic acid agarose (Fujifilm-Wako), and His-tagged Mincle was eluted with elution buffer (50 mM Tris-HCl [pH 8.0], 0.3 M NaCl, and 250 mM imidazole). After removal of His-tag by TEV protease, the eluted protein was concentrated and further applied to Superdex 75 (Cytiva) equilibrated with gel filtration buffer (20 mM Tris-HCl [pH 8.0], 0.1 M NaCl, and 1 mM calcium chloride). The Mincle fractions were concentrated up to 22.9 mg/ml by Vivaspin 500 (molecular weight cutoff, 10 kDa; Sartorius). The purity of the protein was assessed by SDS-PAGE and Coomassie brilliant blue staining.

Crystallization and data collection

All crystallization trials were performed by sitting drop vapor diffusion method. To make complex form of Mincle and the synthetic ligand HD-275 or HD-276, Mincle (final 4.58 mg/ml) and each ligand (final

1.0 mM and 11.58 mM, respectively) were mixed and left at 4°C overnight. Initial conditions were screened using Index (Hampton Research) and SG1 Screen (Molecular Dimensions). The best diffracted crystal was obtained under the condition of 0.1 M imidazole [pH 8.5], 0.2 M sodium acetate trihydrate, and 10% (w/v) polyethylene glycol (PEG) 8,000 at 20°C. Prior to X-ray diffraction experiments, crystals were soaked in the reservoir containing 20% ethylene glycol and each ligand, followed by flash cooling in liquid nitrogen. X-ray diffraction data sets were collected at the synchrotron radiation source at BL-17A in the Photon Factory (Tsukuba, Japan). All data sets were processed using the program XDS[13] and scaled using the program AIMLESS[14]. Phase determination was performed by molecular replacement method with program Molrep[15] using bovine Mincle CRD (PDB 4ZRW) as a search model. Model building was performed manually using the program COOT[16]. Atomic coordinates of the PGL trisaccharide were retrieved from PubChem database. Refinement was initially conducted using REFMAC5[17] and Phenix.refine of the Phenix program suite[18] for the final model. The stereochemical quality was assessed by MolProbity[19]. Data collection and refinement statistics are summarized in Table S3. Structure factors and atomic coordinates of the Mincle–HD-275 and Mincle–HD-276 complexes have been deposited in Protein Data Bank under the accession codes 8HB5 and 8H4V, respectively. Structural figures were depicted using COOT and PyMOL (The PyMOL Molecular Graphics System, Version 2.0, Schrödinger, LLC).

Statistical analysis

An unpaired two-tailed Student's t test was used for all the statistical analyses. Asterisks denote level of statistical significance (*, $p < 0.05$; **, $p < 0.01$; ***, $p < 0.005$).

Synthesis procedures

General procedures

All reactions were carried out in oven-dried glassware (80 °C). Prior to reactions, traces of water and solvent were removed by co-evaporation with toluene where appropriate. Reactions sensitive to air or moisture were carried out under N_2 atmosphere (balloon). Commercially available reagents and solvents (Aldrich Chemistry, Honeywell, Merck, Fischer Scientific, Biosolve, Fluka, VWR Chemicals, Acros Organics, Fluorochem, Brunschwig, Carbosynth) were used as received unless stated otherwise.
Solvents for reactions were reagent grade and dried by storage over flame dried 4 Å molecular sieves when needed. Tf_2O used in glycosylations was dried by distillation over P_2O_5 and stored under N_2 atmosphere in a Schlenk flask at -20 °C. Et_2O used for column chromatography was distilled before use and stored over iron filings. EtOAc used for column chromatography was distilled before use. NEt_3 used for Sonogashira couplings was distilled from KOH, degassed with N_2, and stored over KOH for a maximum of 24 hours. DMAP used for Steglich esterifications was recrystallized from toluene before use.
Reaction progress was monitored using aluminium-supported silica gel TLC plates (Merck, Kieselgel 60, F254); visualization was carried out by irradiation with UV light (254 nm), and spraying with 20% H_2SO_4 in EtOH (w/v) or $(NH_4)_6Mo_7O_{24}\cdot 4H_2O$ (25 g/L) and $(NH_4)_4Ce(SO_4)_4\cdot 2H_2O$ (10 g/L) in 10% H_2SO_4 or $KMnO_4$ (7.5 g/L) and K_2CO_3 (50 g/L) in H_2O, followed by charring. Additional analysis with TLC-MS was used when needed.
Column chromatography was carried out using silica gel (Fluka, 40-63 µm mesh). The column was prepared using the apolar component mentioned in the corresponding experimental. If the apolar component was pentane the product was brought up in toluene. If the apolar component was DCM the product was brought up in DCM, possibly with a few drops of methanol if needed. Colum chromatography was performed using a gradient ranging from 0% polar component up to the ratio mentioned in the corresponding experimental in 2 to 5 steps depending on the ease of separation.
NMR spectra were recorded at ambient temperature on a Bruker AV-400LIQ or AV-850 spectrometer. Samples were prepared in $CDCl_3$ unless stated otherwise. Chemical shifts (δ) in $CDCl_3$ are reported in ppm relative to Me_4Si (δ: 0.00 ppm) for ^{1}H-NMR and $CDCl_3$ (δ: 77.16 ppm) for ^{13}C-NMR. Chemical shifts in CD_3OD are reported in ppm relative to H_2O (δ: 4.87 ppm) for ^{1}H-NMR and CD_3OD (δ: 49.00 ppm) for ^{13}C-NMR. ^{13}C-APT spectra are ^{1}H decoupled and structural assignment was achieved using HH-COSY, HSQC and HMBC 2D experiments. Coupling constants (J) are given in Hz. Coupling constants of anomeric carbon atoms ($J_{H1,C1}$) were determined using HMBC-GATED experiments. Optical rotations were measured on an Anton Paar Modular Circular Polarimeter MCP 100/150. High resolution mass spectra were recorded on a Q Exactive HF Orbitrap equipped with an electron spray ion source positive mode. Infrared spectra were recorded on a Perkin Elmer Spectrum 2 FT-IR.

General procedure A: Pre-activation glycosylation

Donor (1.5 eq), Ph_2SO (2.0 eq) and TTBP (3.8 eq) were dried by co-evaporation with toluene (3x) followed by 3 vacuum/nitrogen purges. The mixture was then dissolved in DCM (0.05 M) and flame-dried 3Å molecular sieves were added. The solution was then cooled to -60 °C after which Tf_2O (2.0 eq) was added to the solution. After stirring for 30 minutes, acceptor (1.0 eq), which was also dried by co-evaporation with toluene (3x) followed by 3 vacuum/nitrogen purges, was dissolved in DCM (0.4 M) and slowly added to the solution. After TLC analysis indicated the consumption of the acceptor (1-4 hours) the reaction was quenched by addition of NEt_3. The reaction mixture was then diluted with DCM, filtered over celite, washed with brine, dried with $MgSO_4$ and concentrated *in vacuo*. Thereafter the product was purified by means of column chromatography.

General procedure B: Sonogashira cross coupling

Iodoaryl glycoside (1.0 eq) was dissolved in freshly distilled NEt_3 (0.05 M) together with alkyne (1.2 – 3 eq). A mixture of $Pd(PPh_3)_2Cl_2$, PPh_3 and CuI (ratio 1:1:2) was dissolved in freshly distilled NEt_3 and was stirred for 15 minutes at 40 °C. Of this cocktail, enough was added to the sugar/alkyne mixture to amount to 0.05 eq $Pd(PPh_3)_2Cl_2$, 0.05 eq PPh_3 and 0.1 eq CuI. The reaction was allowed to stir at 40 °C until the complete consumption of the starting material as indicated by TLC (2-16 h). The solvent was then removed under a stream of N_2. The crude was then transferred to a silica column in toluene and the column was flushed with toluene. Thereafter the product was purified by means of column chromatography.

General procedure C: Esterification with mycocerosic acid

Starting material (1.0 eq) was dissolved in dry DCM (0.05 M) together with mycocerosic acid (3.0 eq) and DMAP (9 eq). The resulting mixture was cooled to 0 °C after which DIC (6 eq) was added. The reaction was allowed to stir for 16 hours while warming to rT, after which it was warmed to 40 °C and stirred for a further 5 hours. The reaction mixture was then diluted with Et_2O and the organic layer was washed 1 M HCl, sat. aq. $NaHCO_3$ and brine, dried with $MgSO_4$ and concentrated *in vacuo*. Thereafter the product was purified by means of column chromatography. Note: In order to detect the most prevalent byproducts on TLC, staining with $KMnO_4$ is required.

General procedure D: Hydrogenation

Starting material (1.0 eq) was dissolved in a mixture of THF and EtOH (1:1, 0.007 M) and the solution was purged with N_2. Pd/C (10%, 1.0 eq) was then added to the solution and the resulting mixture was purged with H_2. The reaction was left to stir under H_2 atmosphere until TLC complete conversion of the starting material and reaction intermediates to a single low running spot (DCM-MeOH 19:1). The reaction mixture was then purged with N_2 and filtered over celite and the celite was rinsed with acetone. Thereafter the product was purified by means of column chromatography.

Building block Synthesis

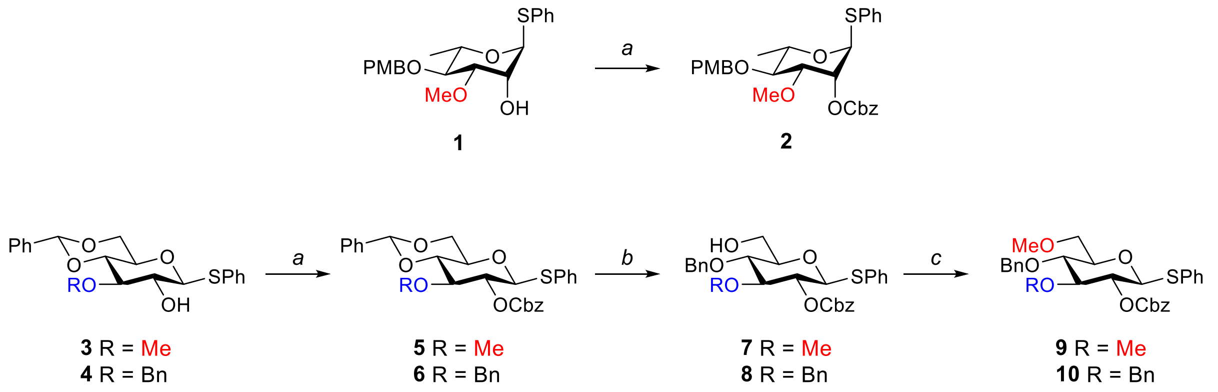

Scheme 1. Building block synthesis. *Reagents and conditions:* (a) CbzCl, DMAP, DCM, 0 °C → RT, 94% (**2**), 89% (**5**), 81% (**6**), (b) $BH_3 \cdot THF$, TMSOTf, DCM, 99% (**7**), 91% (**8**), (c) BF_4OMe_3, TTBP, DCM, 78% (**9**), 72% (**10**).

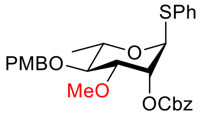

Phenyl 2-*O*-benzyloxycarbonyl-3-*O*-methyl-4-*O*-(4-methoxybenzyl)-1-thio-α-L-rhamnopyranoside (2)

Compound **1**[20] (144 mg, 0.37 mmol, 1.0 eq) was dissolved in DCM (2 mL, 0.2 M) and DMAP (124 mg, 0.94 mmol, 2.5 eq) was added to the solution. The mixture was cooled to 0 °C and CbzCl (0.11 mL, 0.75 mmol, 2.0 eq) was slowly added. The reaction was allowed to stir for 4 hours after while slowly warming to rt. The reaction was quenched by addition of 1 M HCl, and the organic layer was washed with sat. aq. $NaHCO_3$ and brine, dried with $MgSO_4$, filtered and concentrated *in vacuo*. Purification by means of column chromatography (*n*-pentane Et_2O 4:1) gave the title compound (184 mg, 0.35 mmol, 94%) as a clear oil. $[\alpha]_D^{25}$ = -105 (c = 1.0, $CHCl_3$). ^{1}H-NMR (400 MHz) δ: 7.47-7.43 (m, 2H, CH_{arom}); 7.42-7.23 (m, 12H, CH_{arom}); 6.90-6.86 (m, 2H, CH_{arom}); 5.51 (d, 1H, J = 1.6 Hz, H-1); 5.38 (dd, 1H, J = 1.6, 2.8 Hz, H-2); 5.18, 5.17 (ABq, 2H, J_{AB} = 12.4 Hz, PhCH_2); 4.82, 4.55 (ABq, 2H, J_{AB} = 10.4, PhCH_2); 4.19-4.15 (m, 1H, H-5); 3.79 (s, 3H, $CH_{3,PMB}$); 3.62 (dd, 1H, J = 3.2, 9.2 Hz, H-3); 3.49-3.43 (m, 4H, H-4, OCH_3); 1.31 (d, 3H, J = 6.0 Hz, H-6). ^{13}C-APT NMR (101 MHz) δ: 159.4 ($C_{q,arom}$); 154.8 (CO_{Cbz}); 135.0, 134.0 ($C_{q,arom}$); 131.8 (CH_{arom}); 130.7 ($C_{q,arom}$); 129.8, 129.2, 128.7, 128.7, 128.6, 127.8, 113.9 (CH_{arom}); 85.9 (C-1); 80.6 (C-3); 79.8 (C-4); 75.3 (PhCH_2); 74.4 (C-2); 70.1 (PhCH_2); 69.2 (C-5); 58.0 (OCH_3); 55.4 ($CH_{3,PMB}$); 17.8 (C-6). IR (thin film, cm^{-1}): 1027, 1086, 1172, 1248, 1302, 1382, 1457, 1514, 1747. HRMS calculated for $C_{29}H_{36}NO_7S$ 542.2212 $[M+NH_4]^+$; found 542.2208.

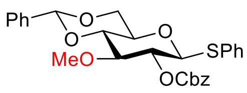

Phenyl 2-*O*-benzyloxycarbonyl-3-*O*-methyl-4,6-*O*-benzylidene-1-thio-ß-D-glucopyranoside (5)

Compound **3**[20] (0.57 g, 1.41 mmol, 1.0 eq) was dissolved in DCM (14 mL, 0.1 M) and DMAP (0.38 g, 3.10 mmol, 2.2 eq) was added to the solution. The mixture was cooled to 0 °C and CbzCl (0.4 mL, 2.82 mmol, 2.0 eq) was slowly added. The reaction was allowed to stir for 6 hours after while slowly warming to rt. The reaction was quenched by addition of 1 M HCl, and the organic layer was washed with sat. aq. $NaHCO_3$ and brine, dried with $MgSO_4$ and concentrated *in vacuo*. Purification by means of column chromatography (*n*-pentane Et_2O 4:1) gave the title compound (0.637 g, 1.25 mmol, 89%) as a white solid. $[\alpha]_D^{25}$ = 2.5 (c = 1.0, $CHCl_3$). ^{1}H-NMR (400 MHz) δ: 7.52-7.18 (m, 15H, CH_{arom}); 5.49 (s, 1H, PhC*H*); 5.26, 5.17 (ABq, 2H, J_{AB} = 12.0 Hz, PhC$H_{2,Cbz}$); 4.78-4.69 (m, 2H, H-1, H-2); 4.32 (dd, 1H, *J* = 5.0, 10.6 Hz, H-6); 3.74-3.68 (m, 1H, H-6); 3.62-3.49 (m, 5H, H-3, H-4, OCH_3); 3.46-3.39 (m, 1H, H-5). ^{13}C-APT NMR (101 MHz) δ: 154.3 (CO_{Cbz}); 137.0, 135.1 ($C_{q,arom}$); 132.9 (CH_{arom}); 132.0 ($C_{q,arom}$); 129.0, 128.9, 128.6, 128.5, 128.2, 128.0, 126.0 (CH_{arom}); 101.1 (Ph*C*H); 86.6 (C-1); 81.8 (C-4); 80.8 (C-3); 75.7 (C-2); 70.3 (C-5); 70.0 (Ph$CH_{2,Cbz}$); 68.4 (C-6); 60.7 (OCH_3). IR (thin film, cm^{-1}): 1026, 1070, 1093, 1248, 1382, 1457, 1753. HRMS calculated for $C_{28}H_{28}O_7SNa$ 531.1453 $[M+Na]^+$; found 531.1444.

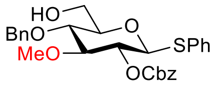

Phenyl 2-*O*-benzyloxycarbonyl-3-*O*-methyl-4-*O*-benzyl-1-thio-ß-D-glucopyranoside (7)

Compound **5** (0.63 g, 1.24 mmol, 1.0 eq.) was co-evaporated with toluene (3x) under N_2 atmosphere before it was dissolved in dry DCM (12.4 mL, 0.1 M). BH_3.THF (1 M in THF, 6.2 mL, 6.2 mmol, 5.0 eq.) was added dropwise to the solution after which TMSOTf (22 μL, 0.12 mmol, 0.1 eq.) was added to the mixture. The reaction mixture was stirred for 5 h and slowly quenched with NEt_3 (1.2 mL) followed by MeOH, which was added until the formation of H_2 ceased. The mixture was concentrated and co-evaporated with MeOH (2x). Purification by means of column chromatography (*n*-pentane-Et_2O 7:3) gave the title compound (0.628 g, 1.23 mmol, 99%) as a white solid. $[\alpha]_D^{25}$ = 16.9 (c = 1.0, $CHCl_3$). ^{1}H-NMR (400 MHz) δ: 7.45-7.25 (m, 15H, CH_{arom}); 5.27 (s, 2H, PhCH_2); 4.82 (d, 1H, *J* = 10.8 Hz, PhC*H*H); 4.72-4.61 (m, 3H, H-1, H-2, PhCH*H*); 3.86 (dd, 1H, *J* = 2.4, 12.0 Hz, H-6); 3.67 (dd, 1H, *J* = 4.6, 12.0 Hz, H-6); 3.52-3.50 (m, 4H, H-3, OCH_3); 3.47-3.42 (m, 1H, H-4); 3.39-3.35 (m, 1H, H-5); 1.87 (bs, 1H, 6-O*H*). ^{13}C-APT NMR (101 MHz) δ: 154.4 (CO_{Cbz}); 137.8, 135.3 ($C_{q,arom}$); 132.7 (CH_{arom}); 132.5 ($C_{q,arom}$); 129.2, 129.1, 128.7, 128.7, 128.6, 128.5, 128.3, 128.2, 128.2, 128.1 (CH_{arom});

86.2 (C-1); 86.0 (C-4); 79.5 (C-5); 77.0 (C-3); 76.4 (C-2); 75.2, 70.3 (Ph*C*H_2); 32.0 (C-6); 61.1 (O*C*H_3). IR (thin film, cm^{-1}): 1029, 1040, 1055, 1078, 1089, 1119, 1142, 1259, 1757, 2930. HRMS calculated for $C_{35}H_{36}O_7SNa$ 533.1610 $[M+Na]^+$; found 533.1605.

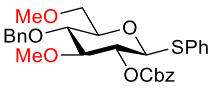

Phenyl 2-*O*-benzyloxycarbonyl-3,6-di-*O*-methyl-4-*O*-benzyl-1-thio-ß-D-glucopyranoside (9)

Compound **7** (179 mg, 0.35 mmol, 1.0 eq.) and TTBP (362 mg, 1.46 mmol, 4.0 eq) were co-evaporated with toluene (3x) under N_2 atmosphere and dissolved in dry DCM (7.2 mL, 0.05 M) and flame-dried rod shaped 3Å molecular sieves were added. Trimethyloxonium tetrafluoroborate (160 mg, 1.08 mmol, 3.0 eq) was then added to the mixture and the reaction was left to stir for 1.5 hours. The reaction was quenched with NEt_3 (0.5 mL), filtered over celite, washed with brine, dried with $MgSO_4$ and concentrated *in vacuo*. Purification by means of column chromatography (*n*-pentane-Et_2O 4:1) gave the title compound (143 mg, 0.27 mmol, 78%) as a white solid. $[\alpha]_D^{25}$ = 13.9 (c = 1.0, $CHCl_3$). ^{1}H-NMR (400 MHz) δ: 7.43-7.22 (m, 15H, CH_{arom}); 5.27 (s, 2H, PhC$H_{2,Cbz}$); 4.81 (d, 1H, *J* = 10.8 Hz, PhC*H*H); 4.73 (t, 1H, *J* = 9.4 Hz, H-2); 4.62-4.59 (m, 2H, H-1, PhCH*H*); 3.66-3.55 (m, 3H, H-3, H-6); 3.49 (s, 3H, OCH_3); 3.48-3.39 (m, 2H, H-4, H-5); 3.36 (s, 3H, OCH_3). 13C-APT NMR (101 MHz) δ: 154.4 (*C*O_{Cbz}); 138.1, 135.3, 133.1 ($C_{q,arom}$); 128.9, 128.7, 128.6, 128.6, 128.4, 128.2, 128.2, 128.1, 128.0 (*C*H_{arom}); 86.4 (C-1); 86.3 (C-4); 79.2 (C-5); 77.0 (C-3); 76.7 (C-2); 75.1 (Ph*C*H_2); 71.1 (C-6); 70.1 (Ph*C*$H_{2,Cbz}$); 60.9, 59.5 (O*C*H_3). IR (thin film, cm^{-1}): 1002, 1026, 1076, 1088, 1143, 1148, 1251, 1381, 1455, 1756, 2929. HRMS calculated for $C_{29}H_{32}O_7SNa$ 547.1766 $[M+Na]^+$; found 547.1761.

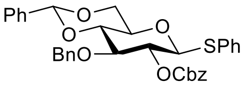

Phenyl 2-*O*-benzyloxycarbonyl-3-*O*-benzyl-4,6-*O*-benzylidene-1-thio-ß-D-glucopyranoside (6)

Compound **4**[20] (2.03 g, 4.51 mmol, 1.0 eq) was dissolved in DCM (173 mL, 0.03 M) and DMAP (1.65 g, 13.5 mmol, 3.0 eq) was added to the solution. The mixture was cooled to 0 °C and CbzCl (1.9 mL, 13.5 mmol, 3.0 eq) was slowly added. The reaction was allowed to stir for 5 hours after while slowly warming to rt. The reaction was quenched by addition of 1 M HCl, and the organic layer was washed with sat. aq. $NaHCO_3$ and brine, dried with $MgSO_4$ and concentrated *in vacuo*. Purification by means of column chromatography (*n*-pentane Et_2O 4:1) gave the title compound (2.14 g, 3.66 mmol, 81%) as a white solid. $[\alpha]_D^{25}$ = 7.2 (c = 1.0, $CHCl_3$). ^{1}H-NMR (400 MHz) δ: 7.46-7.22 (m, 20H, CH_{arom}); 5.56 (s, 1H, PhC*H*); 5.23 (s, 2H, PhC$H_{2,Cbz}$);

4.85-4.82 (m, 2H, H-2, PhC*H*H); 4.73-4.64 (m, 2H, H-1, PhCH*H*); 4.37 (dd, 1H, *J* = 4.8, 10.4 Hz, H-6); 3.82-3.70 (m, 3H, H-3, H-4, H-6); 3.51-3.45 (m, 1H, H-5). ^{13}<u>C-APT NMR</u> (101 MHz) δ: 154.3 (CO_{Cbz}); 138.0, 137.1, 135.2 ($C_{q,arom}$); 133.2 (CH_{arom}); 131.9 ($C_{q,arom}$); 129.2, 129.1, 128.7, 128.5, 128.4, 127.9, 127.8, 126.1 (CH_{arom}); 101.3 (Ph*C*H); 86.7 (C-1); 81.2 (C-4); 79.9 (C-3); 75.8 (C-2); 74.7 ($PhCH_2$); 70.6 (C-5); 70.2 ($PhCH_{2,Cbz}$); 68.6 (C-6). <u>IR</u> (thin film, cm^{-1}): 1027, 1069, 1096, 1249, 1312, 1382, 1441, 1455, 1754. <u>HRMS</u> calculated for $C_{34}H_{32}O_7SNa$ 607.1766 $[M+Na]^+$; found 607.1761.

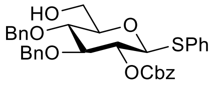

Phenyl 2-*O*-benzyloxycarbonyl-3,4-di-*O*-benzyl-1-thio-ß-D-glucopyranoside (8)

Compound **6** (257 mg, 0.44 mmol, 1.0 eq.) was co-evaporated with toluene (3x) under N_2 atmosphere before it was dissolved in dry DCM (4.4 mL, 0.1 M). A 1M solution of BH_3□THF (4.4 mL, 4.4 mmol, 10.0 eq) in THF was added dropwise to the solution after which TMSOTf (0.08 mL, 0.44 mmol, 1.0 eq) was added to the mixture. The reaction mixture was stirred for 4 h and slowly quenched with NEt_3 (1 mL) followed by MeOH, which was added until the formation of H_2 ceased. The mixture was concentrated and co-evaporated with MeOH (2x). Purification by means of column chromatography (*n*-pentane-Et_2O 7:3) gave the title compound (0.234 g, 0.40 mmol, 91%) as a white solid. $[\alpha]_D^{25}$ = 21.5 (c = 1.0, $CHCl_3$). ^{1}<u>H-NMR</u> (400 MHz) δ: 7.45-7.41 (m, 2H, CH_{arom}); 7.37-7.17 (m, 18H, CH_{arom}); 5.21, 5.17 (ABq, 2H, J_{AB} = 12.2 Hz, $PhCH_{2,Cbz}$); 4.83-4.74 (m, 3H, H-2, PhC*H*H, PhC*H*H); 4.68-4.60 (m, 3H, H-1, PhCH*H*, PhCH*H*); 3.86 (dd, 1H, *J* = 2.4, 12.0 Hz, H-6); 3.74-3.57 (m, 2H, H-4, H-6); 3.40 (t, 1H, *J* = 2.4 Hz, H-3); 3.39-3.36 (m, 1H, H-5); 2.11 (bs, 1H, 6-O*H*). ^{13}<u>C-APT NMR</u> (101 MHz) δ: 154.3 (CO_{Cbz}); 137.8, 137.7, 135.1 ($C_{q,arom}$); 129.1, 129.0, 128.6, 128.5, 128.4, 128.4, 128.1, 128.0, 127.8, 127.8 (CH_{arom}); 85.9 (C-1); 84.0 (C-4); 79.5 (C-5); 77.1 (C-3); 76.3 (C-2); 75.5, 75.1 ($PhCH_2$); 70.1 ($PhCH_{2,Cbz}$); 61.8 (C-6). <u>IR</u> (thin film, cm^{-1}): 1002, 1012, 1027, 1039, 1072, 1090, 1146, 1252, 1454, 1731, 1756, 2928. <u>HRMS</u> calculated for $C_{34}H_{34}O_7SNa$ 609.1923 $[M+Na]^+$; found 609.1918.

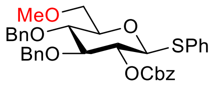

Phenyl 2-*O*-benzyloxycarbonyl-3,4-di-*O*-benzyl-6-*O*-methyl-1-thio-ß-D-glucopyranoside (10)

Compound **8** (135 mg, 0.23 mmol, 1.0 eq.) and TTBP (229 mg, 0.92 mmol, 4.0 eq) were co-evaporated with toluene (3x) under N_2 atmosphere and dissolved in dry DCM (4.6 mL, 0.05 M) and flame-dried rod shaped 3Å molecular sieves were added. Trimethyloxonium

tetrafluoroborate (102 mg, 0.69 mmol, 3.0 eq) was then added to the mixture and the reaction was left to stir for 2 hours. The reaction was quenched with NEt_3 (0.5 mL), filtered over celite, washed with brine, dried with $MgSO_4$ and concentrated *in vacuo*. Purification by means of column chromatography (*n*-pentane-Et_2O 4:1) gave the title compound (99 mg, 0.165 mmol, 72%) as a white solid. $[\alpha]_D^{25}$ = 26.1 (c = 1.0, $CHCl_3$). ^{1}H-NMR (400 MHz) δ: 7.48-7.44 (m, 2H, CH_{arom}); 7.39-7.18 (m, 18H, CH_{arom}); 5.20, 5.15 (ABq, 2H, J_{AB} = 12.2 Hz, $PhCH_{2,Cbz}$); 4.85-4.74 (m, 3H, H-2, PhC*H*H, PhC*H*H); 4.68-4.60 (m, 3H, H-1, PhCH*H*, PhCH*H*); 3.73-3.59 (m, 4H, H-3, H-4, H-6); 3.47-3.43 (m, 1H, H-5); 3.37 (OCH_3). ^{13}C-APT NMR (101 MHz) δ: 154.3 (CO_{Cbz}); 138.0, 138.0, 135.2, 133.1 ($C_{q,arom}$); 132.6, 129.0, 128.7, 128.7, 128.6, 128.5, 128.1, 128.1, 128.0, 127.9, 127.8 (CH_{arom}); 86.5 (C-1); 84.4 (C-4); 79.3 (C-5); 77.5 (C-3); 76.4 (C-2); 75.6, 75.3 ($PhCH_2$); 71.2 (C-6); 70.2 ($PhCH_{2,Cbz}$); 59.6 (OCH_3). IR (thin film, cm^{-1}): 1000, 1026, 1076, 1142, 1246, 1362, 1381, 1440, 1454, 1753. HRMS calculated for $C_{35}H_{36}O_7SNa$ 623.2079 $[M+Na]^+$; found 623.2074.

Oligosaccharide synthesis

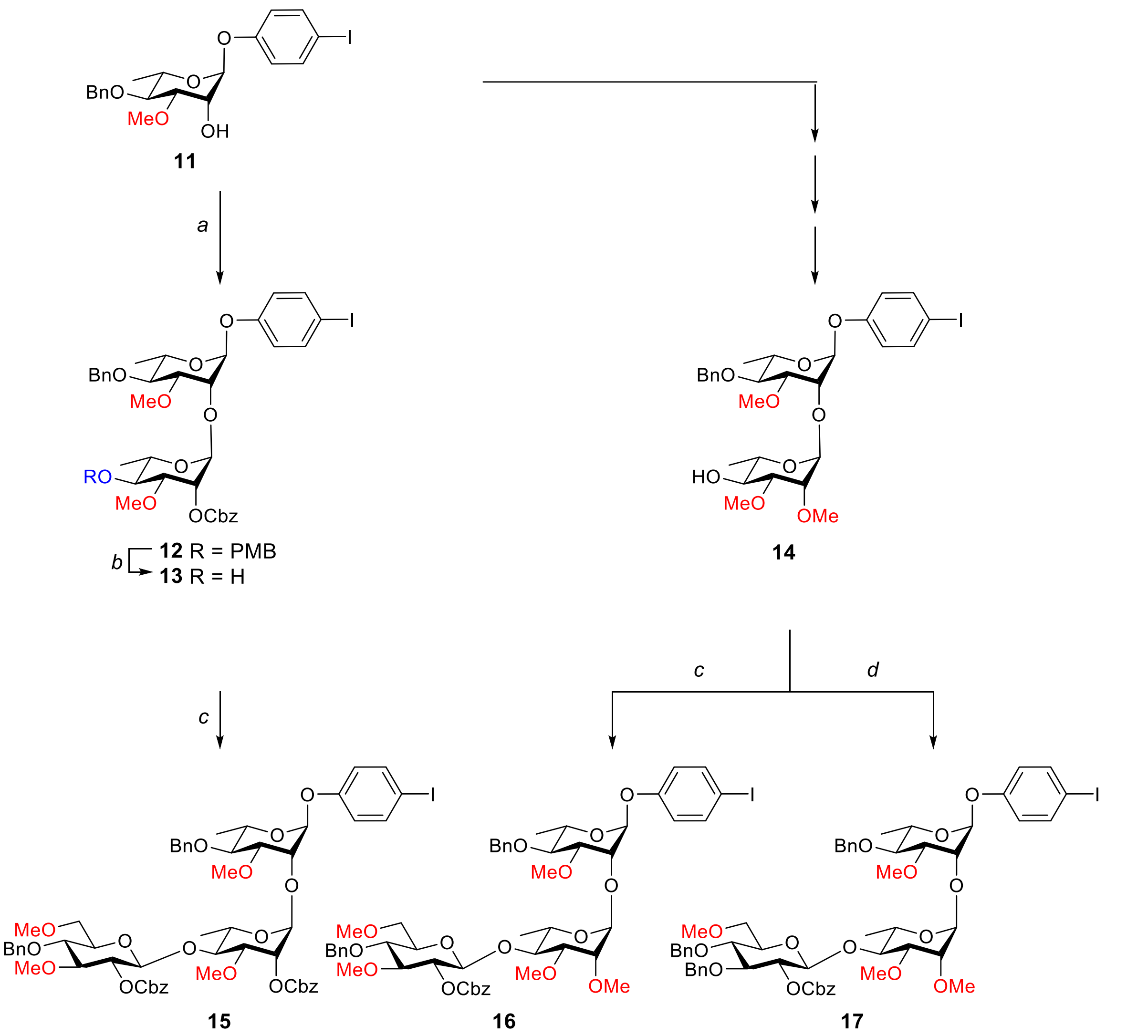

Scheme 2. Synthesis of protected *M. leprae* trisaccharides. *Reagents and conditions:* (a) Donor **2**, Ph_2SO, Tf_2O, TTBP, DCM -60 °C, 47%, (b) HCl/HFIP, HFIP/DCM, 98%, (c) Donor **9**, Ph_2SO, Tf_2O, TTBP, DCM -60 °C, 66% (**15**), 68% (**16**), (d) Donor **10**, Ph_2SO, Tf_2O, TTBP, DCM -60 °C, 96%.

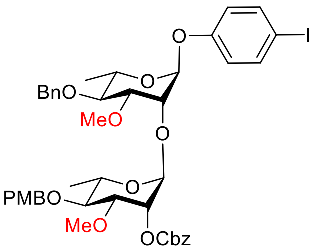

4-iodophenyl 2-*O*-(2-*O*-benzyloxycarbonyl-3-*O*-methyl-4-*O*-(4-methoxybenzyl)-α-L-rhamnopyranosyl)-3-*O*-methyl-4-*O*-benzyl-α-L-rhamnopyranoside (12)

Prepared according to glycosylation procedure A using donor **2** (393 mg, 0.75 mmol, 1.5 eq) and acceptor **1**[20] (235 mg, 0.5 mmol, 1.0 eq). The title compound

was obtained after column chromatography (*n*-pentane-Et_2O 4:1) as a slightly yellow oil (206 mg, 0.23 mmol, 47%). $[\alpha]_D^{25}$ = -69.5 (c =0.8, $CHCl_3$). ^{1}H-NMR (400 MHz) δ: 7.56-7.53 (m, 2H, CH_{arom}); 7.42-7.24 (m, 10H, CH_{arom}); 6.89-6.87 (m, 2H, CH_{arom}); 6.79-6.76 (m, 2H, CH_{arom}); 5.41 (s, 1H, H-1); 5.28-5.27 (m, 1H, H-2'); 5.27-5.18 (m, 2H, PhCH_2); 5.14 (s, 1H, H-1'); 4.90 (d, 1H, *J* = 10.8 Hz, PhC*H*H); 4.81 (d, 1H, *J* = 10.4 Hz, PhC*H*H); 4.63 (d, 1H, *J* = 10.8 Hz, PhCH*H*); 4.53 (d, 1H, *J* = 10.4 Hz, PhCH*H*); 4.17 (t, 1H, *J* = 2.2 Hz, H-2); 3.79-3.65 (m, 7H, H-3, H-3', H-5, H-5', $CH_{3,PMB}$); 3.53 (s, 3H, OCH_3); 3.52 (s, 3H, OCH_3); 3.44-3.37 (m, 2H, H-4, H-4'); 1.28 (d, 3H, *J* = 6.0 Hz, H-6); 1.22 (d, 3H, *J* = 6.0 Hz, H-6'); ^{13}C-APT NMR (101 MHz) δ: 159.4, 156.0 ($C_{q,arom}$); 154.8 (CO_{Cbz}); 138.5 ($C_{q,arom}$); 138.5 (CH_{arom}); 135.2, 130.7 ($C_{q,arom}$); 128.7, 128.5, 128.5, 128.1, 127.8, 118.6, 114.0 (CH_{arom}); 99.0 (C-1'); 96.8 (C-1); 84.8 (CI_{arom}); 81.4 (C-3); 79.9 (C-3'); 79.9 (C-4); 79.6 (C-4'); 75.3, 75.3 (PhCH_2); 73.5 (C-2); 72.6 (C-2'); 70.0 (PhCH_2); 68.8 (C-5'); 68.5 (C-5); 58.1, 58.0 (OCH_3); 55.4 ($CH_{3,PMB}$); 18.1 (C-6'); 18.0 (C-6). IR (thin film, cm^{-1}): 1036, 1072, 1093, 1120, 1173, 1233, 1264, 1387, 1457, 1484, 1513, 1750. HRMS calculated for $C_{43}H_{49}IO_{12}Na$ 907.2166 $[M+Na]^+$; found 907.2143.

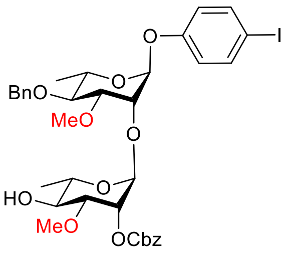

4-iodophenyl 2-*O*-(2-*O*-benzyloxycarbonyl-3-*O*-methyl-α-L-rhamnopyranosyl)-3-*O*-methyl-4-*O*-benzyl-α-L-rhamnopyranoside (13)

Compound **12** (192 mg, 0.22 mmol, 1.0 eq) was dissolved in a mixture of DCM and HFIP (1:1, 2.2 mL, 0.1 M) after which a solution of HCl in HFIP (0.11 mL, 0.2 M, 0.1 eq) was added. After complete conversion of the starting material, indicated by a dark purple colour, the reaction was quenched by addition of sat. aq. $NaHCO_3$. The mixture was diluted with DCM, washed with brine, dried with $MgSO_4$ and concentrated *in vacuo*. Purification by means of column chromatography (*n*-pentane-Et_2O 1:1) gave the title compound (163 mg, 0.21 mmol, 98%) as a pale oil. $[\alpha]_D^{25}$ = -49.9 (c = 1.0, $CHCl_3$). ^{1}H-NMR (400 MHz) δ: 7.58-7.55 (m, 2H, CH_{arom}); 7.46-7.29 (m, 10H, CH_{arom}); 6.82-6.79 (m, 2H, CH_{arom}); 5.45 (d, 1H, *J* = 2.0 Hz, H-1); 5.28 (dd, 1H, *J* = 1.8, 2.6 Hz, H-2'); 5.20-5.18 (m, 3H, H-1', PhCH_2); 4.91 (d, 1H, *J* = 11.2 Hz, PhC*H*H); 4.65 (d, 1H, *J* = 10.8 Hz, PhCH*H*); 4.21 (dd, 1H, *J* = 2.4, 2.8 Hz, H-2); 3.81-3.68 (m, 3H, H-3, H-5, H-5'); 3.58-3.52 (m, 5H, H-3', H-4', OCH_3); 3.47-3.42 (m, 4H, H-4, OCH_3); 2.45 (d, 1H, *J* = 2.0 Hz, 4'-O*H*); 1.30 (d, 3H, *J* = 6.4 Hz, H-6); 1.25 (d, 3H, *J* = 6.4 Hz, H-6'); ^{13}C-APT NMR (101 MHz) δ: 156.0 ($C_{q,arom}$); 154.8 (CO_{Cbz}); 138.5 (CH_{arom}); 138.5, 135.1 ($C_{q,arom}$); 128.7, 128.5, 128.5, 128.2, 127.9, 118.6 (CH_{arom}); 99.2 (C-1'); 96.9 (C-1); 84.9 (CI_{arom}); 81.4 (C-3); 79.9 (C-4); 79.4 (C-4'); 75.3 (PhCH_2); 73.5 (C-2); 71.6 (C-3'); 71.3 (C-2'); 70.1 (Ph*C*H); 68.9 (C-5); 68.8 (C-5'); 58.2, 57.7 (OCH_3); 18.2 (C-6'); 17.8 (C-

6). IR (thin film, cm^{-1}): 1033, 1049, 1073, 1092, 1138, 1232, 1264, 1385, 1445, 1454, 1484, 1747, 2932, 3450. HRMS calculated for $C_{35}H_{41}IO_{11}Na$ 787.1591 $[M+Na]^+$; found 787.1567.

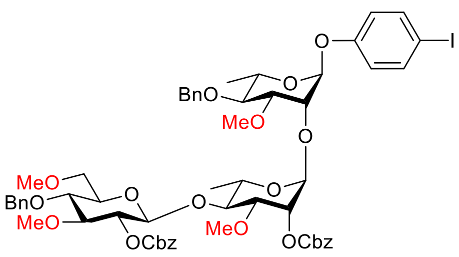

4-iodophenyl 2-*O*-(2-*O*-benzyloxycarbonyl-3-*O*-methyl-4-*O*-(2-*O*-benzyloxycarbonyl-3,6-di-*O*-methyl-4-*O*-benzyl-ß-D-glucopyranosyl)-α-L-rhamnopyranosyl)-3-*O*-methyl-4-*O*-benzyl-α-L-rhamnopyranoside (15)

Prepared according to glycosylation procedure A using donor **9** (67 mg, 0.12 mmol, 1.5 eq) and acceptor **13** (63 mg, 0.08 mmol, 1.0 eq). The title compound was obtained after column chromatography (*n*-pentane-Et_2O 3:2) as a slightly yellow oil (66 mg, 0.06 mmol, 68%). $[\alpha]_D^{25}$ = -54.7 (c = 1.0, $CHCl_3$). ^{1}H-NMR (400 MHz) δ: 7.57 (dd, 2H, *J* = 2.0, 7.2 Hz, CH_{arom}); 7.42-7.24 (m, 20H, CH_{arom}); 6.81 (d, 2H, *J* = 9.2 Hz, CH_{arom}); 5.44 (d, 1H, *J* = 1.2 Hz, H-1); 5.25-5.14 (m, 6H, H-1', H-2', $PhCH_2$); 4.93 (d, 1H, *J* = 10.8 Hz, PhC*H*H); 4.80 (d, 1H, *J* = 10.8 Hz, PhC*H*H); 4.69-4.62 (m, 4H, H-1", H-2", PhCH*H*, PhCH*H*); 4.22 (dd, 1H, *J* = 2.0, 2.8 Hz, H-2); 3.78-3.45 (m, 15H, H-3, H-3', H-4, H-4', H-4", H-5, H-5', H-6", OCH_3); 3.40-3.34 (m, 5H, H-3", H-5", OCH_3); 3.26 (s, 3H, OCH_3); 1.30-1.25 (m, 6H, H-6, H-6'). ^{13}C-APT NMR (101 MHz) δ: 155.9 ($C_{q,arom}$); 154.8, 154.8 (CO_{Cbz}); 138.5 (CH_{arom}); 138.5, 138.3, 135.5, 135.1 ($C_{q,arom}$); 128.8, 128.7, 128.7, 128.6, 128.5, 128.5, 128.2, 128.1, 128.0, 127.9, 118.7 (CH_{arom}); 101.2 (C-1"); 98.7 (C-1'); 96.9 (C-1); 84.9 (CI_{arom}); 84.9 (C-3"); 81.6 (C-3); 79.9 (C-3'); 79.3 (C-4); 78.0 (C-2"); 77.9 (C-4"); 77.5 (C-4'); 75.3, 75.0 ($PhCH_2$); 74.8 (C-5"); 72.8 (C-2); 72.1 (C-2'); 71.0 (C-6"); 70.1, 69.9 ($PhCH_2$); 68.8, 67.9 (C-5 and C-5'); 60.9, 59.8, 58.2, 57.8 (OCH_3); 18.1, 17.9 (C-6 and C-6'). IR (thin film, cm^{-1}): 1003, 1035, 1057, 1073, 1140, 1262, 1385, 1455, 1484, 1751. HRMS calculated for $C_{58}H_{67}IO_{18}Na$ 1201.3270 $[M+Na]^+$; found 1201.3257.

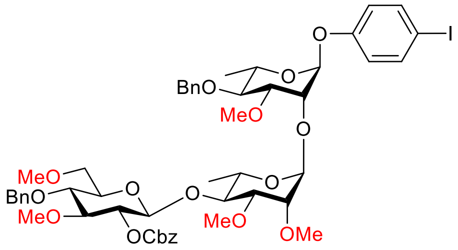

4-iodophenyl 2-*O*-(2,3-di-*O*-methyl-4-*O*-(2-*O*-benzyloxycarbonyl-3,6-di-*O*-methyl-4-*O*-benzyl-ß-D-glucopyranosyl)-α-L-rhamnopyranosyl)-3-*O*-methyl-4-*O*-benzyl-α-L-rhamnopyranoside (16)

Prepared according to general procedure A using donor **9** (2.57 g, 4.9 mmol, 1.4 eq) and acceptor **14**[20] (2.23 g, 3.47 mmol, 1.0 eq). The title compound was obtained after column chromatography (*n*-pentane-Et_2O 2:3) as a pale oil (2.93 g, 2.77 mmol, 80%). $[\alpha]_D^{25}$ = -63.7 (c = 1.0, $CHCl_3$). ^{1}H-NMR (400 MHz) δ: 7.58 (dd, 2H, *J* = 2.0, 6.8 Hz, CH_{arom}); 7.40-7.26 (m, 15H, CH_{arom}); 6.82 (dd, 2H, *J* = 2.2, 7.0 Hz,

CH_{arom}); 5.43 (d, 1H, J = 1.6 Hz, H-1); 5.29-5.22 (m, 2H, PhCH_2); 5.18 (d, 1H, J = 1.2 Hz, H-1'); 4.89 (d, 1H, J = 10.8 Hz, PhC*H*H); 4.79 (d, 1H, J = 10.8 Hz, PhC*H*H); 4.74 (d, 1H, J = 8.0 Hz, H-1''); 4.67-4.62 (m, 3H, H-2'', PhCH*H*, PhCH*H*); 4.24 (t, 1H, J = 2.4 Hz, H-2); 3.78 (dd, 1H, J = 3.2, 9.2 Hz, H-3); 3.75-3.33 (m, 27H, H-2', H-3, H-3', H-3'', H-4, H-4', H-4'', H-5, H-5', H-5'', H-6'', OCH_3); 1.29-1.26 (m, 6H, H-6, H-6'). ^{13}C-APT NMR (101 MHz) δ: 155.9 ($C_{q,arom}$); 154.8 (*C*O_{Cbz}); 138.5 (*C*H_{arom}); 138.4, 138.2, 135.6 ($C_{q,arom}$); 128.8, 128.6, 128.6, 128.5, 128.4, 128.2, 128.1, 128.0, 127.9, 118.6 (*C*H_{arom}); 100.9 (C-1''); 98.5 (C-1'); 97.0 (C-1); 84.9 (*C*I_{arom}); 84.9 (C-3''); 80.8 (C-4'); 80.0 (C-4); 78.1 (C-3); 77.6 (C-4''); 77.6 (C-5''); 76.9 (C-2); 75.2, 75.0 (Ph*C*H_2); 74.8 (C-3'); 72.9 (C-2); 71.0 (Ph*C*H_2); 69.8 (C-6''); 68.8, 67.9 (C-5 and C-5'); 61.0, 59.8, 59.1, 58.3, 57.6 (O*C*H_3); 18.2, 18.0 (C-6 and C-6'). IR (thin film, cm^{-1}): 1029, 1055, 1072, 1092, 1120, 1139, 1235, 1259, 1454, 1484, 1757, 2932. HRMS calculated for $C_{51}H_{63}IO_{16}Na$ 1081.3058 $[M+Na]^+$; found 1081.3053.

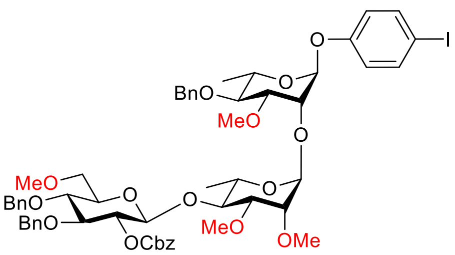

4-iodophenyl 2-*O*-(2,3-di-*O*-methyl-4-*O*-(2-*O*-benzyloxycarbonyl-3,4-di-*O*-benzyl-6-*O*-methyl-ß-D-glucopyranosyl)-α-L-rhamnopyranosyl)-3-*O*-methyl-4-*O*-benzyl-α-L-rhamnopyranoside (17)

Prepared according to general procedure A using donor **10** (105 mg, 0.17 mmol, 1.5 eq) and acceptor **14**[20] (91 mg, 0.12 mmol) the title compound was obtained after column chromatography (*n*-pentane-Et_2O 1:4) as a slightly yellow oil (127 mg, 0.11 mmol, 96%). $[\alpha]_D^{25}$ = -66.3 (c = 1.0, $CHCl_3$). ^{1}H-NMR (400 MHz) δ: 7.58 (dd, 2H, J = 2.0, 6.8 Hz, CH_{arom}); 7.36-7.21 (m, 20H, CH_{arom}); 6.83 (dd, 2H, J = 2.2, 7.0 Hz, CH_{arom}); 5.44 (d, 1H, J = 1.6 Hz, H-1); 5.24-5.15 (m, 3H, H-1', PhCH_2); 4.89 (d, 1H, J = 7.2 Hz, PhC*H*H); 4.80-4.76 (m, 4H, H-1'', H-2'', PhC*H*H, PhC*H*H); 4.70-4.62 (m, 3H, PhCH*H*, PhCH*H*, PhCH*H*); 4.24 (dd, 1H, J = 2.0, 2.8 Hz, H-2); 3.80-3.63 (m, 9H, H-2', H-3, H-3'', H-4'', H-5, H-5', H-5'', H-6''); 3.56-3.42 (m, 10H, H-4, H-4', H-6'', OCH_3, OCH_3); 3.37-3.27 (m, 7H, H-3'', OCH_3); 1.30-1.26 (m, 6H, H-6, H-6'). ^{13}C-APT NMR (101 MHz) δ: 155.9 ($C_{q,arom}$); 154.7, (*C*O_{Cbz}); 138.5 (*C*H_{arom}); 138.4, 138.3, 138.2, 135.5 ($C_{q,arom}$); 128.7, 128.6, 128.5, 128.5, 128.4, 128.1, 128.1, 127.9, 127.9, 127.7, 127.7, 118.6 (*C*H_{arom}); 101.0 (C-1''); 98.5 (C-1'); 97.0 (C-1); 84.9 (*C*I_{arom}); 83.3 (C-4''); 81.9 (C-3); 80.8, 80.0 (C-4 and C-4'); 78.2 (C-2''); 77.8, 77.7, 76.9 (C-2', C-3'', and C-5''); 75.4, 75.2, 75.1 (Ph*C*H_2); 74.9 (C-3'); 72.9 (C-2); 71.1 (C-6''); 69.8 (Ph*C*H_2); 68.8, 67.9 (C-5 and C-5'); 59.8, 59.1, 58.3, 57.6 (O*C*H_3); 18.2, 18.0 (C-6 and C-6'). IR (thin film, cm^{-1}): 1005, 1016, 1030, 1053, 1072, 1093, 1120, 1140, 1236, 1259, 1484, 1757. HRMS calculated for $C_{57}H_{67}IO_{16}Na$ 1157.3371 $[M+Na]^+$; found 1157.3366.

Final PGL assembly

Table 1. Final stages of PGL assembly

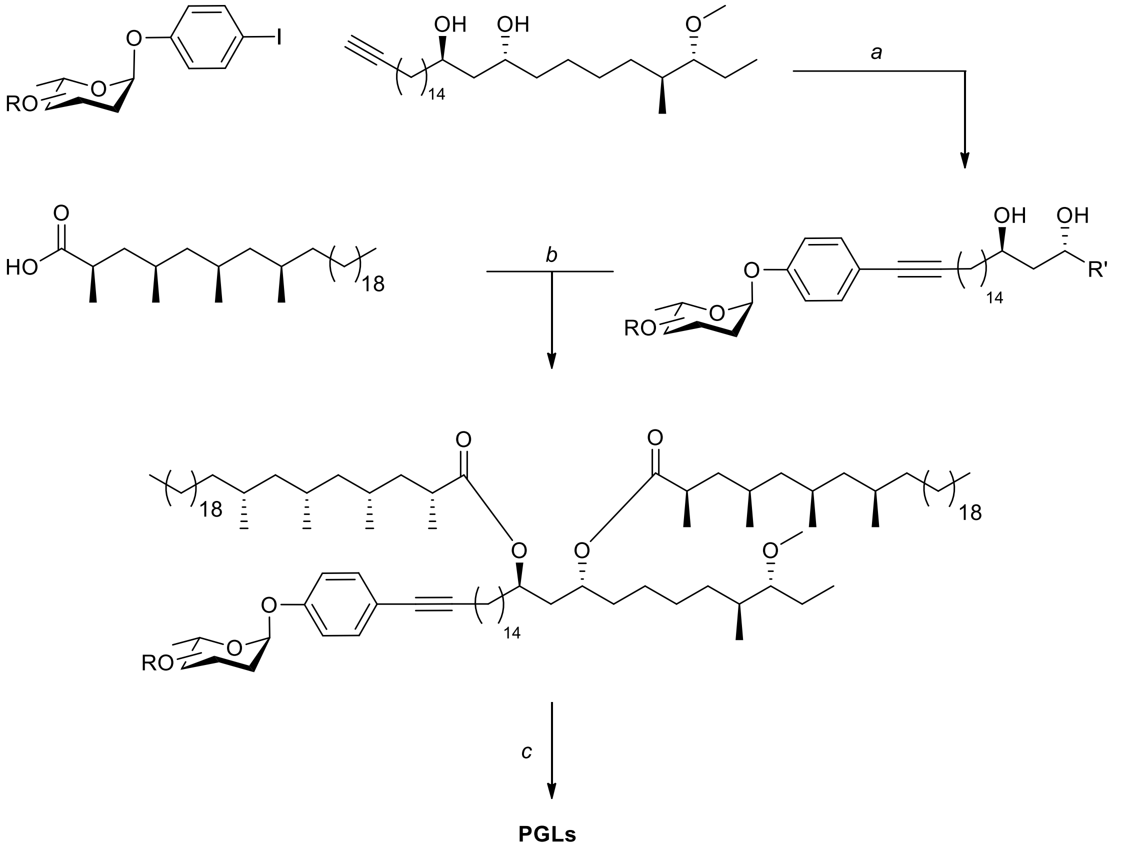

Reagents and conditions: (a) $Pd(PPh_3)_2Cl_2$, PPh_3, CuI, Et_3N, 40 °C, (b) DIC, DMAP, DCM, 0 °C → RT → 40 °C, (c) Pd/C, H_2, THF/EtOH.

Starting material	Sonogashira	Esterification	Hydrogenation	Overall yield
**16**	83%	79%	79%	52%
**15**	93%	80%	76%	57%
**17**	83%	76%	40%	25%

4-((3*R*,4*S*,9*R*,11*R*)-3-methoxy-4-methylheptacos-26-yne-9,11-diol)phenyl 2-*O*-(2,3-di-*O*-methyl-4-*O*-(2-*O*-benzyloxycarbonyl-3,6-di-*O*-methyl-4-*O*-benzyl-ß-D-glucopyranosyl)-α-L-rhamnopyranosyl)-3-*O*-methyl-4-*O*-benzyl-α-L-rhamnopyranoside (18)

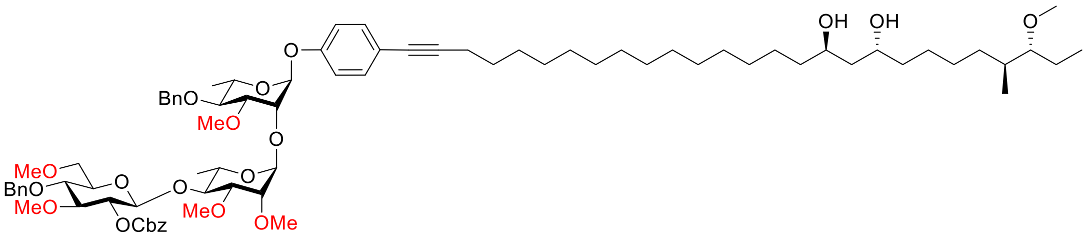

The title compound was synthesized according to general procedure B using **16** (23 mg, 22 μmol, 1.0 eq) and phtiocerol[21] (12 mg, 26 μmol, 1.2 eq). Column chromatography (DCM-acetone 4:1) yielded the product (25 mg, 18 μmol, 83%) as a yellow oil. $[\alpha]_D^{25}$ = -50.9 (c = 1.0, $CHCl_3$). ^{1}H-NMR (400 MHz) δ: 7.40-7.26 (m, 17, CH_{arom}); 6.96-6.94 (m, 2H, CH_{arom}); 5.47 (d, 1H, *J* = 1.6 Hz, H-1); 5.39-5.32 (m, 2H, PhCH_2); 5.18 (d, 1H, *J* = 1.2 Hz, H-1'); 4.89 (d, 1H, *J* = 10.8 Hz, PhC*H*H); 4.79 (d, 1H, *J* = 10.8 Hz, PhC*H*H); 4.74 (d, 1H, *J* = 8.0 Hz, H-1"); 4.67-4.62 (m, 3H, H-2", PhCH*H*, PhCH*H*); 4.24 (s, 1H, H-2); 3.96-3.90 (m, 2H, CH_{Phth}); 3.67-3.33 (m, 30H, H-2', H-3, H-3', H-3", H-4, H-4', H-4", H-5, H-5', H-5", H-6", OCH_3); 2.90-2.84 (m, 1H, CH_{Phth}); 2.38 (t, 2H, *J* = 7.2 Hz, C$H_{2,Phth}$); 2.05 (bs, 2H, OH_{Phth}); 1.72-1.64 (m, 1H, CH_{Phth}); 1.62-1.05 (m, 60H, C$H_{2,Phth}$, H-6, H-6'); 0.91 (t, 3H, *J* = 7.4 Hz, C$H_{3,Phth}$); 0.83 (d, 3H, *J* = 6.8 Hz, C$H_{3,Phth}$). ^{13}C-APT NMR (101 MHz) δ: 155.3 ($C_{q,arom}$); 154.8 (*C*O_{Cbz}); 138.5, 138.3, 135.6 ($C_{q,arom}$); 133.0, 128.8, 128.6, 128.6, 128.5, 128.4, 128.3, 128.1, 128.0, 127.9 (*C*H_{arom}); 118.0 ($C_{q,arom}$); 116.2 (*C*H_{arom}); 100.9 (C-1"); 98.5 (C-1'); 96.9 (C-1); 89.5 ($C_{q,alkyne}$); 86.8 (*C*H_{Phth}); 85.0 (C-3"); 82.0 (C-3); 80.8 (C-4'); 80.1 (C-4); 80.1 ($C_{q,alkyne}$); 78.1 (C-2"); 77.7, 77.6 (C-4" and C-5"); 77.0 (C-2'); 75.3, 75.0 (Ph*C*H_2); 74.8 (C-3'); 73.0 (C-2); 71.1 (C-6"); 69.9 (Ph*C*H_2); 69.6, 69.6 (*C*H_{Phth}); 68.7 (C-5); 67.9 (C-5'); 61.0, 59.8, 59.1, 58.3, 57.6, 57.5 (O*C*H_3); 42.4, 37.7 (*C*$H_{2,Phth}$); 34.9 (*C*H_{Phth}); 32.8, 29.8, 29.7, 29.3, 29.1, 29.0, 27.7, 26.3, 25.9, 22.5, 19.5 (*C*$H_{2,Phth}$); 18.2, 18.0 (C-6 and C-6'); 14.9, 10.2 (*C*$H_{3,Phth}$). IR (thin film, cm^{-1}): 1000, 1030, 1055, 1075, 1093, 1122, 1139, 1175, 1205, 1235, 1259, 1383, 1455, 1507, 1749, 2854, 2928, 3470. HRMS calculated for $C_{80}H_{118}O_{19}Na$ 1405.8165 $[M+Na]^+$; found 1405.8160.

4-((3*R*,4*S*,9*R*,11*R*)-3-methoxy-4-methylheptacos-26-yne-9,11-diol)phenyl 2-*O*-(2-*O*-benzyloxycarbonyl-3-*O*-methyl-4-*O*-(2-*O*-benzyloxycarbonyl-3,6-di-*O*-methyl-4-*O*-benzyl-ß-D-glucopyranosyl)-α-L-rhamnopyranosyl)-3-*O*-methyl-4-*O*-benzyl-α-L-rhamnopyranoside (19)

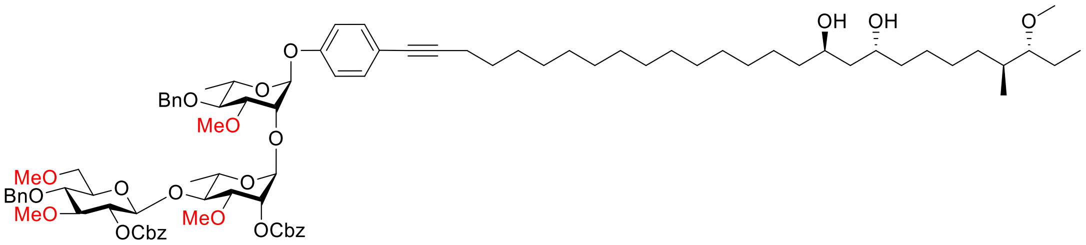

The title compound was synthesized according to general procedure B using **15** (59 mg, 50 μmol, 1.0 eq) and phthiocerol[21] (27 mg, 60 μmol, 1.2 eq). Column chromatography (DCM-acetone 4:1) yielded the product (70 mg, 47 μmol, 93%) as a yellow oil. $[\alpha]_D^{25}$ = -174.8 (c = 1.0, $CHCl_3$). ^{1}H-NMR (400 MHz) δ: 7.41-7.26 (m, 22H, CH_{arom}); 6.94 (dd, 2H, *J* = 2.0, 8.8 Hz, CH_{arom}); 5.47 (d, 1H, *J* = 1.6 Hz, H-1); 5.25-5.17 (m, 6H, H-1', H-2', PhCH_2); 4.92 (d, 1H, *J* = 10.8 Hz, PhC*H*H); 4.80 (d, 1H, *J* = 10.8 Hz, PhC*H*H); 4.67-4.61 (m, 4H, H-1", H-2", PhCH*H*, PhCH*H*); 4.22 (s, 1H, H-2); 3.96-3.90 (m, 2H, CH_{Phth}); 3.64-3.47 (m, 15H, H-3, H-3', H-4, H-4', H-4", H-5, H-5', H-6", OCH_3); 3.37-3.33 (m, 8H, H-3", H-5", OCH_3); 3.26 (s, 3H, OCH_3); 2.90-2.84 (m, 1H, CH_{Phth}); 2.38 (t, 2H, *J* = 7.2 Hz, $CH_{2,Phth}$); 2.05 (bs, 2H, OH_{Phth}); 1.62-1.05 (m, 60H, H-6, H-6', $CH_{2,Phth}$); 0.91 (t, 3H, *J* = 7.4 Hz, $CH_{3,Phth}$); 0.83 (d, 3H, *J* = 6.8 Hz, $CH_{3,Phth}$). ^{13}C-APT NMR (101 MHz) δ: 155.3 ($C_{q,arom}$); 154.8, 154.8 (CO_{Cbz}); 138.5, 138.3, 135.5, 135.1 ($C_{q,arom}$); 133.0, 128.8, 128.7, 128.7, 128.6, 128.5, 128.5, 128.2, 128.2, 128.0, 127.8 (CH_{arom}); 118.0 ($C_{q,arom}$); 116.2 (CH_{arom}); 101.3 (C-1"); 98.8 (C-1'); 96.7 (C-1); 89.5 ($C_{q,alkyne}$); 86.8 (CH_{Phth}); 84.9 (C-3"); 81.7 (C-3); 80.1 ($C_{q,alkyne}$); 80.0 (C-3'); 79.3 (C-4); 78.0 (C-4"); 77.9 (C-2"); 77.6 (C-4'); 75.3, 75.0 (PhCH_2); 74.8 (C-5"); 72.9 (C-2); 72.1 (C-2'); 71.1 (C-6"); 70.1, 69.9 (PhCH_2); 69.6, 69.6 (CH_{Phth}); 68.8 (C-5'); 67.9 (C-5); 61.0, 59.8, 58.2, 57.8, 57.5 (OCH_3); 42.4, 37.7 ($CH_{2,Phth}$); 34.9 (CH_{Phth}); 32.8, 29.8, 29.8, 29.7, 29.3, 29.1, 29.0, 27.7, 26.3, 25.9, 22.5, 19.5 ($CH_{2,Phth}$); 18.1, 17.9 (C-6 and C-6'); 14.9, 10.2 ($CH_{3,Phth}$). IR (thin film, cm^{-1}): 1029, 1037, 1057, 1073, 1093, 1125, 1142, 1262, 1507, 1753, 2855, 2926. HRMS calculated for $C_{87}H_{122}O_{21}Na$ 1525.8376[M+Na]$^+$; found 1525.8374.

4-((3*R*,4*S*,9*R*,11*R*)-3-methoxy-4-methylheptacos-26-yne-9,11-diol)phenyl 2-*O*-(2,3-di-*O*-methyl-4-*O*-(2-*O*-benzyloxycarbonyl-3,4-di-*O*-benzyl-6-*O*-methyl-ß-D-glucopyranosyl)-α-L-rhamnopyranosyl)-3-*O*-methyl-4-*O*-benzyl-α-L-rhamnopyranoside (20)

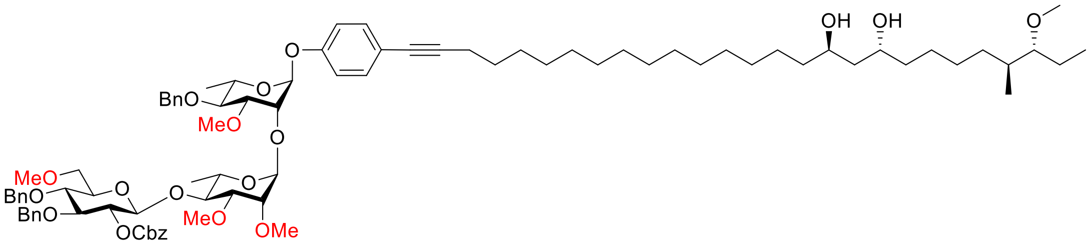

The title compound was synthesized according to general procedure B using **17** (62 mg, 55 μmol, 1.0 eq) and phthiocerol[21] (30 mg, 66 μmol, 1.2 eq). Column chromatography (DCM-EtOAc 1:1) yielded the product (66 mg, 45 μmol, 83%) as a yellow oil. $[\alpha]_D^{25}$ = -51.3 (c = 1.0, $CHCl_3$). ^{1}H-NMR (400 MHz) δ: 7.36-7.21 (m, 22H, CH_{arom}); 6.96-6.94 (m, 2H, CH_{arom}); 5.48 (d, 1H, *J* = 1.6 Hz, H-1); 5.24-5.15 (m, 3H, H-1', PhCH_2); 4.89 (d, 1H, *J* = 10.8 Hz, PhC*H*H); 4.80-4.76 (m, 4H, H-1'', H-2'', PhC*H*H, PhC*H*H); 4.69-4.62 (m, 3H, PhCH*H*, PhCH*H*, PhCH*H*); 4.24 (d, 1H, *J* = 2.0 Hz, H-2); 3.98-3.89 (m, 2H, CH_{Phth}); 3.79 (dd, 1H, *J* = 3.0, 9.4 Hz, H-3); 3.76-3.61 (m, 7H, H-2', H-3'', H-4'', H-5, H-5', H-5'', H-6''); 3.58-3.42 (m, 7H, H-6'', OCH_3); 3.38-3.33 (m, 7H, H-3', OCH_3); 2.90-2.82 (m, 1H, CH_{Phth}); 2.38 (t, 1H, *J* = 7.0 Hz, $CH_{2,Phth}$); 1.95 (bs, 2H, OH_{Phth}); 1.72-1.64 (m, 1H, CH_{Phth}); 1.62-1.25 (m, 47H, $CH_{2,Phth}$, H-6, H-6'); 1.15-1.05 (m, 1H, $CH_{2,Phth}$); 0.91 (t, 3H, *J* = 7.4 Hz, $CH_{3,Phth}$); 0.83 (d, 3H, *J* = 6.8 Hz, $CH_{3,Phth}$). ^{13}C-APT NMR (101 MHz) δ: 155.3 ($C_{q,arom}$); 154.7 (CO_{Cbz}); 138.5, 138.4, 138.2, 135.5 ($C_{q,arom}$); 133.0, 128.8, 128.6, 128.6, 128.5, 128.4, 128.2, 128.1, 128.0, 127.9, 127.8, 127.7 (CH_{arom}); 118.0 ($C_{q,arom}$); 116.2 (CH_{arom}); 101.1 (C-1''); 98.6 (C-1'); 96.9 (C-1); 89.5 ($C_{q,alkyne}$); 86.8 (CH_{Phth}); 83.3 (C-4''); 82.0 (C-3); 80.8, 80.1 (C-4 and C-4'); 80.1 ($C_{q,alkyne}$); 78.2 (C-2''); 77.9, 77.8 77.0 (C-2', C-3'', C-5''); 75.4, 75.3, 75.2 (PhCH_2); 75.0 (C-3') 73.0 (C-2); 71.1 (C-6''); 69.9 (PhCH_2); 69.7, 69.6 (CH_{Phth}); 68.7 (C-5); 68.0 (C-5'); 59.9, 59.1, 58.3, 57.6, 57.5 (OCH_3); 42.4, 37.7 ($CH_{2,Phth}$); 34.9 (CH_{Phth}); 32.8, 29.8, 29.8, 29.7, 29.3, 29.1, 29.0, 27.7, 26.3, 25.9, 22.5, 19.5 ($CH_{2,Phth}$); 18.2, 18.1 (C-6 and C-6'); 14.9, 10.2 ($CH_{3,Phth}$). IR (thin film, cm^{-1}): 1002, 1009, 1020, 1027, 1053, 1073, 1093, 1110, 1120, 1136, 1143, 1236, 1263, 1457, 1507, 1734, 2855, 2869, 2927, 2965, 2969. HRMS calculated for $C_{86}H_{122}O_{19}Na$ 1481.8478 $[M+Na]^+$; found 1481.8473.

4-((3*R*,4*S*,9*R*,11*R*)-3-methoxy-4-methylheptacos-26-yne-9,11-diyl bismycocerosate)phenyl 2-*O*-(2,3-di-*O*-methyl-4-*O*-(2-*O*-benzyloxycarbonyl-3,6-di-*O*-methyl-4-*O*-benzyl-ß-D-glucopyranosyl)-α-L-rhamnopyranosyl)-3-*O*-methyl-4-*O*-benzyl-α-L-rhamnopyranoside (21)

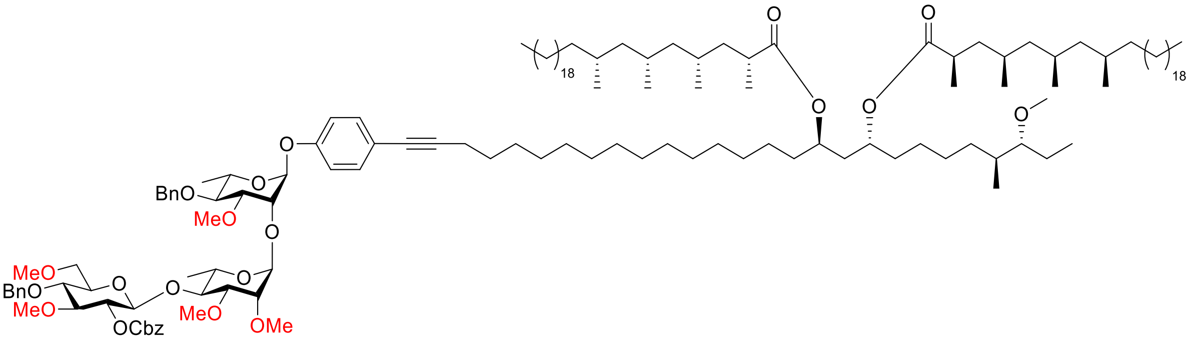

The title compound was synthesized according to general procedure C using **18** (34 mg, 25 μmol, 1.0 eq), mycocerosic acid[22] (35 mg, 74 μmol, 3.0 eq), DIC (23 μL, 147 μmol, 6.0 eq) and DMAP (27 mg, 221 μmol, 9.0 eq). Column chromatography (*n*-pentane-Et_2O 1:1) yielded the product (45 mg, 19 μmol, 79%) as a waxy solid. $[\alpha]_D^{25}$ = -36.3 (c = 1.0, $CHCl_3$). ^{1}H-NMR (400 MHz) δ: 7.45-7.26 (m, 17H, CH_{arom}); 6.96-6.93 (m, 2H, CH_{arom}); 5.47 (d, 1H, *J* = 1.6 Hz, H-1); 5.26. 5.25 (ABq, 2H, J_{AB} = 12.2 Hz, PhCH_2); 5.19 (d, 1H, *J* = 1.2 Hz, H-1'); 4.91-4.78 (m, 4H, PhC*H*H, PhC*H*H, CH_{Phth}); 4.74 (d, 1H, *J* = 8.0 Hz, H-1"); 4.67-4.62 (m, 3H, PhCH*H*, PhCH*H*, H-2"); 4.24 (dd, 1H, *J* = 2.2, 2.6 Hz, H-2); 3.79 (dd, 1H, *J* = 3.2, 9.2 Hz, H-3); 3.76-3.49 (m, 17H, H-2, H-4', H-4", H-5, H-5', H-6", OCH_3); 3.47-3.39 (m, 2H, H-3', H-4); 3.37-3.31 (m, 11H, H-3", H-5", OCH_3); 2.88-2.84 (m, 1H, CH_{Phth}); 2.55-2.50 (m, 2H, CH_{Myc}); 2.37 (t, 2H, *J* = 7.0 Hz, C$H_{2,Phth}$); 1.77-0.81 (m, 204H, CH_{Phth}, C$H_{2,Phth}$, C$H_{3,Phth}$, C$H_{Myc,}$ C$H_{2,Myc}$, C$H_{3,Myc}$, H-6, H-6'). ^{13}C-APT NMR (101 MHz) δ: 176.1 (CO_{Myc}); 155.3 ($C_{q,arom}$); 154.8 (CO_{Cbz}); 138.5, 138.3, 133.0 ($C_{q,arom}$); 128.8, 128.6, 128.6, 128.5, 128.5, 128.3, 128.1, 128.0, 127.9 (CH_{arom}); 118.0 ($C_{q,arom}$); 116.2 (CH_{arom}); 100.9 (C-1"); 98.5 (C-1'); 96.9 (C-1); 89.5 ($C_{q,alkyne}$); 86.8 (CH_{Phth}); 85.0 (C-3"); 82.0 (C-3); 80.8 (C-3'); 80.1 ($C_{q,alkyne}$); 80.1 (C-4); 78.1 (C-2"); 77.7 (C-4'); 77.6 (C-4"); 77.0 (C-2'); 75.3, 75.1 (PhCH_2); 74.8 (C-5"); 73.0 (C-2); 71.1 (C-6"); 70.4 (CH_{Phth}); 69.9 (PhCH_2); 68.7 (C-5); 67.9 (C-5'); 61.0, 59.8, 59.1, 58.3, 57.6, 57.5 (OCH_3); 45.6, 45.4 ($CH_{2,Myc}$); 41.1, 38.6 ($CH_{2,Phth}$); 37.9, 37.9 (CH_{Myc}); 36.8 ($CH_{2,Myc}$); 34.9 (CH_{Phth}); 34.8, 32.8 ($CH_{2,Phth}$); 32.1 ($CH_{2,Myc}$); 30.2 ($CH_{2,Phth}$); 30.1 (CH_{Myc}); 29.9, 29.9, 29.8, 29.8, 29.8, 29.7, 29.5, 29.4, 29.2, 29.1 (CH_2); 28.2 (CH_{Myc}); 27.6 ($CH_{2,Phth}$); 27.3 (CH_{Myc}); 27.1 ($CH_{2,Myc}$); 25.7, 25.3 ($CH_{2,Phth}$); 22.8 ($CH_{2,Myc}$); 22.5 ($CH_{2,Phth}$); 20.9, 20.6, 20.5 ($CH_{3,Myc}$); 19.6 ($CH_{2,Phth}$); 18.6 ($CH_{3,Myc}$); 18.2 (C-6); 18.0 (C-6'); 14.8 ($CH_{3,Phth}$); 14.3 ($CH_{3,Myc}$); 10.2 ($CH_{3,Phth}$). IR (thin film, cm^{-1}): 1093, 1173, 1259, 1378, 1457, 1464, 1507, 1734, 1760, 2853, 2923. HRMS calculated for $C_{144}H_{243}O_{21}$ 2309.79755 $[M+H]^+$; found 2309.80566.

4-((3*R*,4*S*,9*R*,11*R*)-3-methoxy-4-methylheptacos-26-yne-9,11-diyl bismycocerosate)phenyl 2-*O*-(2-*O*-benzyloxycarbonyl-3-*O*-methyl-4-*O*-(2-*O*-benzyloxycarbonyl-3,6-di-*O*-methyl-4-*O*-benzyl-ß-D-glucopyranosyl)-α-L-rhamnopyranosyl)-3-*O*-methyl-4-*O*-benzyl-α-L-rhamnopyranoside (22)

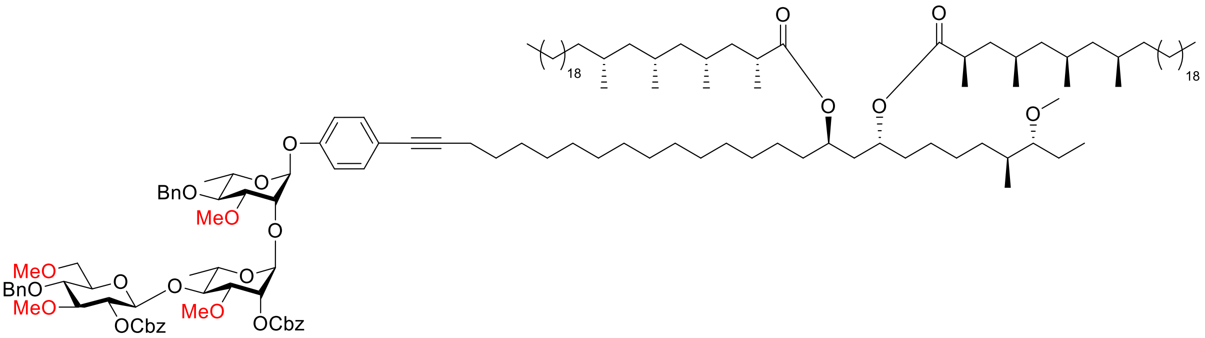

The title compound was synthesized according to general procedure C using **19** (37 mg, 25 μmol, 1.0 eq), mycocerosic acid[22] (35 mg, 74 μmol, 3.0 eq), DIC (23 μL, 148 μmol, 6.0 eq) and DMAP (27 mg, 221 μmol, 9.0 eq). Column chromatography (*n*-pentane-Et_2O 4:1) yielded the product (48 mg, 20 μmol, 80%) as a waxy solid. $[\alpha]_D^{25}$ = -35.1 (c = 1.0, $CHCl_3$). ^{1}H-NMR (400 MHz) δ: 7.42-7.26 (m, 22H, CH_{arom}); 6.95-6.92 (m, 2H, CH_{arom}); 5.47 (d, 1H, *J* = 2.0 Hz, H-1); 5.28-5.14 (m, 6H, H-1', H-2', PhCH_2, PhCH_2); 4.93-4.78 (m, 4H, PhC*H*H, PhC*H*H, CH_{Phth}); 4.69-4.61 (m, 4H, H-1", H-2", PhCH*H*, PhCH*H*); 4.22 (dd, 1H, *J* = 2.0, 3.2 Hz, H-2); 3.78 (dd, 1H, *J* = 3.2, 9.2 Hz, H-3); 3.75-3.61 (m, 3H, H-5, H-5', H-6"); 3.59-3.45 (m, 11H, H-3', H-4, H-4', H-4", H-6", OCH_3); 3.39-3.33 (m, 8H, H-3", H-5", OCH_3); 3.26 (s, 3H, OCH_3); 2.87-2.84 (m, 1H, CH_{Phth}); 2.55-2.50 (m, 2H, CH_{Myc}); 2.37 (t, 2H, *J* = 7.2 Hz, C$H_{2,Phth}$); 1.77-0.81 (m, 205H, H-6, H-6', CH_{Phth}, C$H_{2,Phth}$, C$H_{3,Phth}$, CH_{Myc}, C$H_{2,Myc}$, C$H_{3,Myc}$). ^{13}C-APT NMR (101 MHz) δ: 176.2, 176.1 (CO_{Myc}); 155.3 ($C_{q,arom}$); 154.8 (CO_{Cbz}); 138.6, 138.3, 135.5, 135.1 ($C_{q,arom}$); 133.0, 128.8, 128.8, 128.7, 128.7, 128.6, 128.6, 128.5, 128.5, 128.2, 128.2, 128.0, 127.9 (CH_{arom}); 118.0 ($C_{q,arom}$); 116.2 (CH_{arom}); 101.3 (C-1"); 98.8 (C-1'); 96.7 (C-1); 89.5 ($C_{q,alkyne}$); 86.8 (CH_{Phth}); 84.9 (C-3"); 81.7 (C-3); 80.1 ($C_{q,alkyne}$); 80.0 (C-4); 79.3 (C-3'); 78.0 (C-4'); 78.0 (C-4"); 77.6 (C-2"); 75.4, 75.0 (PhCH_2); 74.8 (C-5"); 72.9 (C-2); 72.1 (C-2'); 71.1 (C-6"); 70.4 (CH_{Phth}); 70.1, 69.9 (PhCH_2); 68.8 (C-5); 67.9 (C-5'); 61.0, 59.8, 58.2, 57.8, 57.5 (OCH_3); 45.6, 45.4 ($CH_{2,Myc}$); 41.1, 38.6 ($CH_{2,Phth}$); 37.9 (CH_{Myc}); 36.8 ($CH_{2,Myc}$); 34.9 (CH_{Phth}); 34.8, 32.8 ($CH_{2,Phth}$); 32.1 ($CH_{2,Myc}$); 30.2 ($CH_{2,Phth}$); 30.1 (CH_{Myc}); 29.9, 29.9, 29.8, 29.5, 29.4, 29.2, 29.1 (CH_2); 28.2 (CH_{Myc}); 27.6 ($CH_{2,Phth}$); 27.3 (CH_{Myc}); 27.1 ($CH_{2,Myc}$); 25.7, 25.3 ($CH_{2,Phth}$); 22.8 ($CH_{2,Myc}$); 22.5 ($CH_{2,Phth}$); 20.9, 20.6, 20.6 ($CH_{3,Myc}$); 19.6 ($CH_{2,Phth}$); 18.6 ($CH_{3,Myc}$); 18.1 (C-6); 17.9 (C-6'); 14.8 ($CH_{3,Phth}$); 14.3 ($CH_{3,Myc}$); 10.3 ($CH_{3,Phth}$). IR (thin film,

cm^{-1}): 1095, 1176, 1259, 1379, 1457, 1507, 1736, 1756, 2853, 2923. HRMS calculated for $C_{151}H_{247}O_{23}$ 2429.81868 [M+H]$^+$; found 2429.82801.

4-((3*R*,4*S*,9*R*,11*R*)-3-methoxy-4-methylheptacos-26-yne-9,11-diyl bismycocerosate)phenyl 2-*O*-(2,3-di-*O*-methyl-4-*O*-(2-*O*-benzyloxycarbonyl-3,4-di-*O*-benzyl-6-*O*-methyl-ß-D-glucopyranosyl)-α-L-rhamnopyranosyl)-3-*O*-methyl-4-*O*-benzyl-α-L-rhamnopyranoside (23)

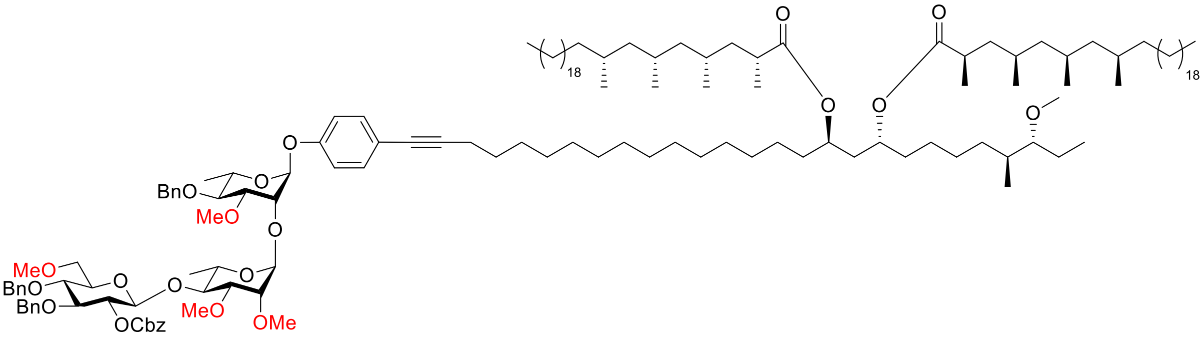

The title compound was synthesized according to general procedure C using **20** (26 mg, 18 μmol, 1.0 eq), mycocerosic acid[22] (25 mg, 53 μmol, 3.0 eq), DIC (16 μL, 105 μmol, 6.0 eq) and DMAP (19 mg, 158 μmol, 9.0 eq). Column chromatography (*n*-pentane-Et_2O 1:1) yielded the product (32 mg, 13.4 μmol, 76%) as a waxy solid. $[\alpha]_D^{25}$ = -32.9 (c = 1.0, $CHCl_3$). ^{1}H-NMR (400 MHz) δ: 7.37-7.21 (m, 22H, CH_{arom}); 6.97-6.93 (m, 2H, CH_{arom}); 5.48 (d, 1H, *J* = 1.6 Hz, H-1); 5.24-5.15 (m, 3H, H-1', PhCH_2,); 4.91-4.73 (m, 7H, H-1", H-2", CH_{Phth}, PhC*H*H, PhC*H*H, PhC*H*H); 4.70-4.62 (m, 3H, PhCH*H*, PhCH*H,* PhCH*H*); 4.25 (dd, 1H, *J* = 2.4, 2.8 Hz, H-2); 3.79 (dd, 1H, *J* = 3.0, 9.4 Hz, H-3); 3.76-3.60 (m, 7H, H-2', H-3", H-4', H-4", H-5, H-5', H-6"); 3.59-3.51 (m, 7H, H-6", OCH_3); 3.48-3.41 (m, 2H, H-3', H-4); 3.38-3.31 (m, 10H, H-5", OCH_3); 2.88-2.83 (m, 1H, CH_{Phth}); 2.55-2.50 (m, 2H, CH_{Myc}); 1.77-0.81 (m, 181H, CH_{Phth}, $CH_{2,Phth}$, $CH_{3,Phth}$, CH_{Myc}, $CH_{2,Myc}$, $CH_{3,Myc}$, H-6, H-6'). ^{13}C-APT NMR (101 MHz) δ: 176.1, 176.1 (CO_{Myc}); 155.3 ($C_{q,arom}$); 154.7 (CO_{Cbz}); 138.5, 138.4, 138.2, 135.5 ($C_{q,arom}$); 133.0, 128.8, 128.6, 128.6, 128.5, 128.5, 128.4, 128.2, 128.1, 128.0, 127.9, 127.8, 127.7 (CH_{arom}); 118.0 ($C_{q,arom}$); 116.2 (CH_{arom}); 101.1 (C-1"); 98.5 (C-1'); 96.9 (C-1); 89.5 ($C_{q,alkyne}$); 86.8 (CH_{Phth}); 83.3 (C-3"); 82.0 (C-3); 80.8 (C-3'); 80.1 ($C_{q,alkyne}$); 80.1 (C-4); 78.2 (C-2"); 77.9 (C-4'); 77.7 (C-4"); 77.0 (C-2'); 75.4, 75.3, 75.2 (PhCH_2); 75.0 (C-5"); 73.0 (C-2); 71.1 (C-6"); 70.4 (CH_{Phth}); 69.9 (PhCH_2); 68.7 (C-5); 68.0 (C-5'); 59.9, 59.1, 58.3, 57.6, 57.5 (OCH_3); 45.6, 45.4 ($CH_{2,Myc}$); 41.1, 38.6 ($CH_{2,Phth}$); 37.9, 37.9 (CH_{Myc}); 36.7 ($CH_{2,Myc}$); 34.9 (CH_{Phth}); 34.8, 32.8 ($CH_{2,Phth}$); 32.1 ($CH_{2,Myc}$); 30.2 ($CH_{2,Phth}$); 30.1 (CH_{Myc}); 29.9, 29.9, 29.8, 29.8, 29.8, 29.7, 29.5, 29.4, 29.2, 29.1 (CH_2); 28.2 (CH_{Myc}); 27.6 ($CH_{2,Phth}$); 27.3 (CH_{Myc}); 27.1 ($CH_{2,Myc}$); 25.7, 25.3 ($CH_{2,Phth}$); 22.8 ($CH_{2,Myc}$);

22.5 ($CH_{2,Phth}$); 20.9, 20.6, 20.5, 20.5 ($CH_{3,Myc}$); 19.6 ($CH_{2,Phth}$); 18.6 ($CH_{3,Myc}$); 18.2 (C-6); 18.0 (C-6'); 14.8 ($CH_{3,Phth}$); 14.3 ($CH_{3,Myc}$); 10.2 ($CH_{3,Phth}$). IR (thin film, cm^{-1}): 1073, 1095, 1140, 1259, 1379, 1457, 1464, 1507, 1734, 1756, 1763, 2853, 2923. HRMS calculated for $C_{150}H_{247}O_{21}$ 2385.82885 $[M+H]^+$; found 2385.83921.

4-((3*R*,4*S*,9*R*,11*R*)-3-methoxy-4-methylheptacos-26-yne-9,11-diyl bismycocerosate)phenyl 2-*O*-(2,3-di-*O*-methyl-4-*O*-(3,6-di-*O*-methyl-ß-D-glucopyranosyl)-α-L-rhamnopyranosyl)-3-*O*-methyl-α-L-rhamnopyranoside (24)

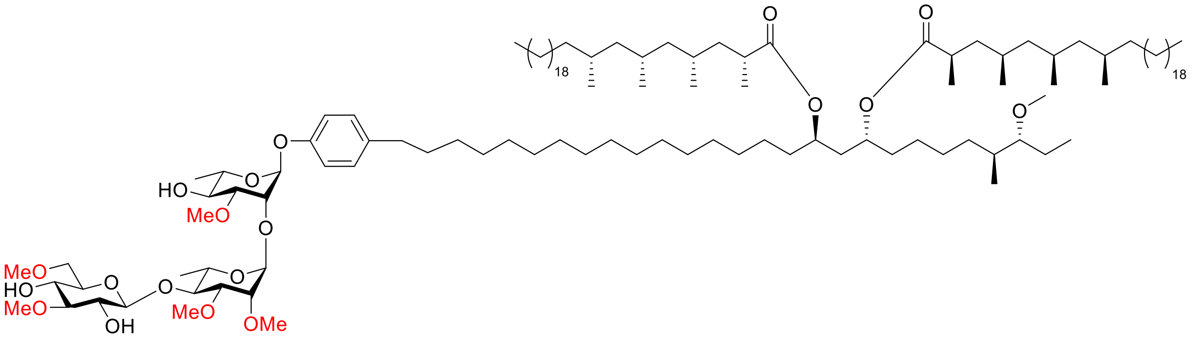

The title compound was synthesized according to general procedure D using **21** (32 mg, 14 μmol, 1.0 eq) and Pd/C (10%, 15 mg, 14 μmol, 1.0 eq). Column chromatography (DCM-acetone 3:2) yielded the product (22 mg, 11 μmol, 79%) as a waxy solid. $[\alpha]_D^{25}$ = -25.2 (c = 1.0, $CHCl_3$). ^{1}H-NMR (850 MHz) δ: 7.10 (d, 2H, J = 9.4 Hz, CH_{arom}); 6.94 (d, 2H, J = 8.5 Hz, CH_{arom}); 5.43 (d, 1H, J = 1.7 Hz, H-1); 5.10 (d, 1H, J = 1.7 Hz, H-1'); 4.84 (quint, 2H, J = 6.4 Hz, CH_{Phth}); 4.41 (d, 1H, J = 7.7 Hz, H-1"); 4.22 (dd, 1H, J = 1.7, 3.4 Hz, H-2); 3.89 (s, 1H, 2"-O*H*); 3.77-3.74 (m, 3H, H-2', H-5, H-5'); 3.69-3.66 (m, 4H, H-3', OCH_3); 3.65-3.61 (m, 4H, H-3, H-4', H-6"); 3.58 (dt, 1H, J = 1.7, 9.4 Hz, H-4); 3.55-3.52 (m, 4H, H-4", OCH_3); 3.50 (s, 3H, OCH_3); 3.48 (s, 3H, OCH_3); 3.38 (s, 3H, OCH_3); 3.17 (t, 1H, J = 9.4 Hz, H-3"); 2.87-2.84 (m, 1H, CH_{Phth}); 2.80 (bs, 1H, 4"-O*H*); 2.56-2.51(m, 4H, $CH_{2,Phth}$, CH_{Myc}); 2.30 (bs, 1H, 4-O*H*); 1.77-0.81 (m, 190H, H-6, H-6', CH_{Phth}, $CH_{2,Phth}$, $CH_{3,Phth}$, CH_{Myc}, $CH_{2,Myc}$, $CH_{3,Myc}$). ^{13}C-APT NMR (214 MHz) δ: 176.2, 176.1 (CO_{Myc}); 154.3, 137.0 ($C_{q,arom}$); 129.5, 116.1 (CH_{arom}); 105.6 (C-1"); 98.5 (C-1'); 97.4 (C-1); 86.8 (CH_{Phth}); 85.6 (C-3"); 81.8 (C-4'); 81.5 (C-3); 80.3 (C-3'); 75.6 (C-2'); 75.1 (C-2"); 74.1 (C-5"); 73.0 (C-6"); 72.2 (C-2); 71.9 (C-4); 71.4 (C-4"); 70.4, 70.4 (CH_{Phth}); 69.1 (C-5); 68.4 (C-5'); 60.7, 59.7, 59.2, 57.8, 57.5, 56.7 (OCH_3); 45.6, 45.6, 45.4 45.4 ($CH_{2,Myc}$); 41.1, 41.1, 38.5 ($CH_{2,Phth}$); 37.9, 37.9 (CH_{Myc}); 36.7, 35.3 ($CH_{2,Myc}$); 34.9 (CH_{Phth}); 34.8, 32.8 ($CH_{2,Phth}$); 32.1 ($CH_{2,Myc}$); 31.9, 30.2 ($CH_{2,Phth}$); 30.0 (CH_{Myc}); 29.9, 29.9, 29.9, 29.9, 29.8, 29.8, 29.7, 29.7, 29.6, 29.5 (CH_2); 28.1 (CH_{Myc}); 27.6 ($CH_{2,Phth}$); 27.3 (CH_{Myc}); 27.1 ($CH_{2,Myc}$); 25.7, 25.3 ($CH_{2,Phth}$); 22.8 ($CH_{2,Myc}$); 22.4 ($CH_{2,Phth}$); 20.9, 20.6, 20.5, 20.5, 18.6, 18.6 ($CH_{3,Myc}$); 17.9 (C-6); 17.7 (C-6'); 14.8 ($CH_{3,Phth}$); 14.3 ($CH_{3,Myc}$); 10.3 ($CH_{3,Phth}$). IR (thin film,

cm^{-1}): 1010, 1070, 1123, 1175, 1229, 1261, 1378, 1457, 1464, 1511, 1734, 2853, 2922, 3434. HRMS calculated for $C_{122}H_{229}O_{19}$ 1998.69476 [M+H]$^+$; found 1998.69683.

4-((3*R*,4*S*,9*R*,11*R*)-3-methoxy-4-methylheptacos-26-yne-9,11-diyl bismycocerosate)phenyl 2-*O*-(3-*O*-methyl-4-*O*-(3,6-di-*O*-methyl-4-*O*-benzyl-ß-D-glucopyranosyl)-α-L-rhamnopyranosyl)-3-*O*-methyl-α-L-rhamnopyranoside (25)

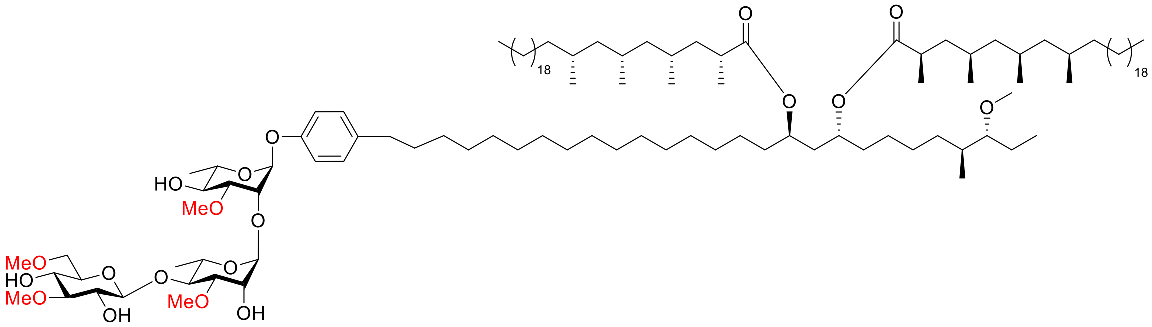

The title compound was synthesized according to general procedure D using **22** (37 mg, 15 μmol, 1.0 eq) and Pd/C (10%, 16 mg, 15 μmol, 1.0 eq). Column chromatography (DCM-MeOH 11:1) yielded the product (23 mg, 12 μmol, 76%) as a waxy solid. $[\alpha]_D^{25}$ = -24.4 (c = 1.0, $CHCl_3$). ^{1}H-NMR (850 MHz) δ: 7.10 (d, 2H, *J* = 8.5 Hz, C*H*$_{arom}$); 6.95-6.94 (m, 2H, C*H*$_{arom}$); 5.46 (d, 1H, *J* = 1.7 Hz, H-1); 5.08 (d, 1H, *J* = 1.7 Hz, H-1'); 4.84 (quint, 2H, *J* = 6.4 Hz, C*H*$_{Phth}$); 4.39 (d, 1H, *J* = 7.7 Hz, H-1"); 4.22 (dd, 1H, *J* = 2.4, 2.8 Hz, H-2); 4.20 (s, 1H, H-2'); 3.82 (dq, 1H, *J* = 3.4, 6.0 Hz, H-5'); 3.76 (dq, 1H, *J* = 3.4, 6.0 Hz, H-5); 3.71 (s, 1H, 2"-O*H*); 3.69 (s, 3H, OC*H*$_3$); 3.66-3.63 (m, 4H, H-3, H-3', H-6"); 3.61-3.57 (m, 2H, H-4, H-4'); 3.55 (dt, 1H, *J* = 2.0, 8.9 Hz, H-4"); 3.51 (s, 3H, OC*H*$_3$); 3.51 (s, 3H, OC*H*$_3$); 3.45-3.39 (m, 2H, H-2", H-5"); 3.33 (s, 3H, OC*H*$_3$); 3.18 (t, 1H, *J* = 8.9 Hz, H-3"); 2.87-2.85 (m, 2H, C*H*$_{Phth}$, 4"-O*H*); 2.55-2.53 (m, 4H, C*H*$_{2,Phth}$, C*H*$_{Myc}$); 2.34 (bs, 2H, 4-O*H*, 2'-O*H*); 1.77-0.81 (m, 189H, H-6, H-6', C*H*$_{Phth}$, C*H*$_{2,Phth}$, C*H*$_{3,Phth}$, C*H*$_{Myc}$, C*H*$_{2,Myc}$, C*H*$_{3,Myc}$). ^{13}C-APT NMR (214 MHz) δ: 176.2, 176.1 (*C*O$_{Myc}$); 154.3, 137.0 (C$_{q,arom}$); 129.5, 116.1 (*C*H$_{arom}$); 105.7 (C-1"); 100.6 (C-1'); 97.4 (C-1); 86.8 (*C*H$_{Phth}$); 85.4 (C-3"); 81.2 (C-3); 81.1 (C-4'); 80.6 (C-3'); 75.2 (C-2"); 74.3 (C-5"); 73.0 (C-6"); 72.5 (C-2); 71.8 (C-4); 71.4 (C-4"); 70.4, 70.4 (*C*H$_{Phth}$); 69.0 (C-5); 67.9 (C-5'); 66.9 (C-2'); 60.8, 59.8, 57.7, 57.5, 56.9 (O*C*H$_3$); 45.6, 45.6, 45.4 45.4 (*C*H$_{2,Myc}$); 41.1, 41.1, 38.5 (*C*H$_{2,Phth}$); 37.9, 37.9 (*C*H$_{Myc}$); 36.7, 35.3 (*C*H$_{2,Myc}$); 34.9 (*C*H$_{Phth}$); 34.8, 32.8 (*C*H$_{2,Phth}$); 32.1 (*C*H$_{2,Myc}$); 31.9, 30.2 (*C*H$_{2,Phth}$); 30.0 (*C*H$_{Myc}$); 29.9, 29.9, 29.9, 29.9, 29.9, 29.9, 29.8, 29.8, 29.7, 29.7, 29.7, 29.6, 29.5 (*C*H$_2$); 28.1 (*C*H$_{Myc}$); 27.6 (*C*H$_{2,Phth}$); 27.3 (*C*H$_{Myc}$); 27.1 (*C*H$_{2,Myc}$); 25.7, 25.3 (*C*H$_{2,Phth}$); 22.8 (*C*H$_{2,Myc}$); 22.4 (*C*H$_{2,Phth}$); 20.9, 20.6, 20.5, 20.5, 18.6 (*C*H$_{3,Myc}$); 17.9 (C-6); 17.6 (C-6'); 14.8 (*C*H$_{3,Phth}$); 14.3 (*C*H$_{3,Myc}$); 10.2 (*C*H$_{3,Phth}$). IR (thin film, cm^{-1}): 1016, 1066, 1132, 1232, 1261,

1378, 1457, 1511, 1736, 2853, 2923, 3451. HRMS calculated for $C_{121}H_{227}O_{19}$ 1985.68253 $[M+H]^+$; found 1985.68284.

4-((3*R*,4*S*,9*R*,11*R*)-3-methoxy-4-methylheptacos-26-yne-9,11-diyl bismycocerosate)phenyl 2-*O*-(2,3-di-*O*-methyl-4-*O*-(6-*O*-methyl-ß-D-glucopyranosyl)-α-L-rhamnopyranosyl)-3-*O*-methyl-α-L-rhamnopyranoside (26)

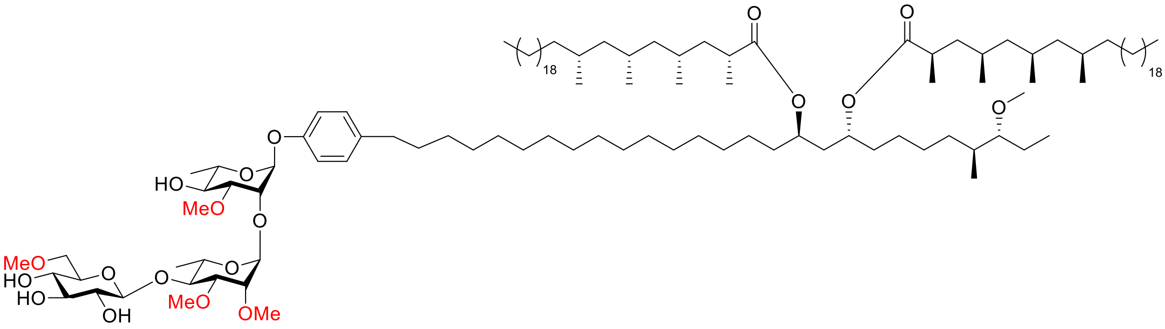

The title compound was synthesized according to general procedure D using **23** (24 mg, 10 μmol, 1.0 eq) and Pd/C (10%, 11 mg, 10 μmol, 1.0 eq). Column chromatography (DCM-MeOH 19:1) yielded the product (8 mg, 4 μmol, 40%) as a waxy solid. $[\alpha]_D^{25}$ = -28 (c = 0.2, $CHCl_3$). ^{1}H-NMR (400 MHz) δ: 7.10 (d, 2H, J = 8.8 Hz, CH_{arom}); 6.94 (d, 2H, J = 8.8 Hz, CH_{arom}); 5.43 (d, 1H, J = 2.0 Hz, H-1); 5.11 (d, 1H, J = 1.6 Hz, H-1'); 4.84 (quint, 2H, J = 6.4 Hz, CH_{Phth}); 4.45 (d, 1H, J = 7.6 Hz, H-1"); 4.23 (dd, 1H, J = 1.6, 2.8 Hz, H-2); 3.99 (bs, 1H, OH); 3.79-3.71 (m, 3H, H-2', H-5, H-5'); 3.69-3.60 (m, 5H, H-3, H-3', H-4', H-6"); 3.58-3.54 (m, 6H, H-3", H-4, H-5", OCH_3); 3.50 (s, 3H, OCH_3); 3.49 (s, 3H, OCH_3); 3.46-3.42 (m, 1H, H-4"); 3.39-3.36 (m, 4H, H-2", OCH_3); 3.35 (s, 3H, OCH_3); 2.98 (bs, 1H, OH); 2.88-2.79 (m, 2H, CH_{Phth}, OH); 2.57-2.50 (m, 4H, $CH_{2,Phth}$, CH_{Myc}); 2.32 (bs, 1H, OH); 1.77-0.81 (m, 207H, H-6, H-6', CH_{Phth}, $CH_{2,Phth}$, $CH_{3,Phth}$, CH_{Myc}, $CH_{2,Myc}$, $CH_{3,Myc}$,). ^{13}C-APT NMR (101 MHz) δ: 176.2 (CO_{Myc}); 154.3, 137.0 ($C_{q,arom}$); 129.5, 116.1 (CH_{arom}); 105.3 (C-1"); 98.5 (C-1'); 97.5 (C-1); 86.8 (CH_{Phth}); 81.5 (C-3); 81.5 (C-4'); 80.3 (C-3'); 76.7 (C-3"); 75.9 (C-2'); 74.8 (C-2"); 74.0 (C-4"); 73.1 (C-6"); 72.3 (C-2); 72.0 (C-4); 71.9 (C-5"); 70.4 (CH_{Phth}); 69.1 (C-5); 68.3 (C-5'); 59.8, 59.1, 57.8, 57.5, 56.7 (OCH_3); 45.6, 45.4 ($CH_{2,Myc}$); 41.1, 38.6 ($CH_{2,Phth}$); 37.9 (CH_{Myc}); 36.8, 35.3 ($CH_{2,Myc}$); 34.9 (CH_{Phth}); 34.8, 32.8 ($CH_{2,Phth}$); 32.1 ($CH_{2,Myc}$); 31.9, 30.2 ($CH_{2,Phth}$); 30.1 (CH_{Myc}); 29.9, 29.9, 29.8, 29.7, 29.6, 29.5 (CH_2); 28.2 (CH_{Myc}); 27.6 ($CH_{2,Phth}$); 27.3 (CH_{Myc}); 27.1 ($CH_{2,Myc}$); 25.7, 25.3 ($CH_{2,Phth}$); 22.9 ($CH_{2,Myc}$); 22.5 ($CH_{2,Phth}$); 20.9, 20.6, 20.6 18.6, 18.5 ($CH_{3,Myc}$); 17.9 (C-6); 17.7 (C-6'); 14.8 ($CH_{3,Phth}$); 14.3 ($CH_{3,Myc}$); 10.3 ($CH_{3,Phth}$). IR (thin film, cm^{-1}): 1016, 1069, 1123, 1229, 1261, 1378, 1457, 1511, 1736, 2853, 2923, 3436. HRMS calculated for $C_{121}H_{227}O_{19}$ 1985.68253 $[M+H]^+$; found 1985.68265.

PGL-III analogues synthesis

4-(hex-1-ynyl)phenyl 2-*O*-(2,3-di-*O*-methyl-4-*O*-(2-*O*-benzyloxycarbonyl-3,4-di-*O*-benzyl-6-*O*-methyl-ß-D-glucopyranosyl)-α-L-rhamnopyranosyl)-3-*O*-methyl-4-*O*-benzyl-α-L-rhamnopyranoside (27)

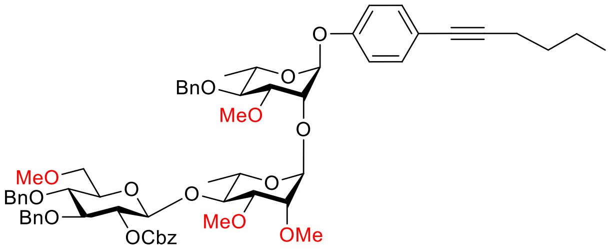

The title compound was synthesized according to general procedure B using glycan **17** (46 mg, 41 μmol, 1.0 eq) and 1-hexyne (14 μL, 122 μmol, 3.0 eq). Column chromatography (*n*-pentane-Et_2O 4:6) yielded the product (40 mg, 37 μmol, 91%) as a yellow oil. $[\alpha]_D^{25}$ = -60.9 (c = 1.0, $CHCl_3$). ^{1}H-NMR (400 MHz) δ: 7.37-7.21 (m, 22H, CH_{arom}); 6.96-6.94 (m, 2H, CH_{arom}); 5.48 (d, 1H, *J* = 1.6 Hz, H-1); 5.24-5.15 (m, 3H, H-1', PhC$H_{2,Cbz}$); 4.89 (d, 1H, *J* = 11.2 Hz, PhC*H*H); 4.80-4.76 (m, 4H, PhC*H*H, PhC*H*H, H-1'', H-2''); 4.70-4.62 (m, 3H, PhCH*H*, PhCH*H*, PhCH*H*); 4.25 (dd, 1H, *J* = 2.4, 2.8 Hz, H-2); 3.79 (dd, 1H, *J* = 3.0, 9.4 Hz, H-3); 3.76-3.61 (m, 7H, H-2', H-3'', H-4'', H-5, H-5', H-5'', H-6''); 3.57-3.43 (m, 9H, H-4, H-4', H-6'', OCH_3); 3.39-3.32 (m, 7H, H-3', OCH_3); 2.39 (t, 1H, *J* = 7.0 Hz, CH_2); 1.63-1.52 (m, 2H, CH_2); 1.51-1.42 (m, 2H, CH_2); 1.32-1.25 (m, 6H, H-6, H-6'); 0.94 (t, 3H, *J* = 7.4 Hz, CH_3). ^{13}C-APT NMR (101 MHz) δ: 155.3 ($C_{q,arom}$); 154.7 (CO_{Cbz}); 138.5, 138.4, 138.2, 135.5 ($C_{q,arom}$); 133.0, 128.8, 128.6, 128.6, 128.5, 128.4, 128.2, 128.1, 128.0, 127.9, 127.8, 127.7 (CH_{arom}); 118.0 ($C_{q,arom}$); 116.2 (CH_{arom}); 101.1 (C-1''); 98.5 (C-1'); 96.9 (C-1); 89.4 ($C_{q,alkyne}$); 83.3 (C-4''); 82.0 (C-3); 80.8, 80.1 (C-4 and C-4'); 80.1 ($C_{q,alkyne}$); 78.2 (C-2''); 77.9, 77.7 (C-3'', C-5''); 77.0 (C-2'); 75.4, 75.2, 75.1 (PhCH_2); 75.0 (C-3') 73.0 (C-2); 71.1 (C-6''); 69.9 (PhCH_2); 68.7 (C-5); 68.0 (C-5'); 59.9, 59.1, 58.3, 57.6, (OCH_3); 31.0, 22.1, 19.2 (CH_2); 18.2, 18.1 (C-6 and C-6'); 13.8 (CH_3). IR (thin film, cm^{-1}): 1030, 1055, 1072, 1093, 1120, 1140, 1258, 1387, 1454, 1507, 1757, 2929. HRMS calculated for $C_{63}H_{76}O_{16}Na$ 1111.50256 $[M+Na]^+$; found 1111.50185.

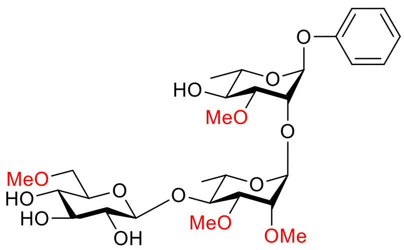

Phenyl 2-*O*-(2,3-di-*O*-methyl-4-*O*-(6-*O*-methyl-ß-D-glucopyranosyl)-α-L-rhamnopyranosyl)-3-*O*-methyl-α-L-rhamnopyranoside (HD-275, 28)

Compound **17** (34 mg, 30 μmol, 1.0 eq) was hydrogenated using general procedure D to give the title compound (14 mg, 23 μmol, 77%) as a pale oil. $[\alpha]_D^{25}$ = -53.7 (c = 1.0, $CHCl_3$). ^{1}H-NMR (400 MHz) δ: 7.33-

7.28 (m, 2H, C*H*arom); 7.05-7.02 (m, 3H, C*H*arom); 5.49 (d, 1H, *J* = 1.6 Hz, H-1); 5.11 (d, 1H, *J* = 1.2 Hz, H-1'); 4.45 (d, 1H, *J* = 7.6 Hz, H-1"); 4.24 (dd, 1H, *J* = 1.6, 3.2 Hz, H-2); 4.12 (bs, 1H, O*H*); 3.79-3.59 (m, 8H, H-2', H-3, H-3', H-4', H-5, H-5', H-6"); 3.57-3.35 (m, 18H, H-2", H-3", H-4, H-4", H-5", OC*H*3); 2.52 (bs, 1H, O*H*); 1.34 (d, 3H, *J* = 6.4 Hz, H-6'); 1.27 (d, 3H, *J* = 6.0 Hz, H-6). ^{13}C-APT NMR (101 MHz) δ: 156.2 ($C_{q,arom}$); 129.7, 122.4, 116.3 (*C*Harom); 105.2 (C-1"); 98.5 (C-1'); 97.2 (C-1); 81.5, (C-3); 81.3 (C-4'); 80.3 (C-3'); 76.6 (C-3"); 75.9 (C-2'); 74.8 (C-2"); 74.3 (C-4"); 72.9 (C-6"); 72.3 (C-2); 71.9 (C-4); 71.5 (C-5"); 69.2 (C-5); 68.3 (C-5'); 59.8, 59.1, 57.8, 56.7 (O*C*H3); 17.9 (C-6); 17.7 (C-6'). IR (thin film, cm^{-1}): 1009, 1067, 1118, 1202, 1229, 1457, 2931, 3400. HRMS calculated for $C_{28}H_{44}O_{14}Na$ 627.26233 $[M+Na]^+$; found 627.26222.

4-hexylphenyl 2-*O*-(2,3-di-*O*-methyl-4-*O*-(6-*O*-methyl-ß-D-glucopyranosyl)-α-L-rhamnopyranosyl)-3-*O*-methyl-α-L-rhamnopyranoside (HD-276, 29)

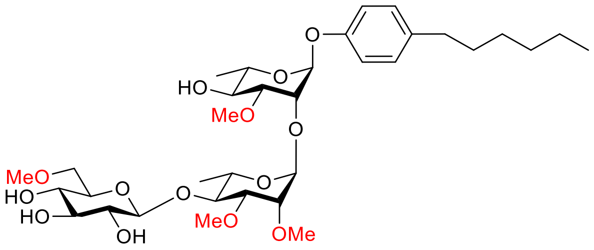

Compound **27** (32 mg, 29 μmol, 1.0 eq) was hydrogenated using general procedure D to give the title compound (15 mg, 22 μmol, 74%) as a pale oil. $[\alpha]_D^{25}$ = -50.3 (c = 1.0, $CHCl_3$). ^{1}H-NMR (400 MHz) δ: 7.10 (d, 2H, *J* = 8.4 Hz, C*H*arom); 6.95 (dd, 2H, *J* = 2.0, 6.8 Hz, C*H*arom); 5.44 (d, 1H, *J* = 2.0 Hz, H-1); 5.10 (d, 1H, *J* = 1.6 Hz, H-1'); 4.45 (d, 1H, *J* = 7.6 Hz, H-1"); 4.24 (dd, 1H, *J* = 2.0, 2.8 Hz, H-2); 4.09 (bs, 1H, O*H*); 3.78-3.59 (m, 8H, H-2', H-3, H-3', H-4', H-5, H-5', H-6"); 3.57-3.35 (m, 18H, H-2", H-3", H-4, H-4", H-5", OC*H*3); 3.21 (bs, 2H, O*H*); 2.55 (t, 2H, *J* = 7.6 Hz, C*H*2); 2.45 (bs, 1H, O*H*); 1.58 (quint, 2H, *J* = 7.2 Hz, C*H*2); 1.33-1.25 (m, 12H, C*H*2, H-6, H-6'); 0.88 (t, 3H, *J* = 6.8 Hz, C*H*3). ^{13}C-APT NMR (101 MHz) δ: 154.3, 137.0 ($C_{q,arom}$); 129.5, 116.1 (*C*Harom); 105.2 (C-1"); 98.4 (C-1'); 97.4 (C-1); 81.5, (C-3); 81.4 (C-4'); 80.3 (C-3'); 76.6 (C-3"); 75.9 (C-2'); 74.8 (C-2"); 74.2 (C-4"); 73.0 (C-6"); 72.3 (C-2); 71.9 (C-4); 71.6 (C-5"); 69.1 (C-5); 68.3 (C-5'); 59.8, 59.1, 57.8, 56.7 (O*C*H3); 35.3, 31.9, 31.8, 29.1, 22.8 (*C*H2); 17.9 (C-6); 17.7 (C-6'); 14.2 (*C*H3). IR (thin film, cm^{-1}): 1013, 1066, 1116, 1457, 1510, 2856, 2929, 3427. HRMS calculated for $C_{34}H_{56}O_{14}Na$ 711.35623 $[M+Na]^+$; found 711.35585.

References

(1) Feinberg, H.; Rambaruth, N. D.; Jegouzo, S. A.; Jacobsen, K. M.; Djurhuus, R.; Poulsen, T. B.; Weis, W. I.; Taylor, M. E.; Drickamer, K. Binding Sites for Acylated Trehalose Analogs of Glycolipid Ligands on an Extended Carbohydrate Recognition Domain of the Macrophage Receptor Mincle. *J Biol Chem* **2016**, *291* (40), 21222-21233. DOI: 10.1074/jbc.M116.749515 From NLM Medline.

(2) Yamasaki, S.; Matsumoto, M.; Takeuchi, O.; Matsuzawa, T.; Ishikawa, E.; Sakuma, M.; Tateno, H.; Uno, J.; Hirabayashi, J.; Mikami, Y.; et al. C-type lectin Mincle is an activating receptor for pathogenic fungus, Malassezia. *Proc Natl Acad Sci U S A* **2009**, *106* (6), 1897-1902. DOI: 10.1073/pnas.0805177106.

(3) Matsuoka, M. The history of Mycobacterium leprae Thai-53 strain. *Lepr Rev* **2010**, *81* (2), 137.

(4) Morita, Y. S.; Velasquez, R.; Taig, E.; Waller, R. F.; Patterson, J. H.; Tull, D.; Williams, S. J.; Billman-Jacobe, H.; McConville, M. J. Compartmentalization of lipid biosynthesis in mycobacteria. *J Biol Chem* **2005**, *280* (22), 21645-21652. DOI: 10.1074/jbc.M414181200.

(5) Yamasaki, S.; Ishikawa, E.; Sakuma, M.; Hara, H.; Ogata, K.; Saito, T. Mincle is an ITAM-coupled activating receptor that senses damaged cells. *Nat Immunol* **2008**, *9* (10), 1179-1188. DOI: 10.1038/ni.1651.

(6) Kiyotake, R.; Oh-Hora, M.; Ishikawa, E.; Miyamoto, T.; Ishibashi, T.; Yamasaki, S. Human Mincle Binds to Cholesterol Crystals and Triggers Innate Immune Responses. *J Biol Chem* **2015**, *290* (42), 25322-25332. DOI: 10.1074/jbc.M115.645234.

(7) Ishikawa, T.; Itoh, F.; Yoshida, S.; Saijo, S.; Matsuzawa, T.; Gonoi, T.; Saito, T.; Okawa, Y.; Shibata, N.; Miyamoto, T.; et al. Identification of distinct ligands for the C-type lectin receptors Mincle and Dectin-2 in the pathogenic fungus Malassezia. *Cell Host Microbe* **2013**, *13* (4), 477-488. DOI: 10.1016/j.chom.2013.03.008.

(8) Kohsaka, K.; Matsuoka, M.; Hirata, T.; Nakamura, M. Preservation of Mycobacterium leprae in vitro for four years by lyophilization. *Int J Lepr Other Mycobact Dis* **1993**, *61* (3), 415-420. From NLM Medline.

(9) Shepard, C. C.; McRae, D. H. A method for counting acid-fast bacteria. *Int J Lepr Other Mycobact Dis* **1968**, *36* (1), 78-82. From NLM Medline.

(10) Miyamoto, Y.; Mukai, T.; Naka, T.; Fujiwara, N.; Maeda, Y.; Kai, M.; Mizuno, S.; Yano, I.; Makino, M. Novel rhamnosyltransferase involved in biosynthesis of serovar 4-specific glycopeptidolipid from Mycobacterium avium complex. *J Bacteriol* **2010**, *192* (21), 5700-5708. DOI: 10.1128/JB.00554-10 From NLM Medline.

(11) Jablonski, K. A.; Amici, S. A.; Webb, L. M.; Ruiz-Rosado Jde, D.; Popovich, P. G.; Partida-Sanchez, S.; Guerau-de-Arellano, M. Novel Markers to Delineate Murine M1 and M2 Macrophages. *PLoS One* **2015**, *10* (12), e0145342. DOI: 10.1371/journal.pone.0145342.

(12) Goto, M.; Nagatomo, Y.; Hasui, K.; Yamanaka, H.; Murashima, S.; Sato, E. Chromaticity analysis of immunostained tumor specimens. *Pathol Res Pract* **1992**, *188* (4-5), 433-437. DOI: 10.1016/S0344-0338(11)80033-6 From NLM Medline.

(13) Kabsch, W. Xds. *Acta Crystallogr D Biol Crystallogr* **2010**, *66* (Pt 2), 125-132. DOI: 10.1107/S0907444909047337.

(14) Evans, P. Scaling and assessment of data quality. *Acta Crystallogr D Biol Crystallogr* **2006**, *62* (Pt 1), 72-82. DOI: 10.1107/S0907444905036693 From NLM Medline.

(15) Vagin, A.; Teplyakov, A. Molecular replacement with MOLREP. *Acta Crystallogr D Biol Crystallogr* **2010**, *66* (Pt 1), 22-25. DOI: 10.1107/S0907444909042589.

(16) Emsley, P.; Lohkamp, B.; Scott, W. G.; Cowtan, K. Features and development of Coot. *Acta Crystallogr D Biol Crystallogr* **2010**, *66* (Pt 4), 486-501. DOI: 10.1107/S0907444910007493.

(17) Vagin, A. A.; Steiner, R. A.; Lebedev, A. A.; Potterton, L.; McNicholas, S.; Long, F.; Murshudov, G. N. REFMAC5 dictionary: organization of prior chemical knowledge and guidelines for its use. *Acta Crystallogr D Biol Crystallogr* **2004**, *60* (Pt 12 Pt 1), 2184-2195. DOI: 10.1107/S0907444904023510.

(18) Afonine, P. V.; Grosse-Kunstleve, R. W.; Echols, N.; Headd, J. J.; Moriarty, N. W.; Mustyakimov, M.; Terwilliger, T. C.; Urzhumtsev, A.; Zwart, P. H.; Adams, P. D. Towards automated crystallographic structure refinement with phenix.refine. *Acta Crystallogr D Biol Crystallogr* **2012**, *68* (Pt 4), 352-367. DOI: 10.1107/S0907444912001308.

(19) Williams, C. J.; Headd, J. J.; Moriarty, N. W.; Prisant, M. G.; Videau, L. L.; Deis, L. N.; Verma, V.; Keedy, D. A.; Hintze, B. J.; Chen, V. B.; et al. MolProbity: More and better reference data for improved all-atom structure validation. *Protein Sci* **2018**, *27* (1), 293-315. DOI: 10.1002/pro.3330.
(20) van Dijk, J. H. M.; van Hooij, A.; Groot, L. M.; Geboers, J.; Moretti, R.; Verhard-Seymonsbergen, E.; de Jong, D.; van der Marel, G. A.; Corstjens, P.; Codee, J. D. C.; et al. Synthetic Phenolic Glycolipids for Application in Diagnostic Tests for Leprosy. *Chembiochem* **2021**, *22* (8), 1487-1493. DOI: 10.1002/cbic.202000810 From NLM Medline.
(21) Barroso, S.; Castelli, R.; Baggelaar, M. P.; Geerdink, D.; ter Horst, B.; Casas-Arce, E.; Overkleeft, H. S.; van der Marel, G. A.; Codee, J. D.; Minnaard, A. J. Total synthesis of the triglycosyl phenolic glycolipid PGL-tb1 from Mycobacterium tuberculosis. *Angew Chem Int Ed Engl* **2012**, *51* (47), 11774-11777. DOI: 10.1002/anie.201206221 From NLM Medline.
(22) ter Horst, B.; Feringa, B. L.; Minnaard, A. J. Catalytic asymmetric synthesis of mycocerosic acid. *Chem Commun (Camb)* **2007**, (5), 489-491. DOI: 10.1039/b612593j From NLM Medline.

NMRs of newly synthesized and isolated compounds

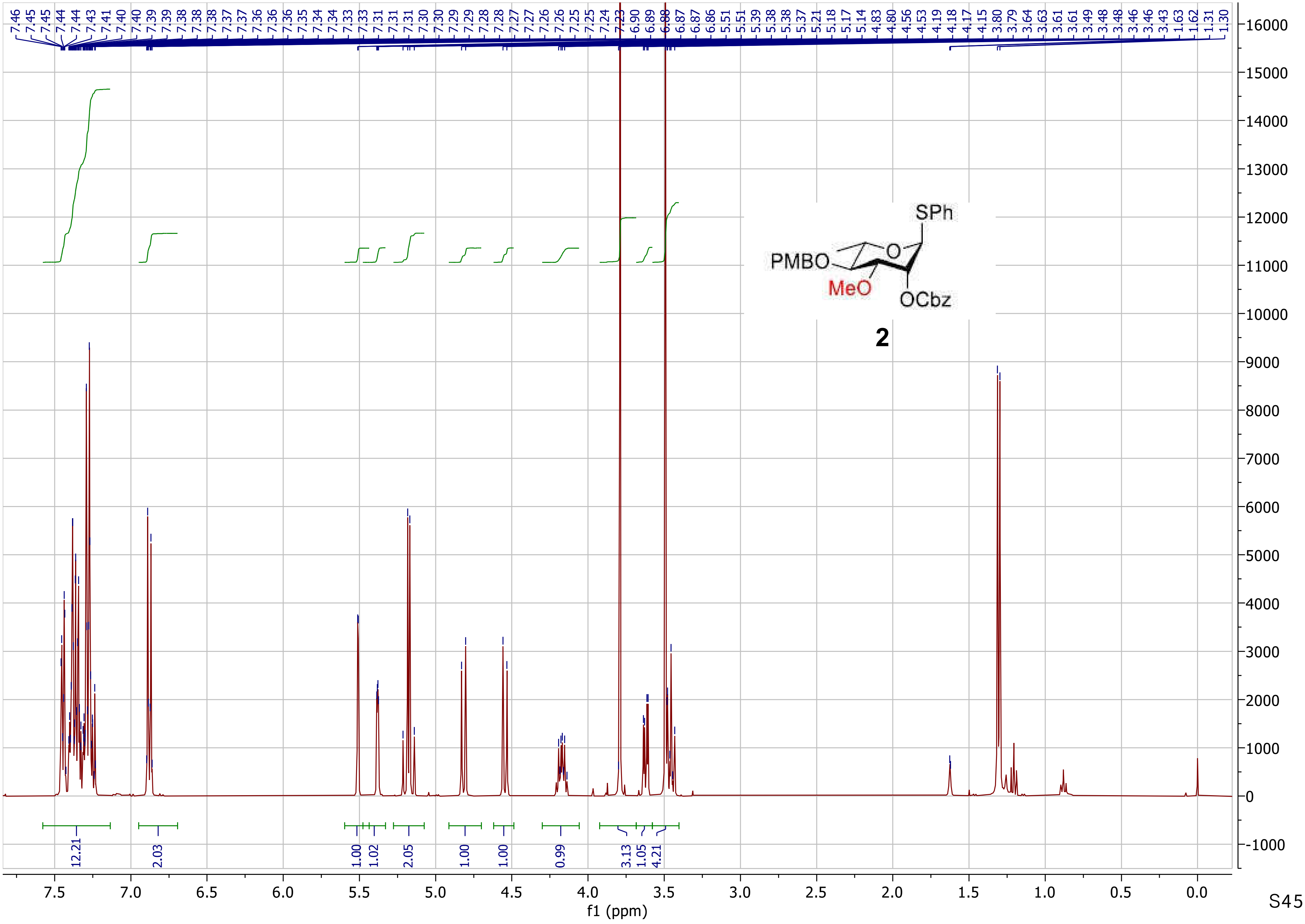
SPh
PMBO
O
MeO
OCbz
2
f1 (ppm)
12.21
2.03
1.00
1.02
2.05
1.00
1.00
0.99
3.13
1.05
4.21

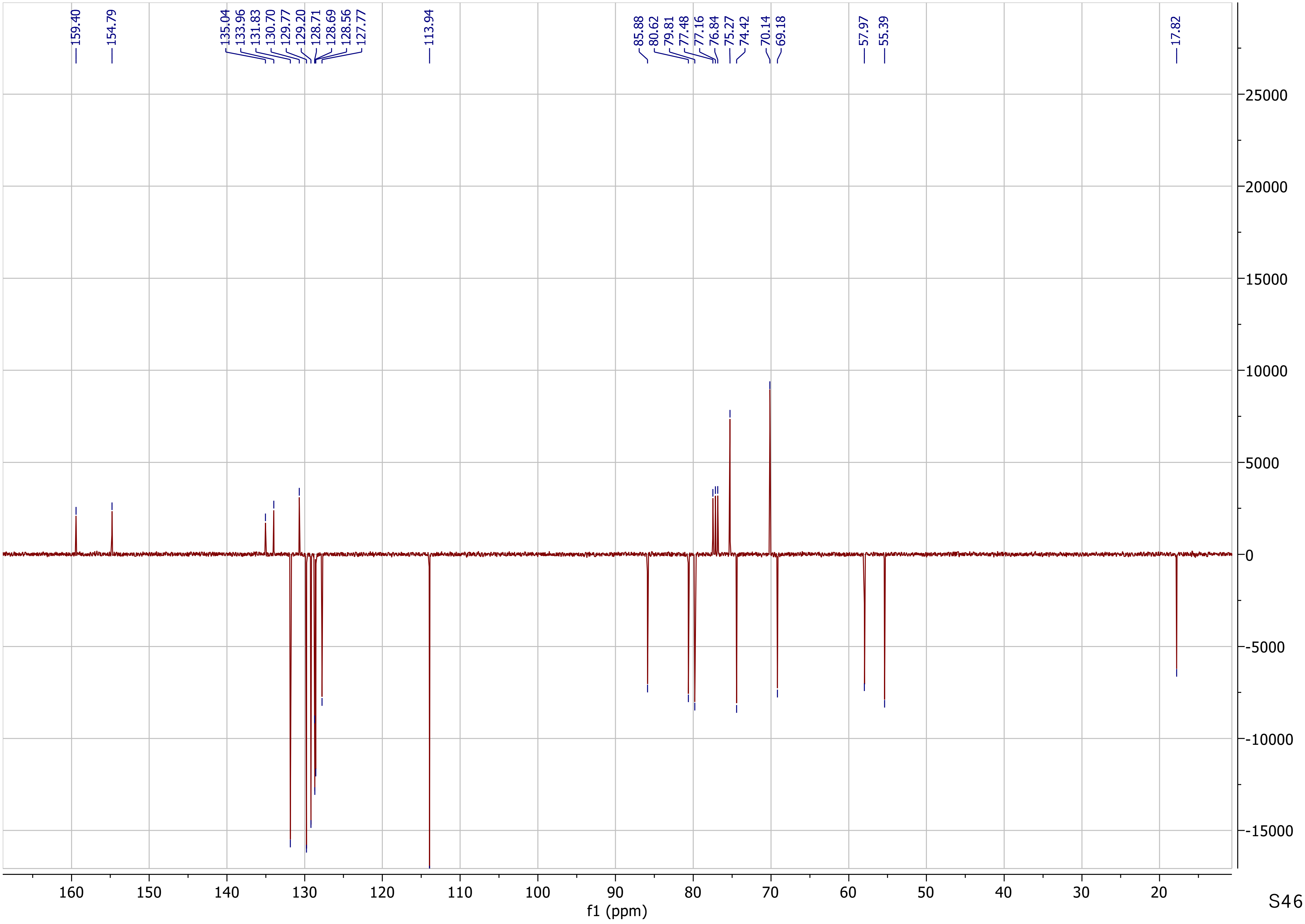
159.40
154.79
135.04
133.96
131.83
130.70
129.77
129.20
128.71
128.69
128.56
127.77
113.94
85.88
80.62
79.81
77.48
77.16
76.84
75.27
74.42
70.14
69.18
57.97
55.39
17.82
f1 (ppm)

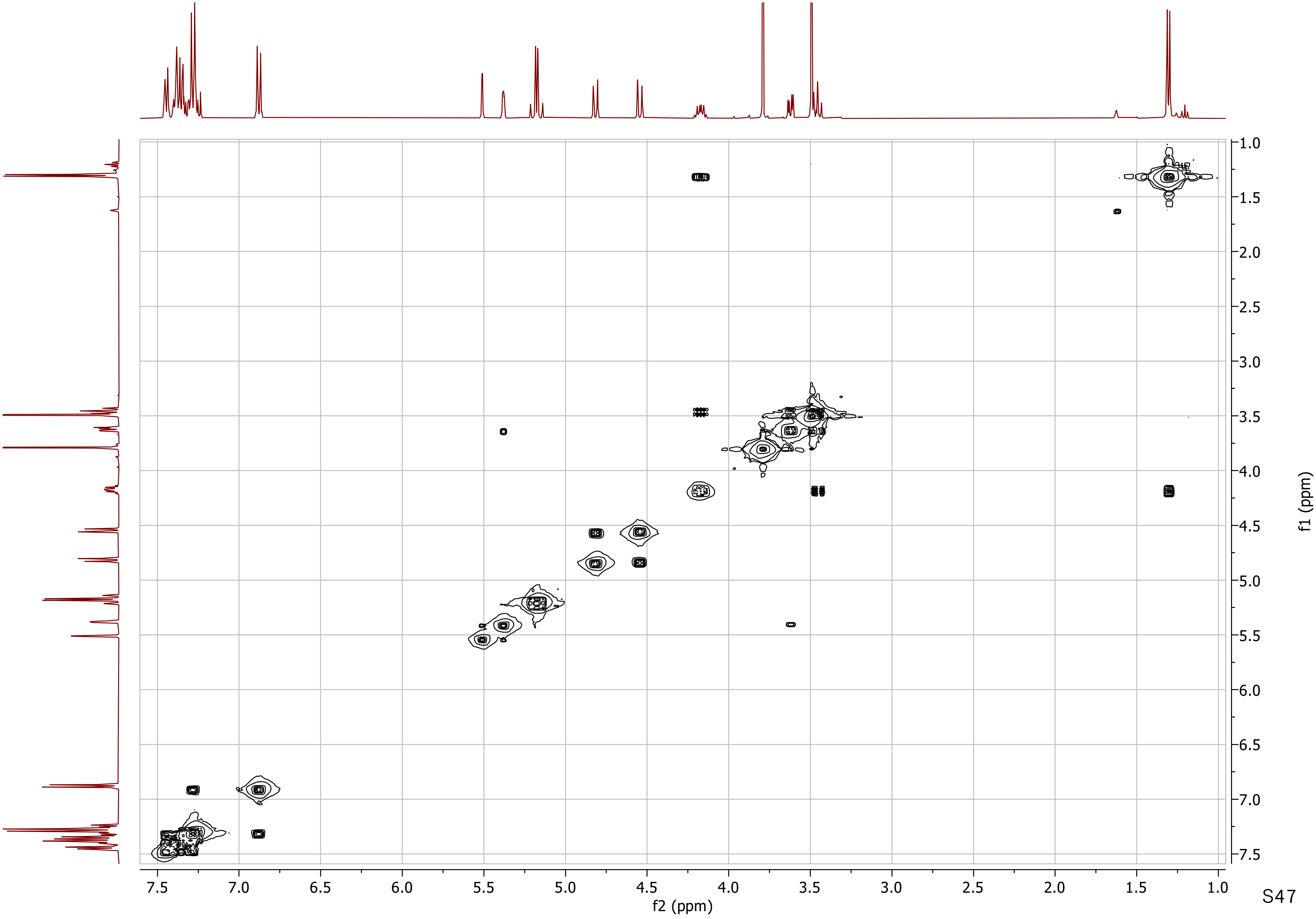

7.5
7.0
6.5
6.0
5.5
5.0
4.5
4.0
3.5
3.0
2.5
2.0
1.5
1.0
f2 (ppm)
f1 (ppm)

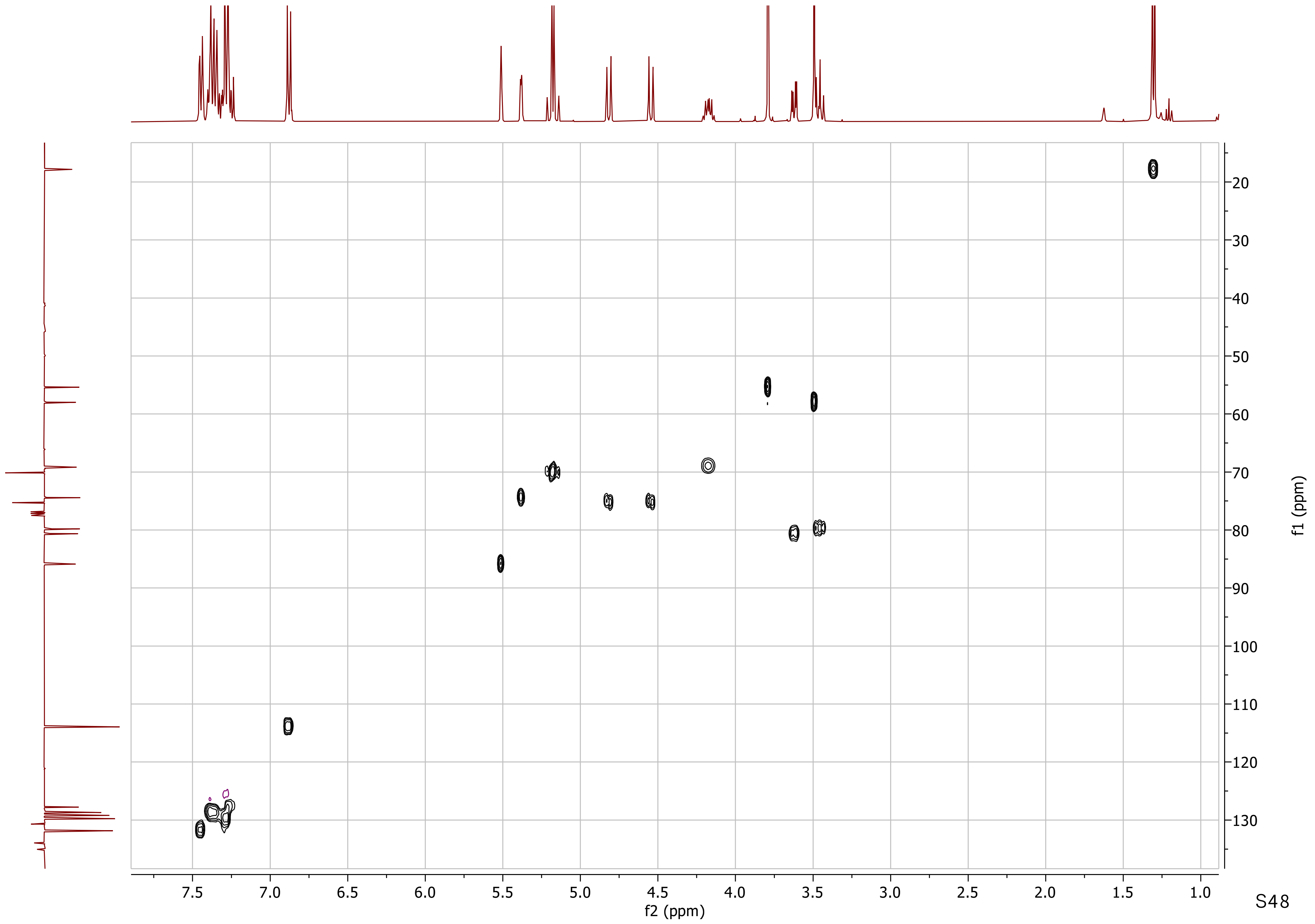

f2 (ppm)
f1 (ppm)

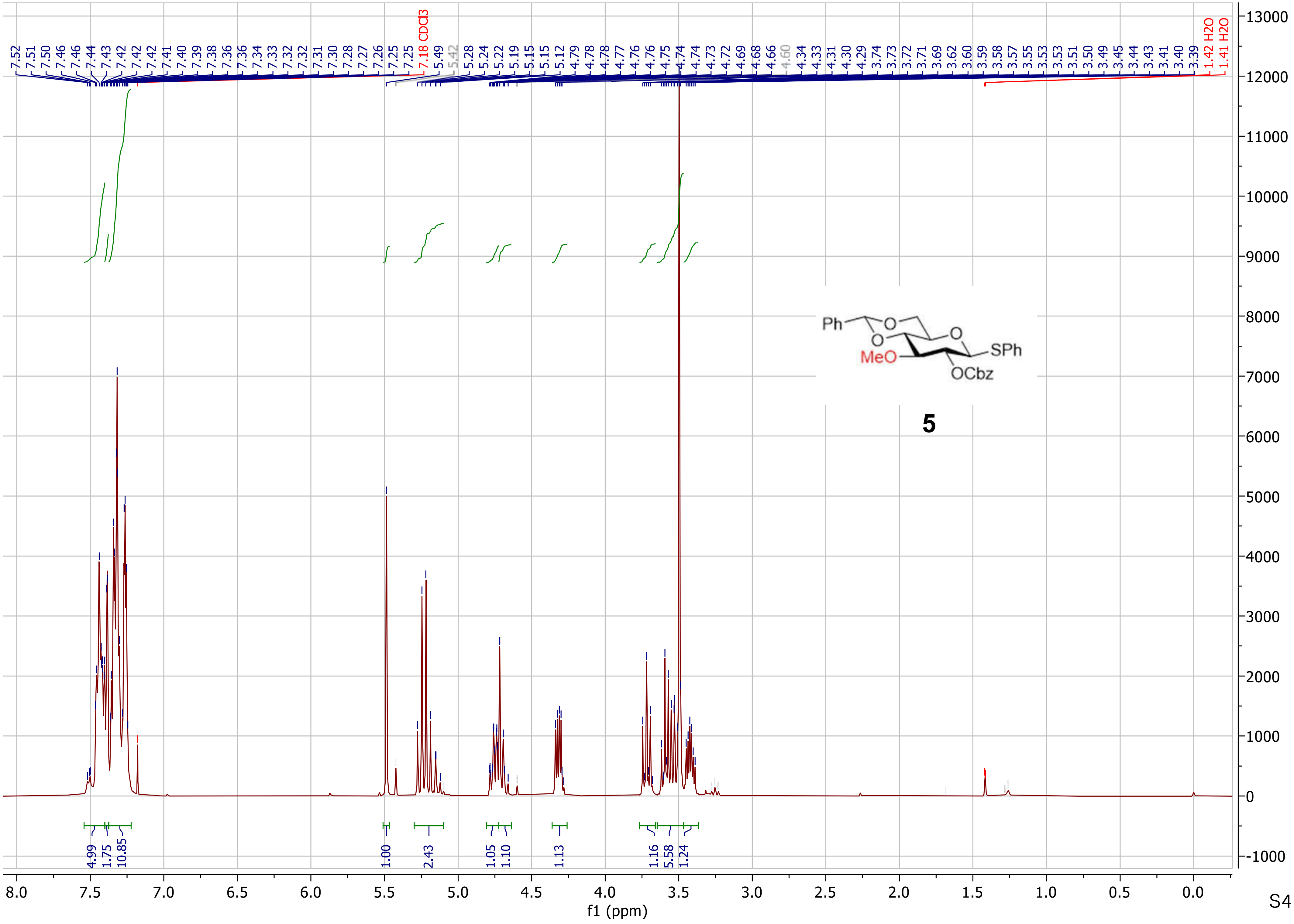
7.52 7.51 7.50 7.46 7.46 7.44 7.43 7.42 7.42 7.42 7.41 7.40 7.39 7.38 7.36 7.36 7.34 7.33 7.32 7.32 7.31 7.30 7.28 7.27 7.26 7.25 7.25 7.18 CDCl3 5.49 5.42 5.28 5.24 5.22 5.19 5.15 5.15 5.12 4.79 4.78 4.78 4.77 4.76 4.76 4.75 4.74 4.74 4.74 4.73 4.72 4.69 4.68 4.66 4.60 4.34 4.33 4.31 4.30 4.29 3.74 3.73 3.72 3.71 3.69 3.62 3.60 3.59 3.58 3.57 3.55 3.53 3.53 3.51 3.50 3.49 3.45 3.44 3.43 3.41 3.40 3.39 1.42 H2O 1.41 H2O
Ph
O
O
O
MeO
SPh
OCbz
5
4.99
1.75
10.85
1.00
2.43
1.05
1.10
1.13
1.16
5.58
1.24
f1 (ppm)

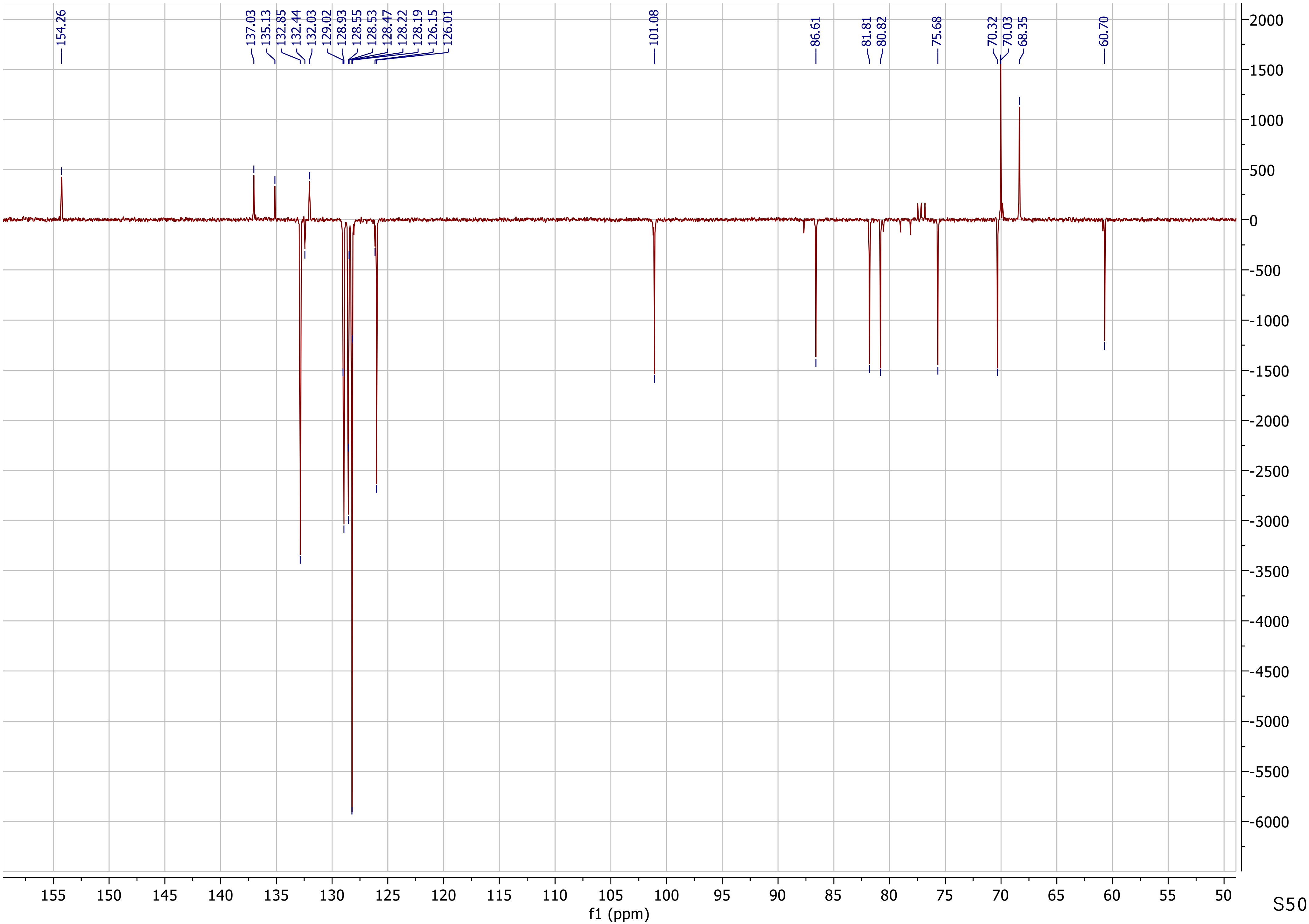
154.26
137.03
135.13
132.85
132.44
132.03
129.02
128.93
128.55
128.53
128.47
128.22
128.19
126.15
126.01
101.08
86.61
81.81
80.82
75.68
70.32
70.03
68.35
60.70
2000
1500
1000
500
0
-500
-1000
-1500
-2000
-2500
-3000
-3500
-4000
-4500
-5000
-5500
-6000
155 150 145 140 135 130 125 120 115 110 105 100 95 90 85 80 75 70 65 60 55 50
f1 (ppm)

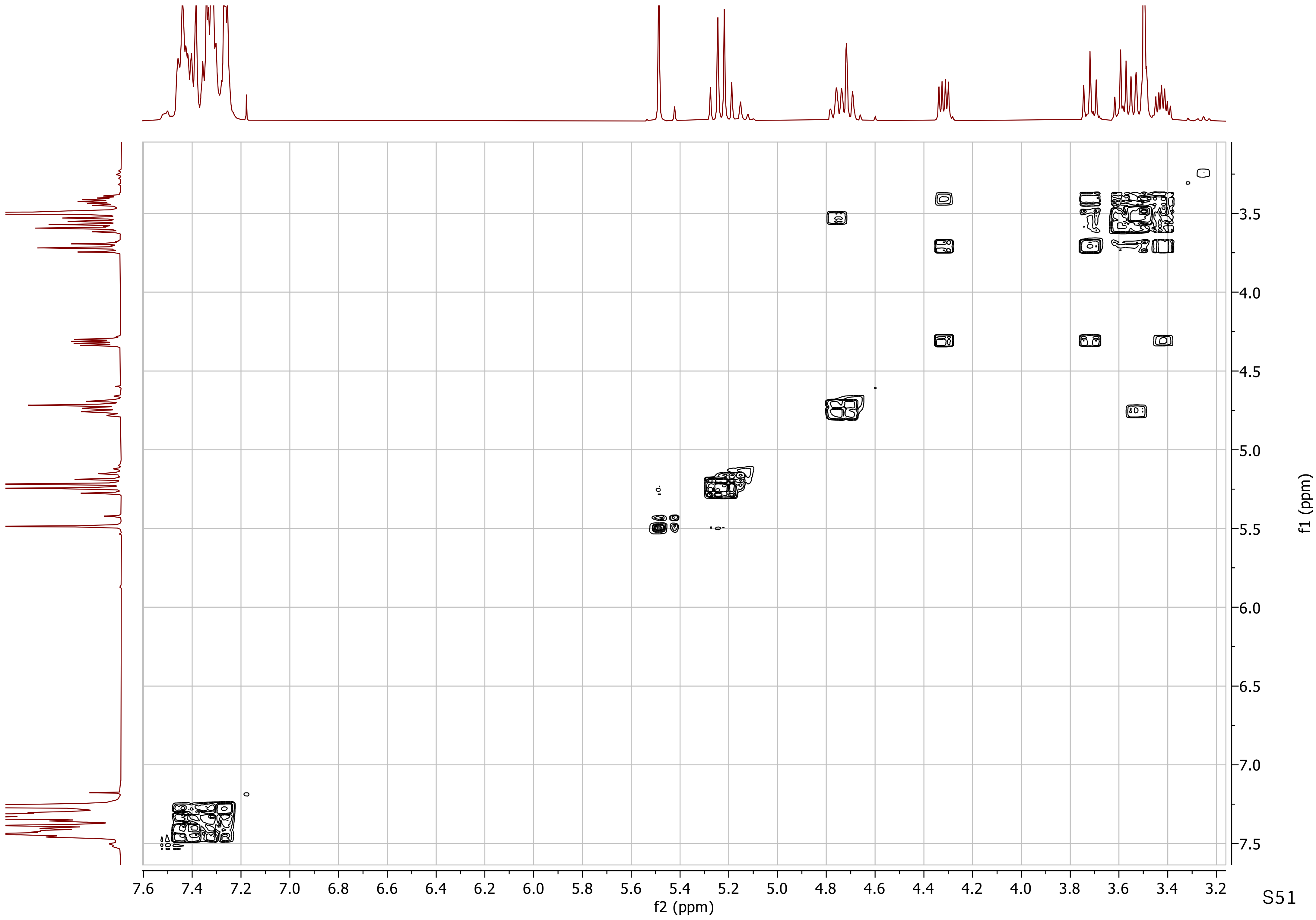

f1 (ppm)
f2 (ppm)

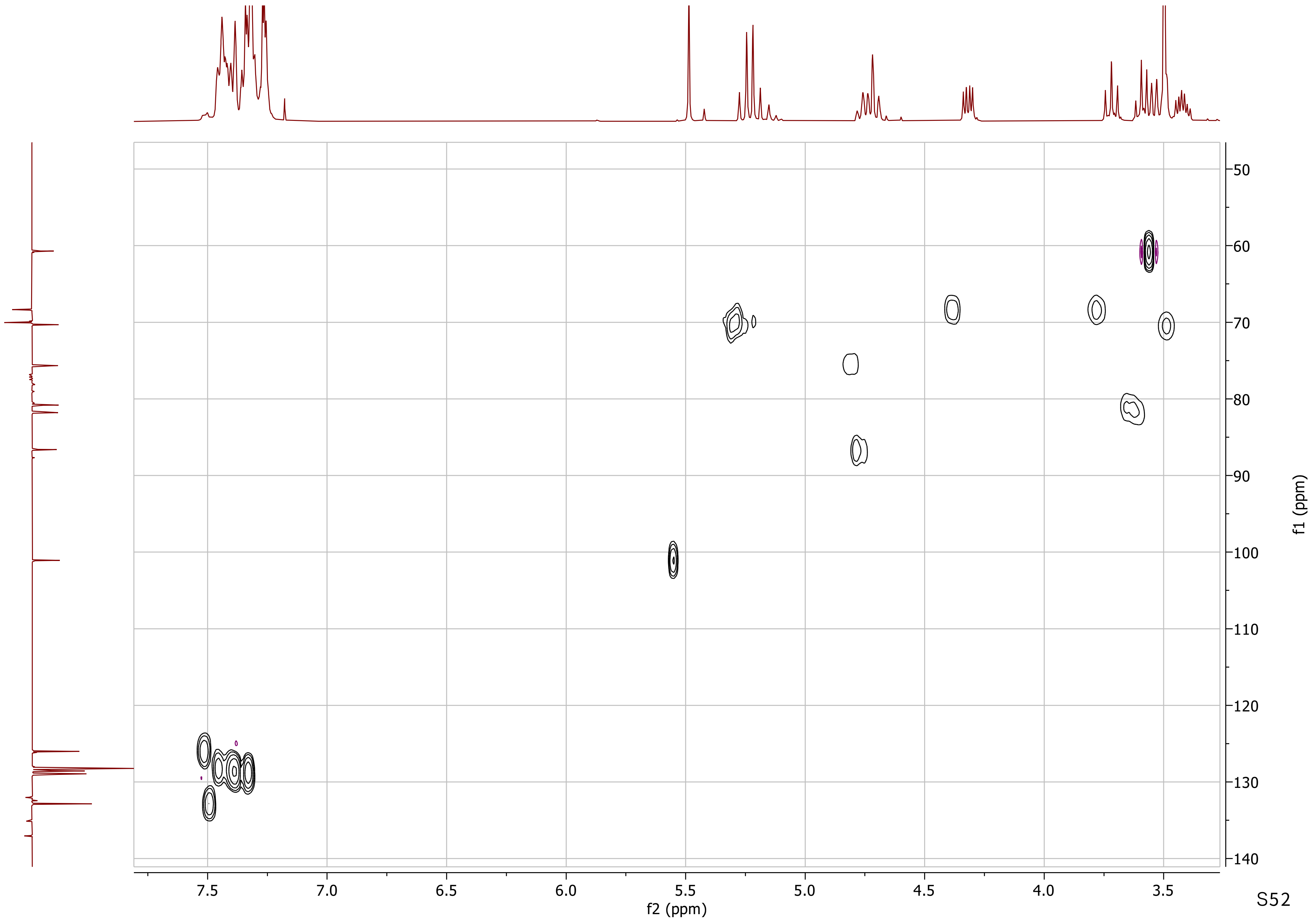

f2 (ppm)
f1 (ppm)
7.5
7.0
6.5
6.0
5.5
5.0
4.5
4.0
3.5
50
60
70
80
90
100
110
120
130
140

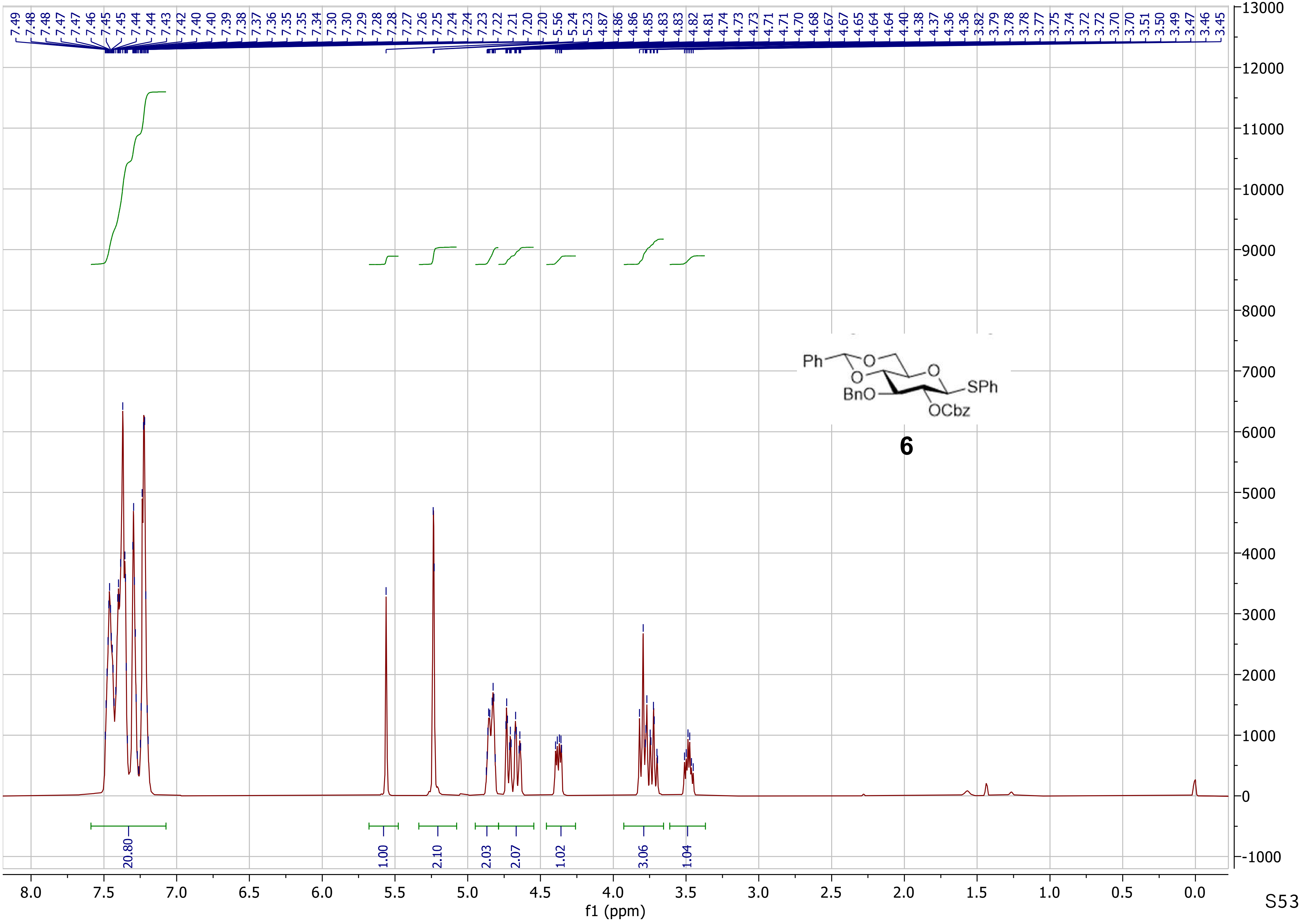
7.49 7.48 7.48 7.47 7.47 7.46 7.45 7.45 7.44 7.44 7.43 7.42 7.40 7.40 7.39 7.38 7.37 7.36 7.35 7.35 7.34 7.30 7.30 7.29 7.28 7.28 7.27 7.26 7.25 7.24 7.24 7.23 7.22 7.21 7.20 7.20 5.56 5.24 5.23 4.87 4.86 4.86 4.85 4.83 4.83 4.82 4.81 4.74 4.73 4.73 4.71 4.71 4.70 4.68 4.67 4.67 4.65 4.64 4.64 4.40 4.38 4.37 4.36 4.36 3.82 3.79 3.78 3.78 3.77 3.75 3.74 3.72 3.72 3.70 3.70 3.51 3.50 3.49 3.47 3.46 3.45
Ph
O
O
O
BnO
SPh
OCbz
6
20.80
1.00
2.10
2.03
2.07
1.02
3.06
1.04
f1 (ppm)

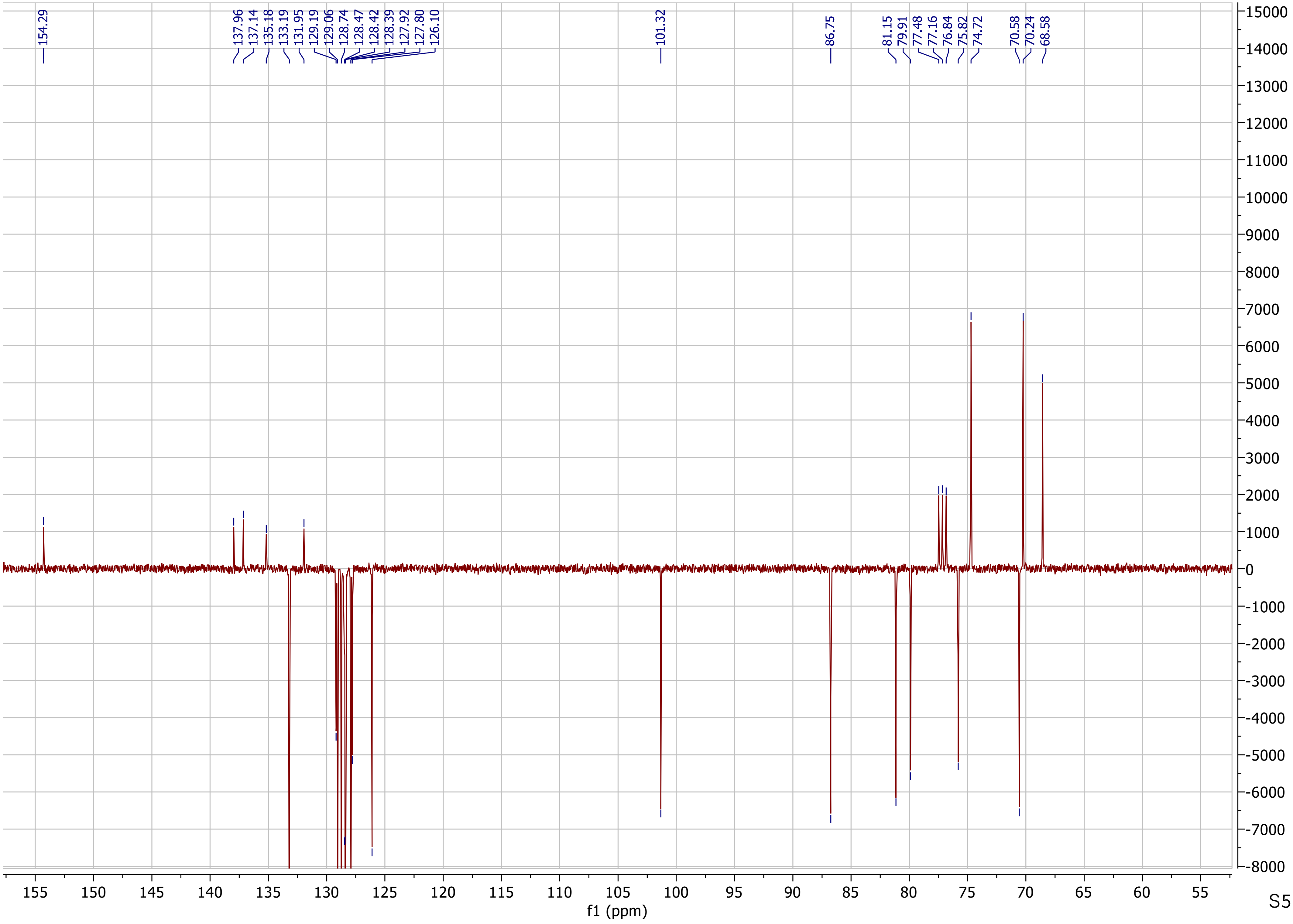

154.29
137.96
137.14
135.18
133.19
131.95
129.19
129.06
128.74
128.47
128.42
128.39
127.92
127.80
126.10
101.32
86.75
81.15
79.91
77.48
77.16
76.84
75.82
74.72
70.58
70.24
68.58
f1 (ppm)

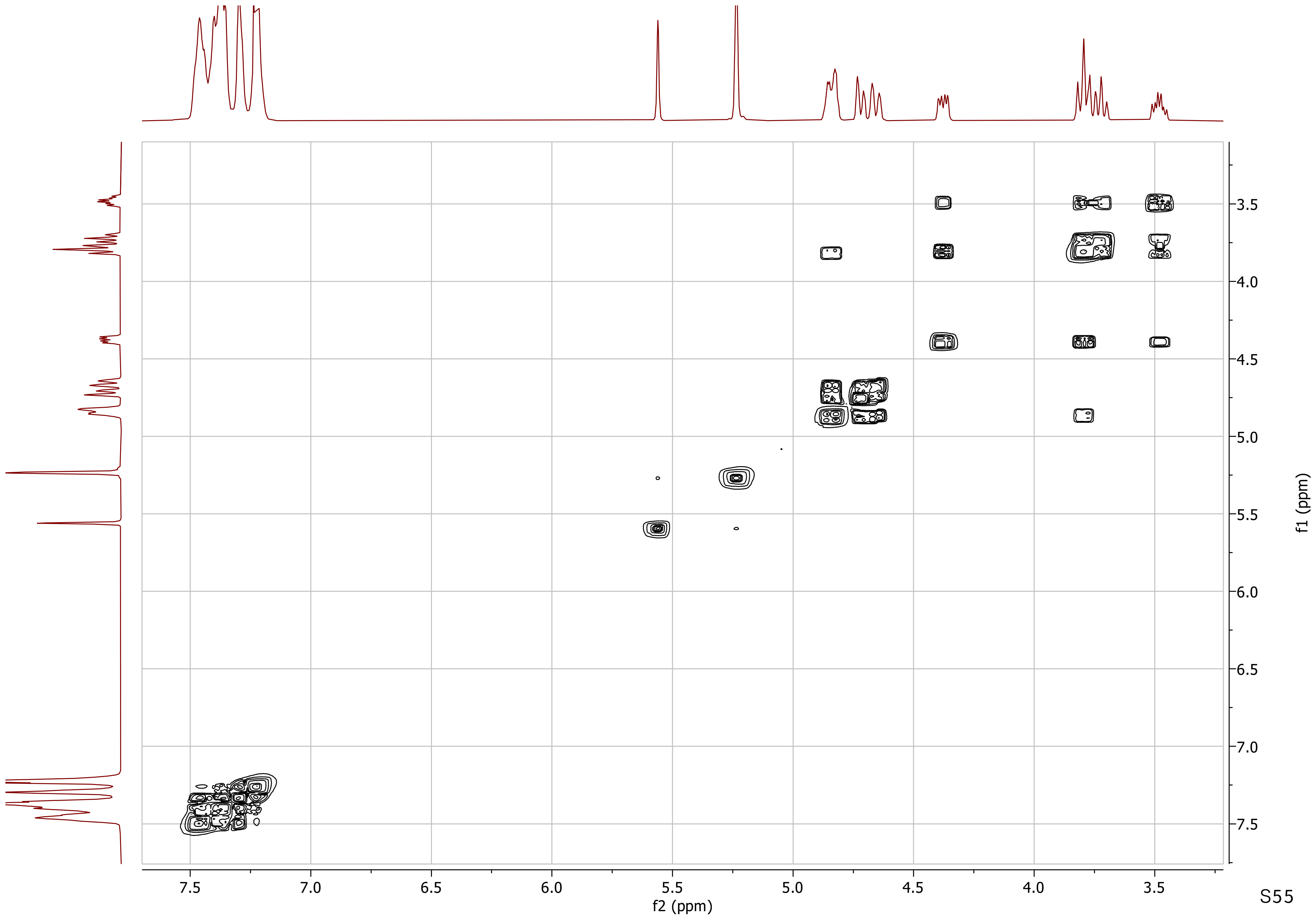

f2 (ppm)
f1 (ppm)
7.5
7.0
6.5
6.0
5.5
5.0
4.5
4.0
3.5

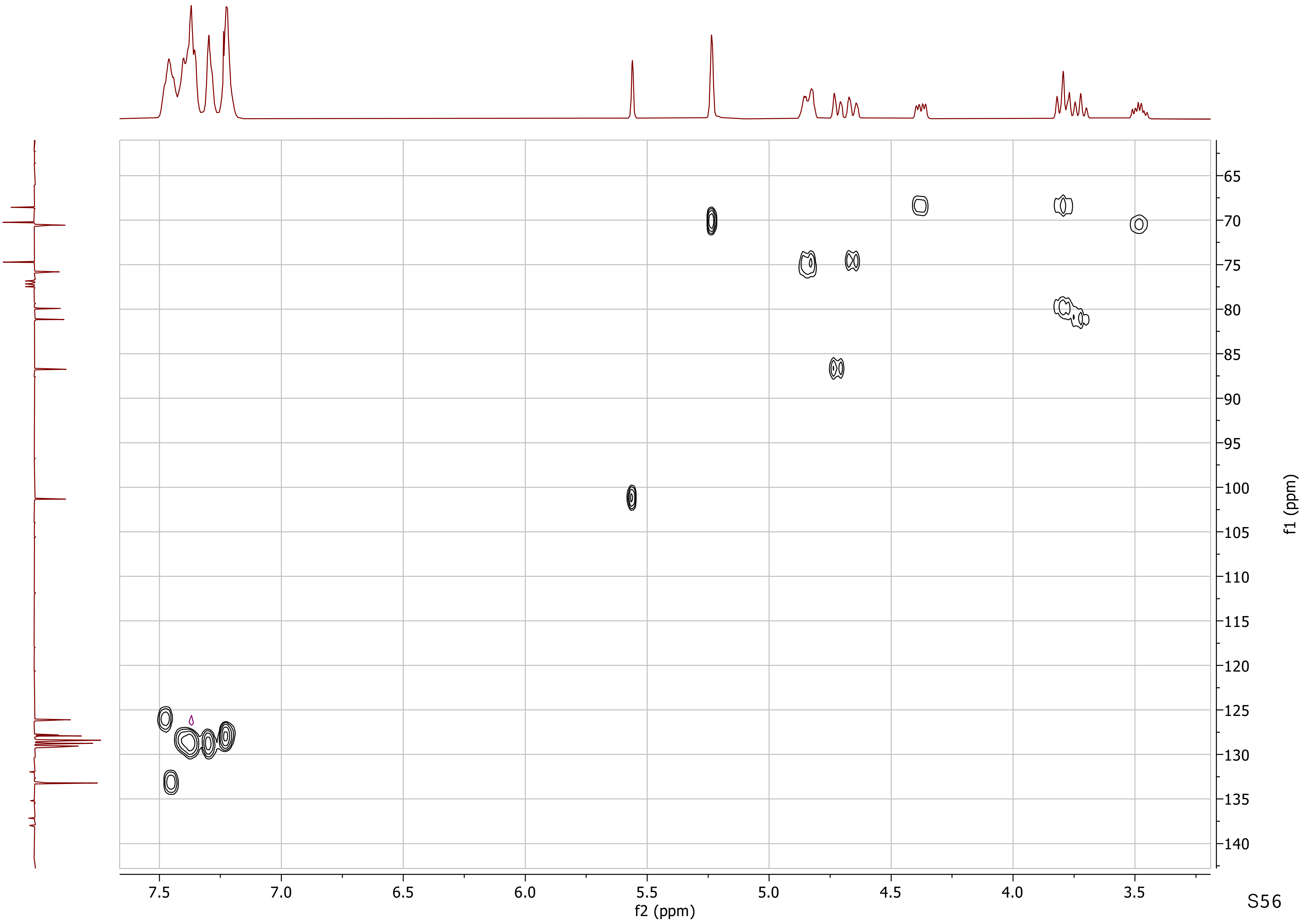

65
70
75
80
85
90
95
100
105
110
115
120
125
130
135
140
f1 (ppm)
7.5
7.0
6.5
6.0
5.5
5.0
4.5
4.0
3.5
f2 (ppm)

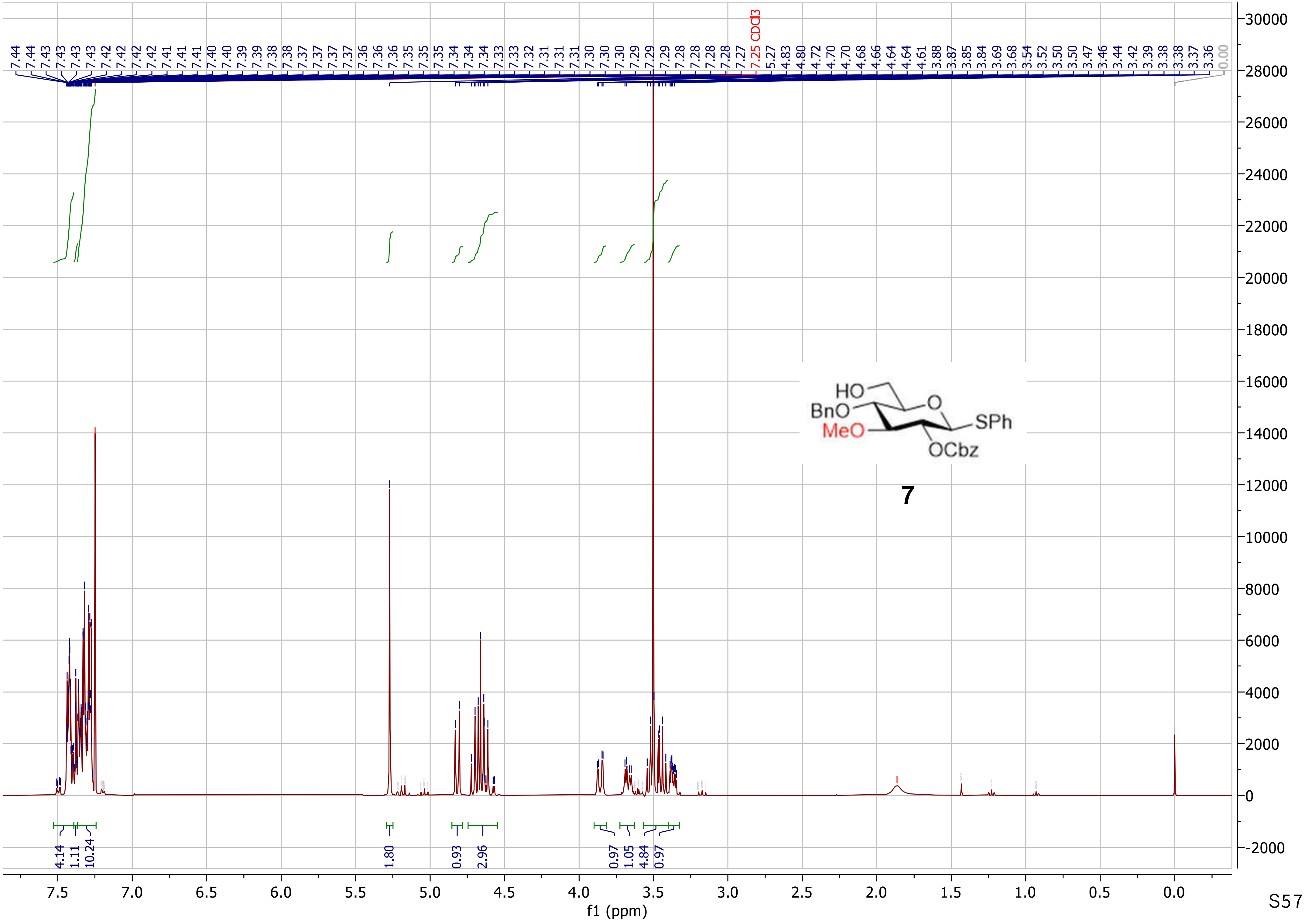

7.25 CDCl3
HO
BnO
MeO
O
SPh
OCbz
4.14
1.11
10.24
1.80
0.93
2.96
0.97
1.05
4.84
0.97
f1 (ppm)

7

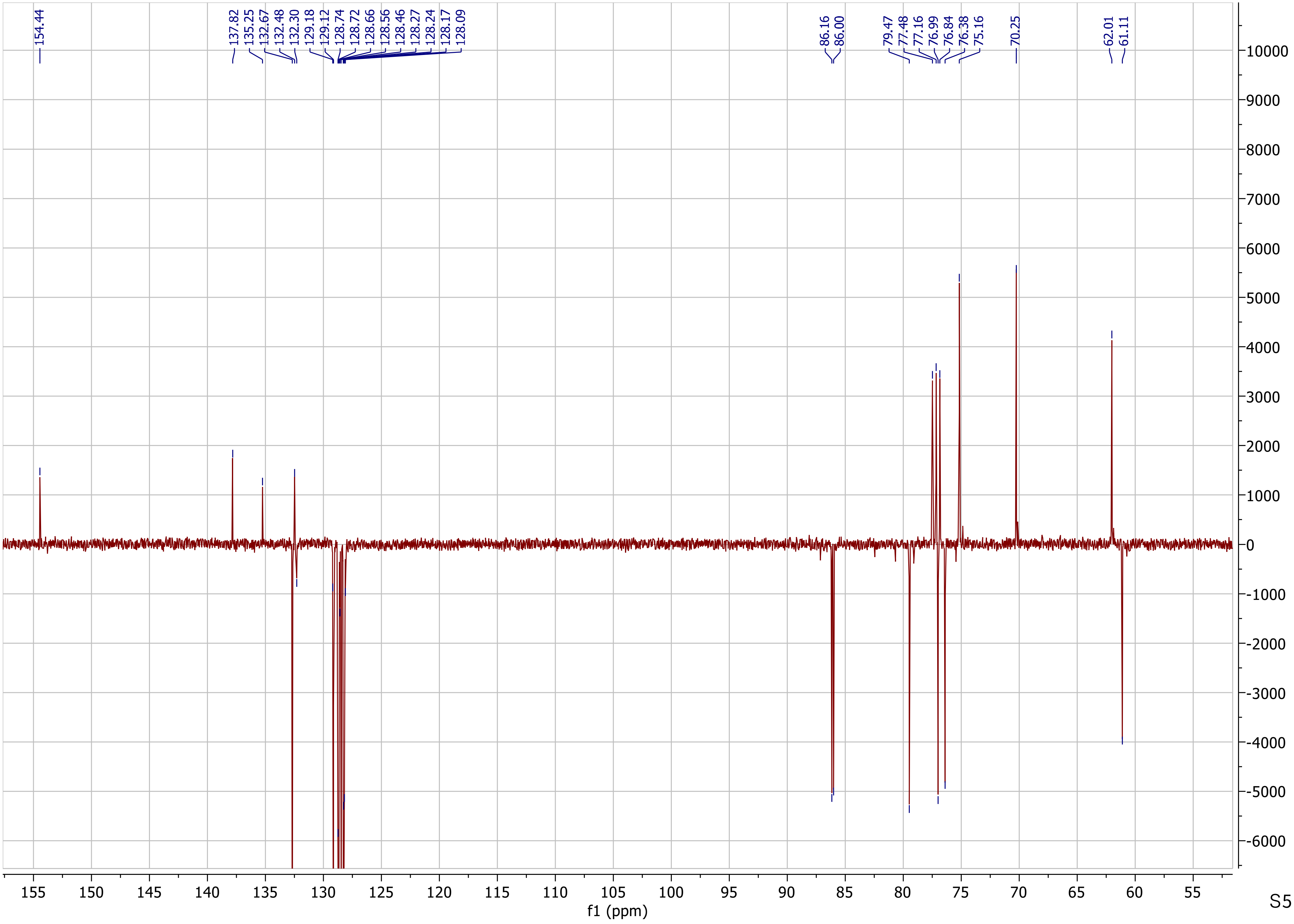
154.44
137.82
135.25
132.67
132.48
132.30
129.18
129.12
128.74
128.72
128.66
128.56
128.46
128.27
128.24
128.17
128.09
86.16
86.00
79.47
77.48
77.16
76.99
76.84
76.38
75.16
70.25
62.01
61.11
f1 (ppm)

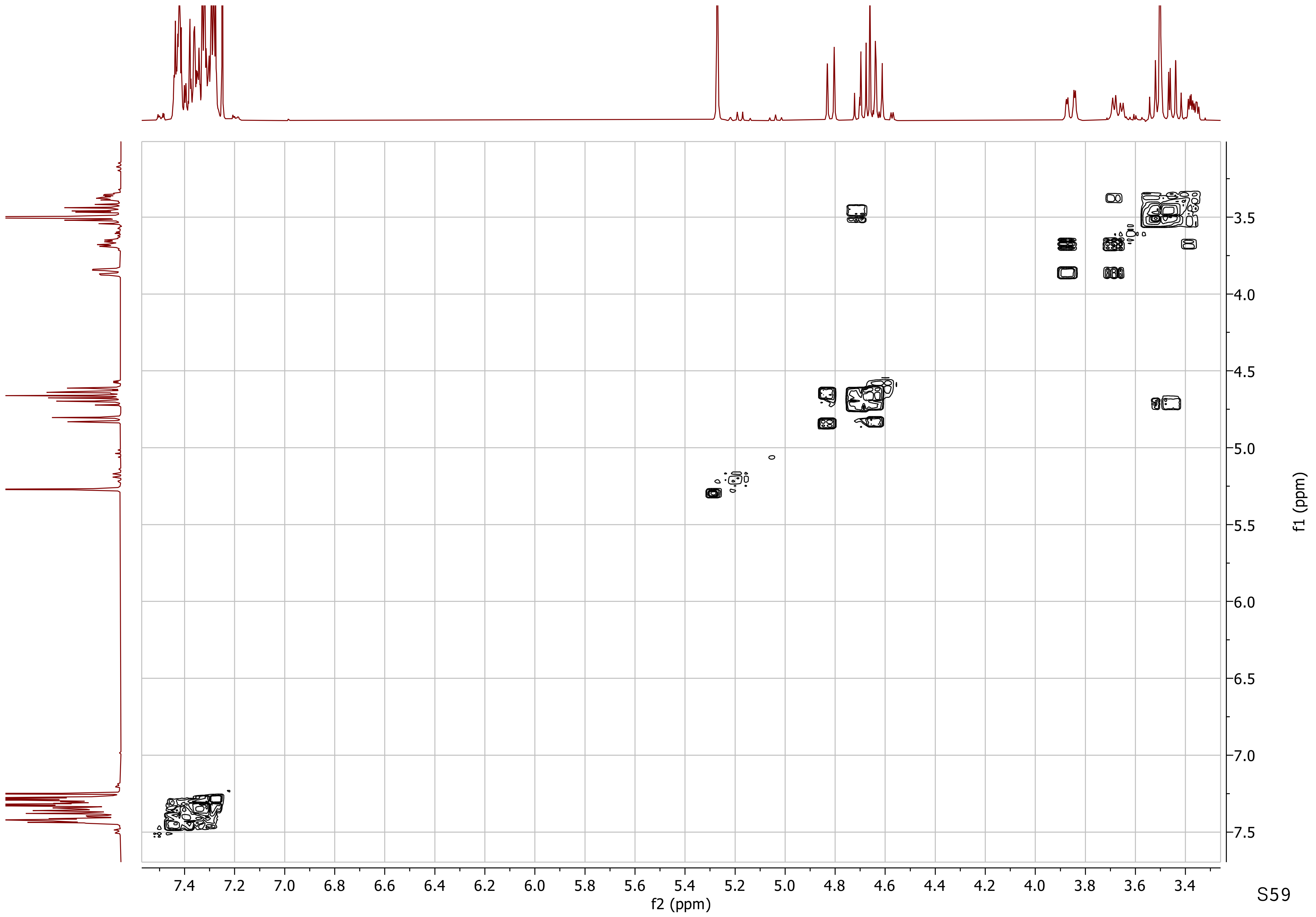

3.5
4.0
4.5
5.0
5.5
6.0
6.5
7.0
7.5
f1 (ppm)
7.4
7.2
7.0
6.8
6.6
6.4
6.2
6.0
5.8
5.6
5.4
5.2
5.0
4.8
4.6
4.4
4.2
4.0
3.8
3.6
3.4
f2 (ppm)

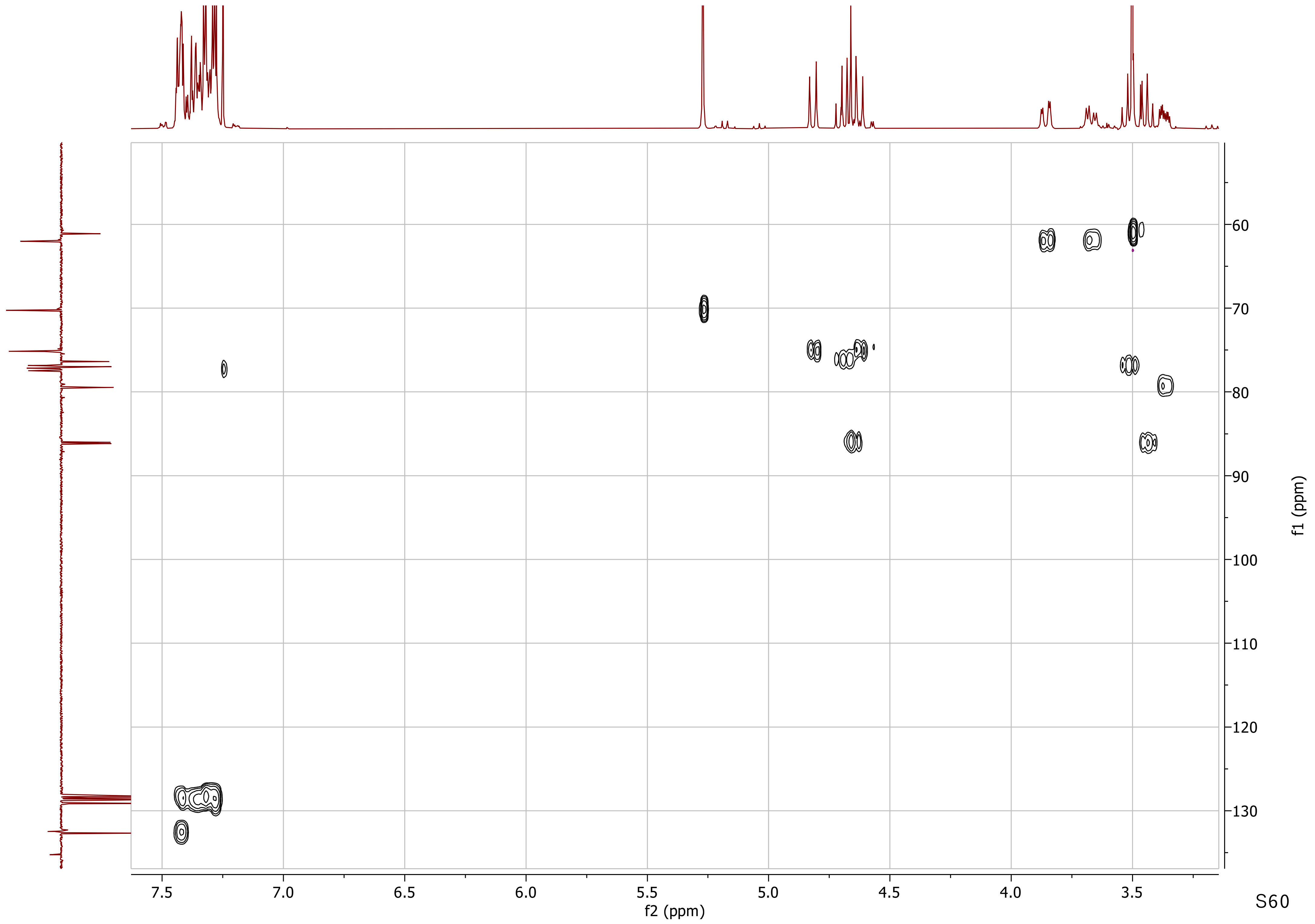
f1 (ppm)
60
70
80
90
100
110
120
130
7.5
7.0
6.5
6.0
5.5
5.0
4.5
4.0
3.5
f2 (ppm)

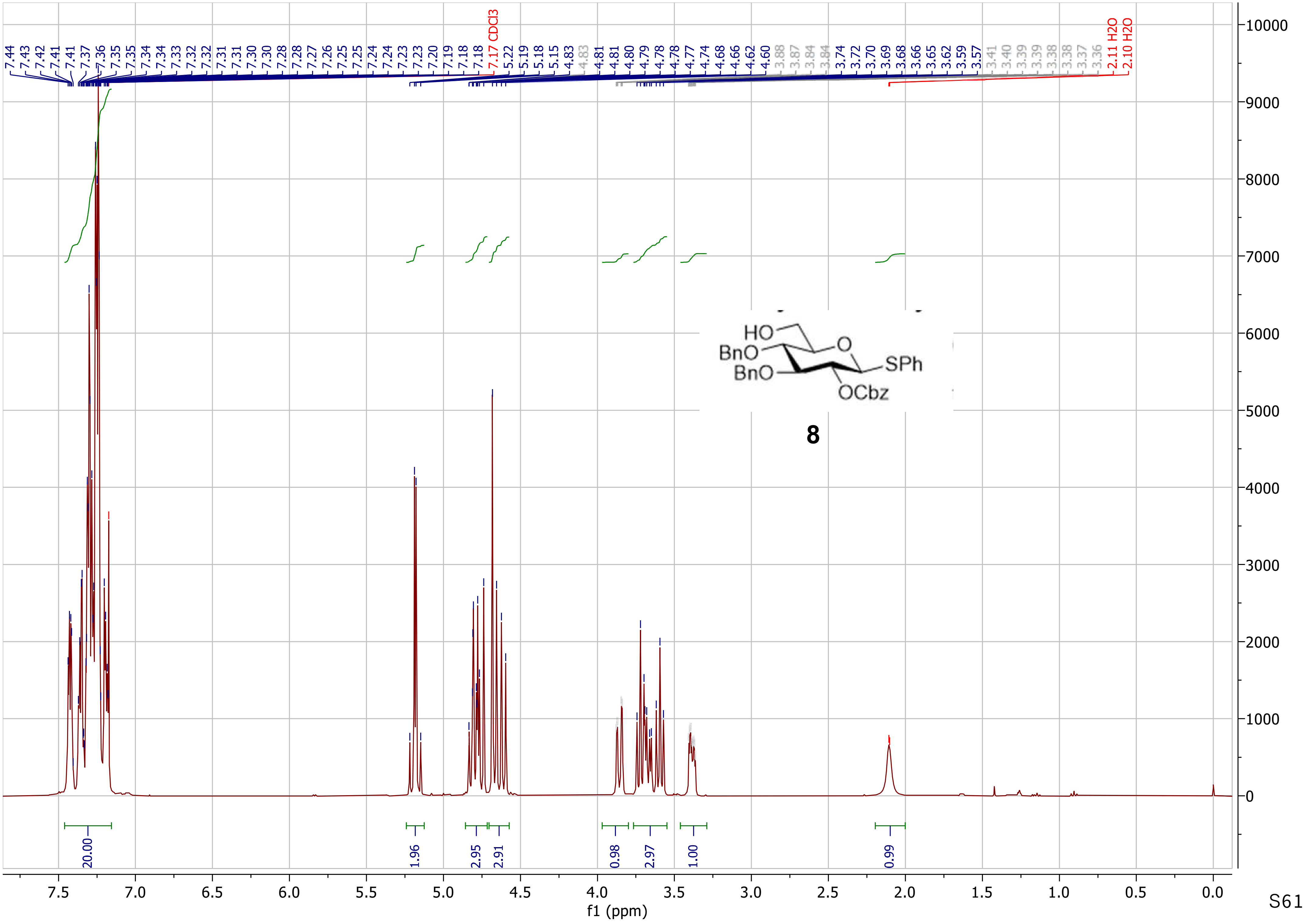
7.44 7.43 7.42 7.41 7.41 7.37 7.36 7.35 7.35 7.34 7.34 7.33 7.32 7.32 7.31 7.31 7.30 7.30 7.28 7.28 7.27 7.26 7.25 7.25 7.24 7.24 7.23 7.23 7.20 7.19 7.18 7.18
7.17 CDCl3
5.22 5.19 5.18 5.15 4.83 4.83 4.81 4.81 4.80 4.79 4.78 4.78 4.77 4.74 4.68 4.66 4.62 4.60 3.88 3.87 3.84 3.84 3.74 3.72 3.70 3.69 3.68 3.66 3.65 3.62 3.59 3.57 3.41 3.40 3.39 3.39 3.38 3.38 3.37 3.36
2.11 H2O
2.10 H2O
HO
BnO
BnO
O
SPh
OCbz
8
20.00
1.96
2.95
2.91
0.98
2.97
1.00
0.99
f1 (ppm)

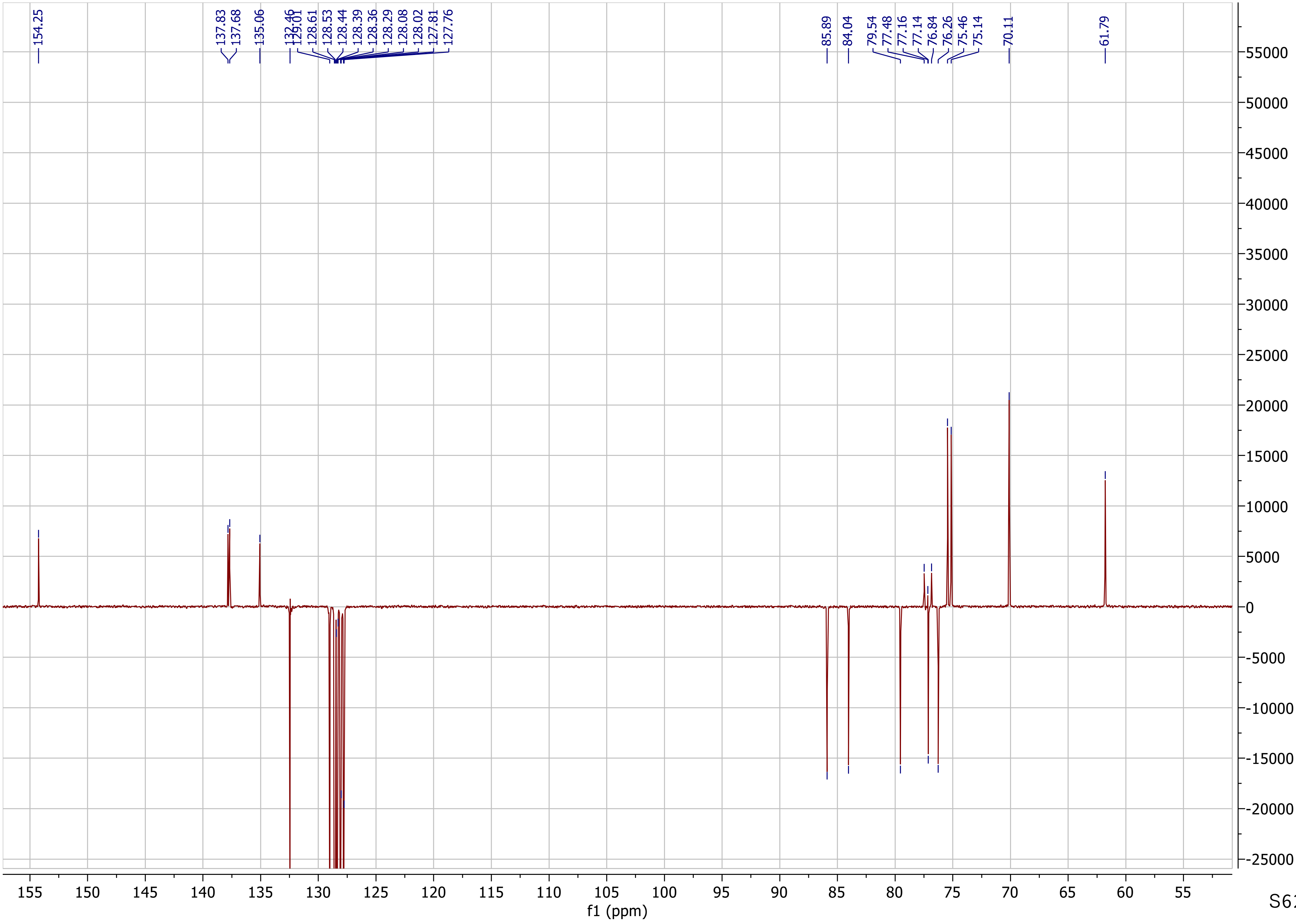

154.25
137.83
137.68
135.06
132.46
129.01
128.61
128.53
128.44
128.39
128.36
128.29
128.08
128.02
127.81
127.76
85.89
84.04
79.54
77.48
77.16
77.14
76.84
76.26
75.46
75.14
70.11
61.79
f1 (ppm)

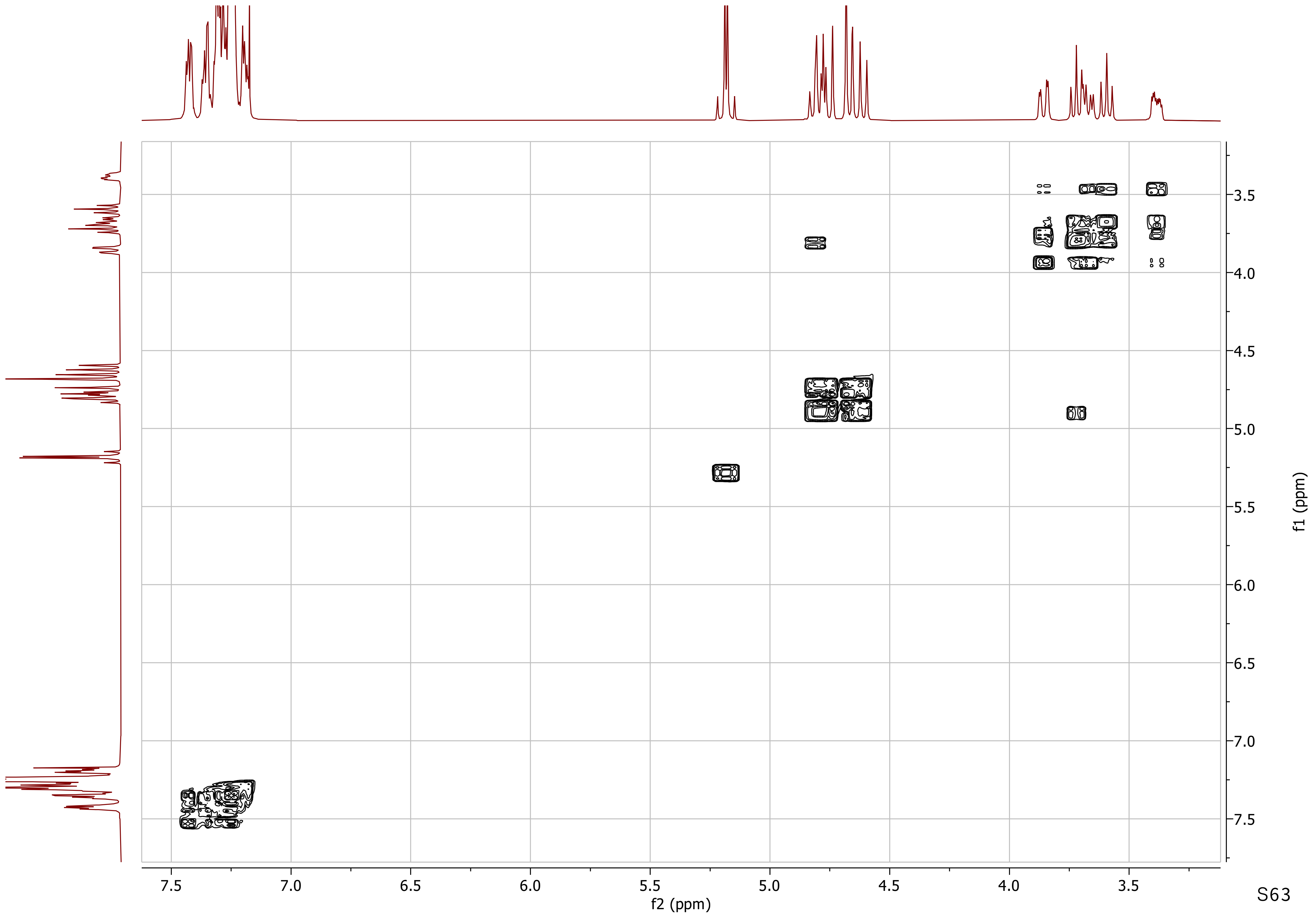
7.5
7.0
6.5
6.0
5.5
5.0
4.5
4.0
3.5
f2 (ppm)
3.5
4.0
4.5
5.0
5.5
6.0
6.5
7.0
7.5
f1 (ppm)

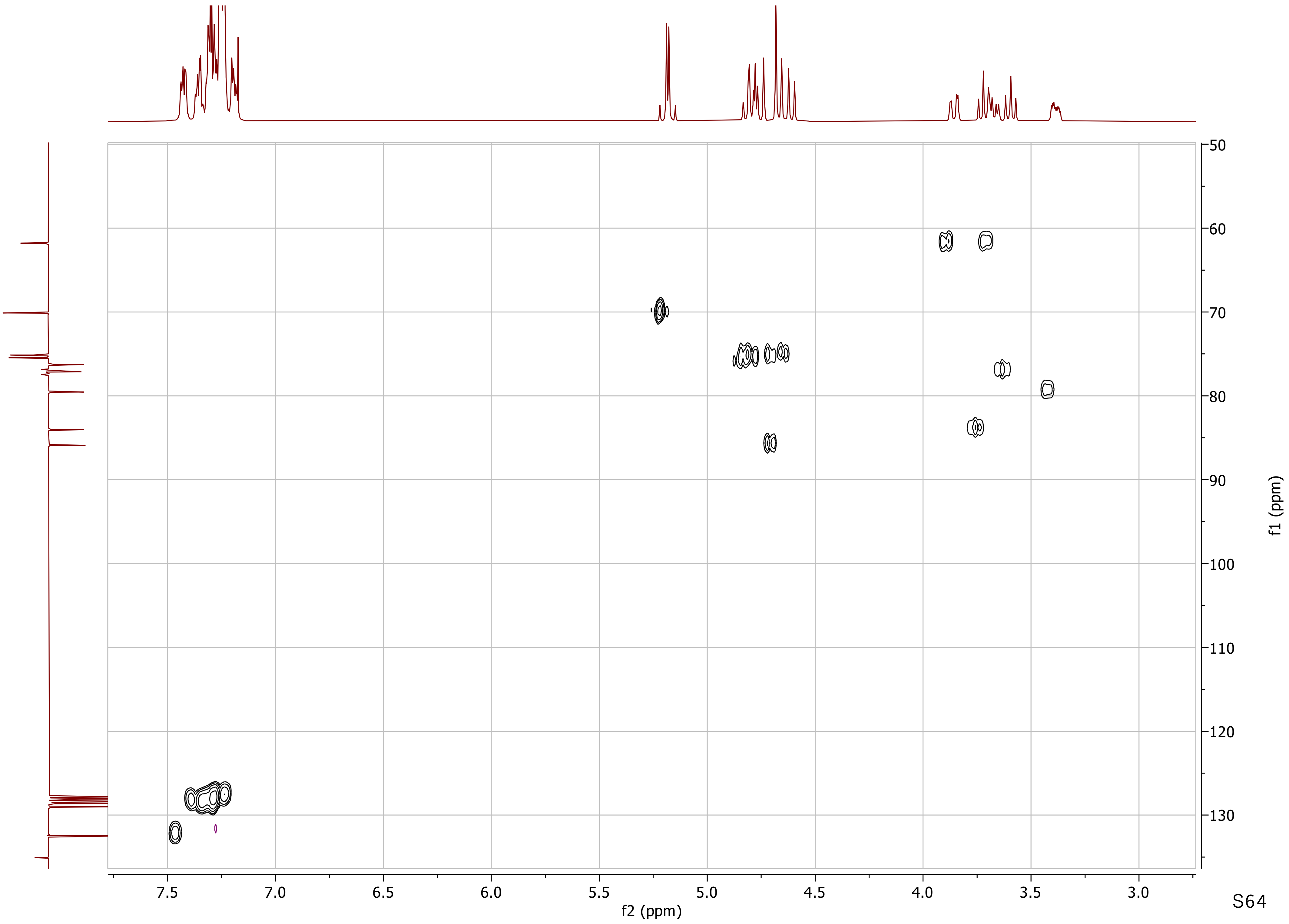

7.5
7.0
6.5
6.0
5.5
5.0
4.5
4.0
3.5
3.0
f2 (ppm)
50
60
70
80
90
100
110
120
130
f1 (ppm)

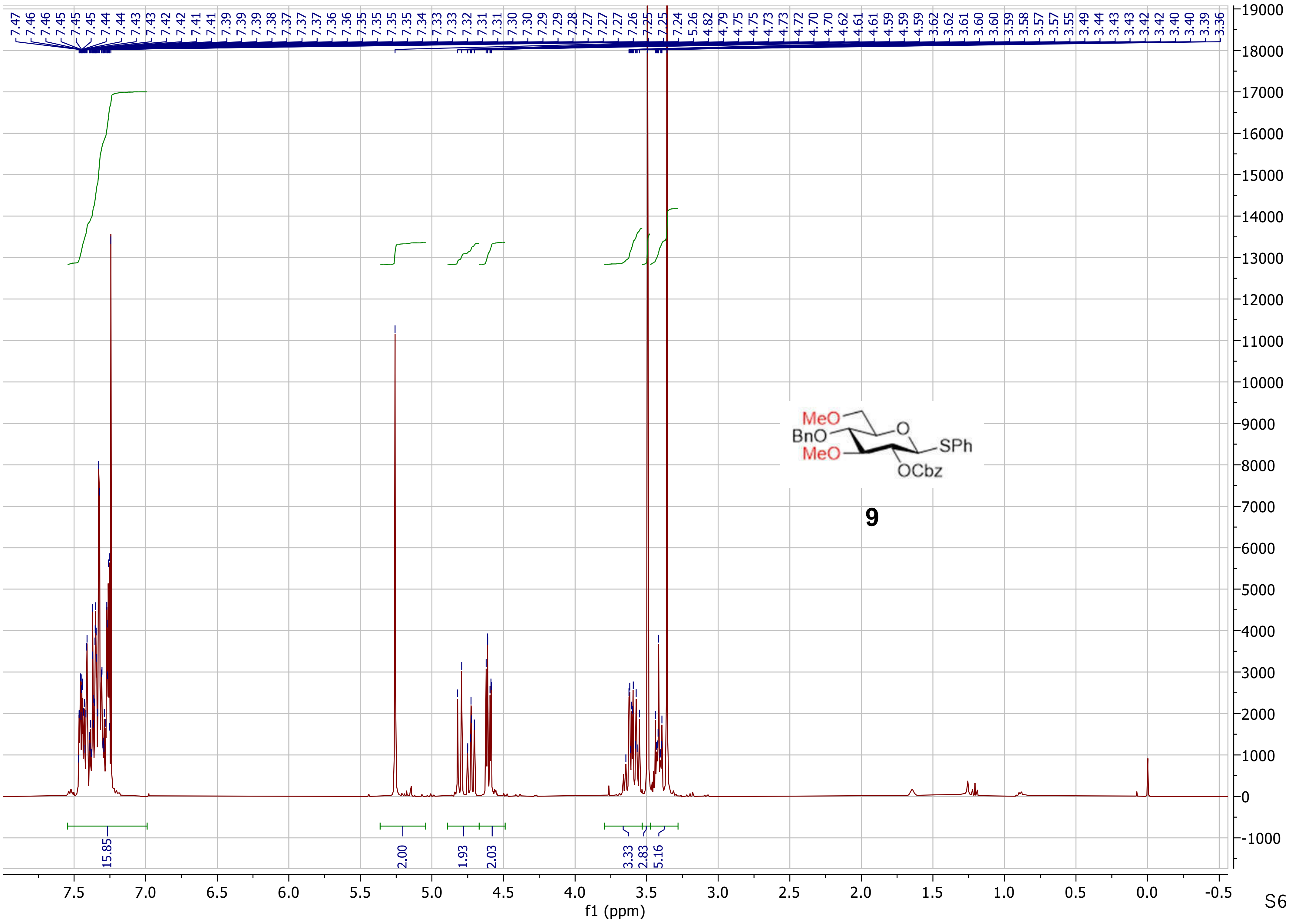
7.47 7.46 7.46 7.45 7.45 7.45 7.44 7.44 7.43 7.43 7.42 7.42 7.41 7.41 7.39 7.39 7.39 7.38 7.37 7.37 7.36 7.36 7.35 7.35 7.35 7.35 7.34 7.33 7.33 7.32 7.31 7.31 7.30 7.30 7.29 7.29 7.28 7.27 7.27 7.27 7.26 7.25 7.25 7.24 5.26 4.82 4.79 4.75 4.75 4.73 4.73 4.72 4.70 4.70 4.62 4.61 4.61 4.59 4.59 4.59 3.62 3.61 3.60 3.60 3.59 3.58 3.57 3.55 3.49 3.44 3.43 3.43 3.42 3.42 3.40 3.40 3.39 3.36
MeO
BnO
MeO
O
SPh
OCbz
9
15.85
2.00
1.93
2.03
3.33
2.83
5.16
f1 (ppm)

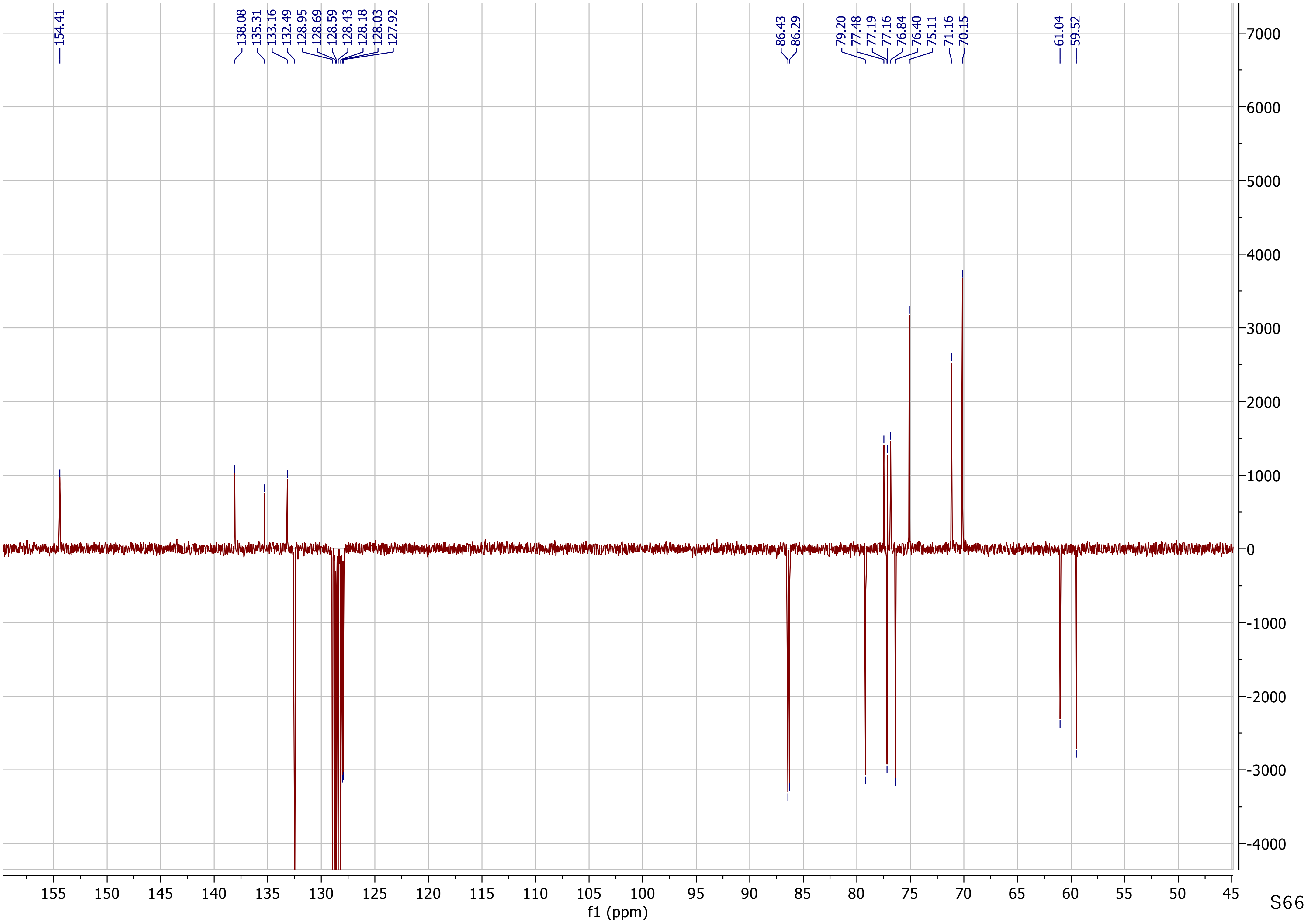
154.41
138.08
135.31
133.16
132.49
128.95
128.69
128.59
128.43
128.18
128.03
127.92
86.43
86.29
79.20
77.48
77.19
77.16
76.84
76.40
75.11
71.16
70.15
61.04
59.52
f1 (ppm)

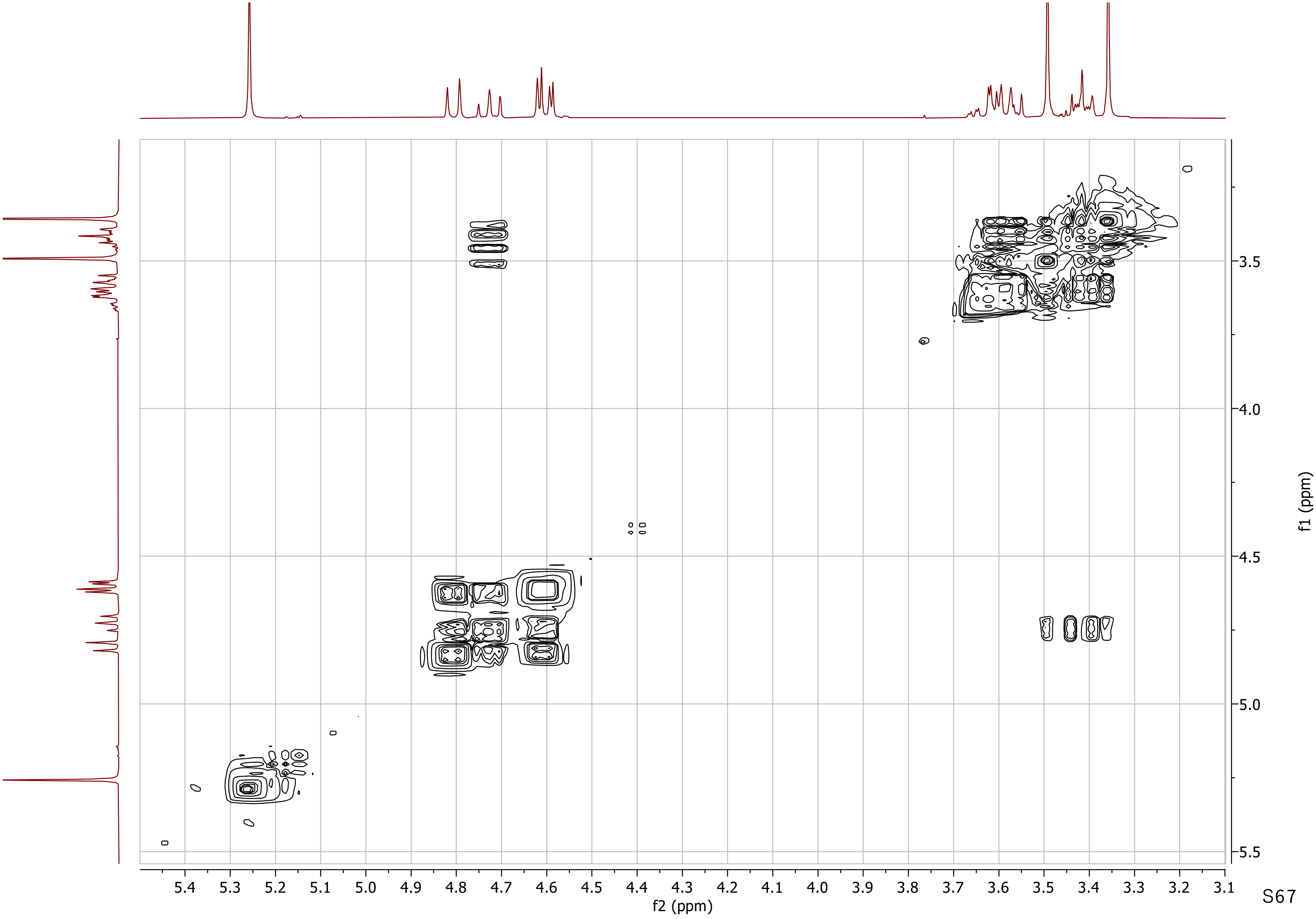

5.4 5.3 5.2 5.1 5.0 4.9 4.8 4.7 4.6 4.5 4.4 4.3 4.2 4.1 4.0 3.9 3.8 3.7 3.6 3.5 3.4 3.3 3.2 3.1
f2 (ppm)
3.5 4.0 4.5 5.0 5.5
f1 (ppm)

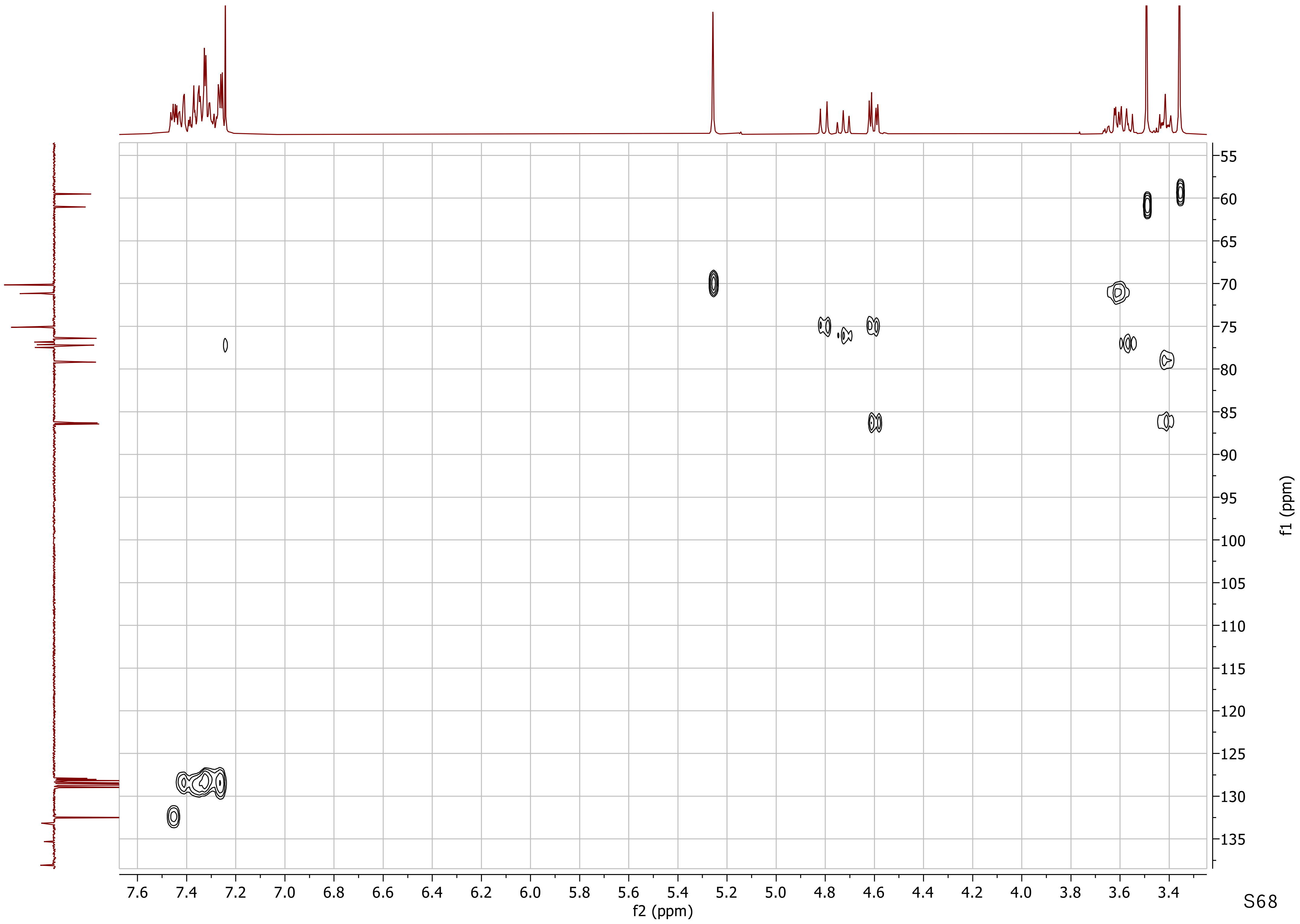
7.6
7.4
7.2
7.0
6.8
6.6
6.4
6.2
6.0
5.8
5.6
5.4
5.2
5.0
4.8
4.6
4.4
4.2
4.0
3.8
3.6
3.4
f2 (ppm)
55
60
65
70
75
80
85
90
95
100
105
110
115
120
125
130
135
f1 (ppm)

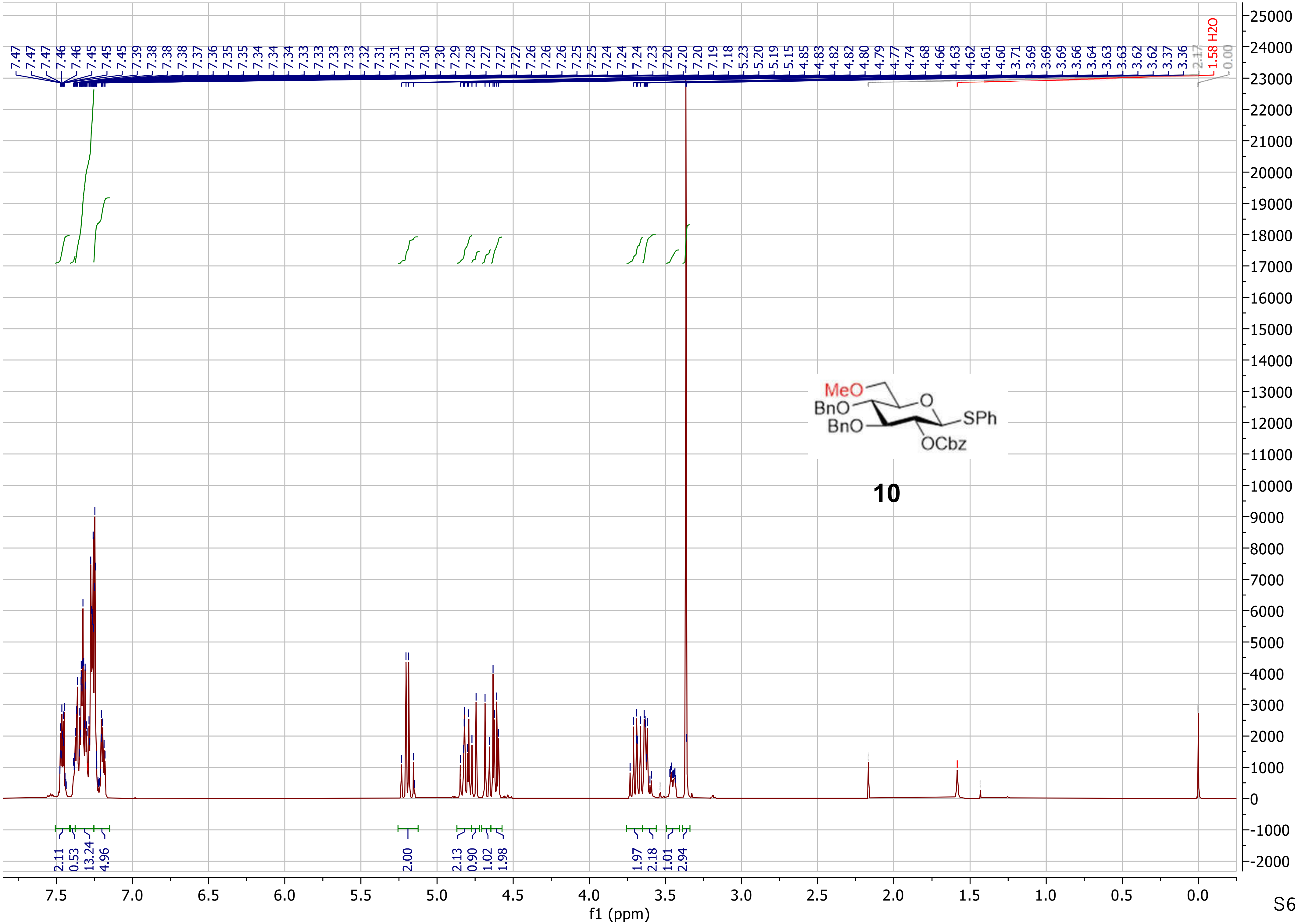

10

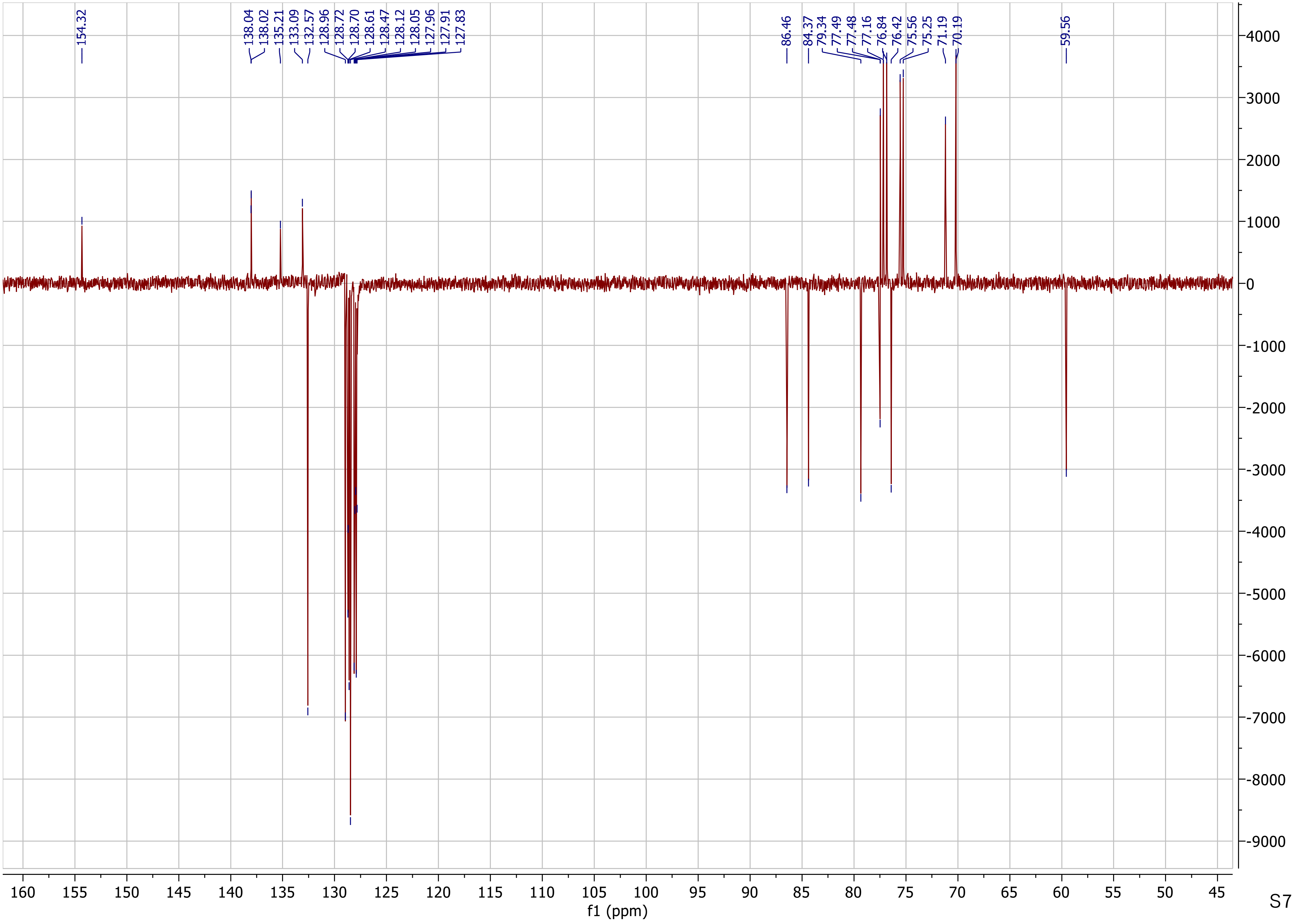

154.32
138.04
138.02
135.21
133.09
132.57
128.96
128.72
128.70
128.61
128.47
128.12
128.05
127.96
127.91
127.83
86.46
84.37
79.34
77.49
77.48
77.16
76.84
76.42
75.56
75.25
71.19
70.19
59.56
f1 (ppm)

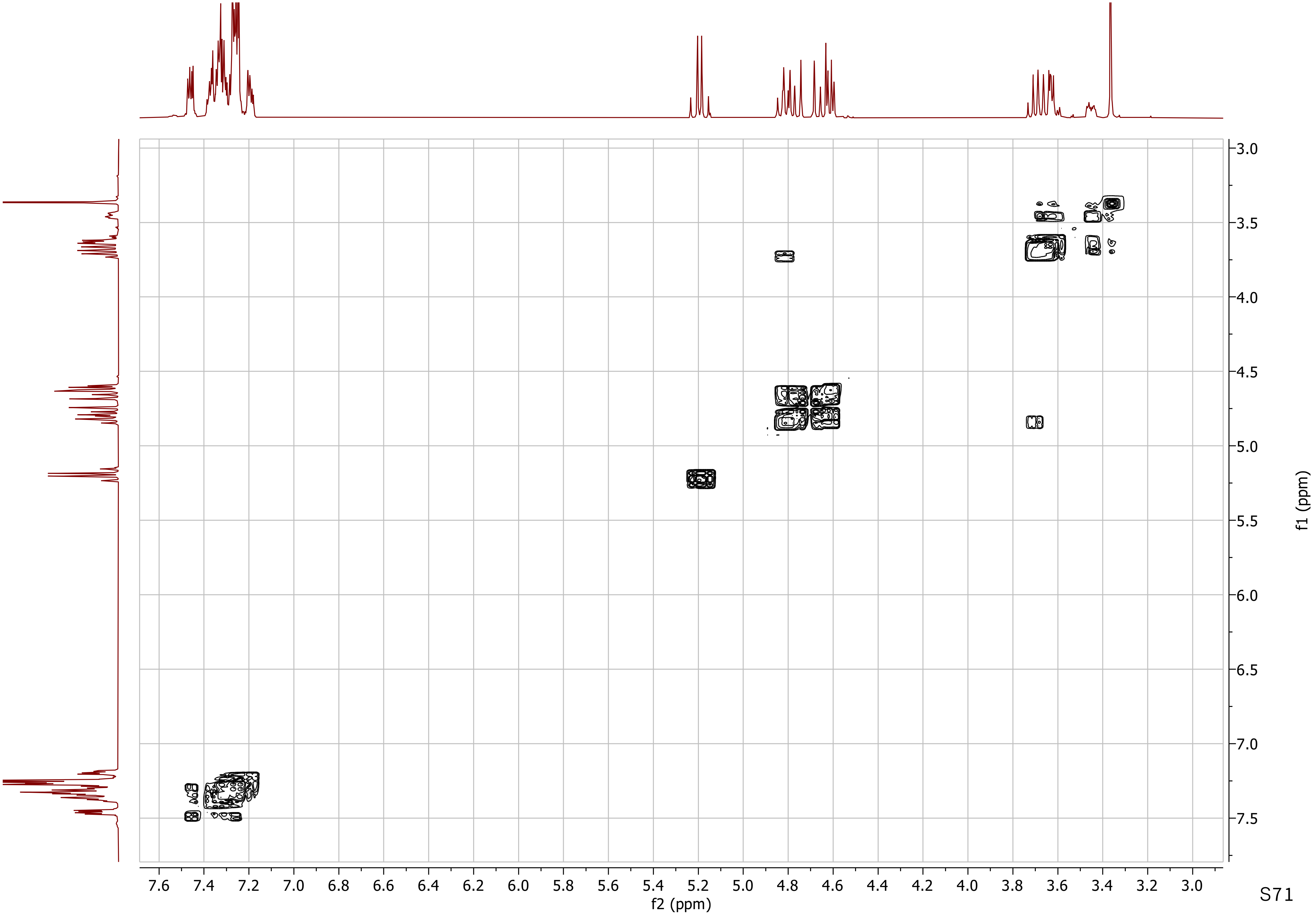

7.6
7.4
7.2
7.0
6.8
6.6
6.4
6.2
6.0
5.8
5.6
5.4
5.2
5.0
4.8
4.6
4.4
4.2
4.0
3.8
3.6
3.4
3.2
3.0
f2 (ppm)
3.0
3.5
4.0
4.5
5.0
5.5
6.0
6.5
7.0
7.5
f1 (ppm)

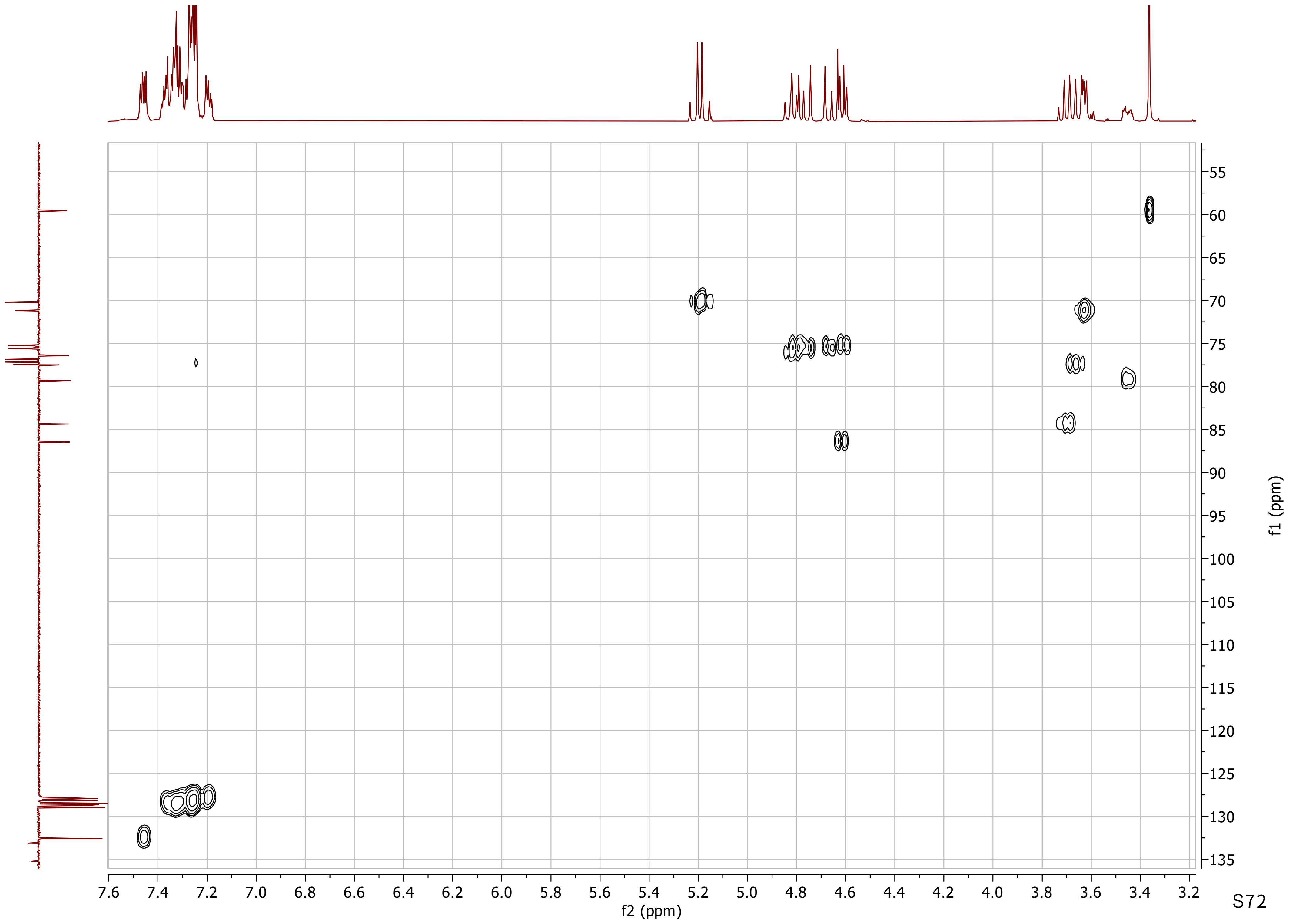

f2 (ppm)
f1 (ppm)
7.6
7.4
7.2
7.0
6.8
6.6
6.4
6.2
6.0
5.8
5.6
5.4
5.2
5.0
4.8
4.6
4.4
4.2
4.0
3.8
3.6
3.4
3.2
55
60
65
70
75
80
85
90
95
100
105
110
115
120
125
130
135

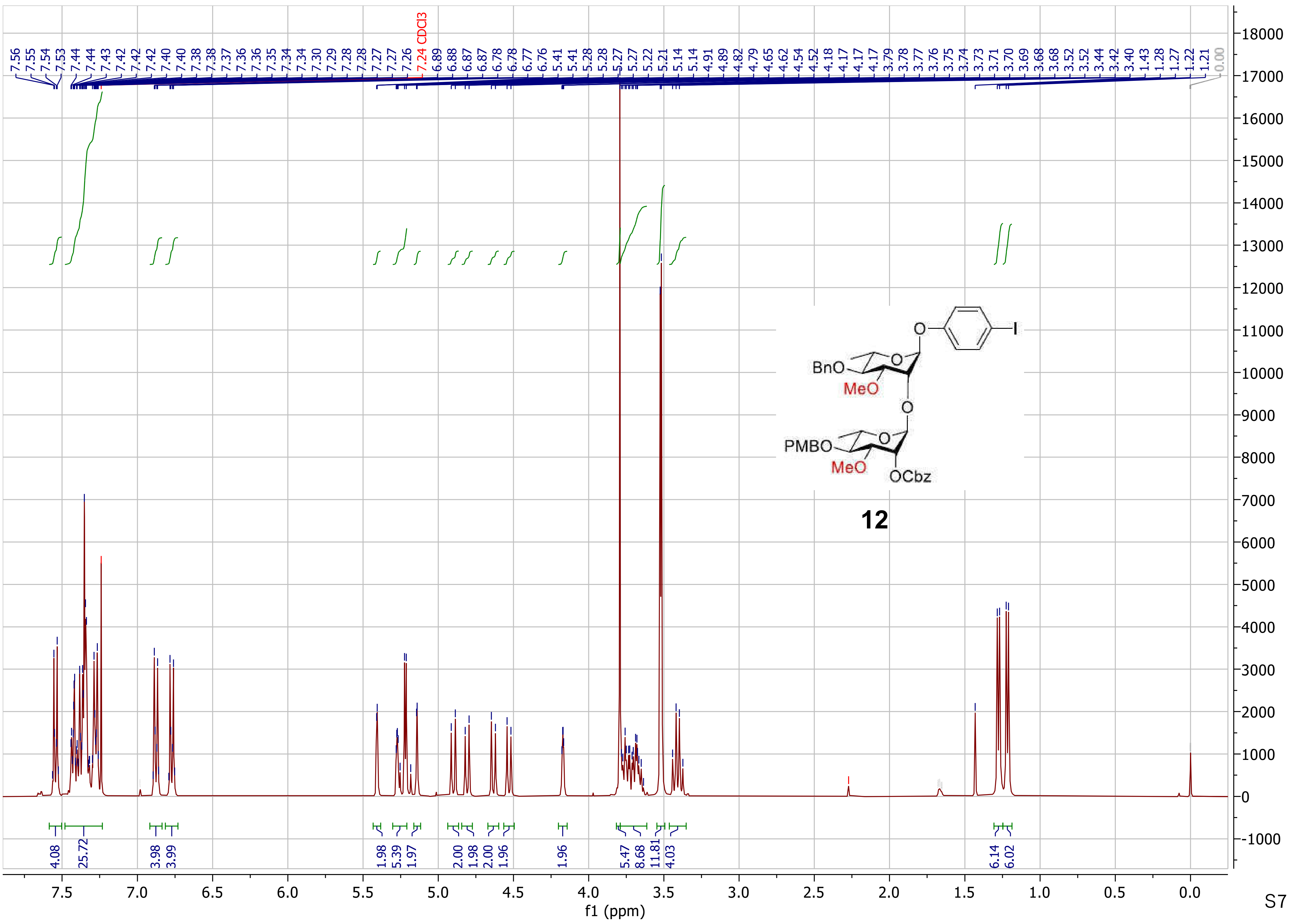

12

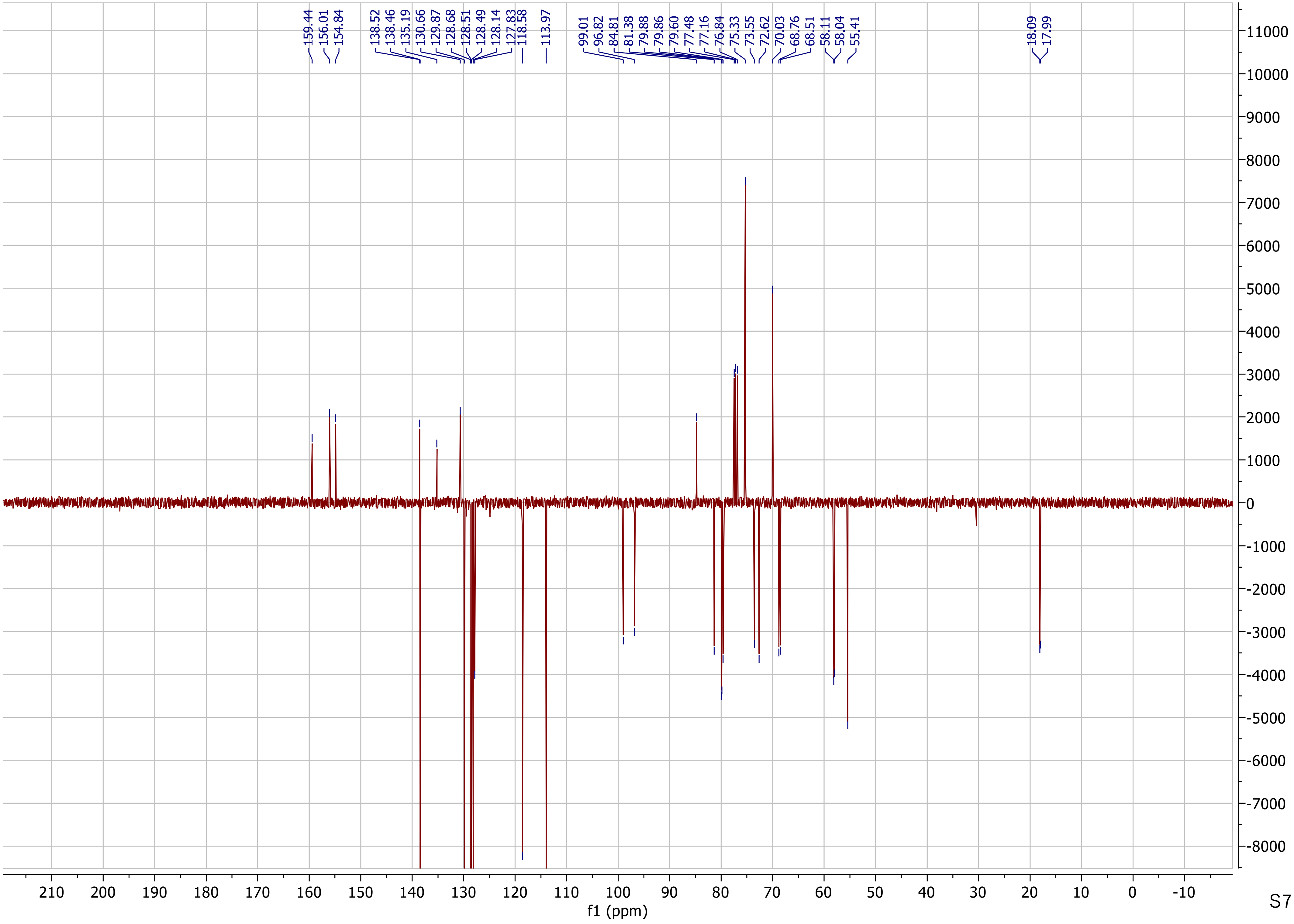
159.44
156.01
154.84
138.52
138.46
135.19
130.66
129.87
128.68
128.51
128.49
128.14
127.83
118.58
113.97
99.01
96.82
84.81
81.38
79.88
79.86
79.60
77.48
77.16
76.84
75.33
73.55
72.62
70.03
68.76
68.51
58.11
58.04
55.41
18.09
17.99
f1 (ppm)

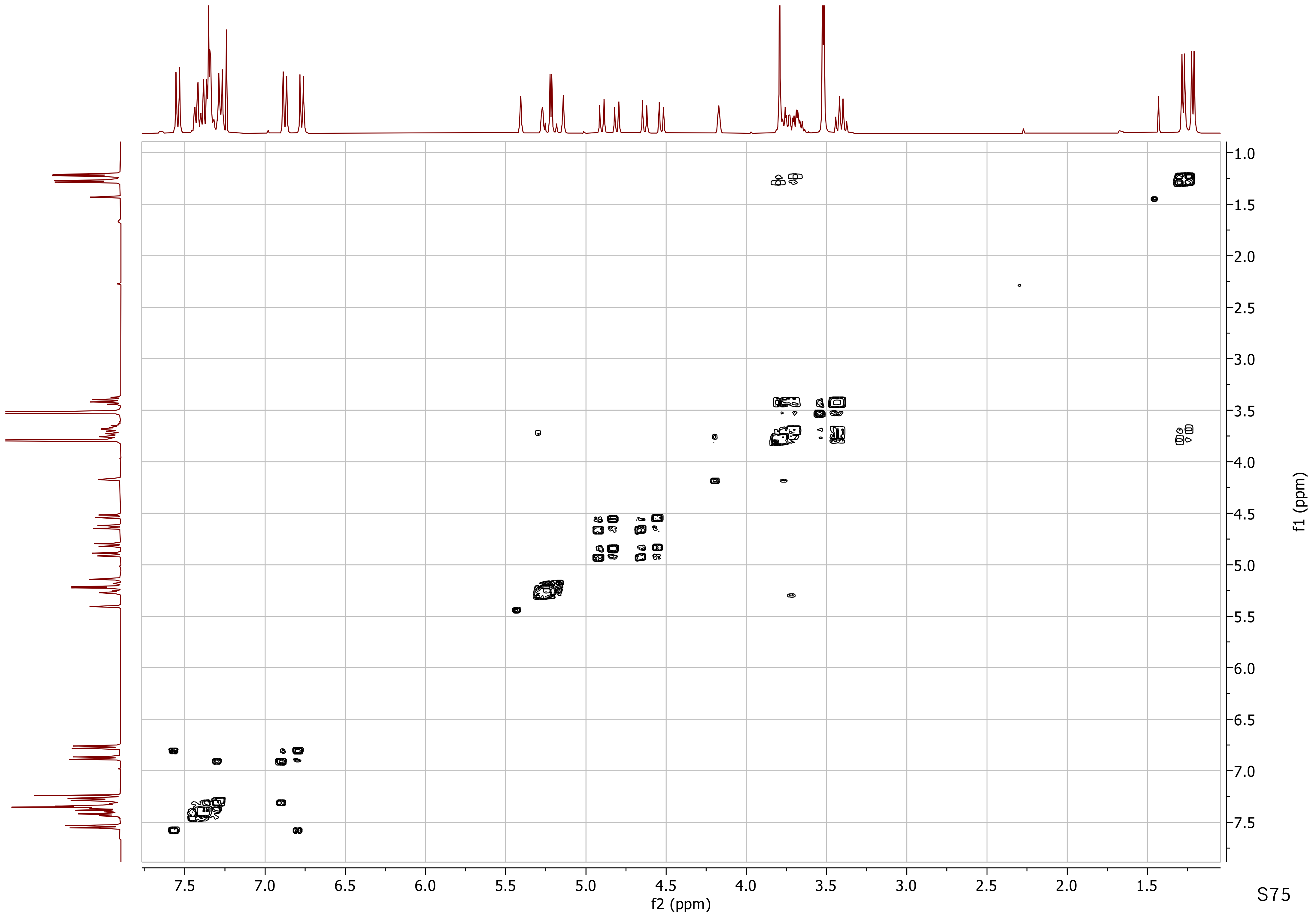

f2 (ppm)
f1 (ppm)

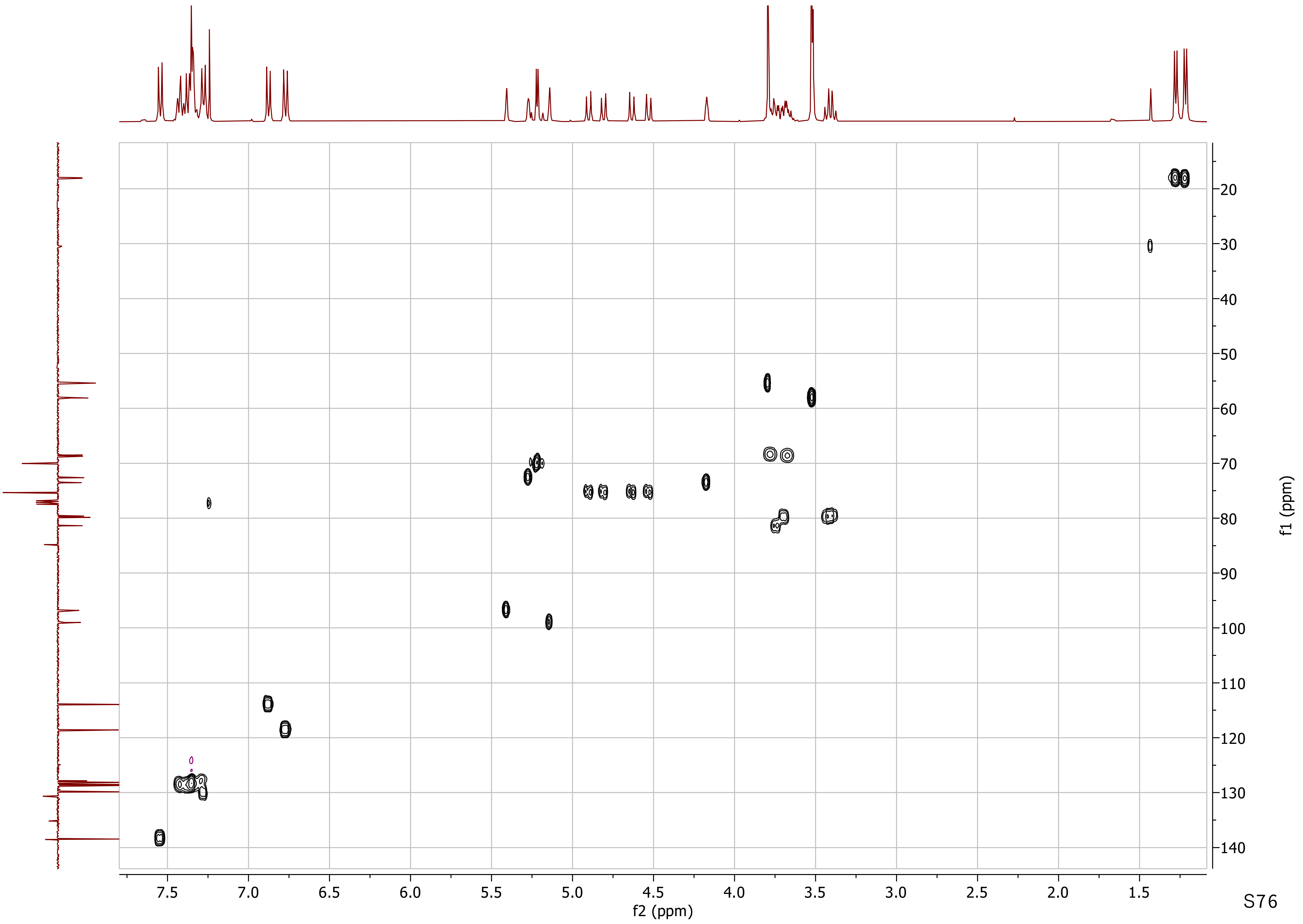
f2 (ppm)
f1 (ppm)

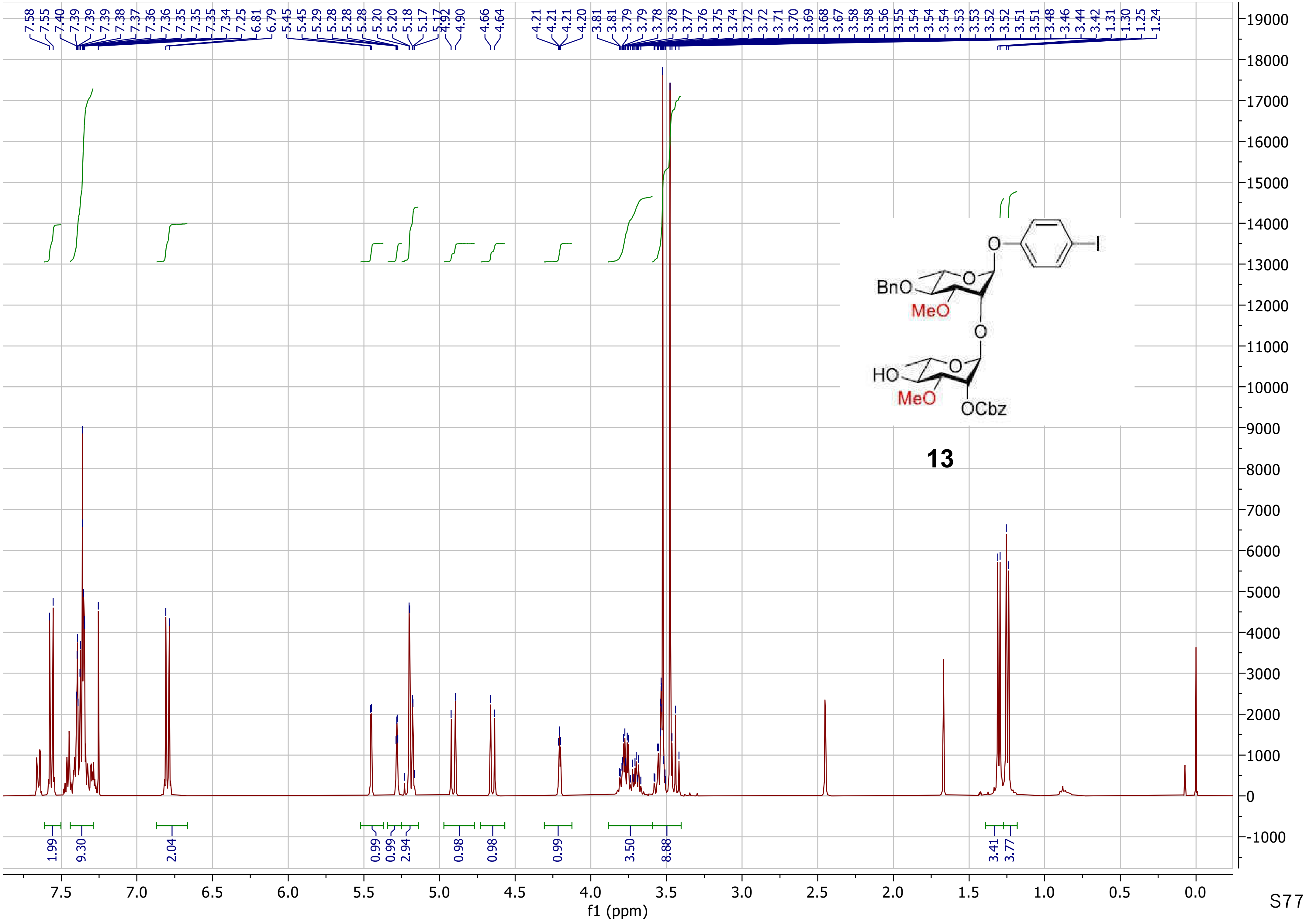
7.58 7.55 7.40 7.39 7.39 7.39 7.38 7.37 7.36 7.36 7.35 7.35 7.34 7.25 6.81 6.79 5.45 5.45 5.29 5.28 5.28 5.28 5.20 5.20 5.18 5.17 5.17 4.92 4.90 4.66 4.64 4.21 4.21 4.21 4.20 3.81 3.81 3.79 3.79 3.78 3.78 3.77 3.76 3.75 3.74 3.72 3.72 3.71 3.70 3.69 3.68 3.67 3.58 3.58 3.56 3.55 3.54 3.54 3.54 3.53 3.53 3.52 3.52 3.51 3.51 3.48 3.46 3.44 3.42 1.31 1.30 1.25 1.24
1.99 9.30 2.04 0.99 0.99 2.94 0.98 0.98 0.99 3.50 8.88 3.41 3.77
BnO
MeO
O
I
HO
MeO
OCbz
13
f1 (ppm)

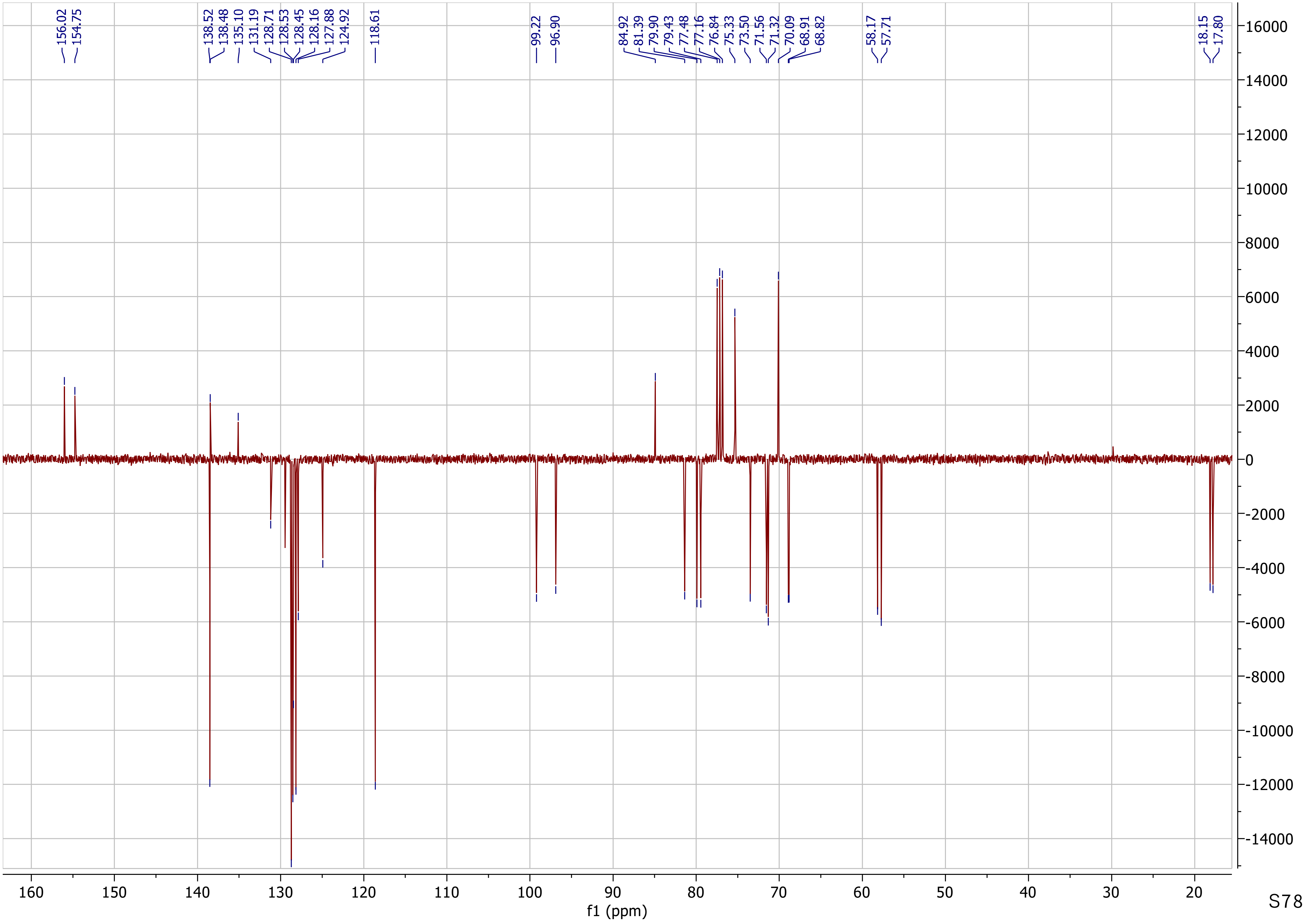
156.02
154.75
138.52
138.48
135.10
131.19
128.71
128.53
128.45
128.16
127.88
124.92
118.61
99.22
96.90
84.92
81.39
79.90
79.43
77.48
77.16
76.84
75.33
73.50
71.56
71.32
70.09
68.91
68.82
58.17
57.71
18.15
17.80
f1 (ppm)

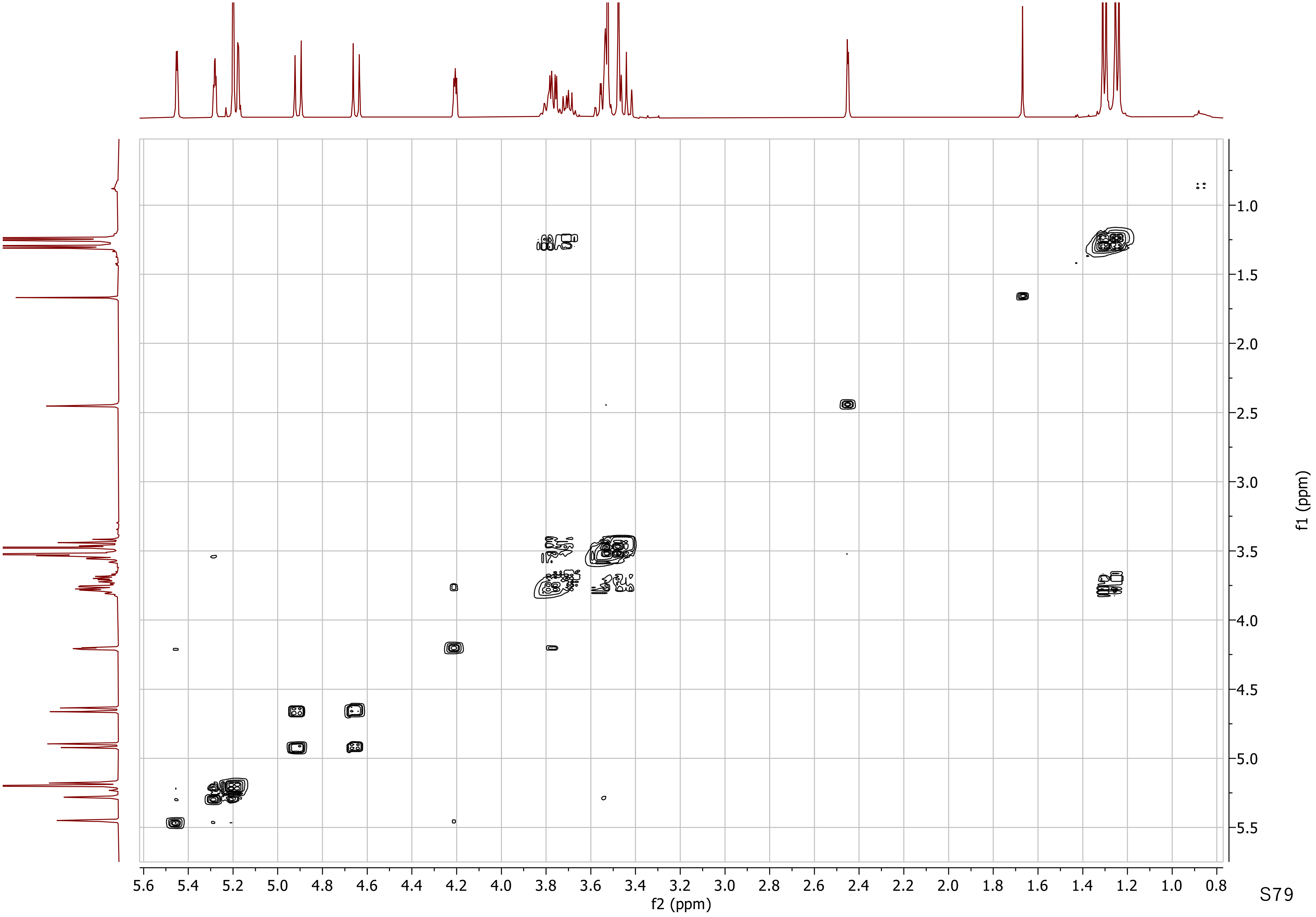

f2 (ppm)
f1 (ppm)
5.6 5.4 5.2 5.0 4.8 4.6 4.4 4.2 4.0 3.8 3.6 3.4 3.2 3.0 2.8 2.6 2.4 2.2 2.0 1.8 1.6 1.4 1.2 1.0 0.8
1.0 1.5 2.0 2.5 3.0 3.5 4.0 4.5 5.0 5.5

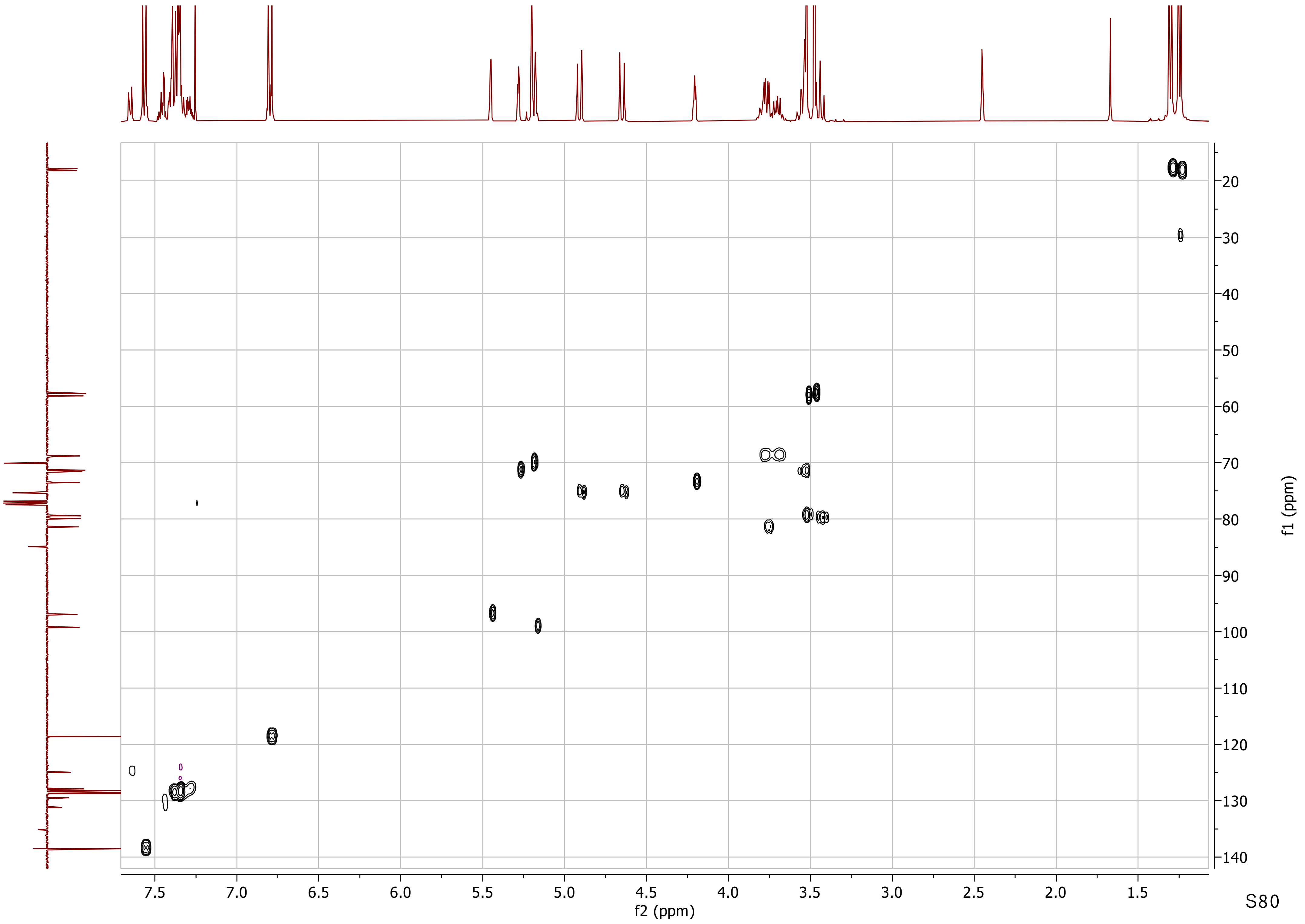
7.5
7.0
6.5
6.0
5.5
5.0
4.5
4.0
3.5
3.0
2.5
2.0
1.5
f2 (ppm)
20
30
40
50
60
70
80
90
100
110
120
130
140
f1 (ppm)

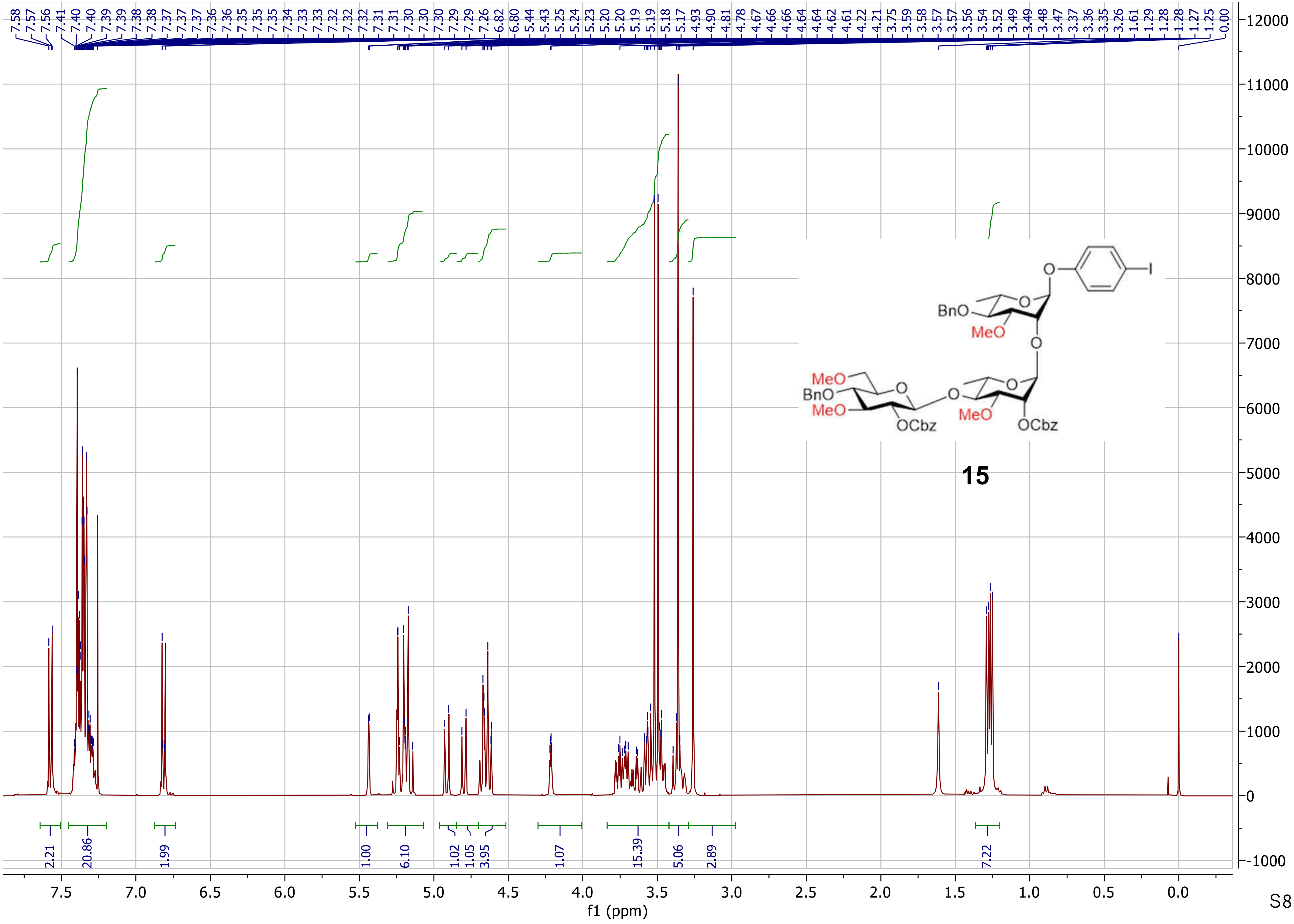
BnO
MeO
O
MeO
BnO
MeO
OCbz
MeO
OCbz
I
15
2.21
20.86
1.99
1.00
6.10
1.02
1.05
3.95
1.07
15.39
5.06
2.89
7.22
f1 (ppm)

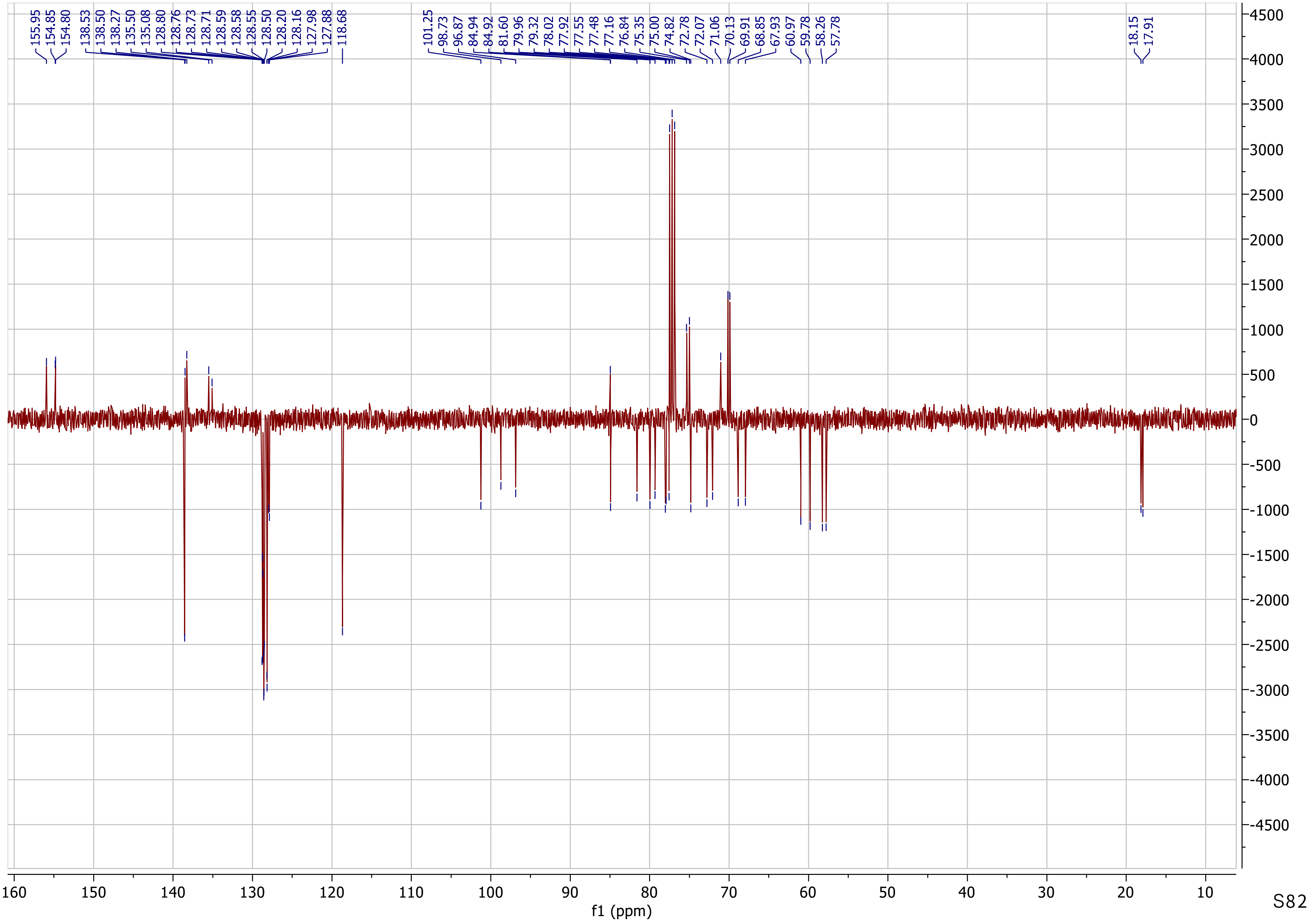
155.95
154.85
154.80
138.53
138.50
138.27
135.50
135.08
128.80
128.76
128.73
128.71
128.59
128.58
128.55
128.50
128.20
128.16
127.98
127.88
118.68
101.25
98.73
96.87
84.94
84.92
81.60
79.96
79.32
78.02
77.92
77.55
77.48
77.16
76.84
75.35
75.00
74.82
72.78
72.07
71.06
70.13
69.91
68.85
67.93
60.97
59.78
58.26
57.78
18.15
17.91
f1 (ppm)

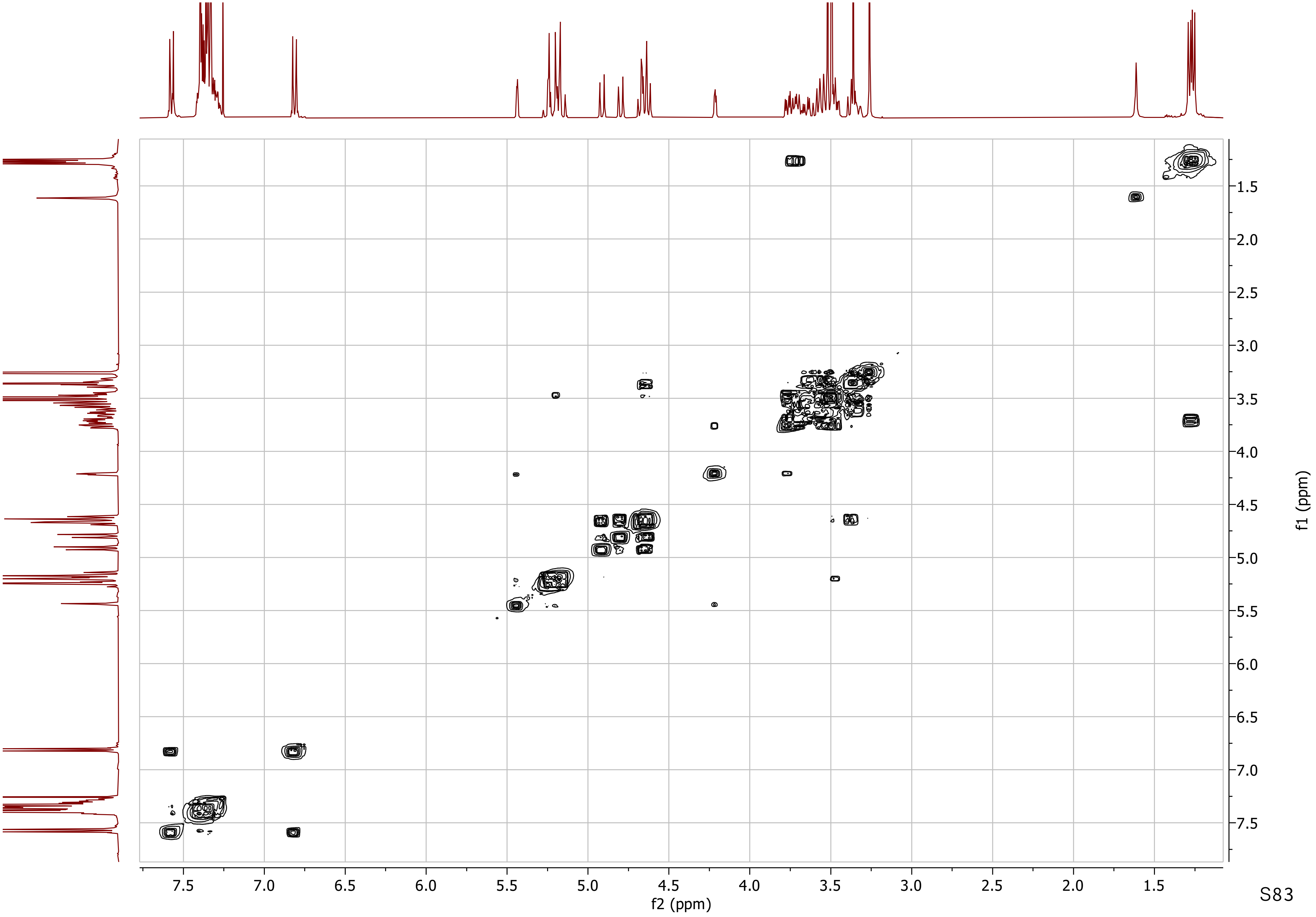

7.5
7.0
6.5
6.0
5.5
5.0
4.5
4.0
3.5
3.0
2.5
2.0
1.5
f2 (ppm)
f1 (ppm)

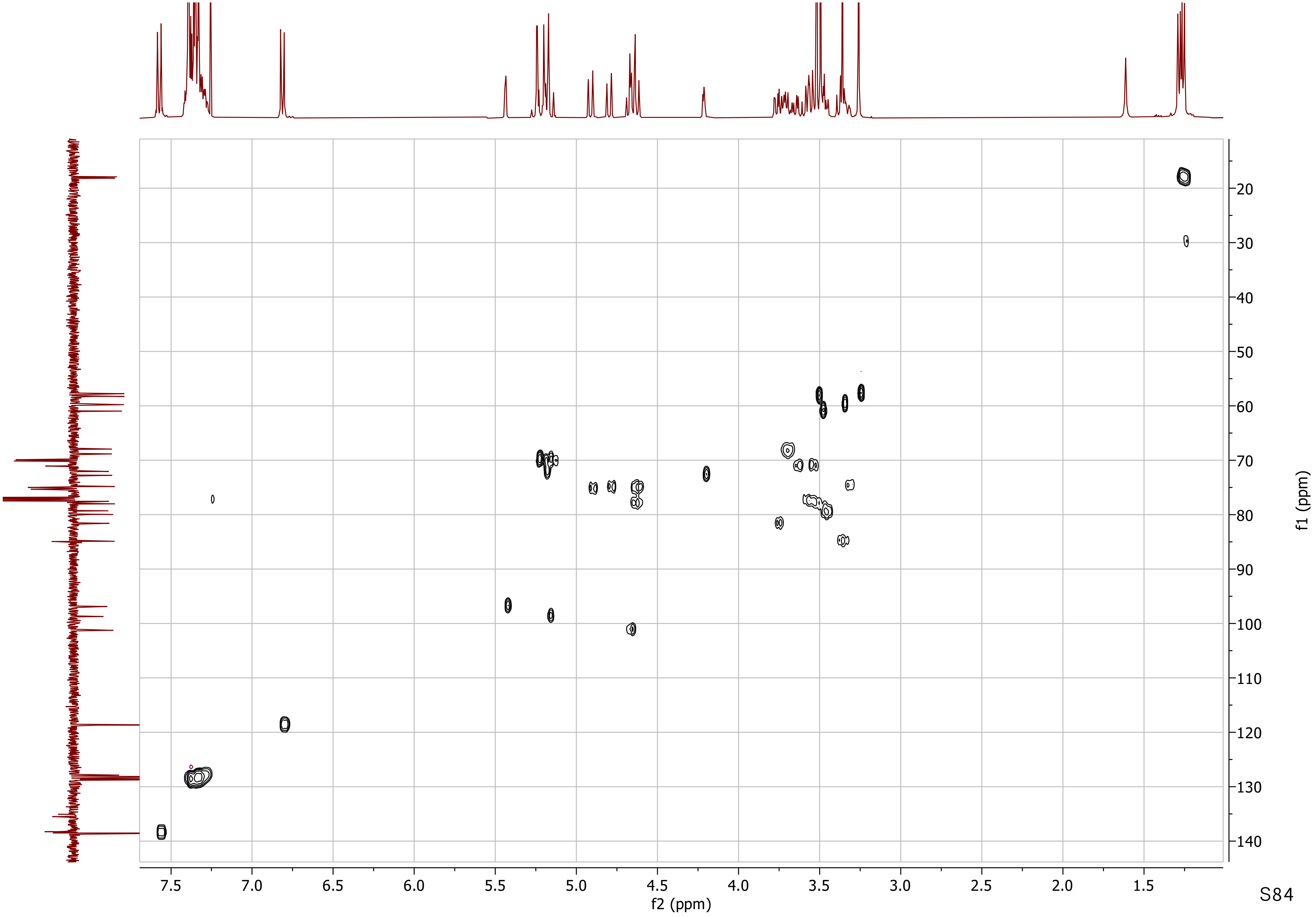

7.5
7.0
6.5
6.0
5.5
5.0
4.5
4.0
3.5
3.0
2.5
2.0
1.5
f2 (ppm)
20
30
40
50
60
70
80
90
100
110
120
130
140
f1 (ppm)

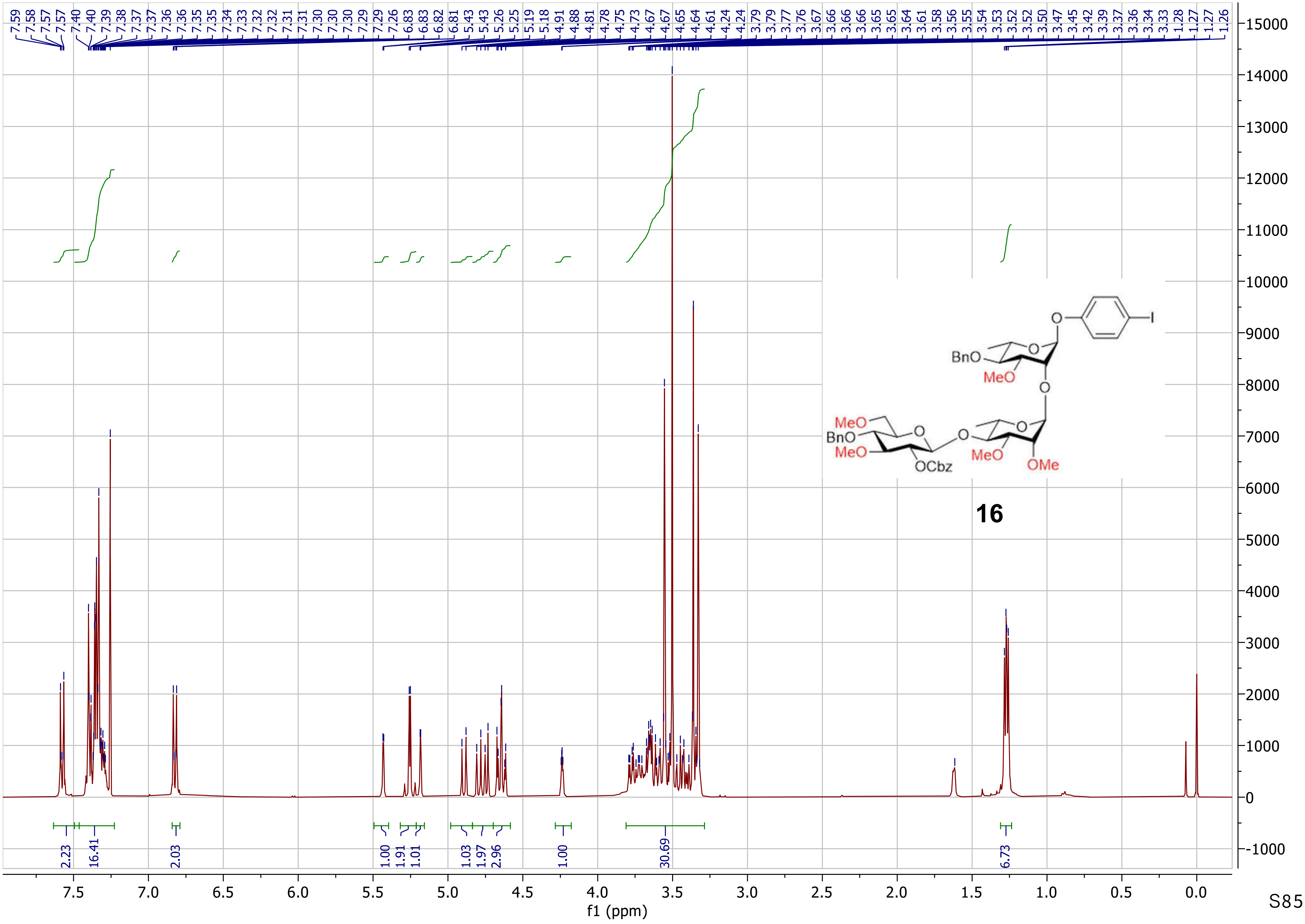

BnO
MeO
O
MeO
BnO
MeO
OCbz
MeO
OMe
I
16
f1 (ppm)

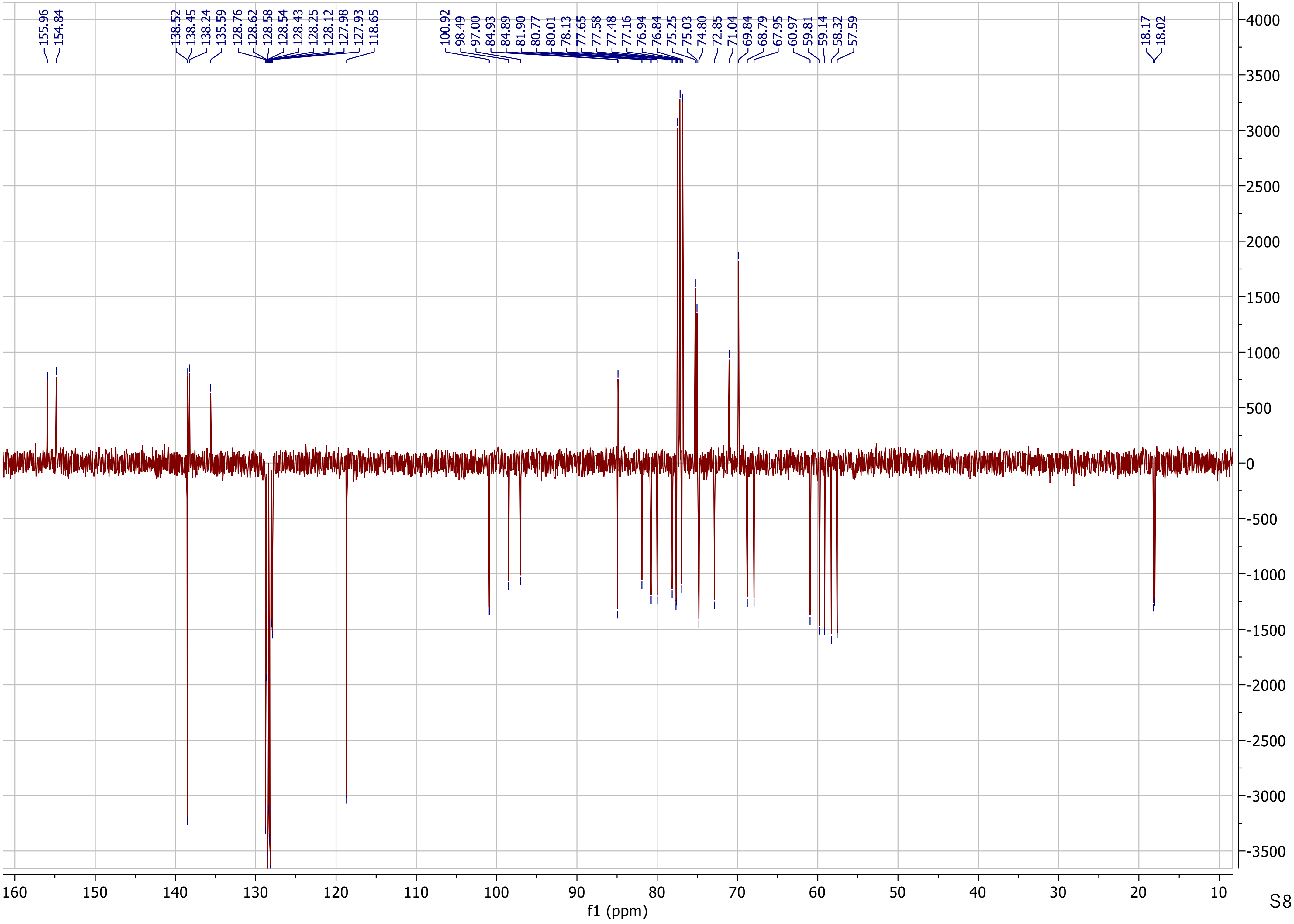
155.96
154.84
138.52
138.45
138.24
135.59
128.76
128.62
128.58
128.54
128.43
128.25
128.12
127.98
127.93
118.65
100.92
98.49
97.00
84.93
84.89
81.90
80.77
80.01
78.13
77.65
77.58
77.48
77.16
76.94
76.84
75.25
75.03
74.80
72.85
71.04
69.84
68.79
67.95
60.97
59.81
59.14
58.32
57.59
18.17
18.02
f1 (ppm)

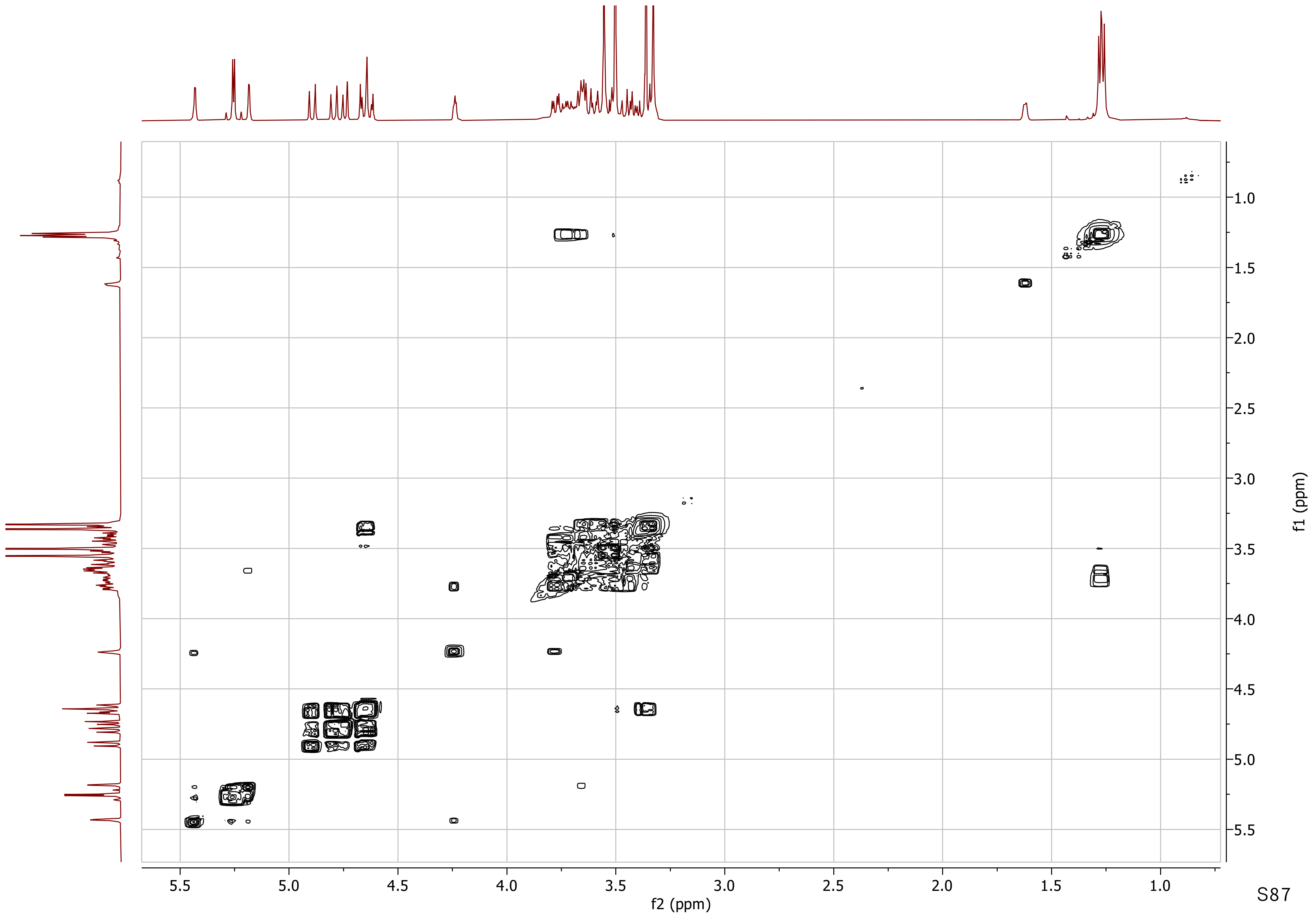

5.5
5.0
4.5
4.0
3.5
3.0
2.5
2.0
1.5
1.0
f2 (ppm)
1.0
1.5
2.0
2.5
3.0
3.5
4.0
4.5
5.0
5.5
f1 (ppm)

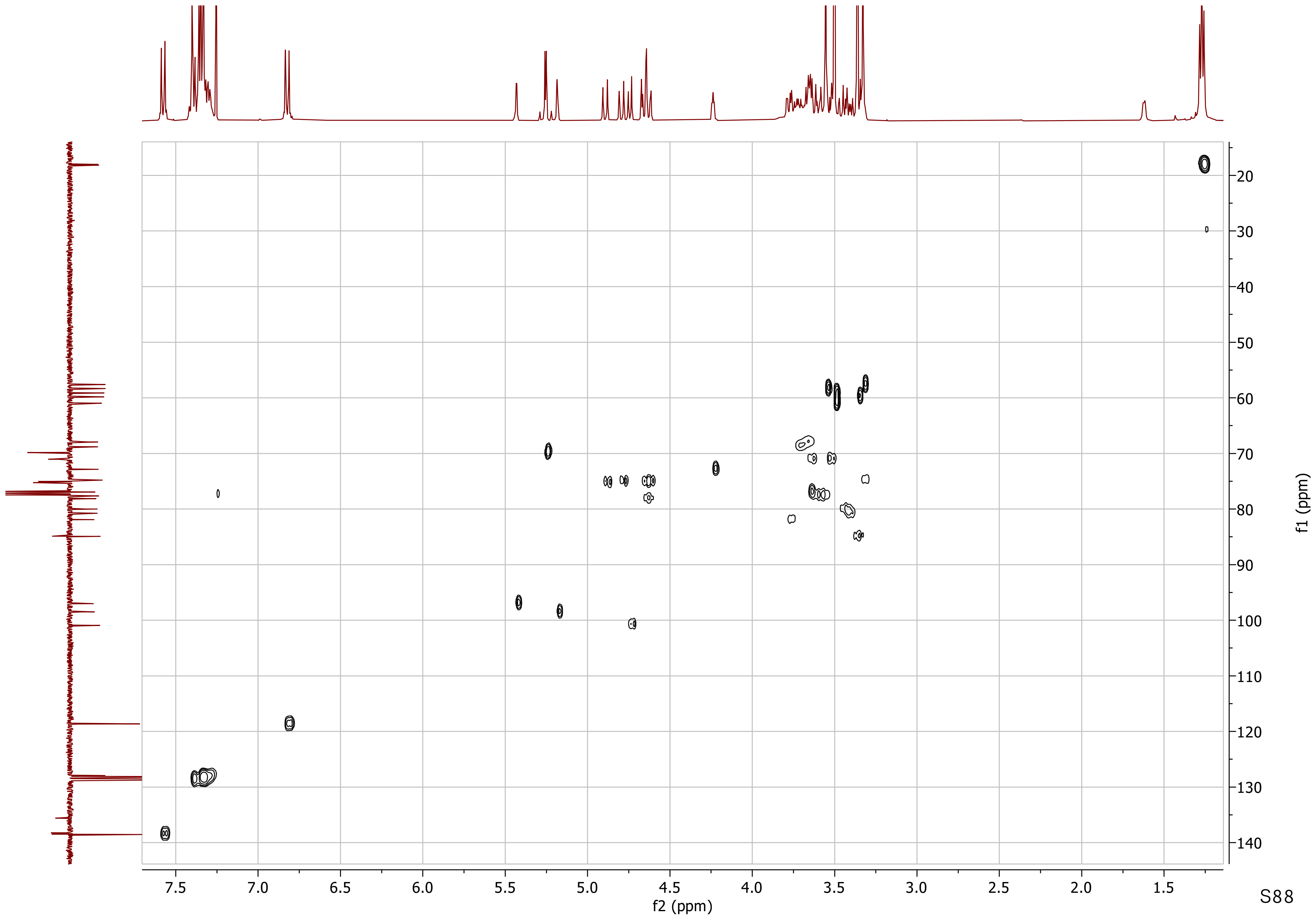
f2 (ppm)
f1 (ppm)

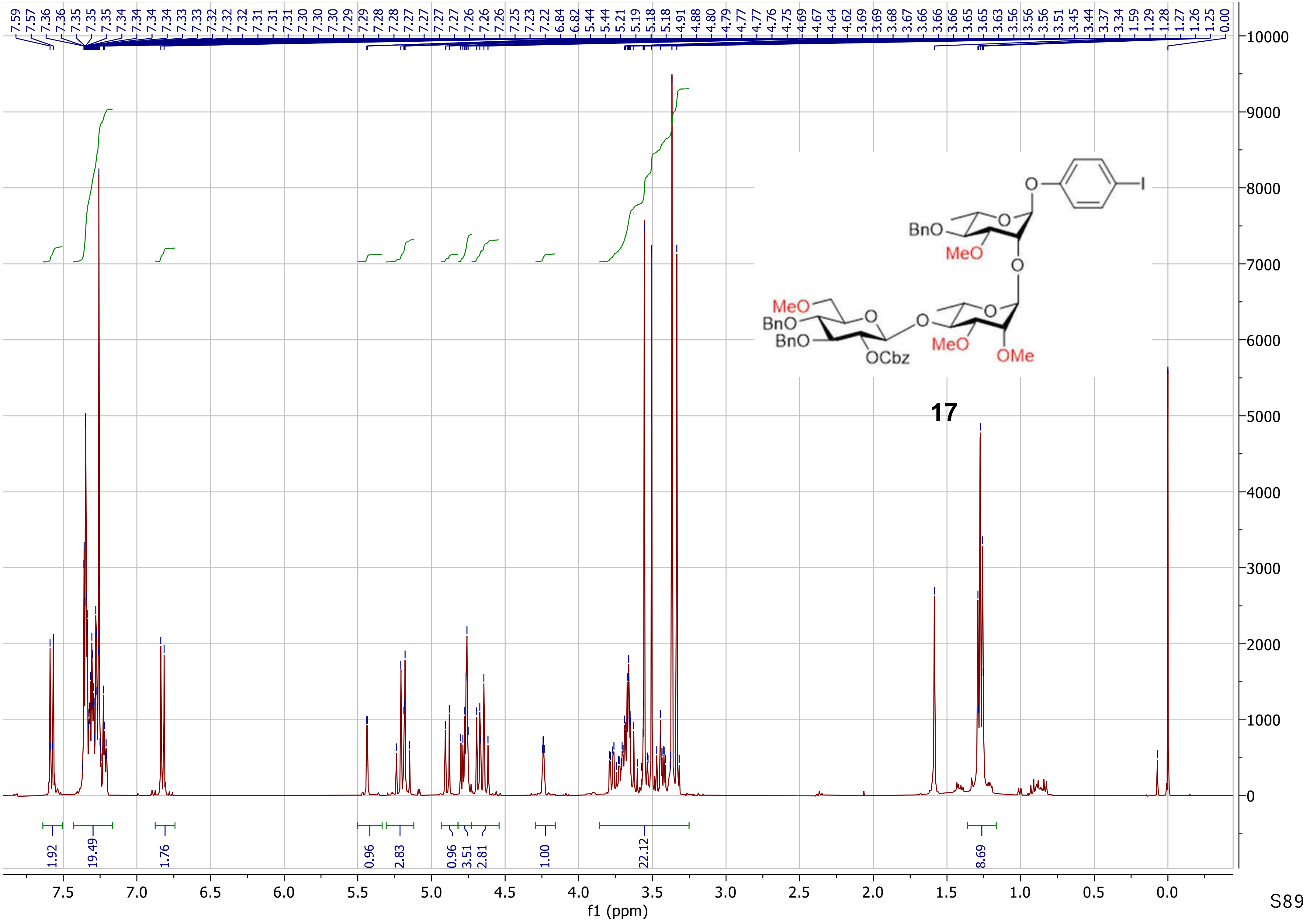

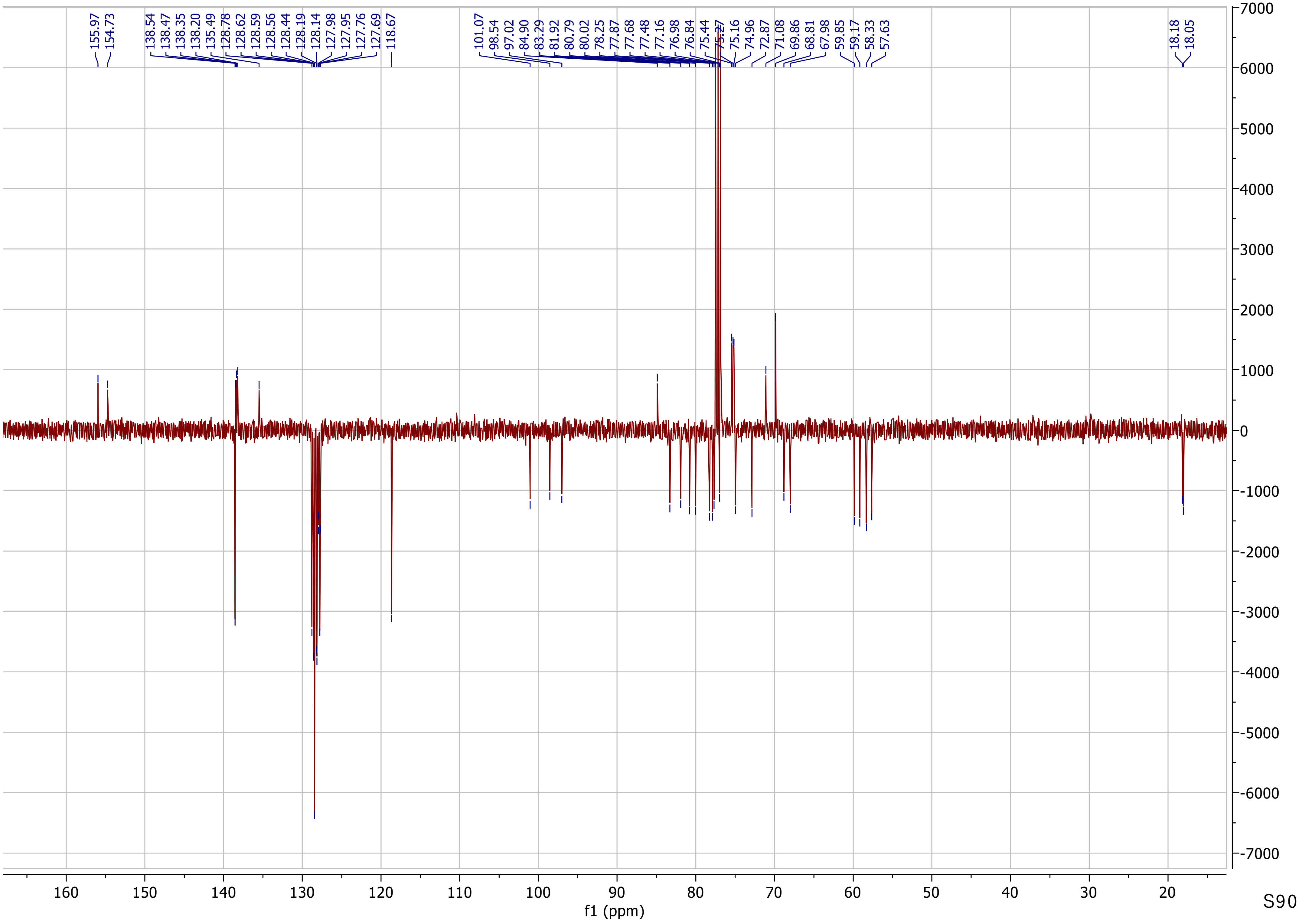
155.97
154.73
138.54
138.47
138.35
138.20
135.49
128.78
128.62
128.59
128.56
128.44
128.19
128.14
127.98
127.95
127.76
127.69
118.67
101.07
98.54
97.02
84.90
83.29
81.92
80.79
80.02
78.25
77.87
77.68
77.48
77.16
76.98
76.84
75.44
75.27
75.16
74.96
72.87
71.08
69.86
68.81
67.98
59.85
59.17
58.33
57.63
18.18
18.05
f1 (ppm)

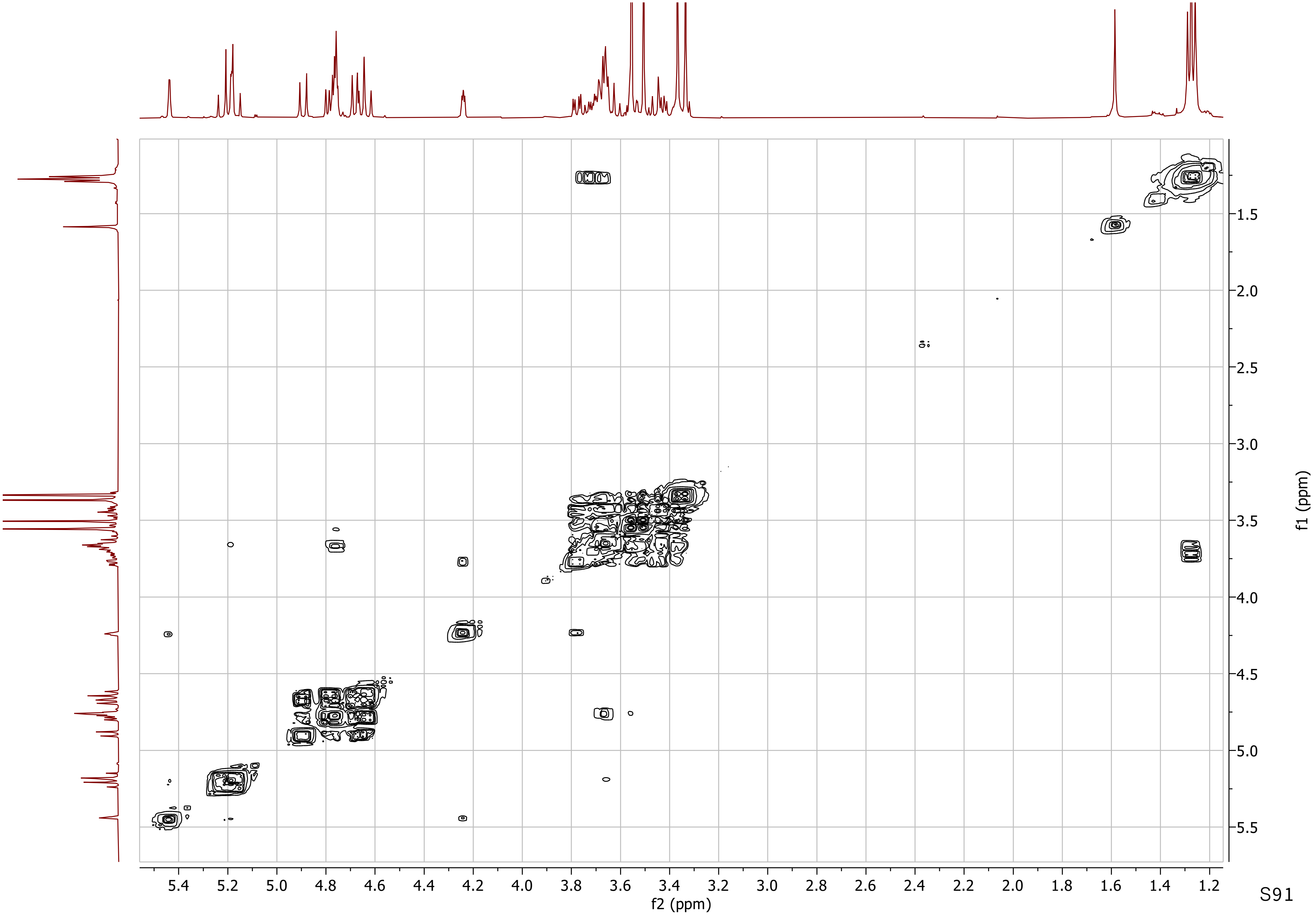

f2 (ppm)
f1 (ppm)

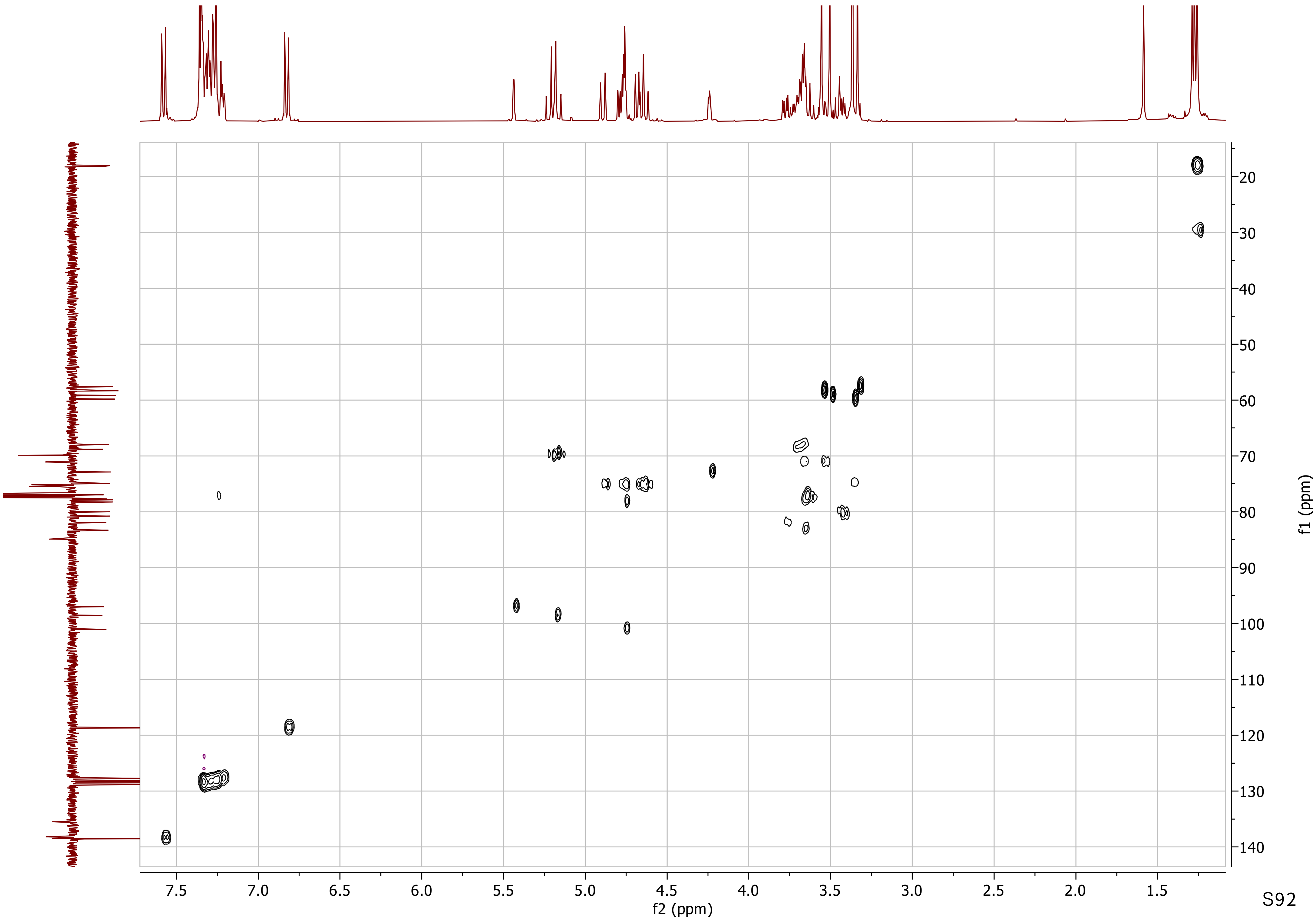

f1 (ppm)
f2 (ppm)
7.5
7.0
6.5
6.0
5.5
5.0
4.5
4.0
3.5
3.0
2.5
2.0
1.5
20
30
40
50
60
70
80
90
100
110
120
130
140

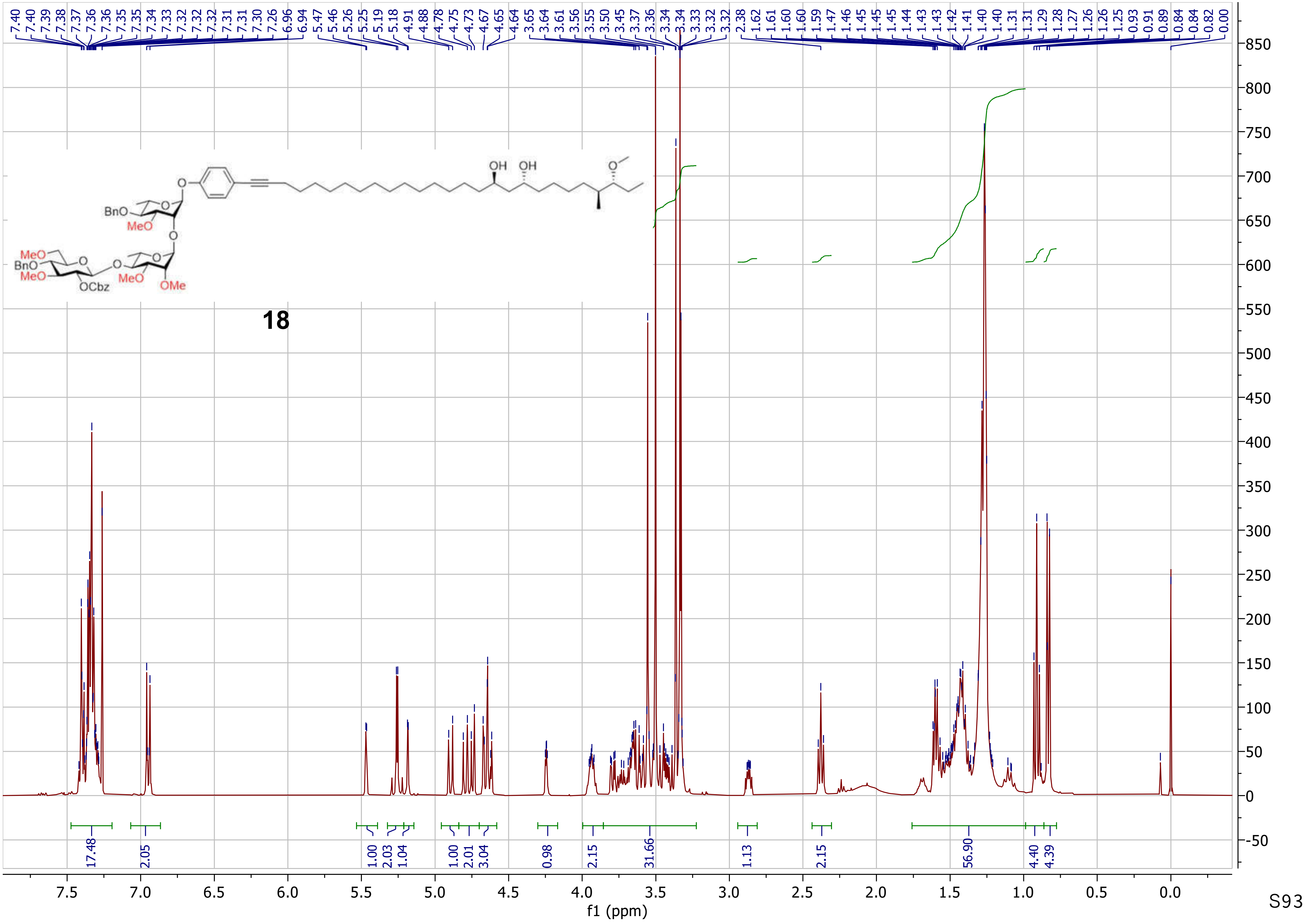
7.40 7.40 7.39 7.38 7.37 7.36 7.36 7.35 7.35 7.34 7.33 7.32 7.32 7.32 7.31 7.31 7.30 7.26 6.96 6.94 5.47 5.46 5.26 5.25 5.19 5.18 4.91 4.88 4.78 4.75 4.73 4.67 4.65 4.64 3.65 3.64 3.61 3.56 3.55 3.50 3.45 3.37 3.36 3.34 3.34 3.33 3.32 3.32 2.38 1.62 1.61 1.60 1.60 1.59 1.47 1.46 1.45 1.45 1.45 1.44 1.43 1.43 1.42 1.41 1.40 1.40 1.31 1.31 1.29 1.28 1.27 1.26 1.26 1.25 0.93 0.91 0.89 0.84 0.84 0.82 0.00
OH
OH
O
BnO
MeO
MeO
BnO
MeO
OCbz
MeO
OMe
18
17.48
2.05
1.00
2.03
1.04
1.00
2.01
3.04
0.98
2.15
31.66
1.13
2.15
56.90
4.40
4.39
f1 (ppm)

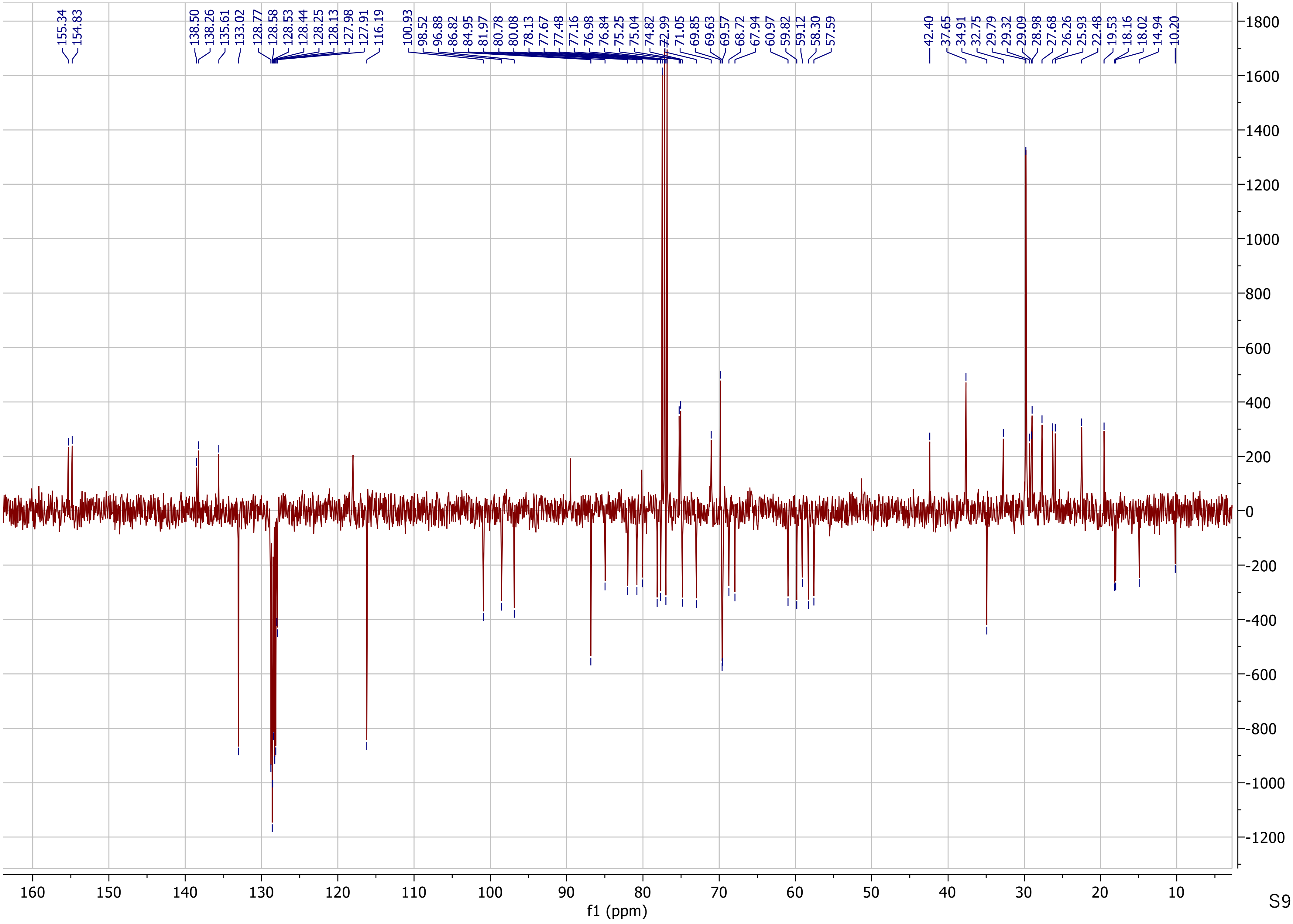
155.34
154.83
138.50
138.26
135.61
133.02
128.77
128.58
128.53
128.44
128.25
128.13
127.98
127.91
116.19
100.93
98.52
96.88
86.82
84.95
81.97
80.78
80.08
78.13
77.67
77.48
77.16
76.98
76.84
75.25
75.04
74.82
72.99
71.05
69.85
69.63
69.57
68.72
67.94
60.97
59.82
59.12
58.30
57.59
42.40
37.65
34.91
32.75
29.79
29.32
29.09
28.98
27.68
26.26
25.93
22.48
19.53
18.16
18.02
14.94
10.20
f1 (ppm)

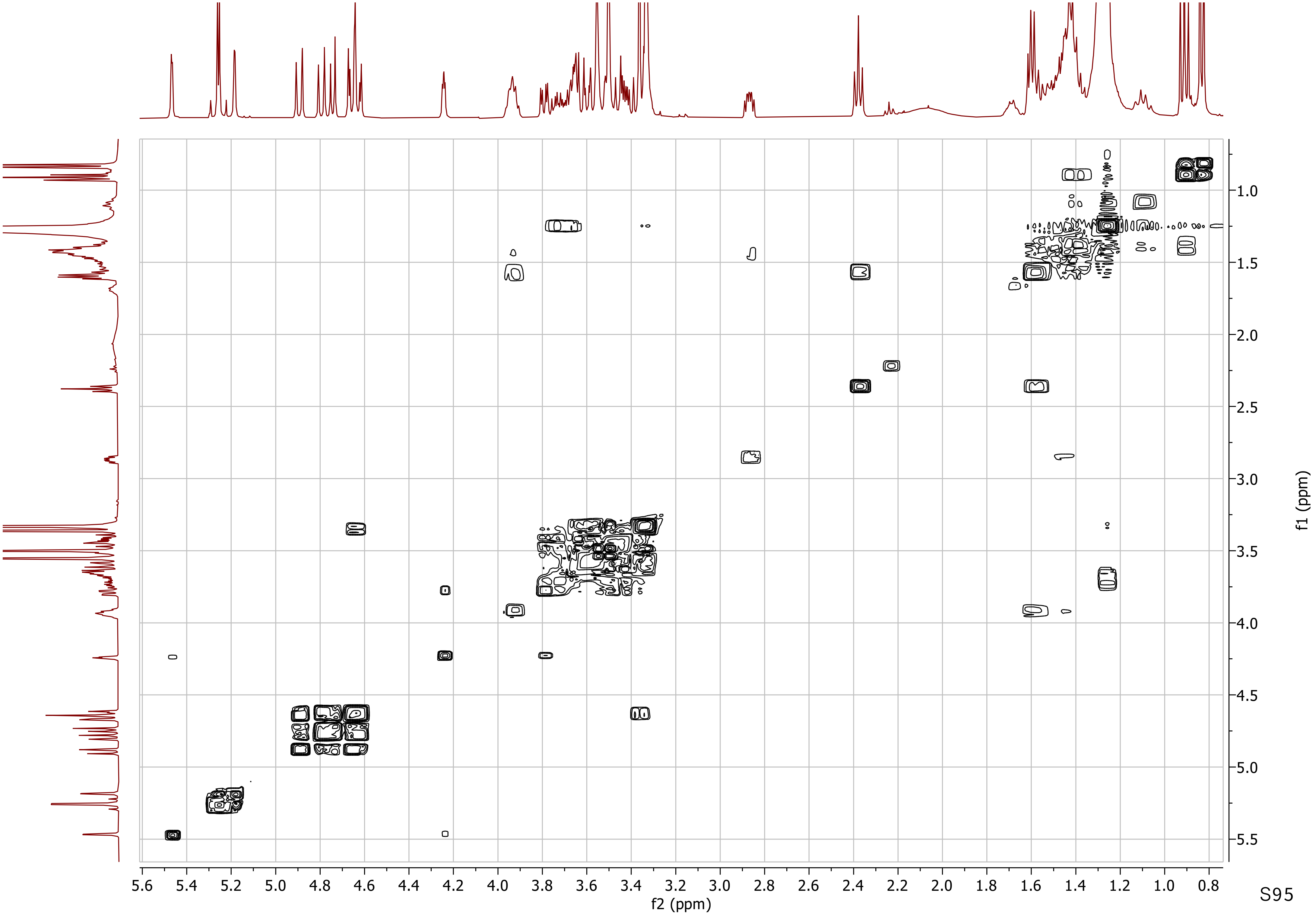

f2 (ppm)
f1 (ppm)

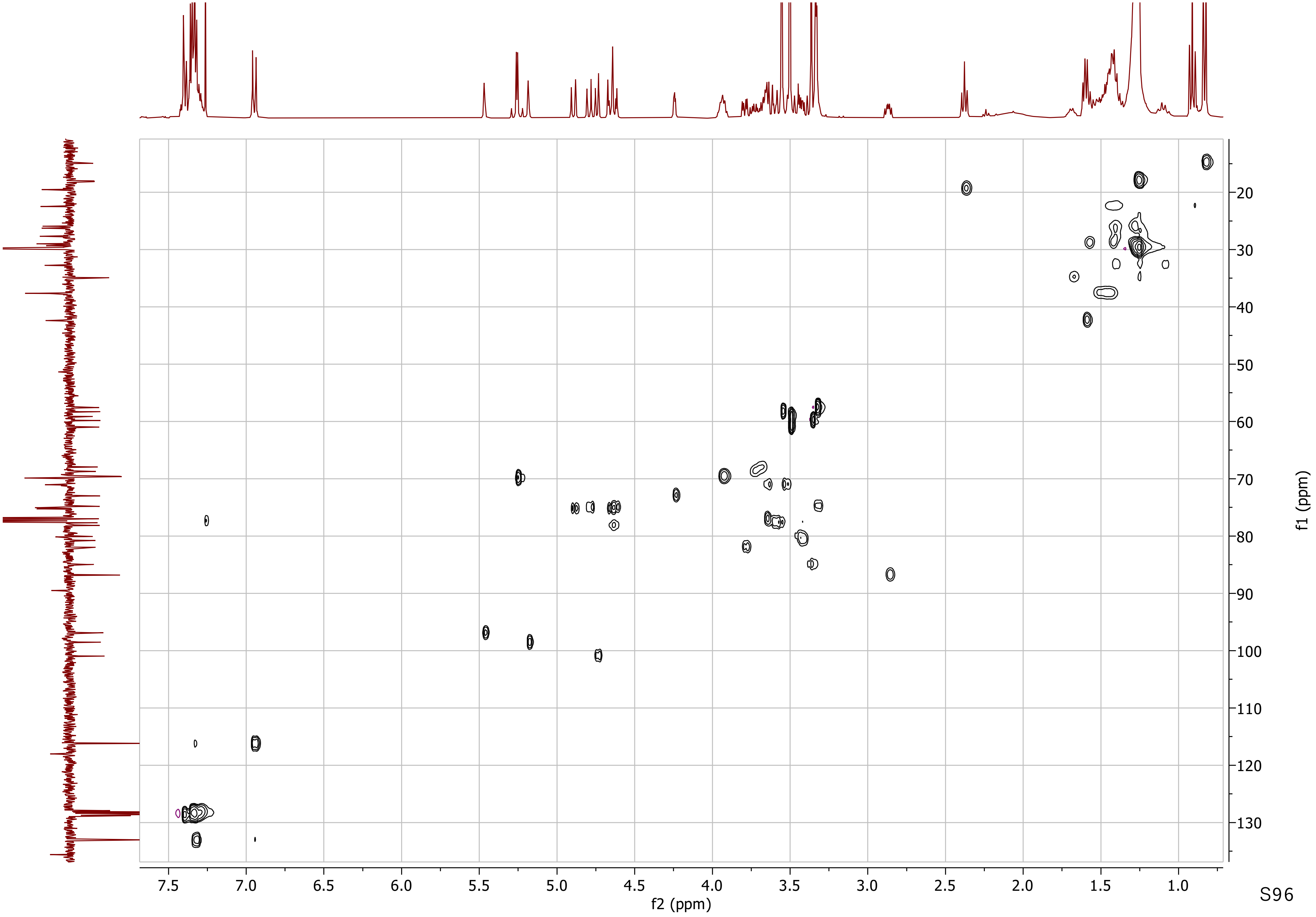
7.5
7.0
6.5
6.0
5.5
5.0
4.5
4.0
3.5
3.0
2.5
2.0
1.5
1.0
f2 (ppm)
20
30
40
50
60
70
80
90
100
110
120
130
f1 (ppm)

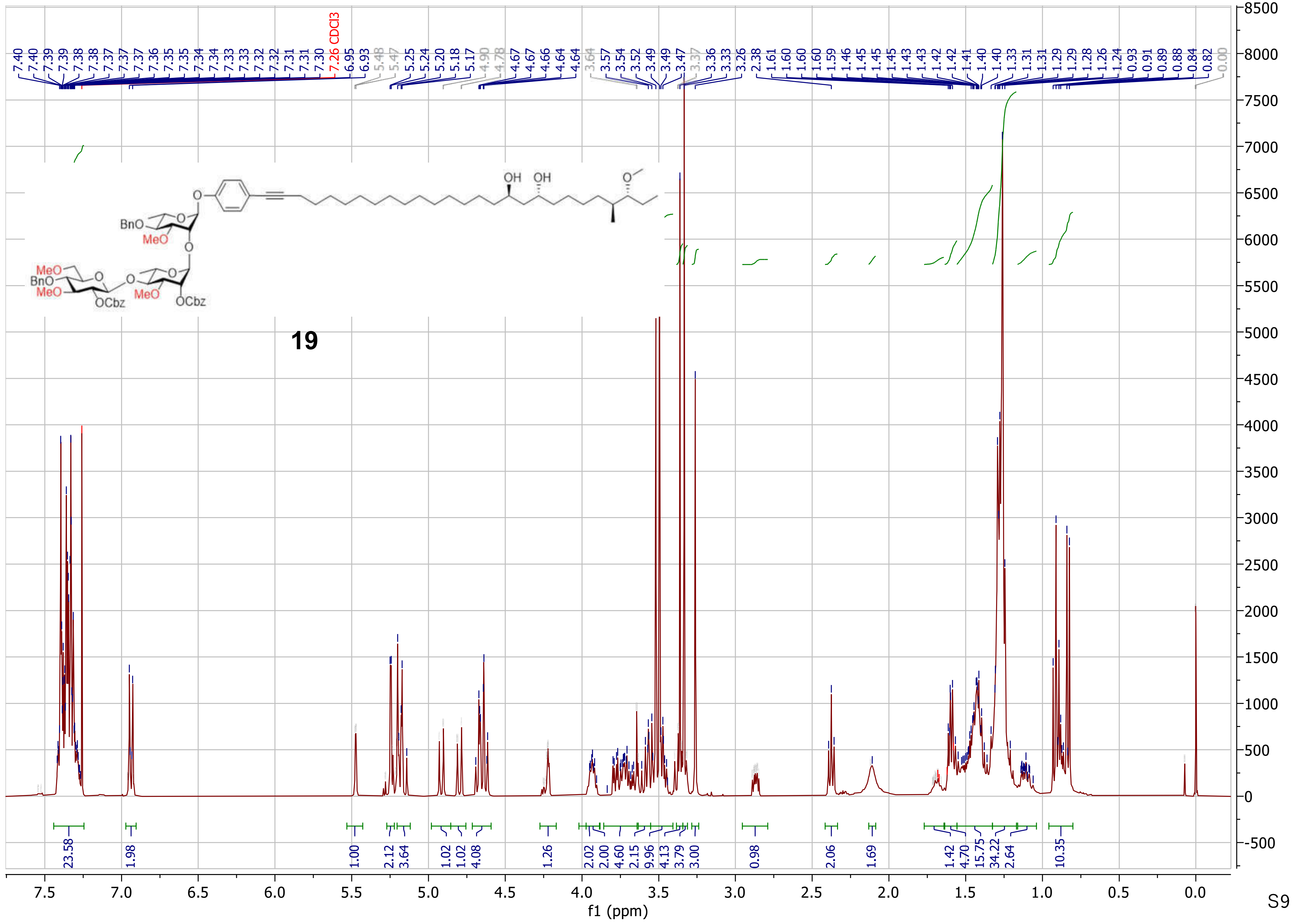
7.26 CDCl3
OH OH
BnO
MeO
MeO
BnO
MeO
OCbz
MeO
OCbz
19
23.58
1.98
1.00
2.12
3.64
1.02
1.02
4.08
1.26
2.02
2.00
4.60
2.15
9.96
4.13
3.79
3.00
0.98
2.06
1.69
1.42
4.70
15.75
34.22
2.64
10.35
f1 (ppm)

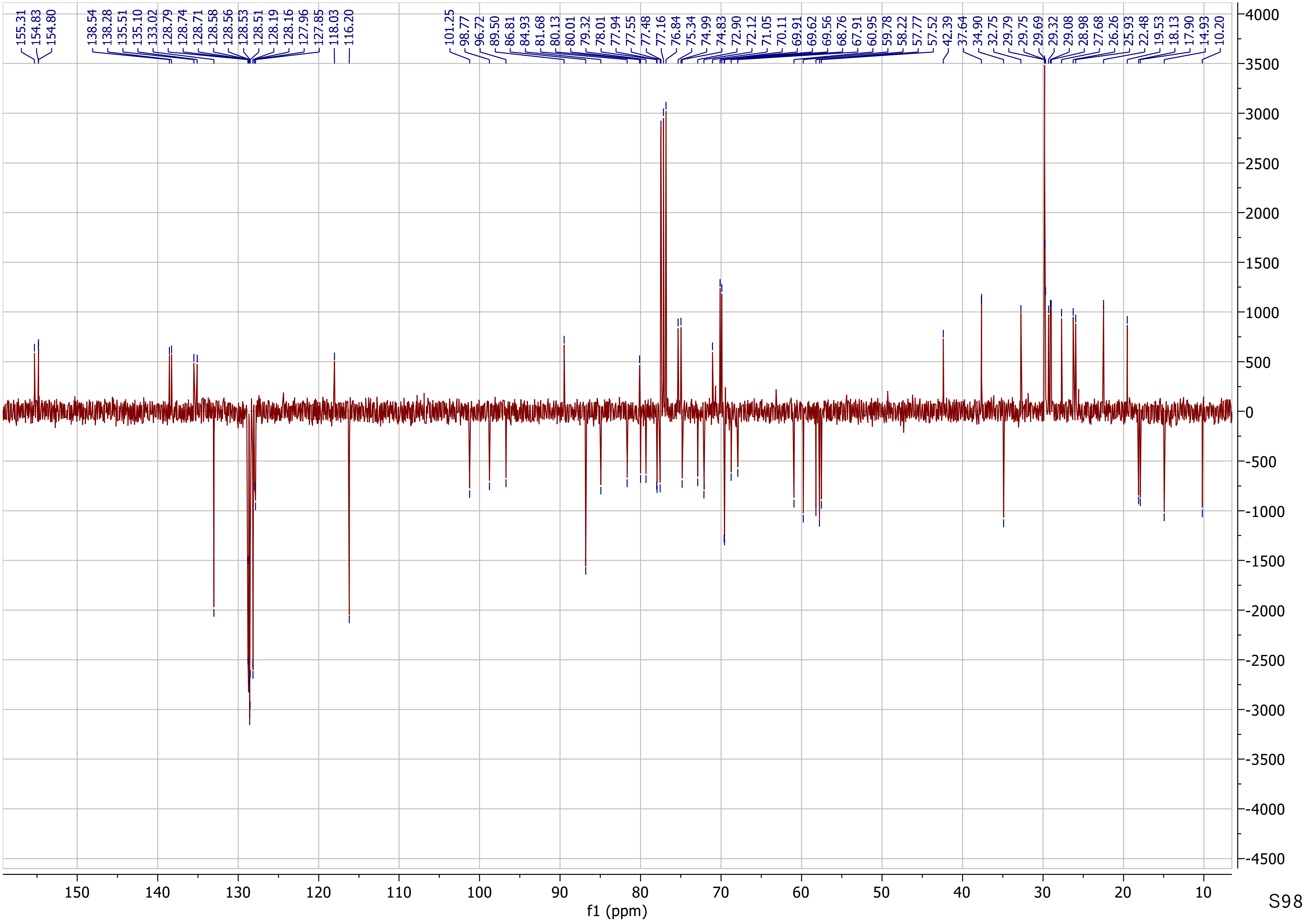
155.31
154.83
154.80
138.54
138.28
135.51
135.10
133.02
128.79
128.74
128.71
128.58
128.56
128.53
128.51
128.19
128.16
127.96
127.85
118.03
116.20
101.25
98.77
96.72
89.50
86.81
84.93
81.68
80.13
80.01
79.32
78.01
77.94
77.55
77.48
77.16
76.84
75.34
74.99
74.83
72.90
72.12
71.05
70.11
69.91
69.62
69.56
68.76
67.91
60.95
59.78
58.22
57.77
57.52
42.39
37.64
34.90
32.75
29.79
29.75
29.69
29.32
29.08
28.98
27.68
26.26
25.93
22.48
19.53
18.13
17.90
14.93
10.20
f1 (ppm)

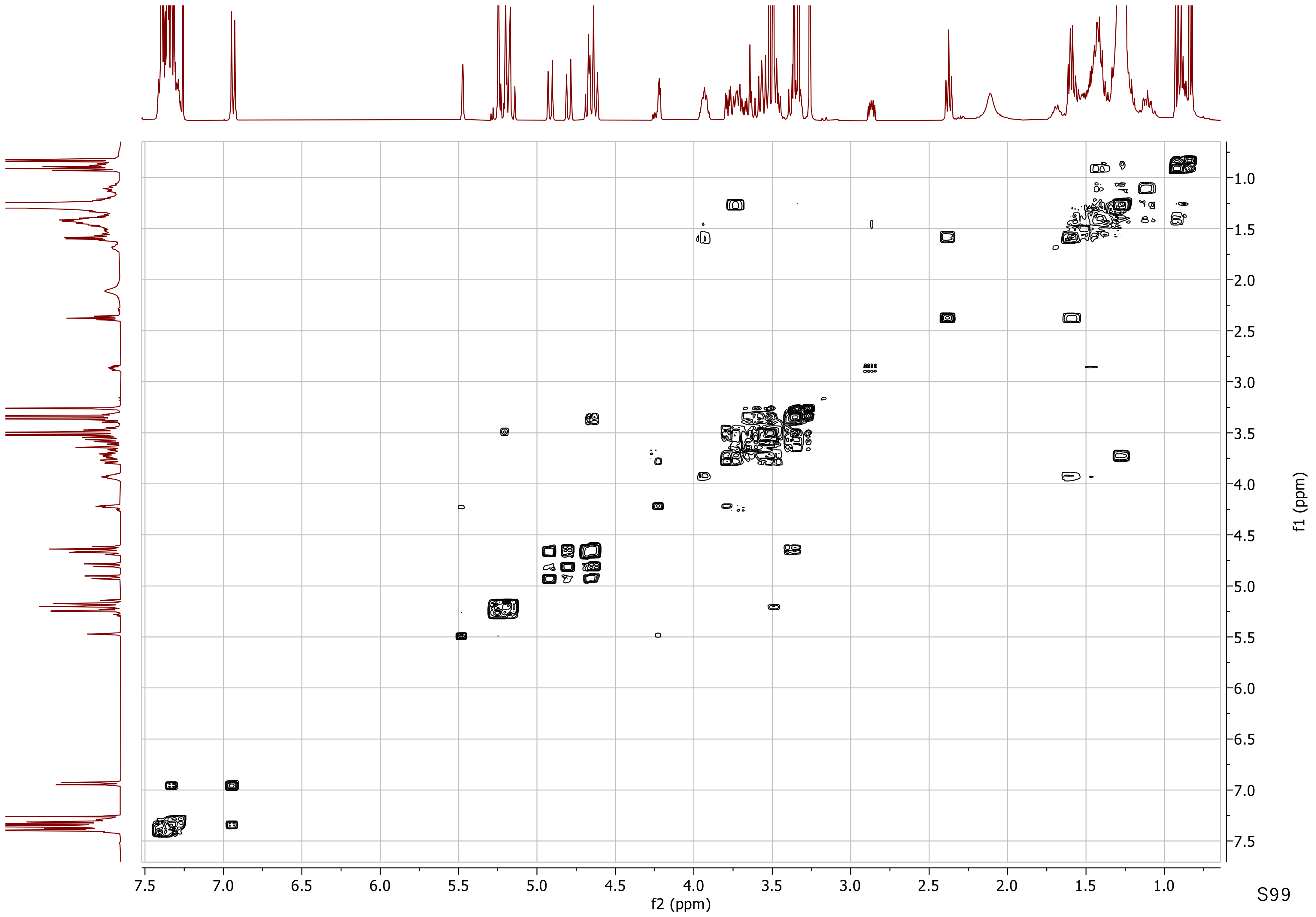

f2 (ppm)
f1 (ppm)

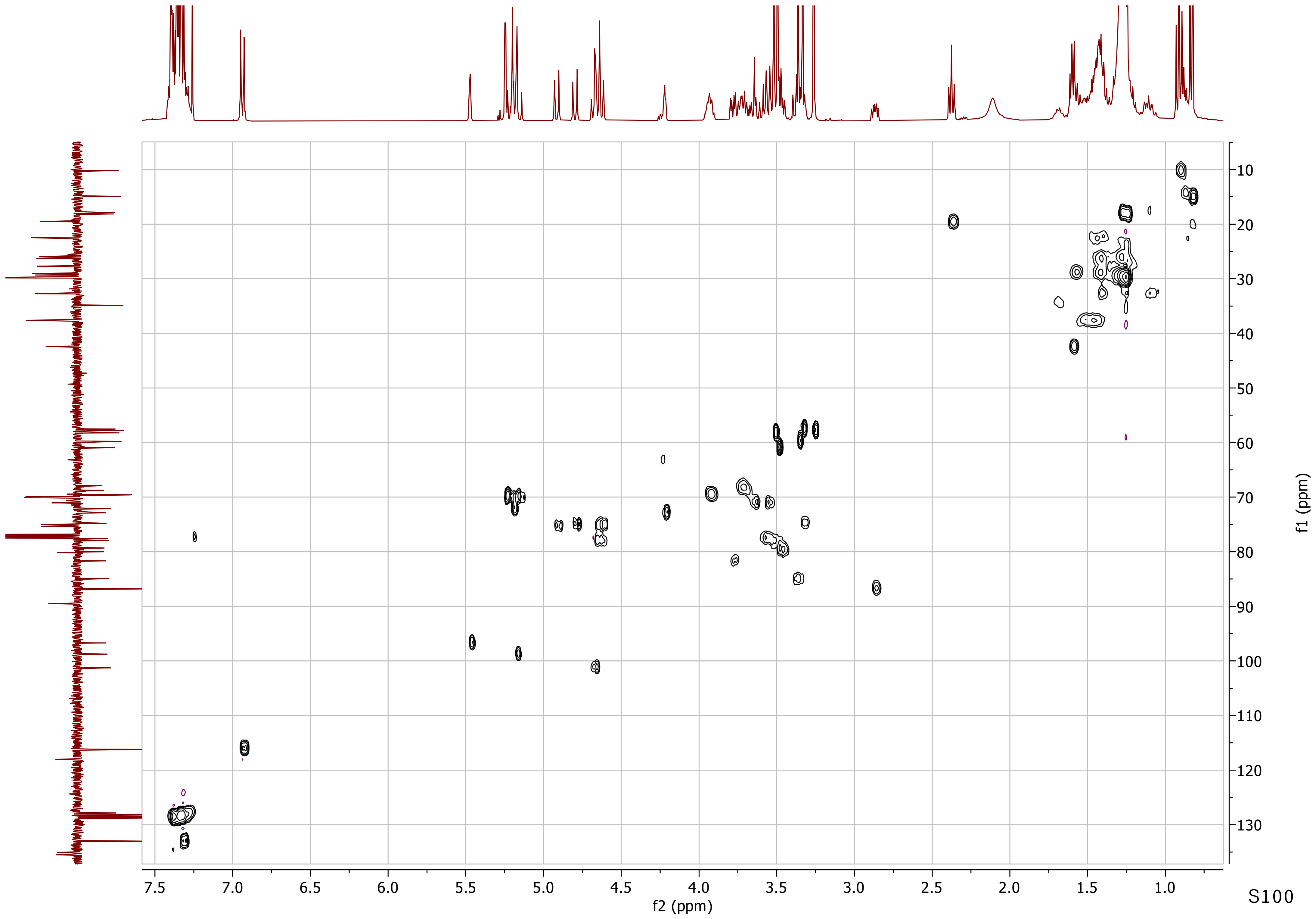

7.5
7.0
6.5
6.0
5.5
5.0
4.5
4.0
3.5
3.0
2.5
2.0
1.5
1.0
f2 (ppm)
10
20
30
40
50
60
70
80
90
100
110
120
130
f1 (ppm)

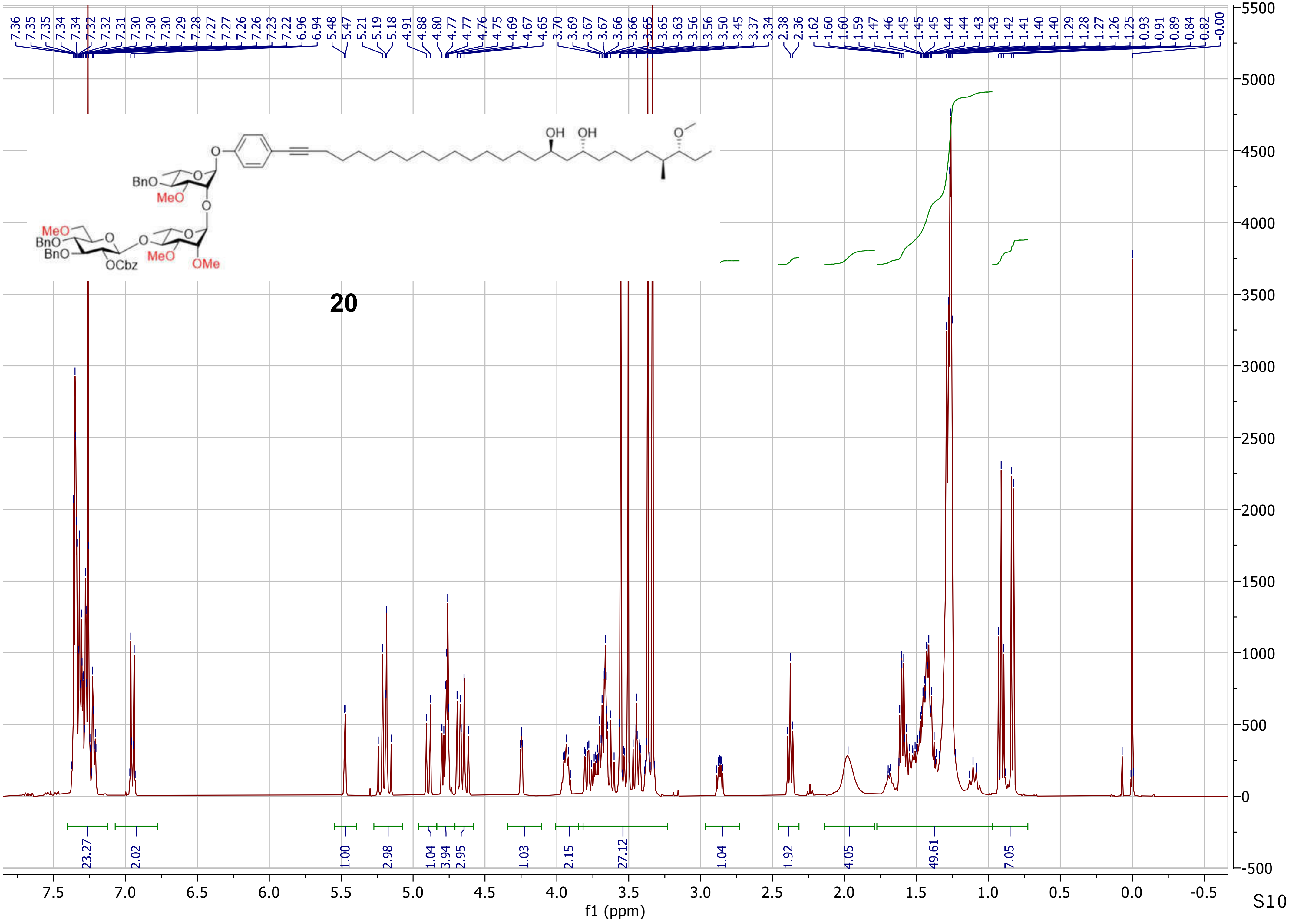

20

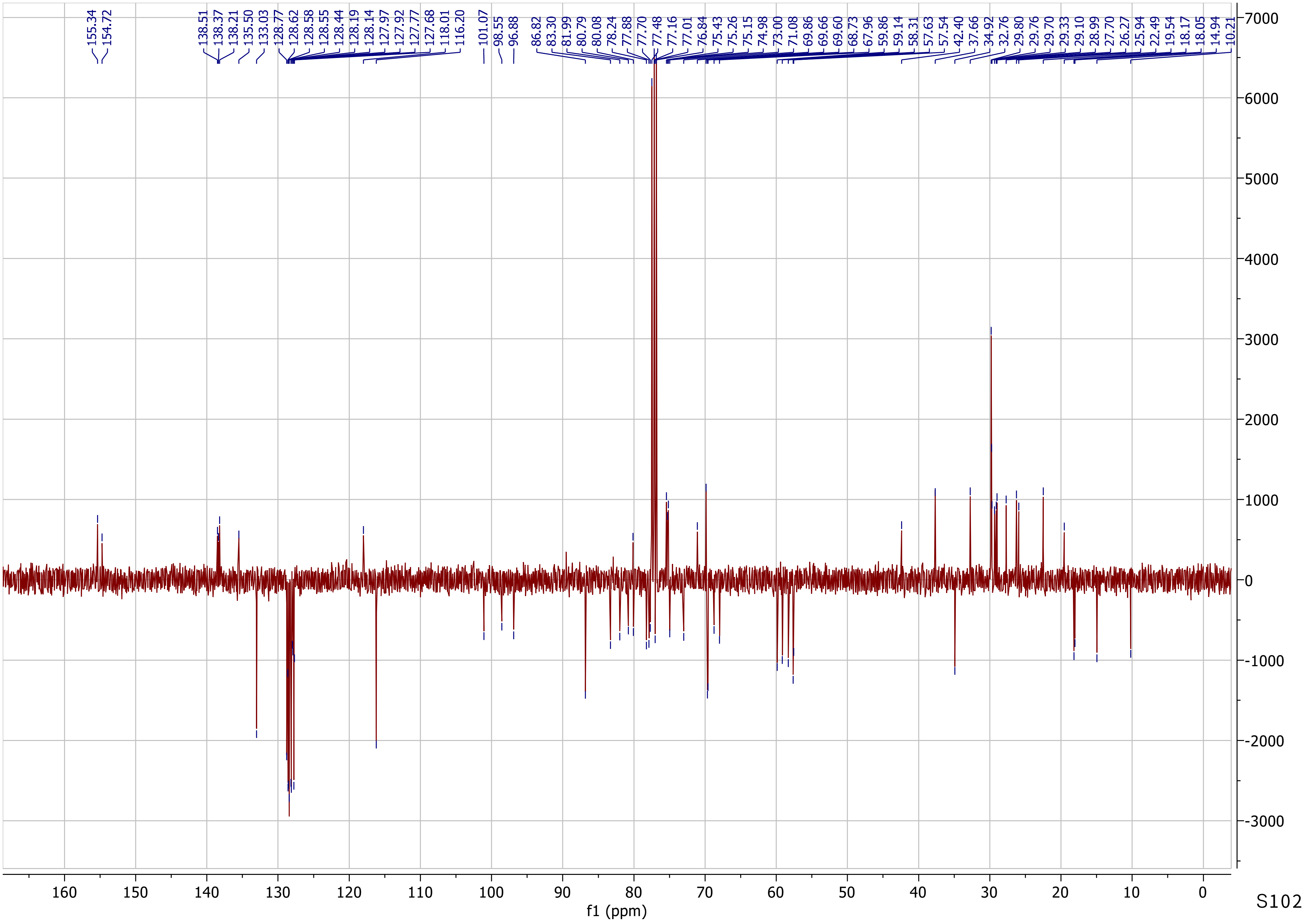
155.34
154.72
138.51
138.37
138.21
135.50
133.03
128.77
128.62
128.58
128.55
128.44
128.19
128.14
127.97
127.92
127.77
127.68
118.01
116.20
101.07
98.55
96.88
86.82
83.30
81.99
80.79
80.08
78.24
77.88
77.70
77.48
77.16
77.01
76.84
75.43
75.26
75.15
74.98
73.00
71.08
69.86
69.66
69.60
68.73
67.96
59.86
59.14
58.31
57.63
57.54
42.40
37.66
34.92
32.76
29.80
29.76
29.70
29.33
29.10
28.99
27.70
26.27
25.94
22.49
19.54
18.17
18.05
14.94
10.21
f1 (ppm)

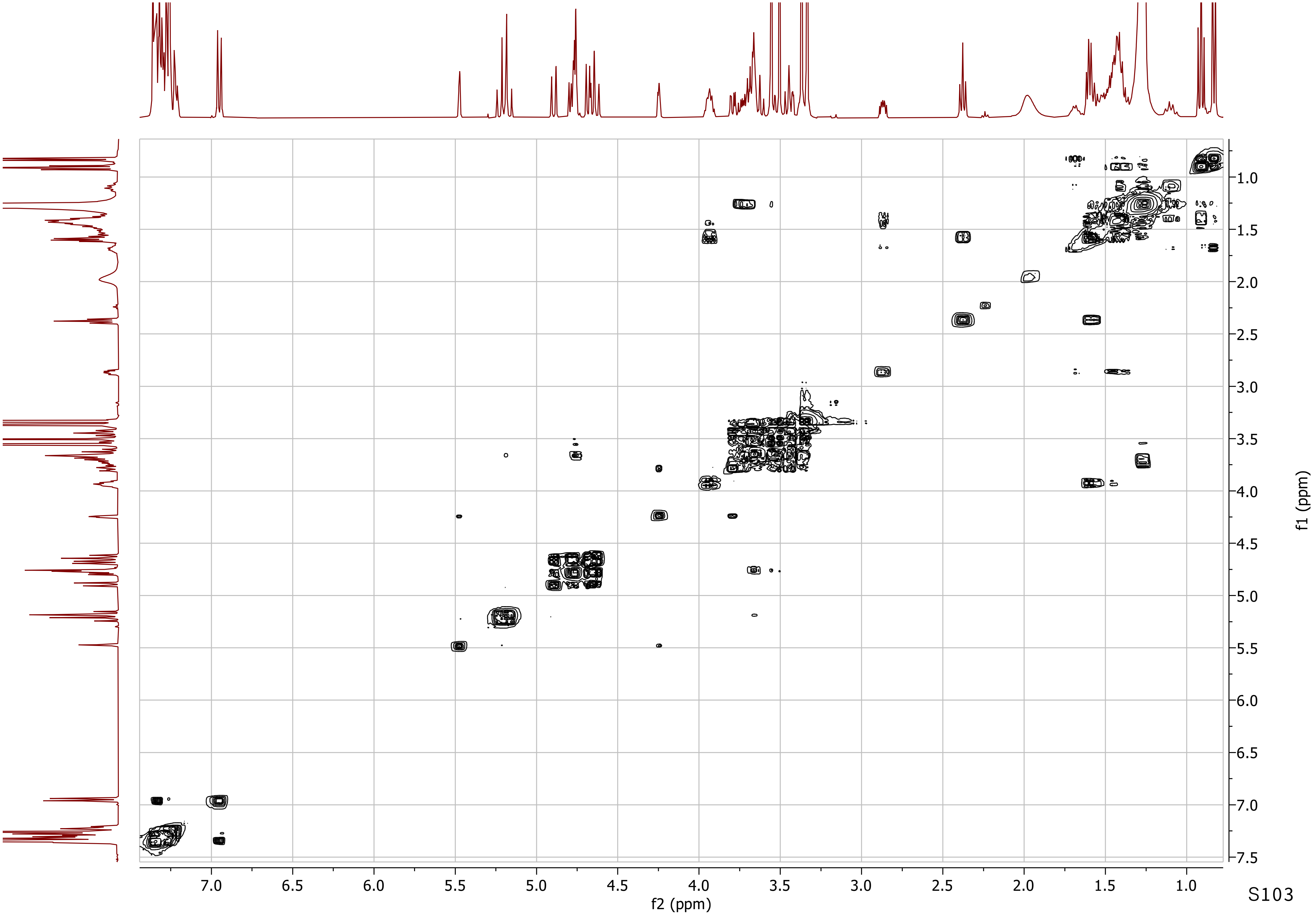

f2 (ppm)
f1 (ppm)

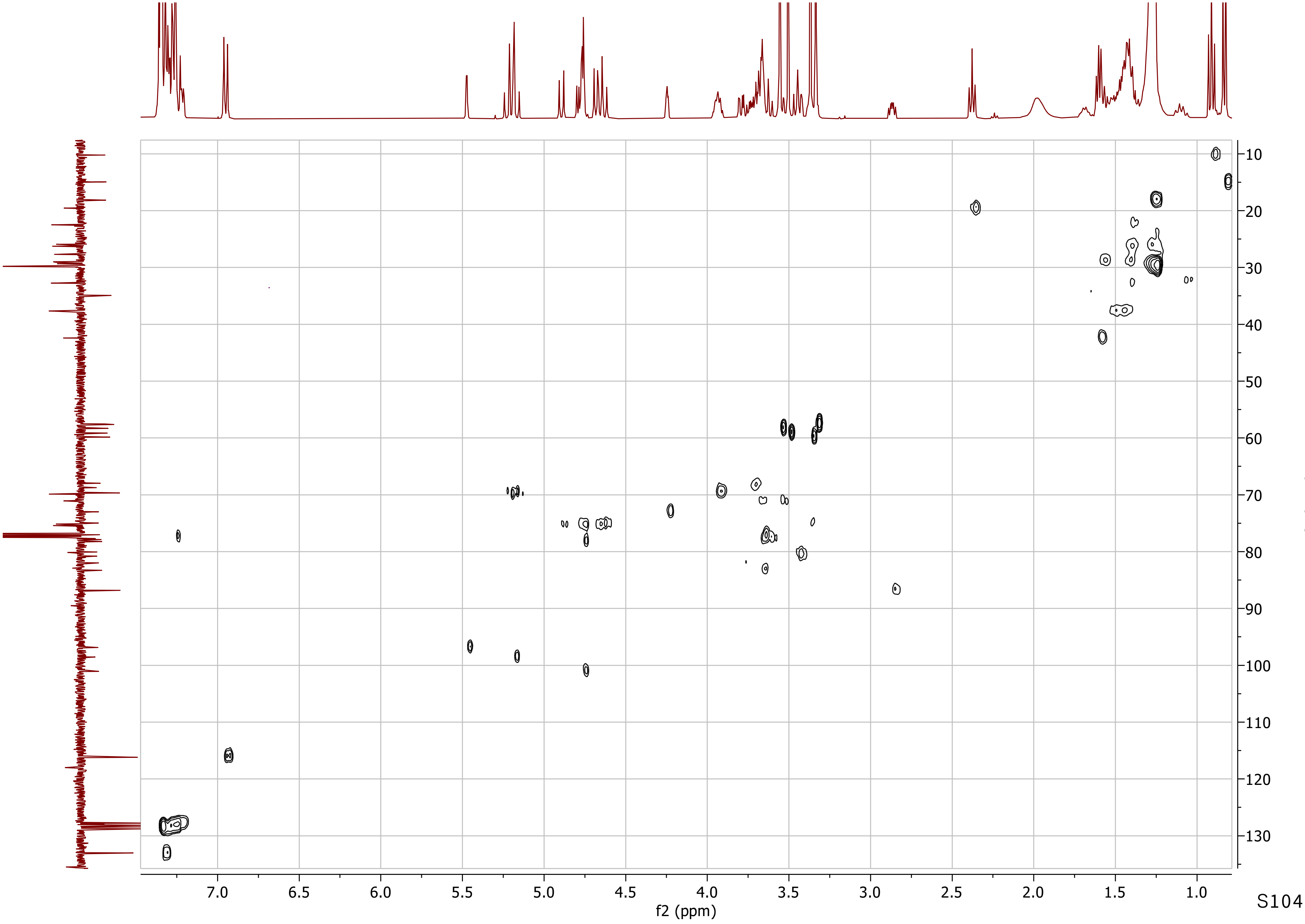
f2 (ppm)
f1 (ppm)

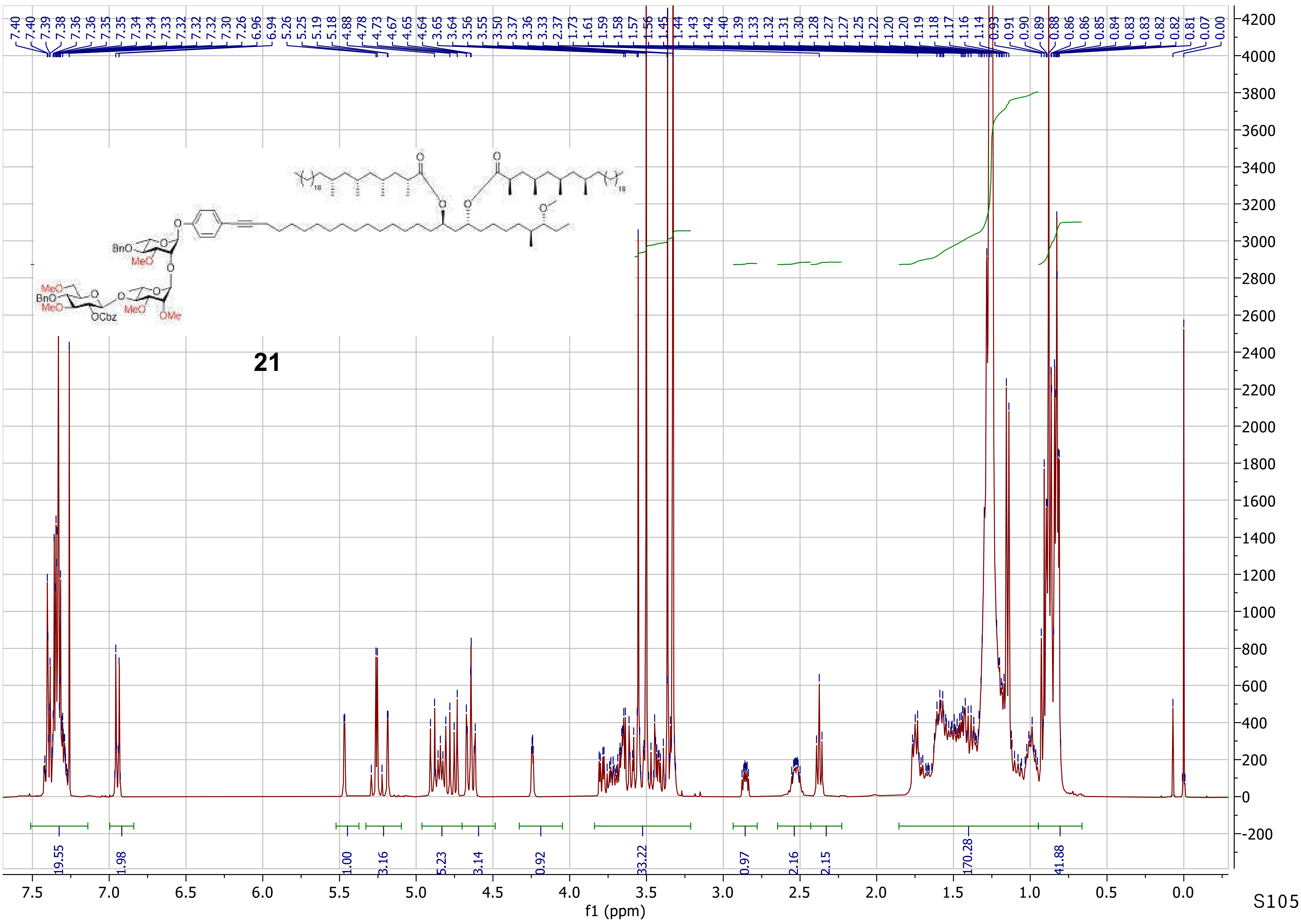
7.40 7.40 7.39 7.38 7.36 7.36 7.35 7.35 7.34 7.34 7.33 7.32 7.32 7.32 7.30 7.26 6.96 6.94 5.26 5.25 5.19 5.18 4.88 4.78 4.73 4.67 4.65 4.64 3.65 3.64 3.56 3.55 3.50 3.37 3.36 3.33 2.37 1.73 1.61 1.59 1.58 1.57 1.56 1.45 1.44 1.43 1.42 1.40 1.39 1.33 1.32 1.31 1.30 1.28 1.27 1.27 1.25 1.22 1.20 1.20 1.19 1.18 1.17 1.16 1.14 0.93 0.91 0.90 0.89 0.88 0.86 0.86 0.85 0.84 0.83 0.83 0.82 0.82 0.81 0.07 0.00
BnO
MeO
MeO
BnO
MeO
OCbz
MeO
OMe
21
19.55 1.98 1.00 3.16 5.23 3.14 0.92 33.22 0.97 2.16 2.15 170.28 41.88
f1 (ppm)

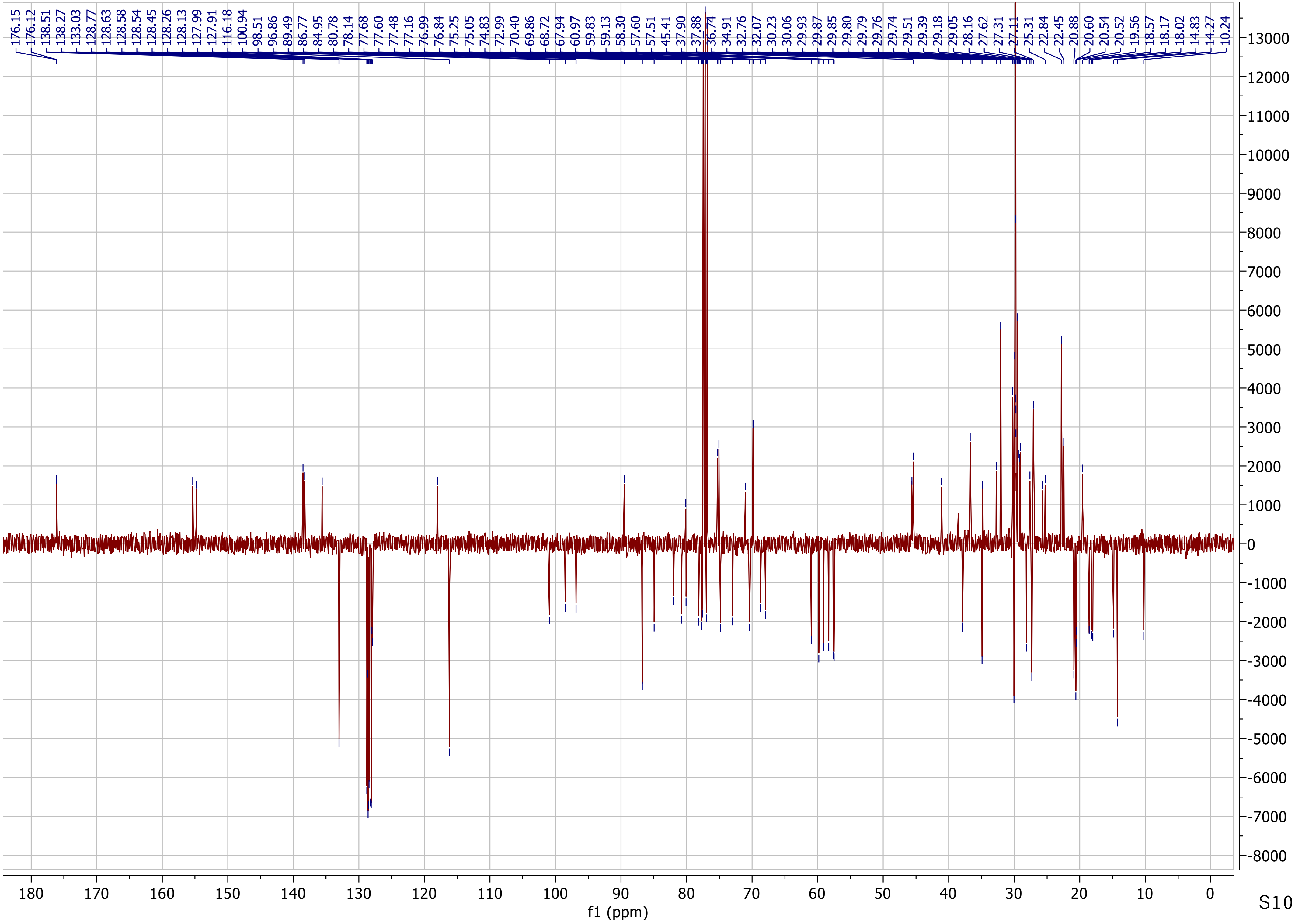
176.15
176.12
138.51
138.27
133.03
128.77
128.63
128.58
128.54
128.45
128.26
128.13
127.99
127.91
116.18
100.94
98.51
96.86
89.49
86.77
84.95
80.78
78.14
77.68
77.60
77.48
77.16
76.99
76.84
75.25
75.05
74.83
72.99
70.40
69.86
68.72
67.94
60.97
59.83
59.13
58.30
57.60
57.51
45.41
37.90
37.88
36.74
34.91
32.76
32.07
30.23
30.06
29.93
29.87
29.85
29.80
29.79
29.76
29.74
29.51
29.39
29.18
29.05
28.16
27.62
27.31
27.11
25.31
22.84
22.45
20.88
20.60
20.54
20.52
19.56
18.57
18.17
18.02
14.83
14.27
10.24
f1 (ppm)

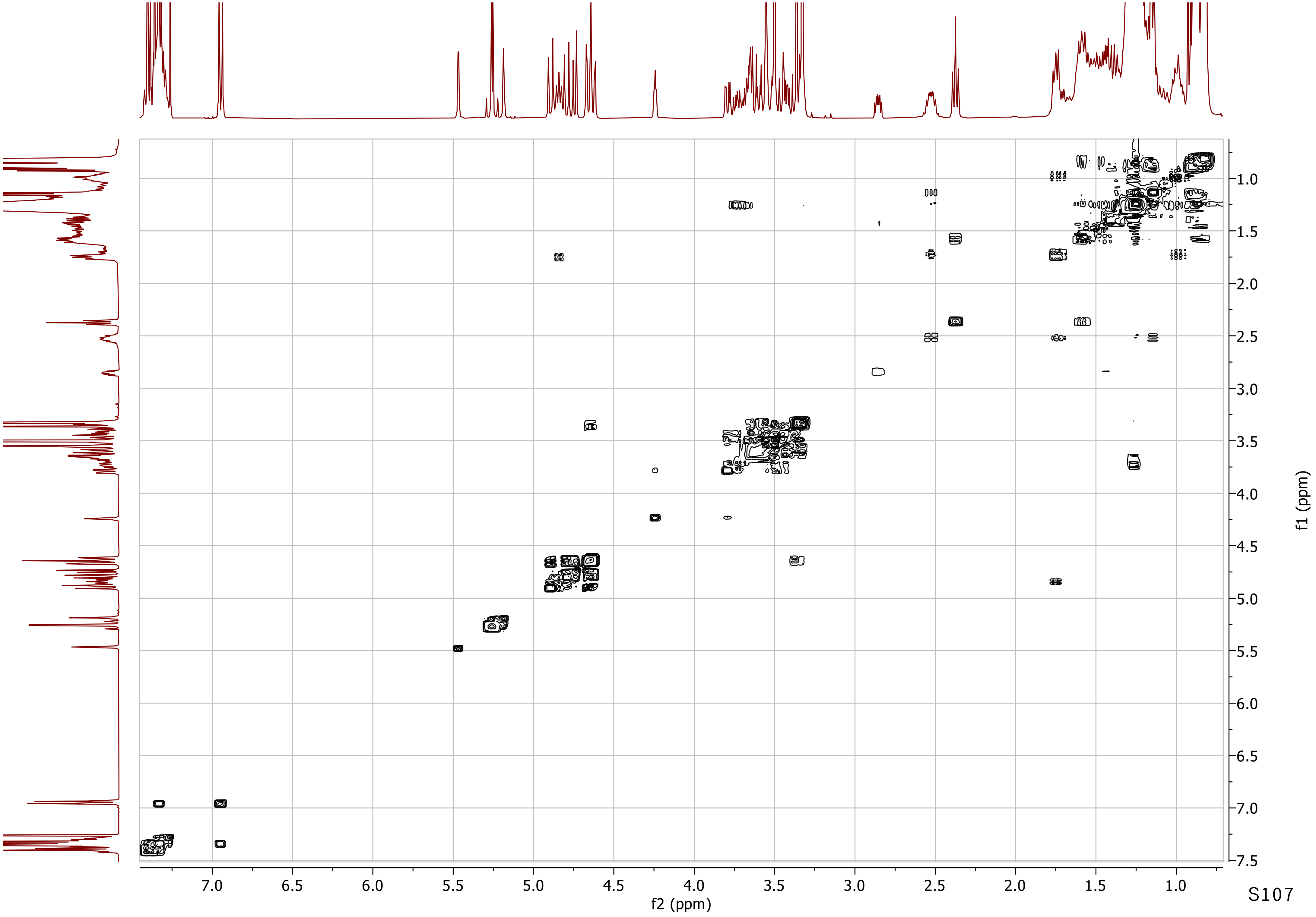

f2 (ppm)
f1 (ppm)

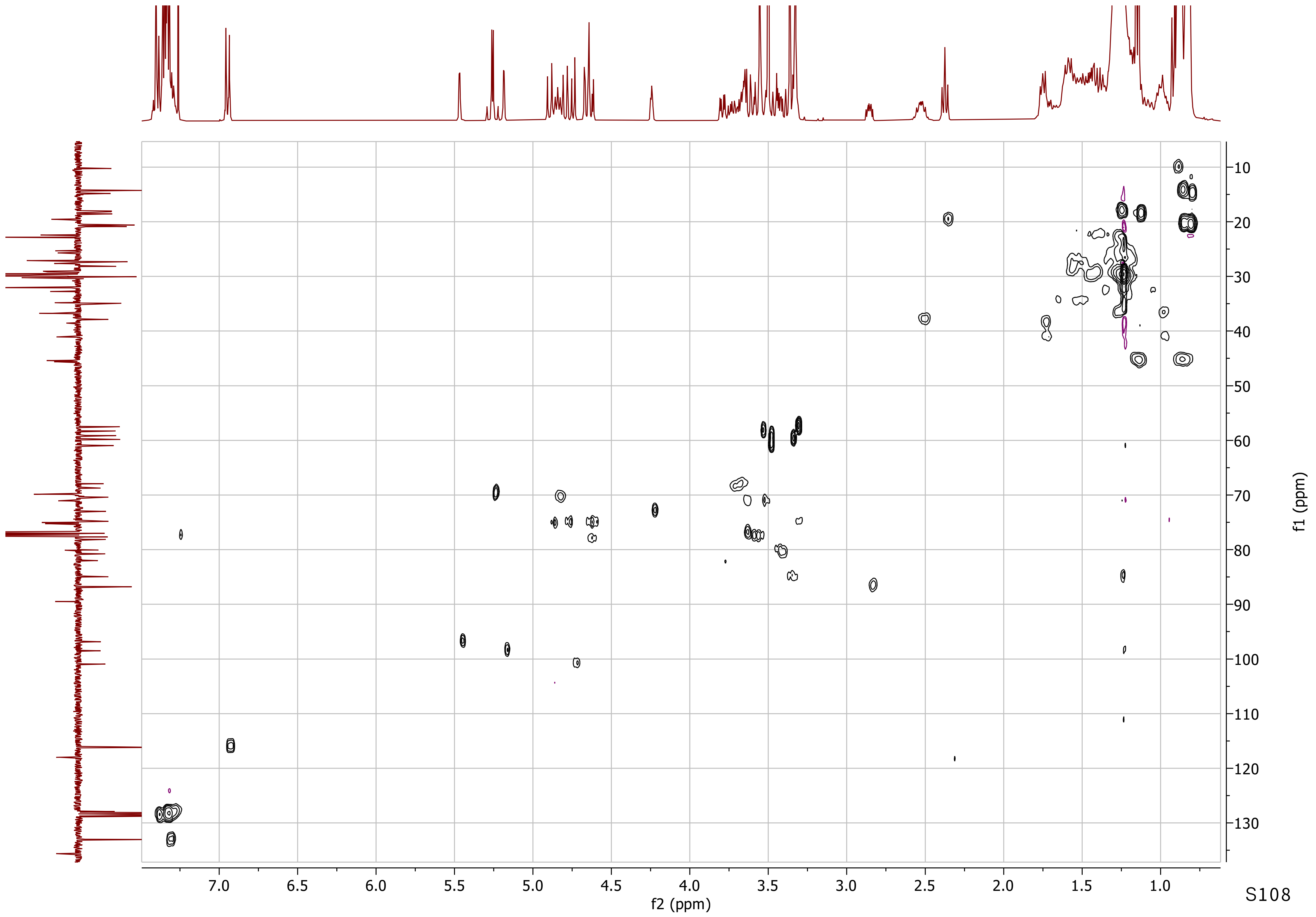

7.0
6.5
6.0
5.5
5.0
4.5
4.0
3.5
3.0
2.5
2.0
1.5
1.0
f2 (ppm)
10
20
30
40
50
60
70
80
90
100
110
120
130
f1 (ppm)

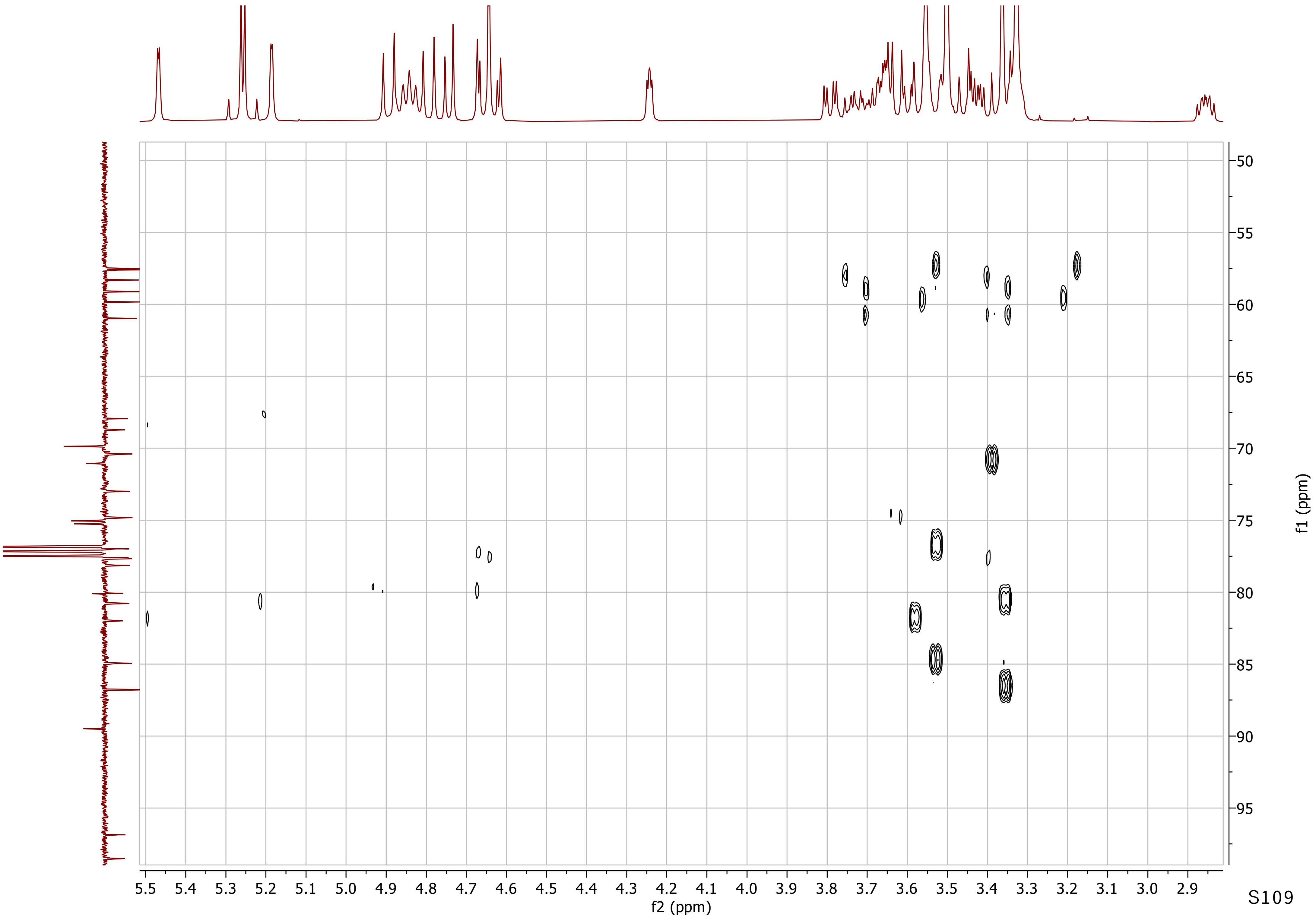

50
55
60
65
70
75
80
85
90
95
f1 (ppm)
5.5 5.4 5.3 5.2 5.1 5.0 4.9 4.8 4.7 4.6 4.5 4.4 4.3 4.2 4.1 4.0 3.9 3.8 3.7 3.6 3.5 3.4 3.3 3.2 3.1 3.0 2.9
f2 (ppm)

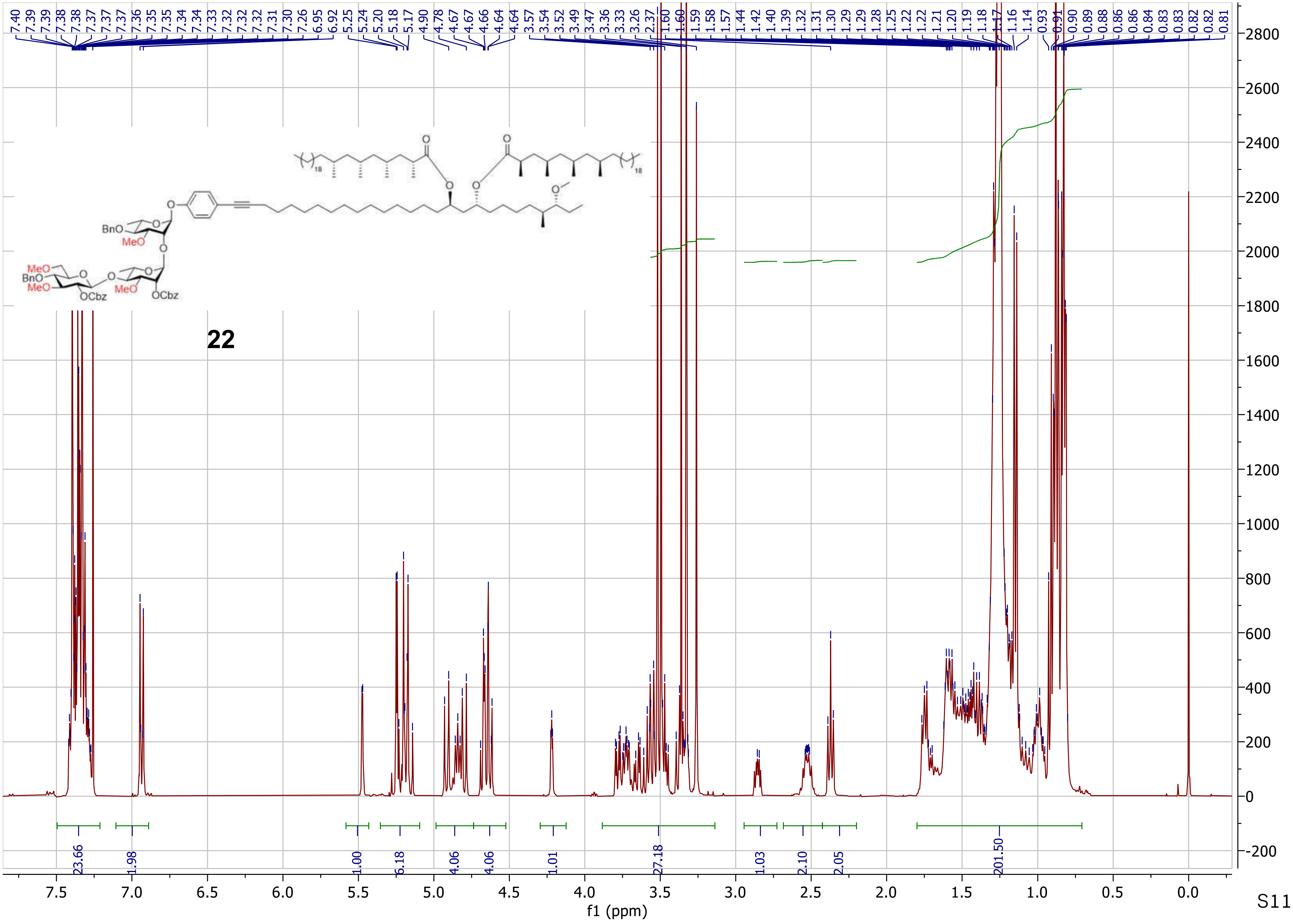
22
BnO
MeO
MeO
BnO
MeO
OCbz
MeO
OCbz
23.66
1.98
1.00
6.18
4.06
4.06
1.01
27.18
1.03
2.10
2.05
201.50
f1 (ppm)

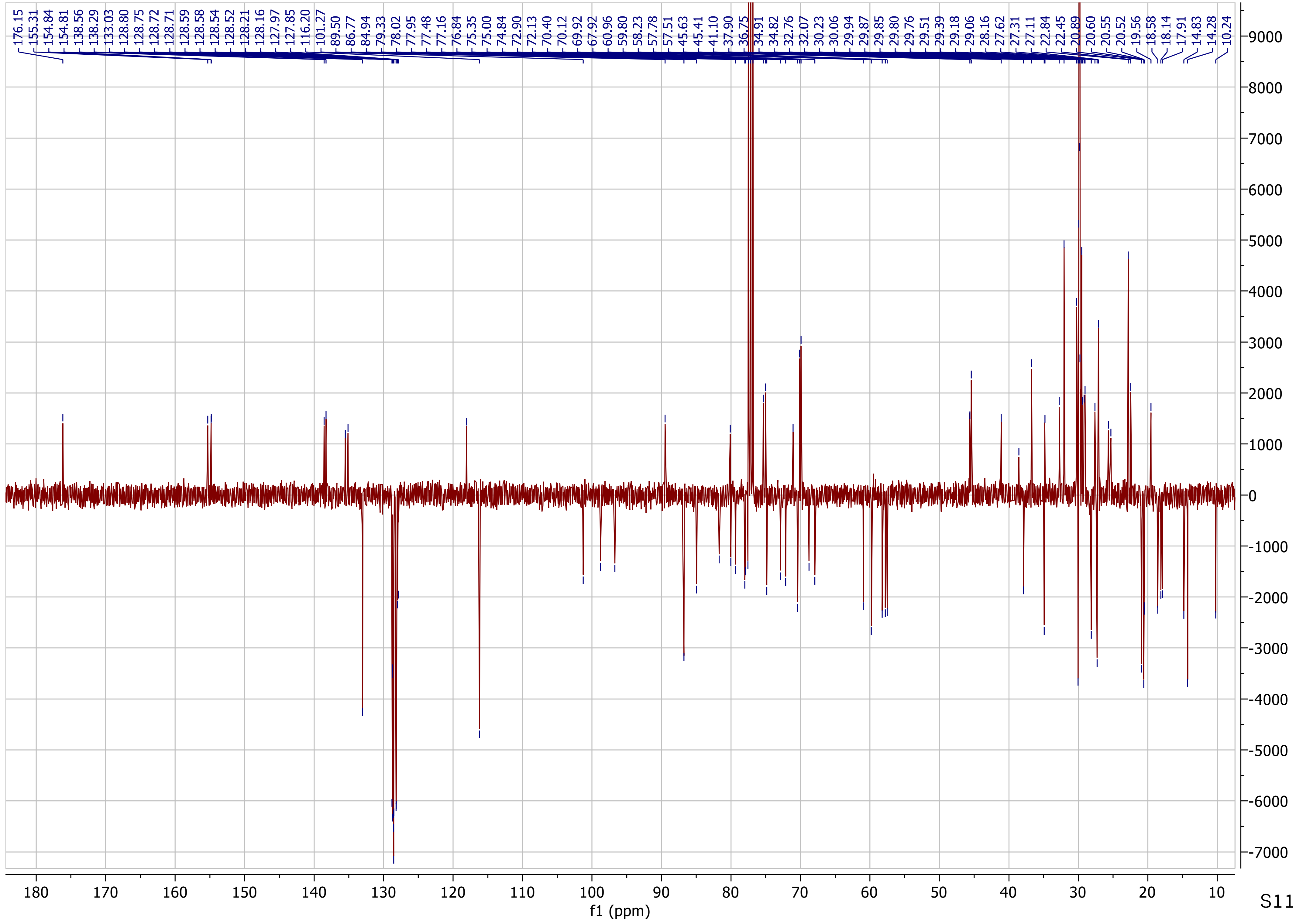
176.15
155.31
154.84
154.81
138.56
138.29
133.03
128.80
128.75
128.72
128.71
128.59
128.58
128.54
128.52
128.21
128.16
127.97
127.85
116.20
101.27
89.50
86.77
84.94
79.33
78.02
77.95
77.48
77.16
76.84
75.35
75.00
74.84
72.90
72.13
70.40
70.12
69.92
67.92
60.96
59.80
58.23
57.78
57.51
45.63
45.41
41.10
37.90
36.75
34.91
34.82
32.76
32.07
30.23
30.06
29.94
29.87
29.85
29.80
29.76
29.51
29.39
29.18
29.06
28.16
27.62
27.31
27.11
22.84
22.45
20.89
20.60
20.55
20.52
19.56
18.58
18.14
17.91
14.83
14.28
10.24
f1 (ppm)

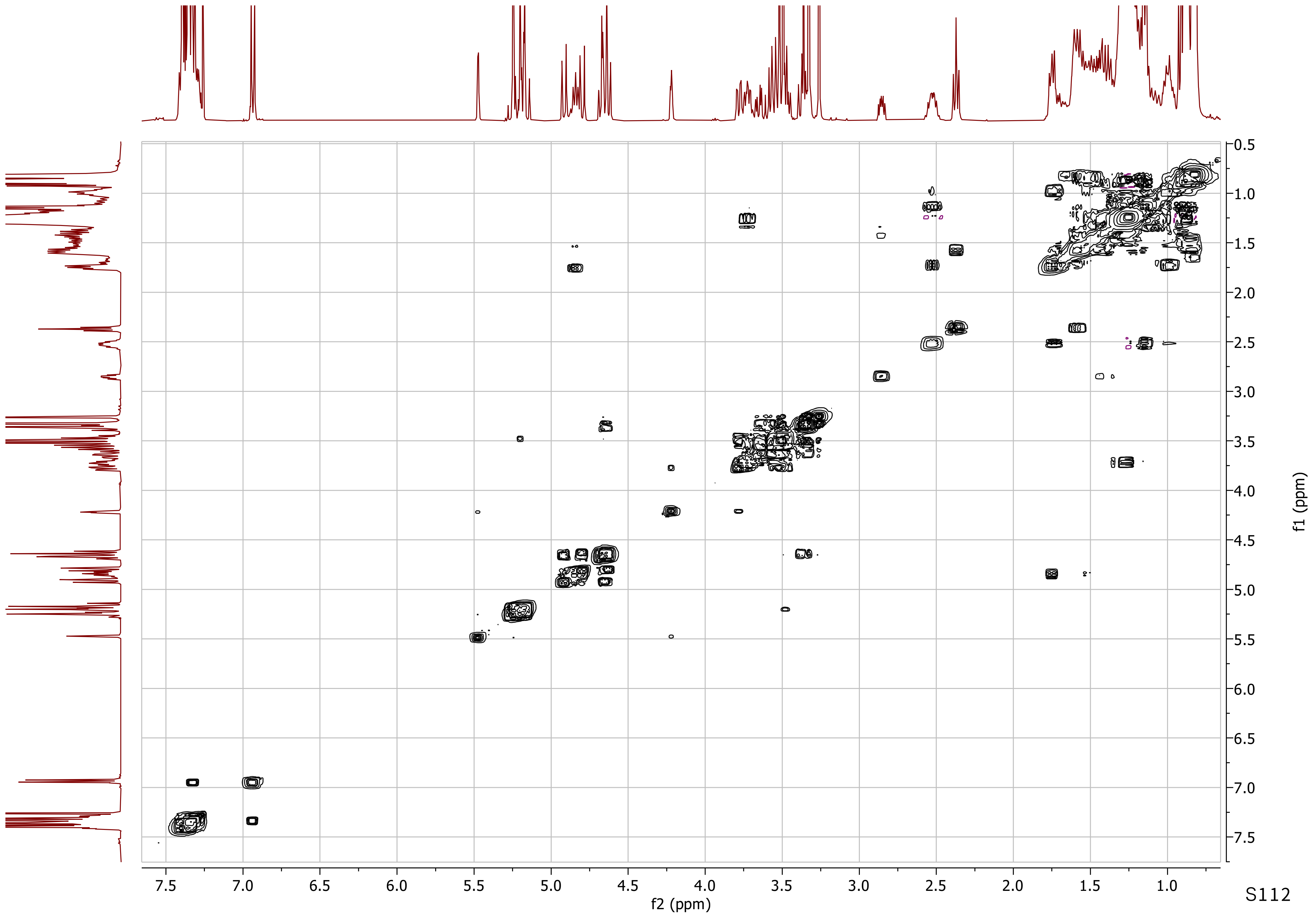
f2 (ppm)
f1 (ppm)

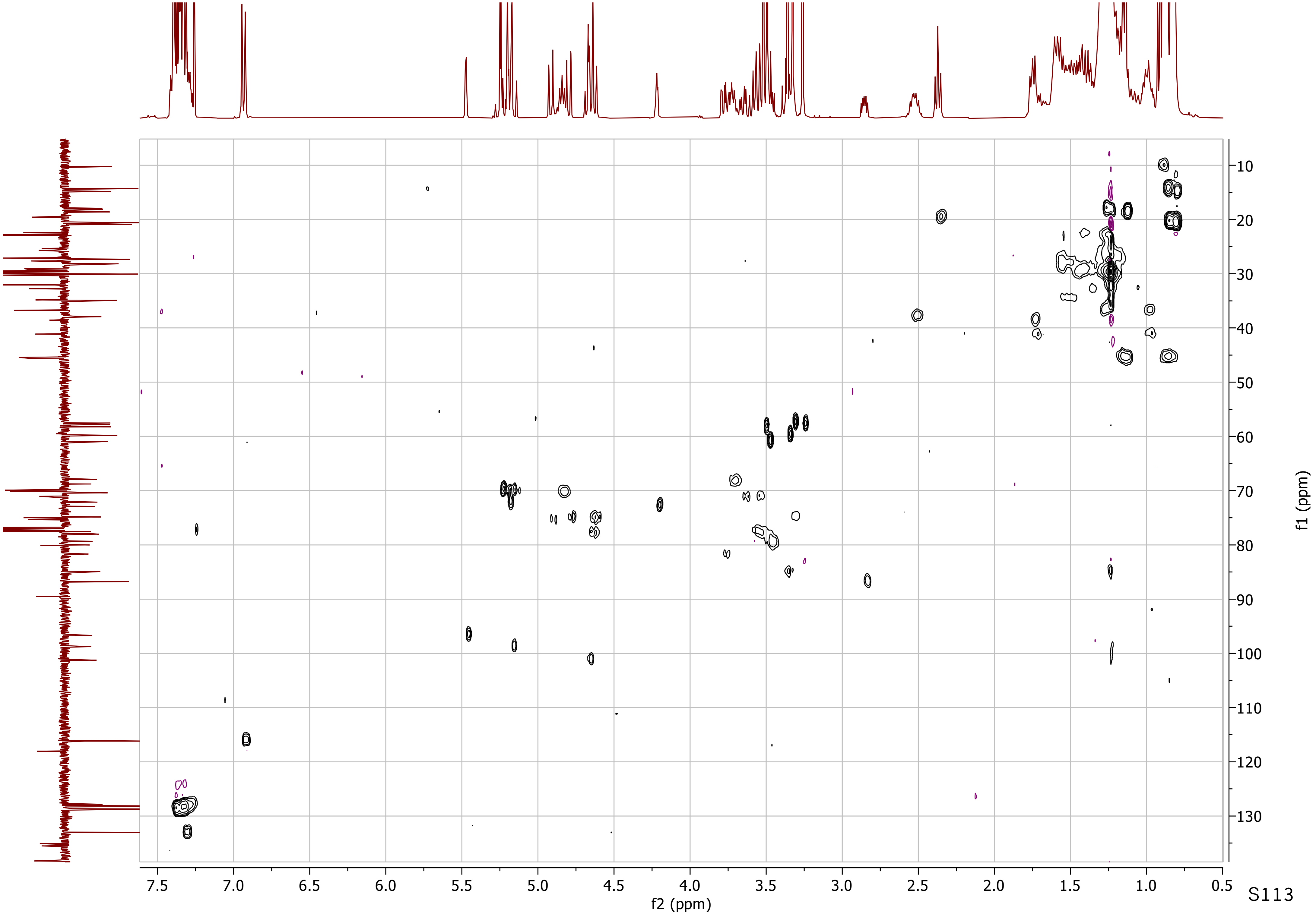

f2 (ppm)
f1 (ppm)

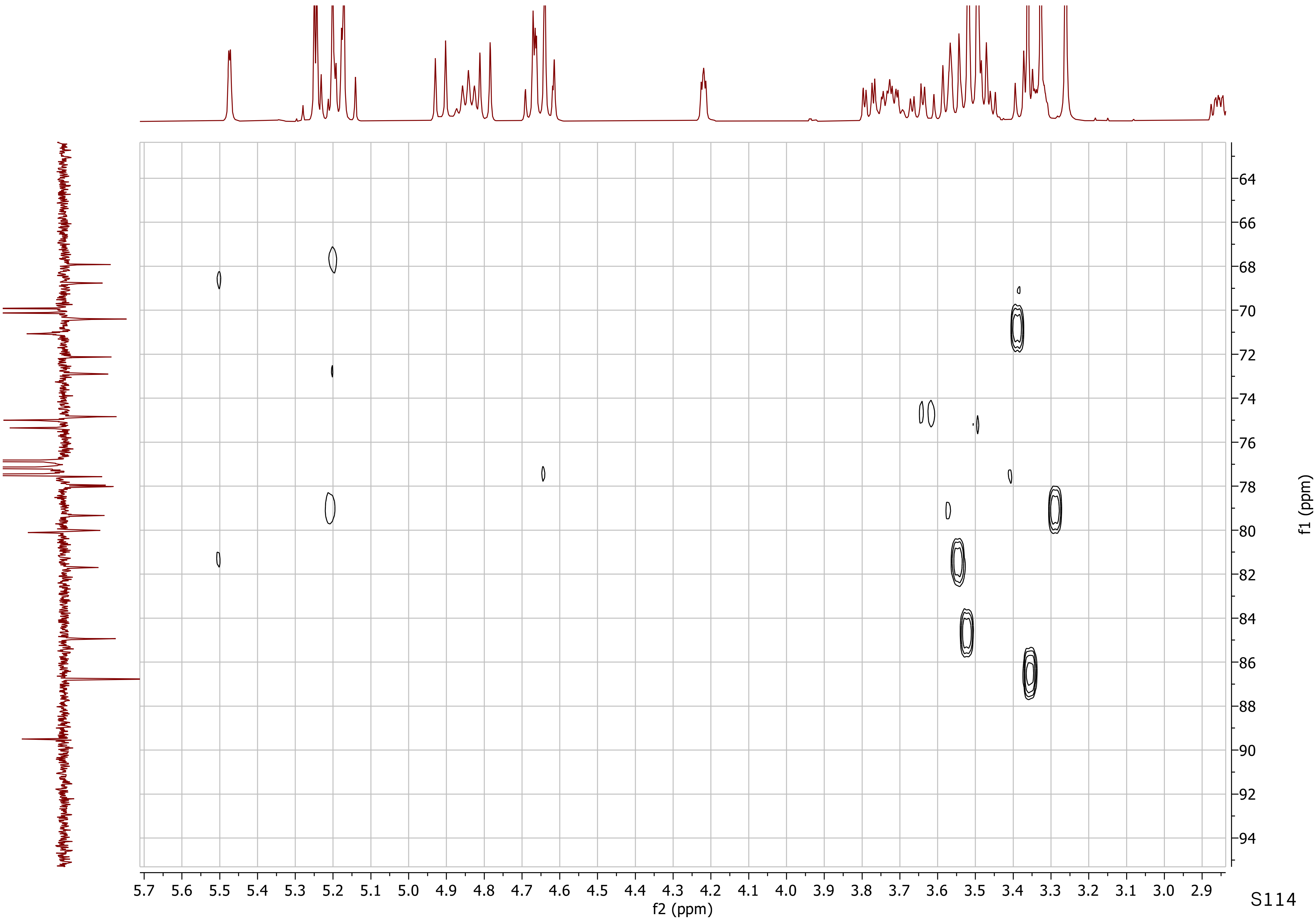

f2 (ppm)
f1 (ppm)

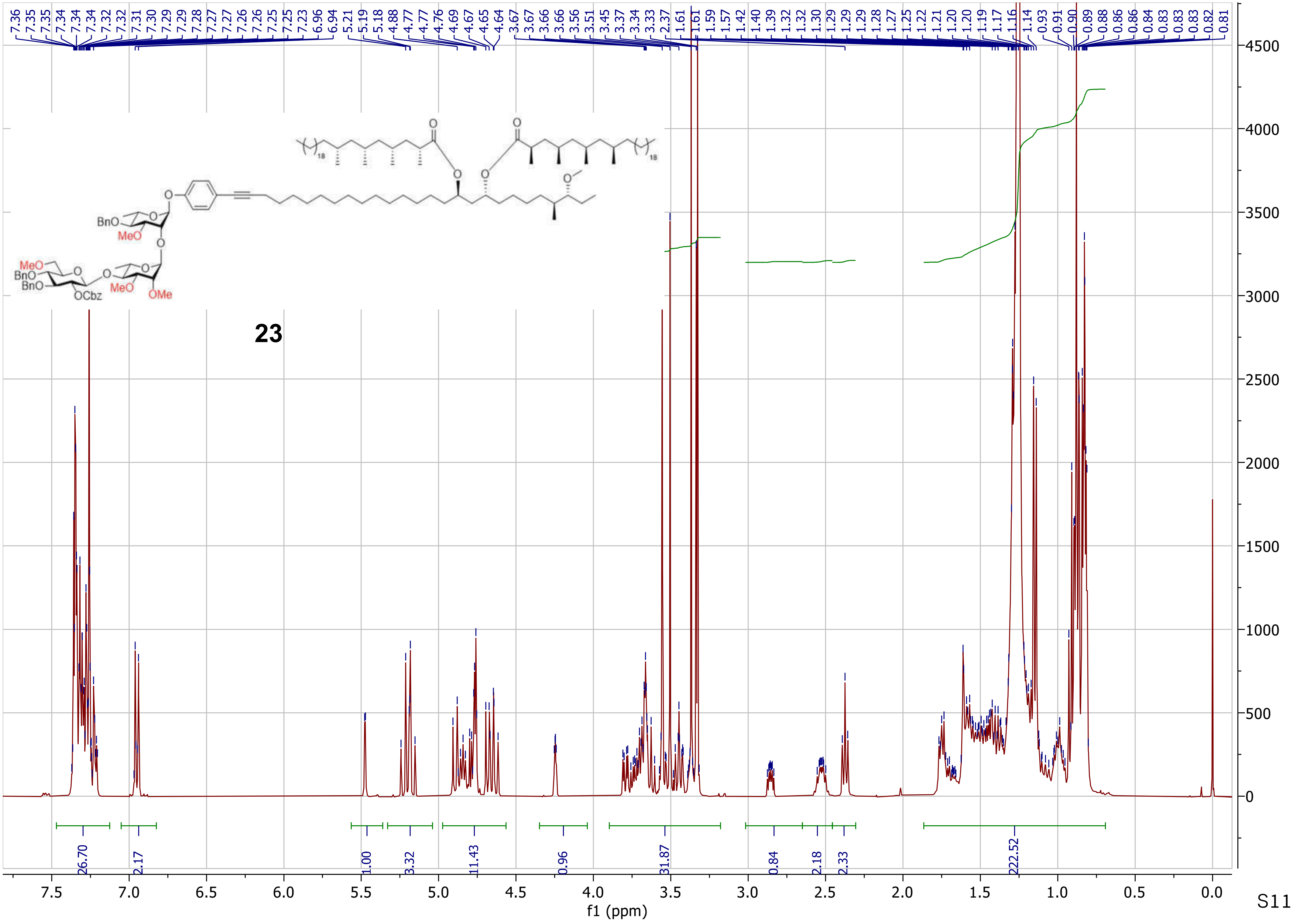
7.36 7.35 7.35 7.34 7.34 7.34 7.32 7.32 7.31 7.30 7.29 7.29 7.28 7.27 7.27 7.26 7.26 7.25 7.25 7.23 6.96 6.94 5.21 5.19 5.18 4.88 4.77 4.77 4.76 4.69 4.67 4.65 4.64 3.67 3.67 3.66 3.66 3.56 3.51 3.45 3.37 3.34 3.33 2.37 1.61 1.61 1.59 1.57 1.42 1.40 1.39 1.32 1.32 1.30 1.29 1.29 1.29 1.28 1.27 1.25 1.22 1.21 1.20 1.20 1.19 1.17 1.16 1.14 0.93 0.91 0.90 0.89 0.88 0.86 0.84 0.83 0.83 0.83 0.82 0.81
BnO
MeO
OCbz
OMe
23
26.70
2.17
1.00
3.32
11.43
0.96
31.87
0.84
2.18
2.33
222.52
f1 (ppm)

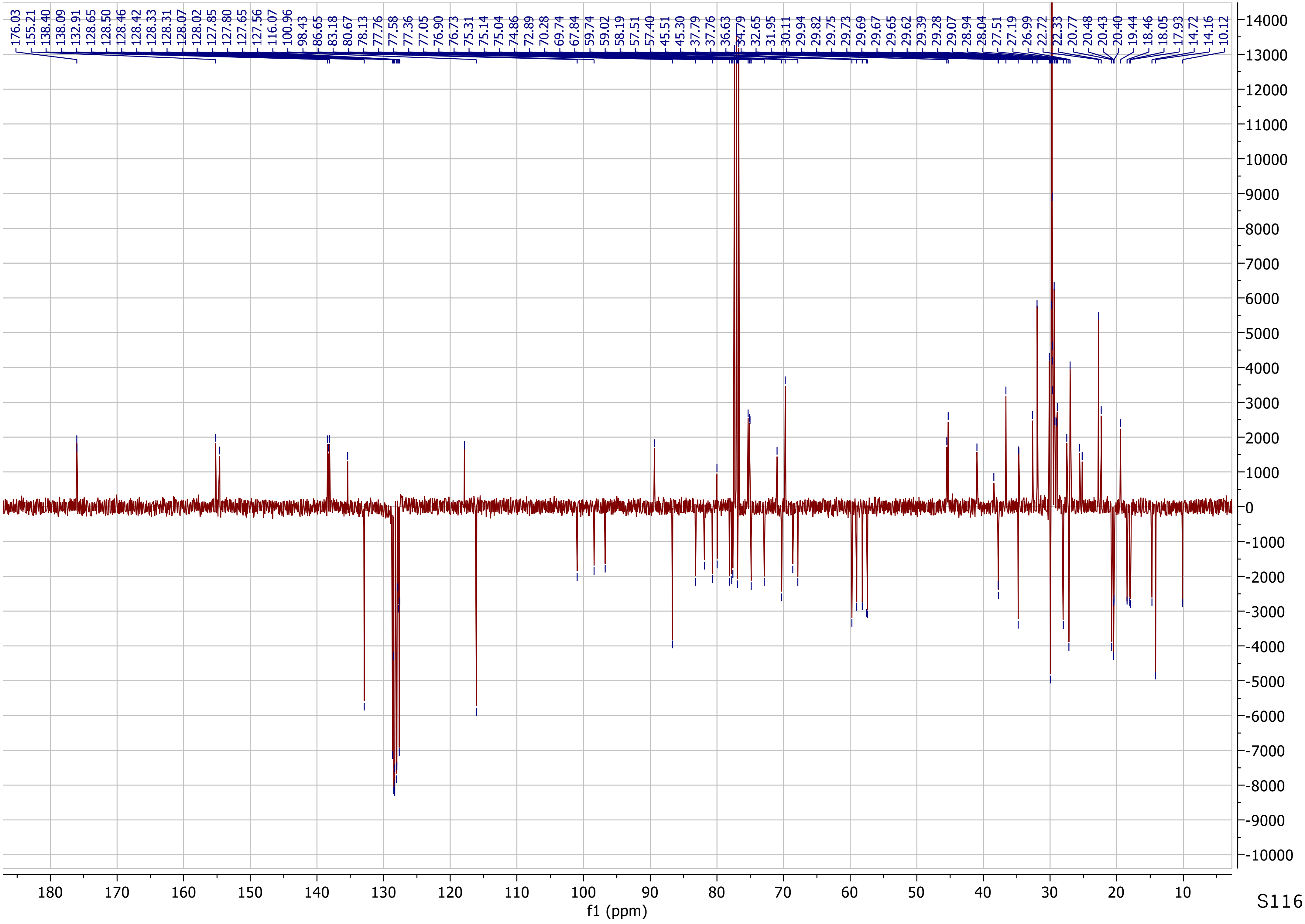
176.03
155.21
138.40
138.09
132.91
128.65
128.50
128.46
128.42
128.33
128.31
128.07
128.02
127.85
127.80
127.65
127.56
116.07
100.96
98.43
86.65
83.18
80.67
78.13
77.76
77.58
77.36
77.05
76.90
76.73
75.31
75.14
75.04
74.86
72.89
70.28
69.74
67.84
59.74
59.02
58.19
57.51
57.40
45.51
45.30
37.79
37.76
36.63
34.79
32.65
31.95
30.11
29.94
29.82
29.75
29.73
29.69
29.67
29.65
29.62
29.39
29.28
29.07
28.94
28.04
27.51
27.19
26.99
22.72
22.33
20.77
20.48
20.43
20.40
19.44
18.46
18.05
17.93
14.72
14.16
10.12
f1 (ppm)

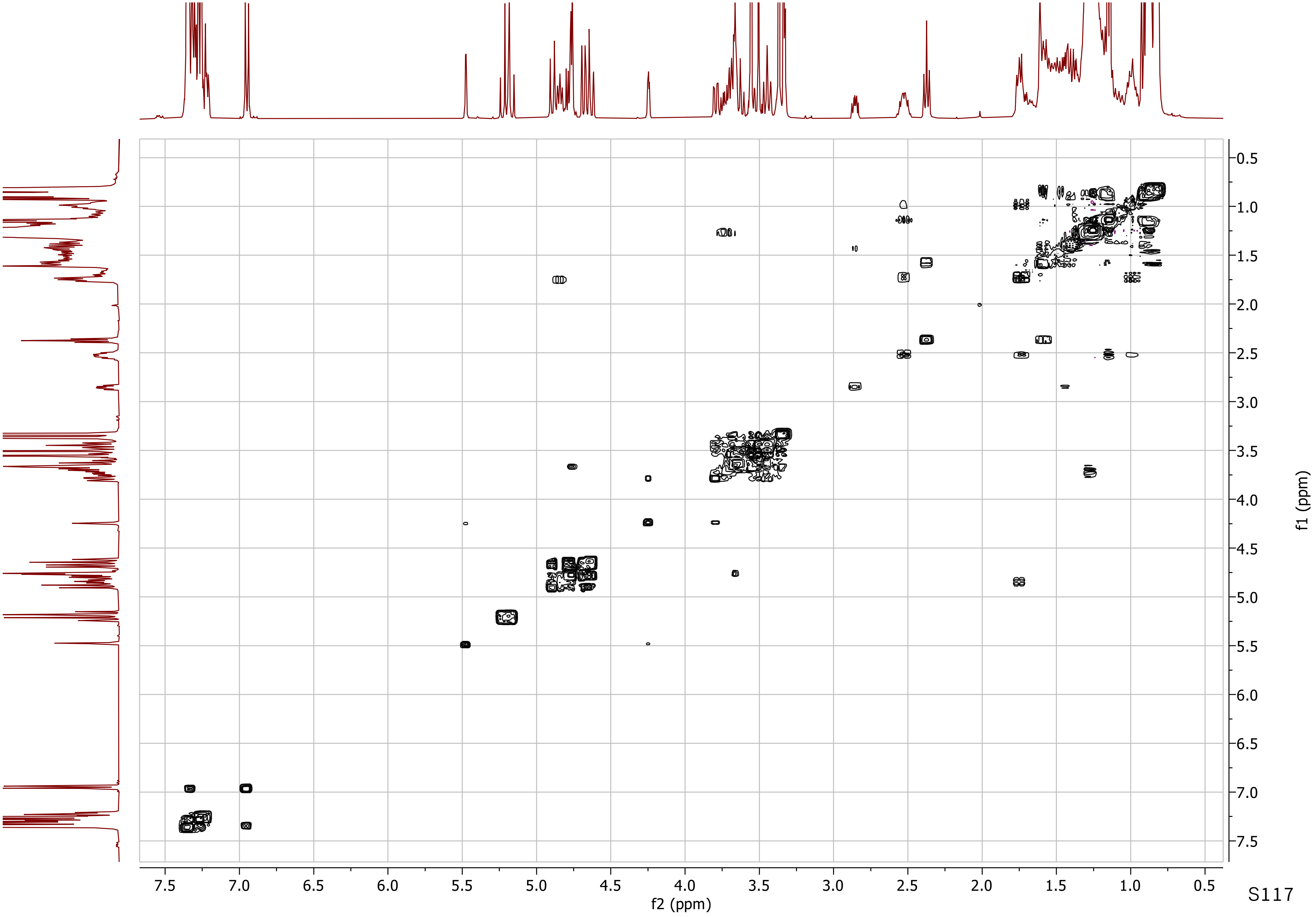

f2 (ppm)
f1 (ppm)

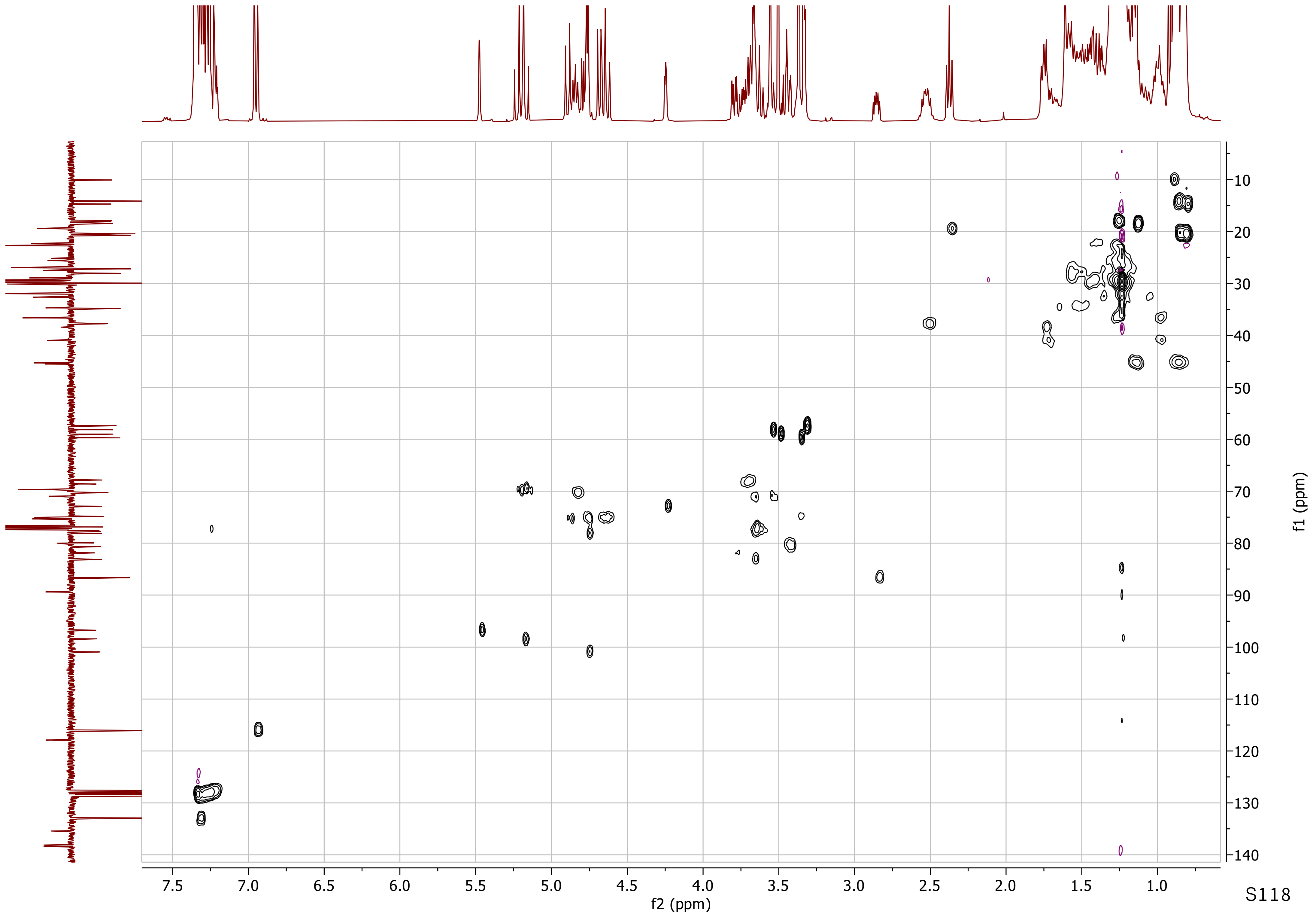

7.5
7.0
6.5
6.0
5.5
5.0
4.5
4.0
3.5
3.0
2.5
2.0
1.5
1.0
f2 (ppm)
10
20
30
40
50
60
70
80
90
100
110
120
130
140
f1 (ppm)

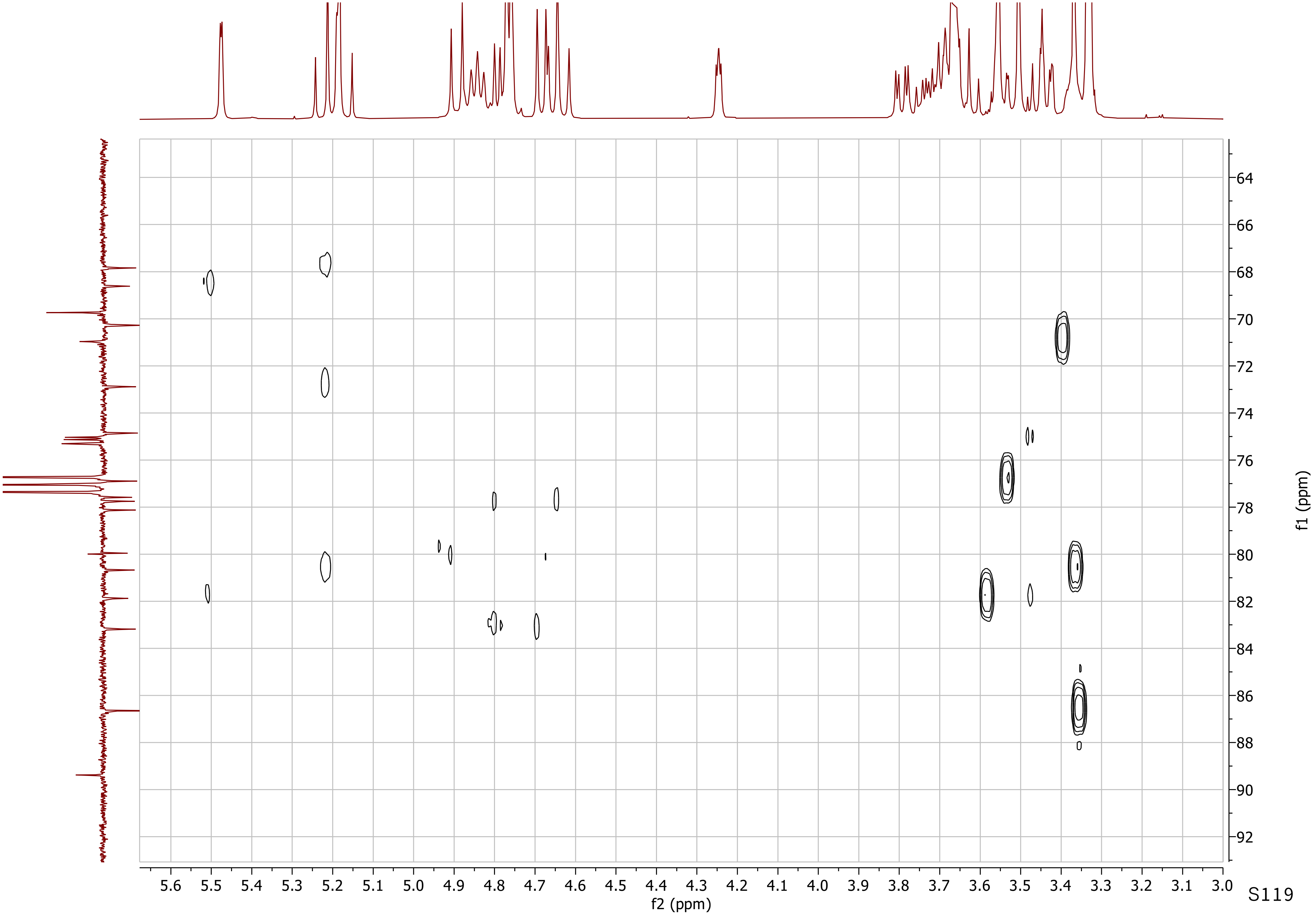

64
66
68
70
72
74
76
78
80
82
84
86
88
90
92
f1 (ppm)
5.6 5.5 5.4 5.3 5.2 5.1 5.0 4.9 4.8 4.7 4.6 4.5 4.4 4.3 4.2 4.1 4.0 3.9 3.8 3.7 3.6 3.5 3.4 3.3 3.2 3.1 3.0
f2 (ppm)

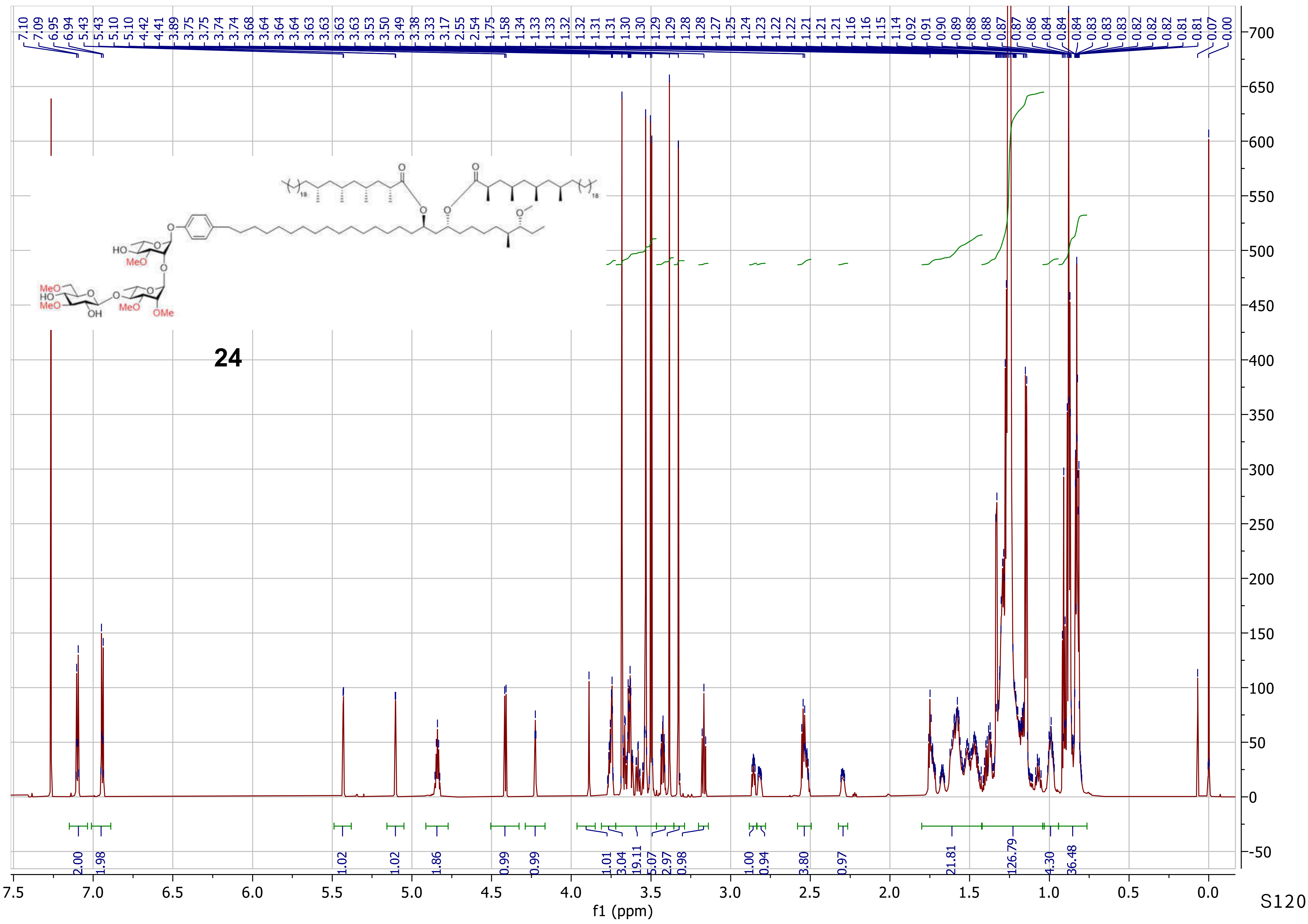
24
f1 (ppm)

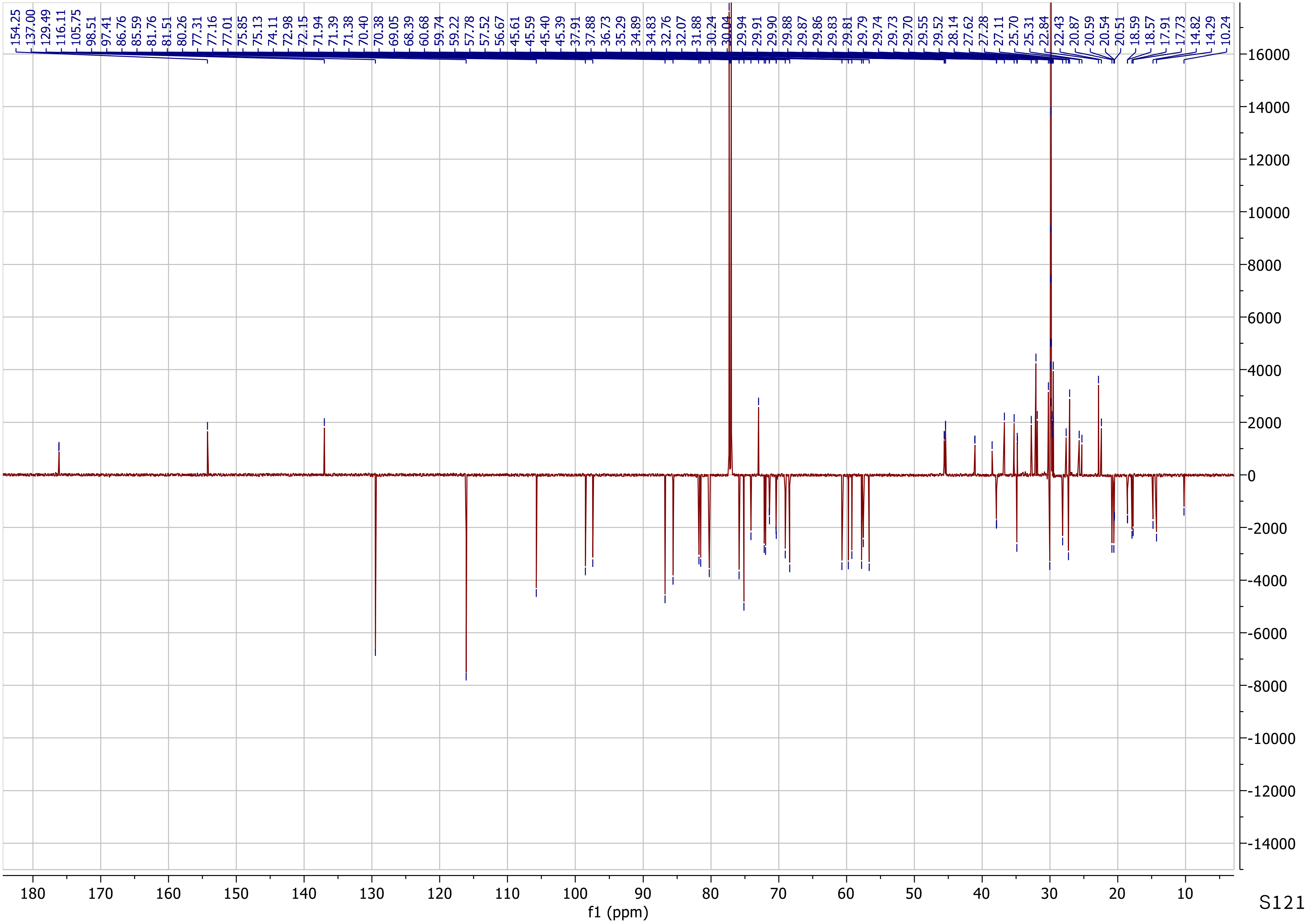
154.25
137.00
129.49
116.11
105.75
98.51
97.41
86.76
85.59
81.76
81.51
80.26
77.31
77.16
77.01
75.85
75.13
74.11
72.98
72.15
71.94
71.39
71.38
70.40
70.38
69.05
68.39
60.68
59.74
59.22
57.78
57.52
56.67
45.61
45.59
45.40
45.39
37.91
37.88
36.73
35.29
34.89
34.83
32.76
32.07
31.88
30.24
30.04
29.94
29.91
29.90
29.88
29.87
29.86
29.83
29.81
29.79
29.74
29.73
29.70
29.55
29.52
28.14
27.62
27.28
27.11
25.70
25.31
22.84
22.43
20.87
20.59
20.54
20.51
18.59
18.57
17.91
17.73
14.82
14.29
10.24
16000
14000
12000
10000
8000
6000
4000
2000
0
-2000
-4000
-6000
-8000
-10000
-12000
-14000
180 170 160 150 140 130 120 110 100 90 80 70 60 50 40 30 20 10
f1 (ppm)

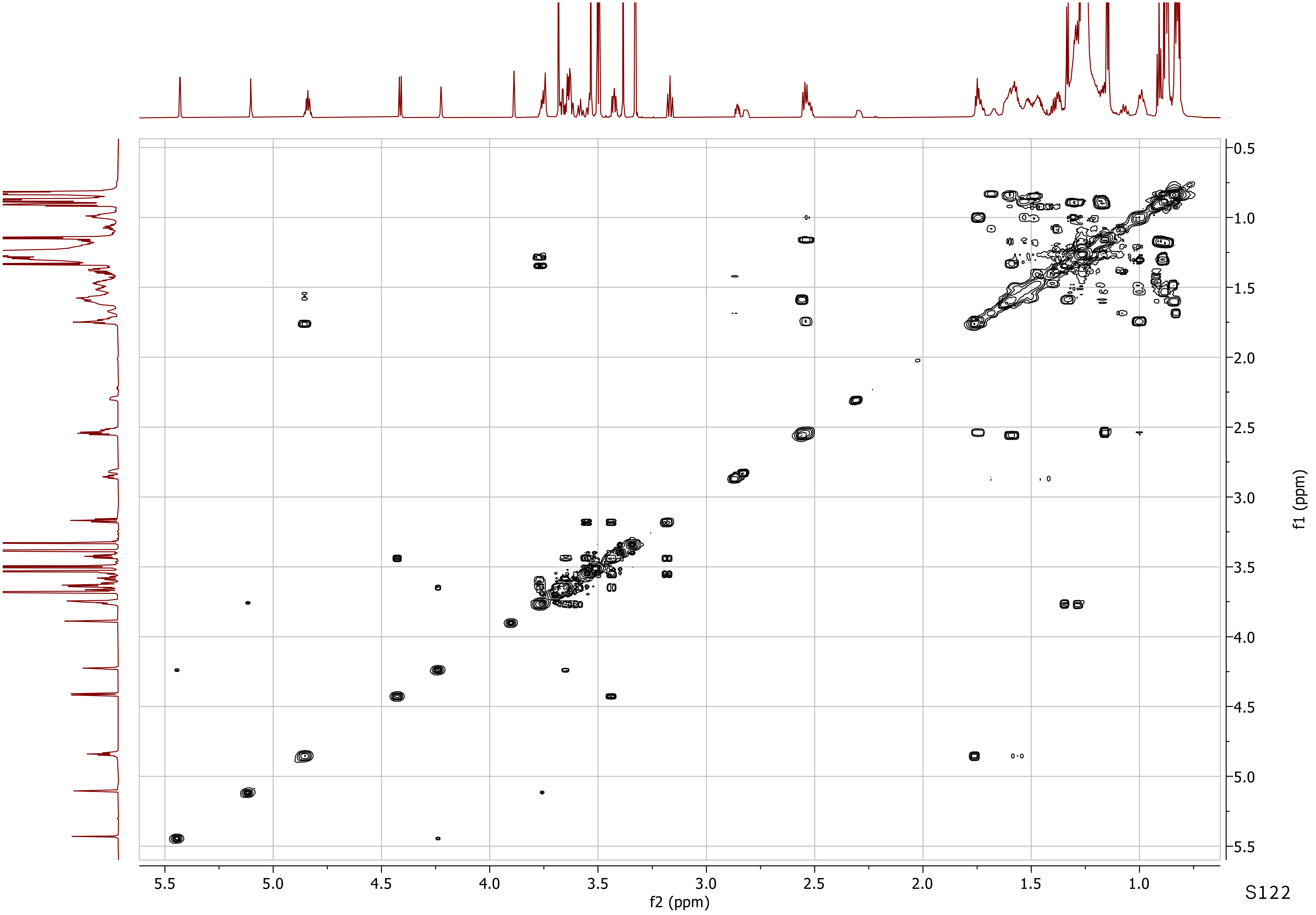

5.5
5.0
4.5
4.0
3.5
3.0
2.5
2.0
1.5
1.0
f2 (ppm)
0.5
1.0
1.5
2.0
2.5
3.0
3.5
4.0
4.5
5.0
5.5
f1 (ppm)

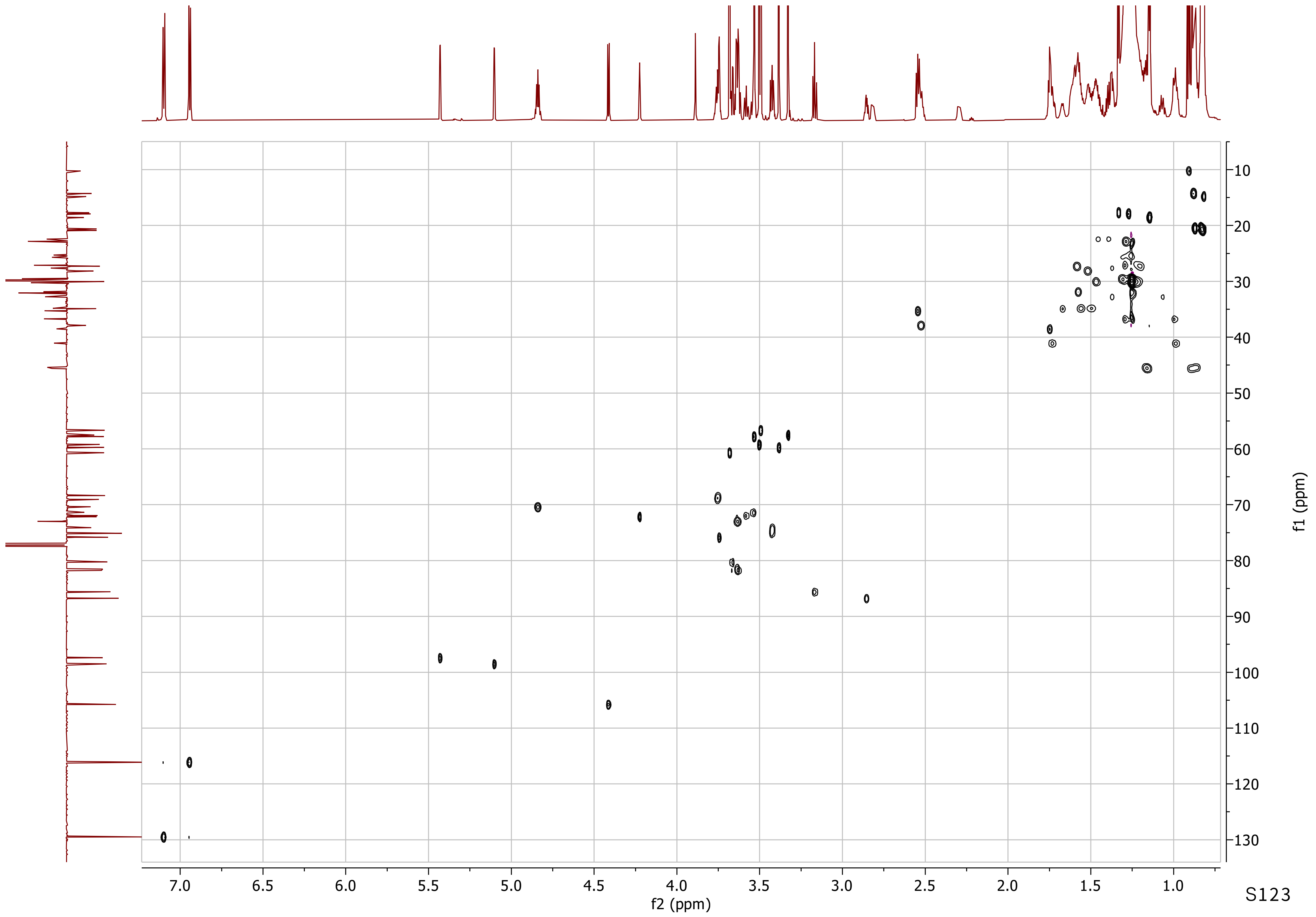
f2 (ppm)
f1 (ppm)

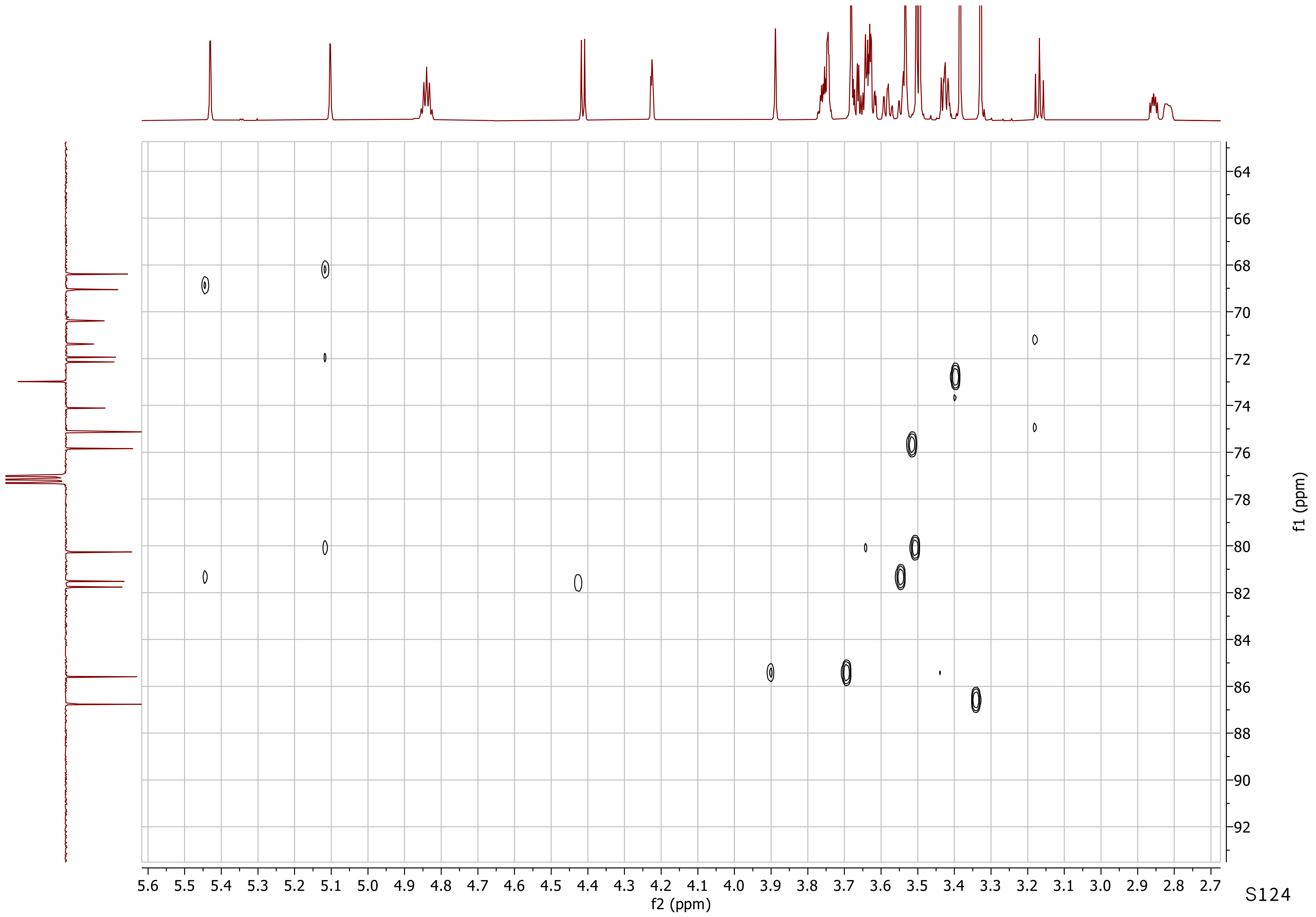
f2 (ppm)
f1 (ppm)

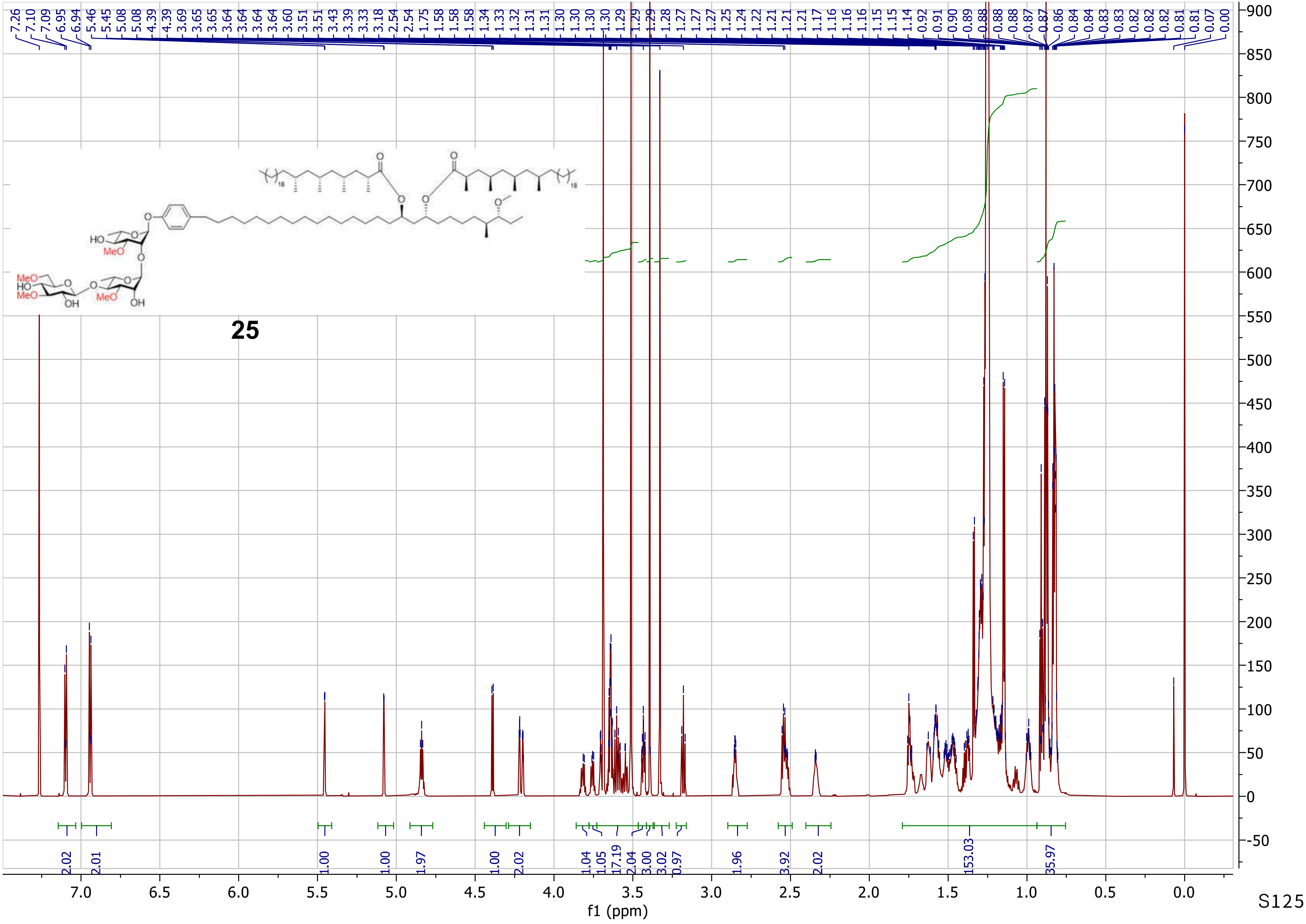

25

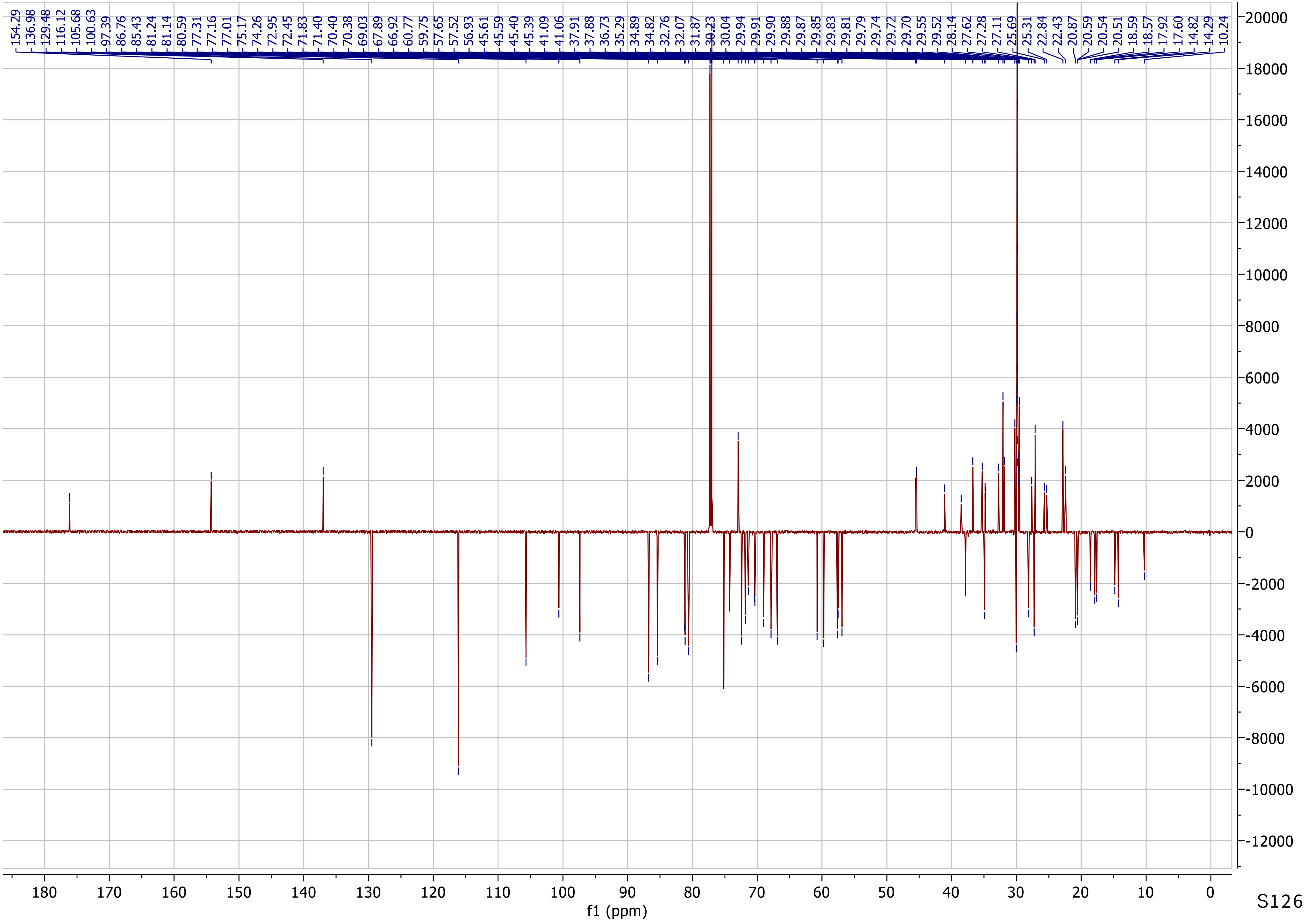

154.29
136.98
129.48
116.12
105.68
100.63
97.39
86.76
85.43
81.24
81.14
80.59
77.31
77.16
77.01
75.17
74.26
72.95
72.45
71.83
71.40
70.40
70.38
69.03
67.89
66.92
60.77
59.75
57.65
57.52
56.93
45.61
45.59
45.40
45.39
41.09
41.06
37.91
37.88
36.73
35.29
34.89
34.82
32.76
32.07
31.87
30.23
30.04
29.94
29.91
29.90
29.88
29.87
29.85
29.83
29.81
29.79
29.74
29.72
29.70
29.55
29.52
28.14
27.62
27.28
27.11
25.69
25.31
22.84
22.43
20.87
20.59
20.54
20.51
18.59
18.57
17.92
17.60
14.82
14.29
10.24
f1 (ppm)

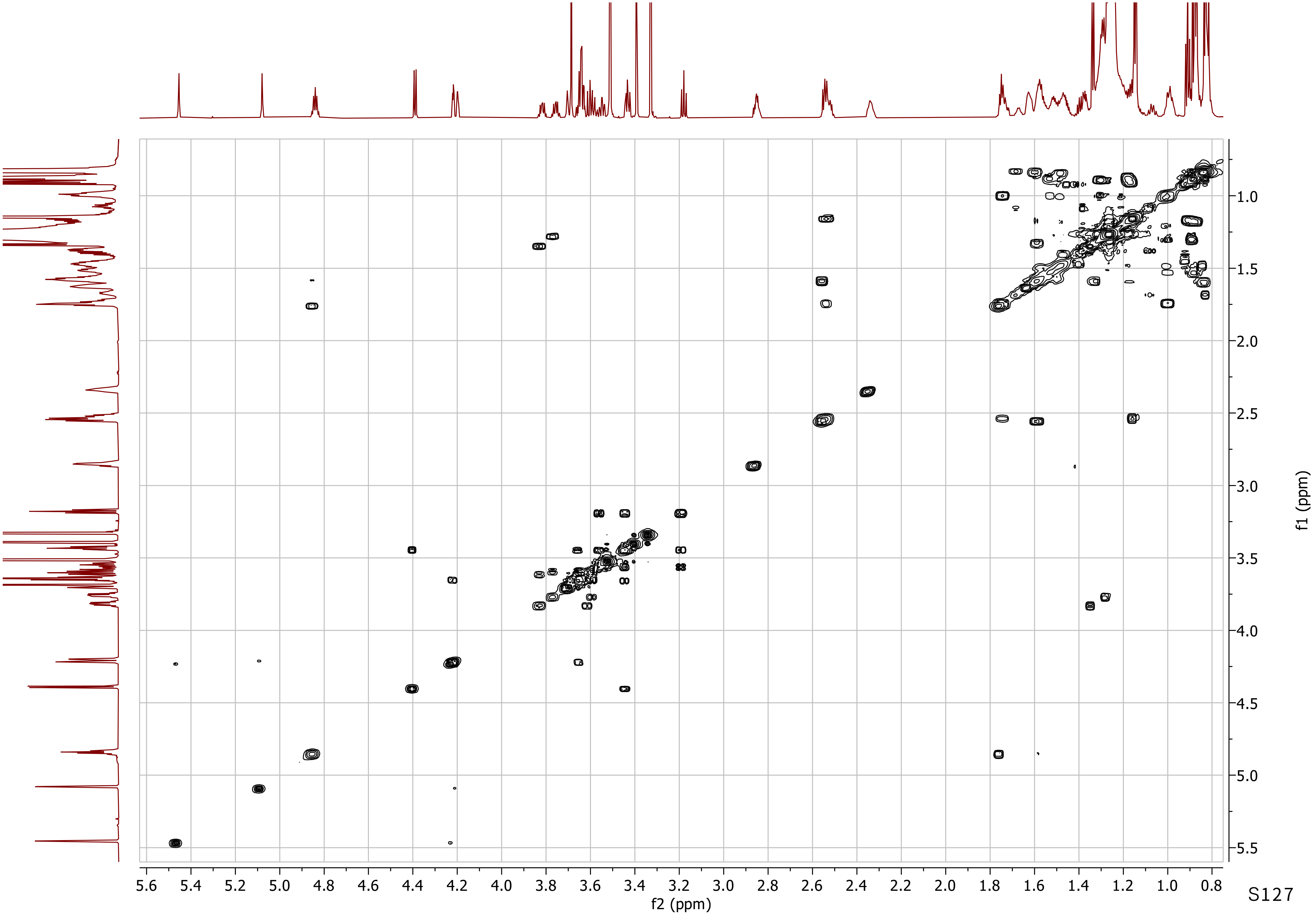

5.6
5.4
5.2
5.0
4.8
4.6
4.4
4.2
4.0
3.8
3.6
3.4
3.2
3.0
2.8
2.6
2.4
2.2
2.0
1.8
1.6
1.4
1.2
1.0
0.8
f2 (ppm)
1.0
1.5
2.0
2.5
3.0
3.5
4.0
4.5
5.0
5.5
f1 (ppm)

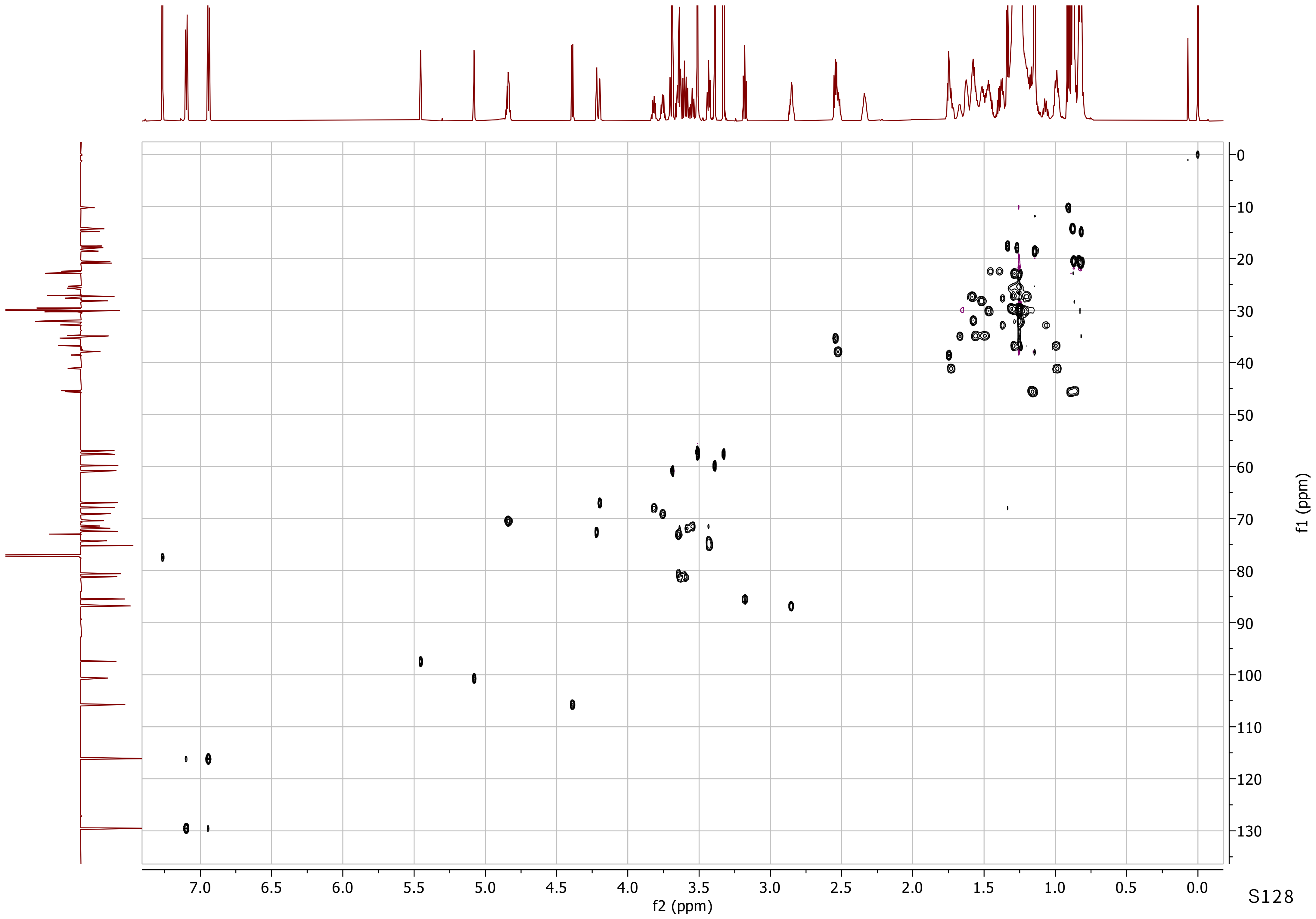

f2 (ppm)
f1 (ppm)

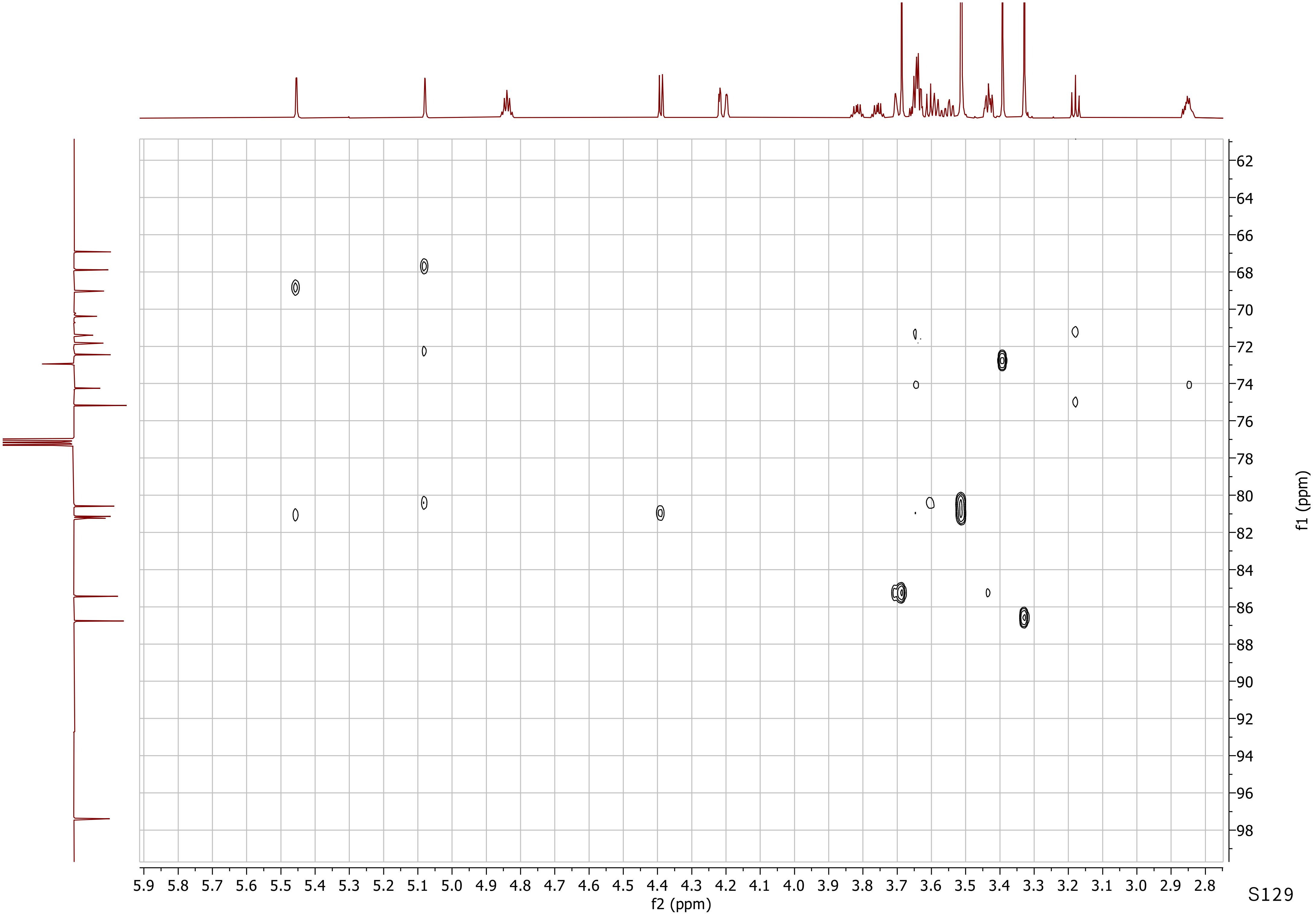

5.9 5.8 5.7 5.6 5.5 5.4 5.3 5.2 5.1 5.0 4.9 4.8 4.7 4.6 4.5 4.4 4.3 4.2 4.1 4.0 3.9 3.8 3.7 3.6 3.5 3.4 3.3 3.2 3.1 3.0 2.9 2.8
f2 (ppm)
62 64 66 68 70 72 74 76 78 80 82 84 86 88 90 92 94 96 98
f1 (ppm)

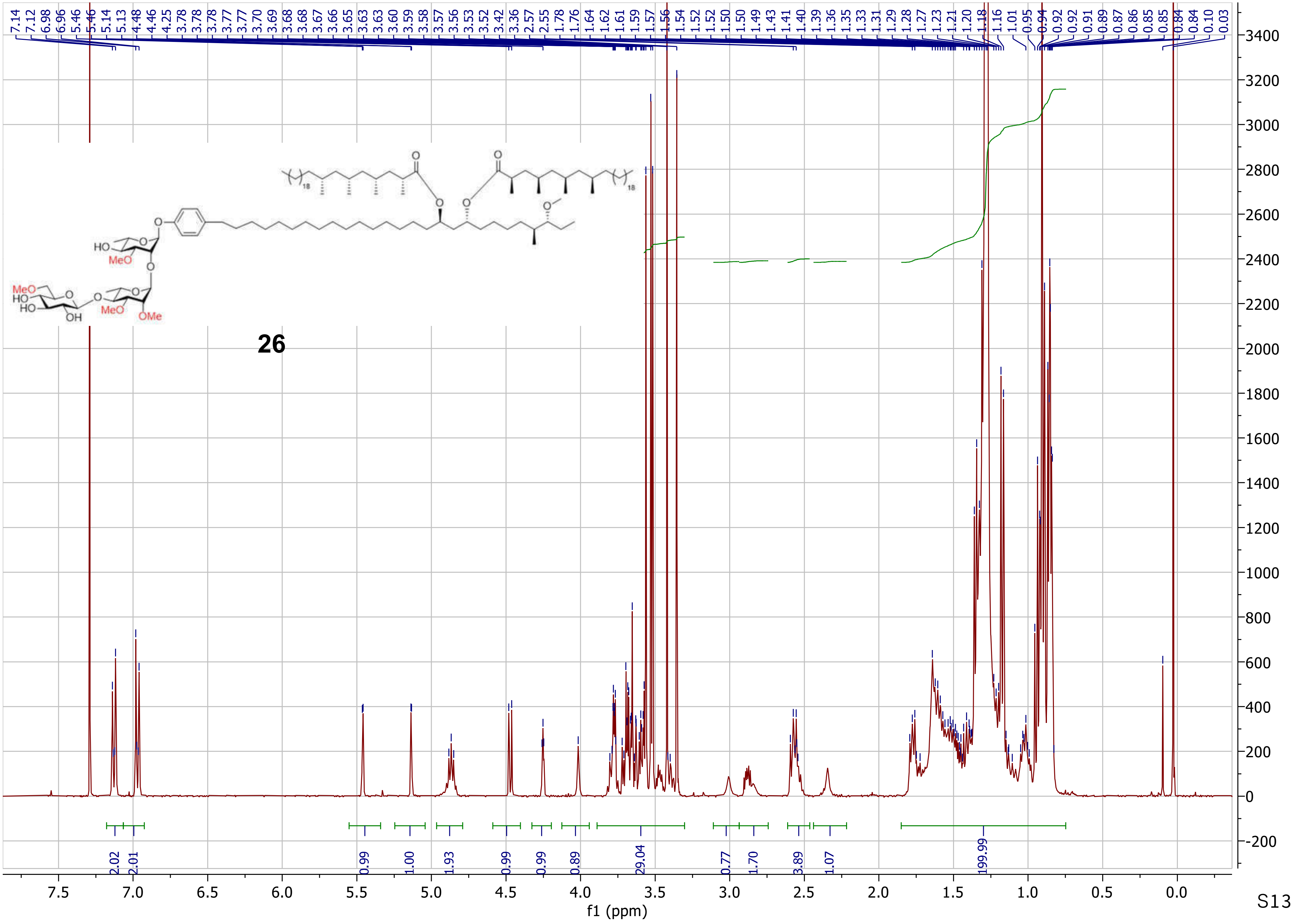
7.14 7.12 6.98 6.96 5.46 5.46 5.14 5.13 4.48 4.46 4.25 3.78 3.78 3.78 3.77 3.77 3.70 3.69 3.68 3.68 3.67 3.66 3.65 3.63 3.63 3.60 3.59 3.58 3.57 3.56 3.53 3.52 3.42 3.36 2.57 2.55 1.78 1.76 1.64 1.62 1.61 1.59 1.57 1.56 1.54 1.52 1.52 1.50 1.50 1.49 1.43 1.41 1.40 1.39 1.36 1.35 1.33 1.31 1.29 1.28 1.27 1.23 1.21 1.20 1.18 1.16 1.01 0.95 0.94 0.92 0.92 0.91 0.89 0.87 0.86 0.85 0.85 0.84 0.84 0.10 0.03
26
2.02 2.01 0.99 1.00 1.93 0.99 0.99 0.89 29.04 0.77 1.70 3.89 1.07 199.99
f1 (ppm)

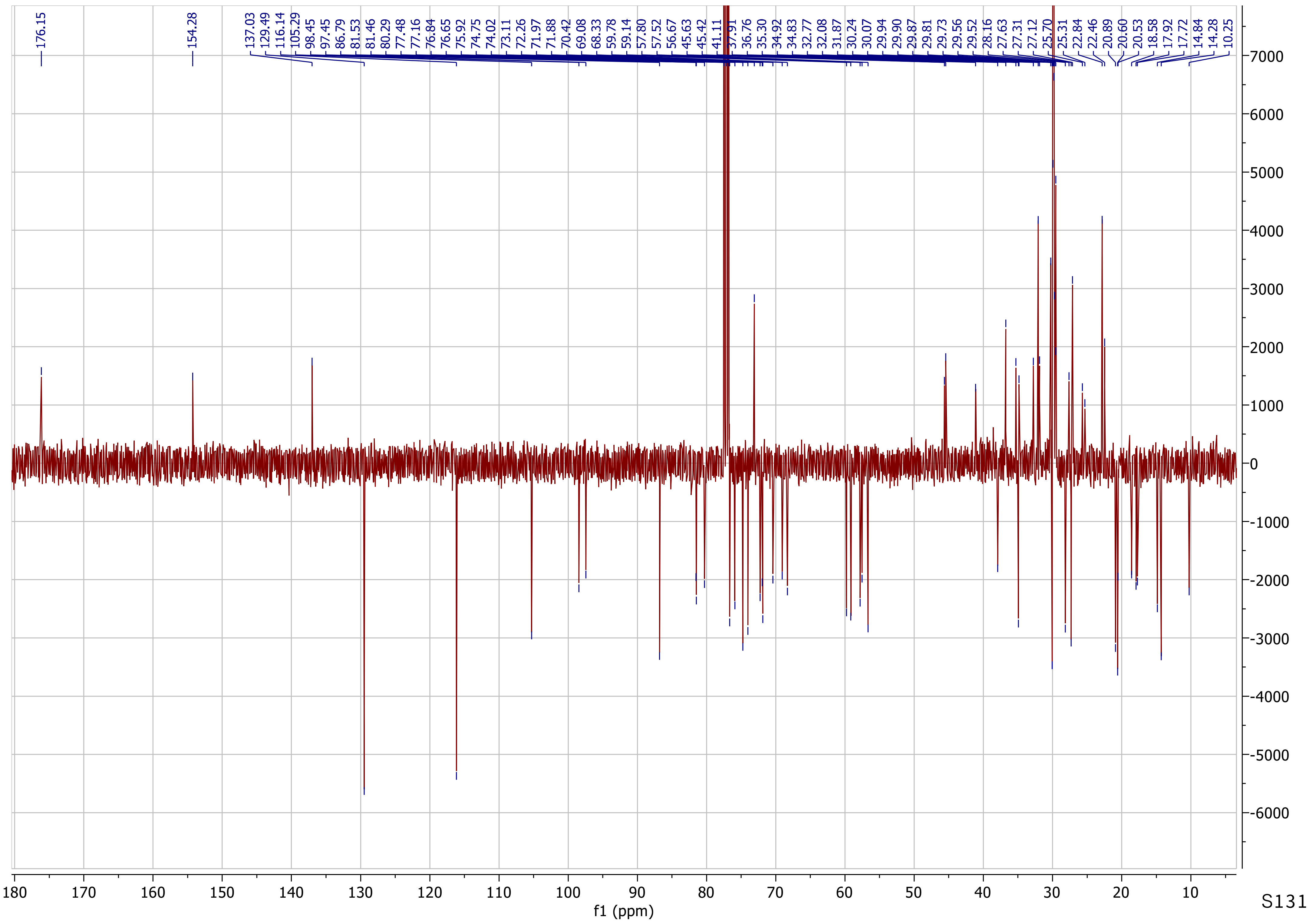
176.15
154.28
137.03
129.49
116.14
105.29
98.45
97.45
86.79
81.53
81.46
80.29
77.48
77.16
76.84
76.65
75.92
74.75
74.02
73.11
72.26
71.97
71.88
70.42
69.08
68.33
59.78
59.14
57.80
57.52
56.67
45.63
45.42
41.11
37.91
36.76
35.30
34.92
34.83
32.77
32.08
31.87
30.24
30.07
29.94
29.90
29.87
29.81
29.73
29.56
29.52
28.16
27.63
27.31
27.12
25.70
25.31
22.84
22.46
20.89
20.60
20.53
18.58
17.92
17.72
14.84
14.28
10.25
f1 (ppm)

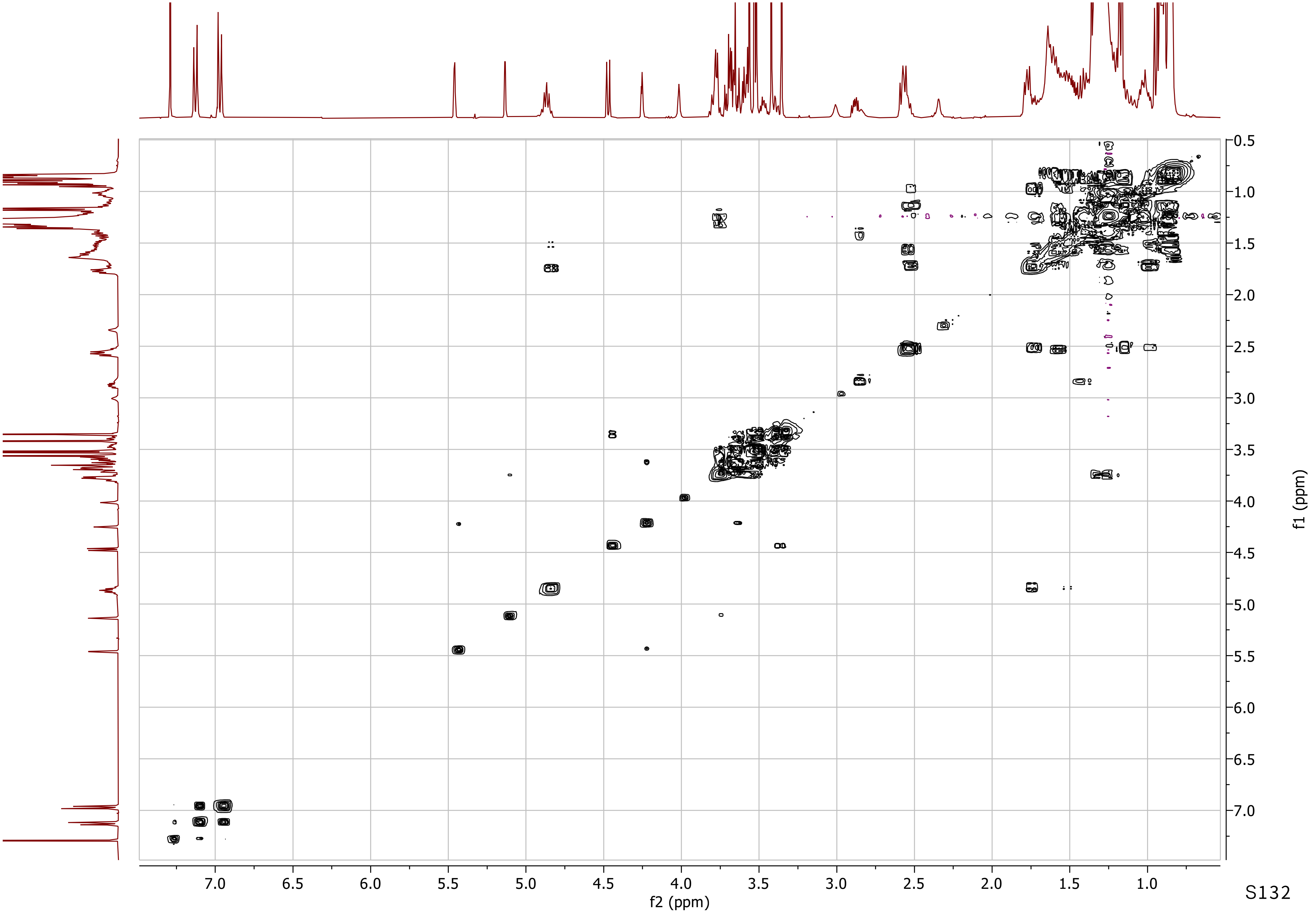
f2 (ppm)
f1 (ppm)

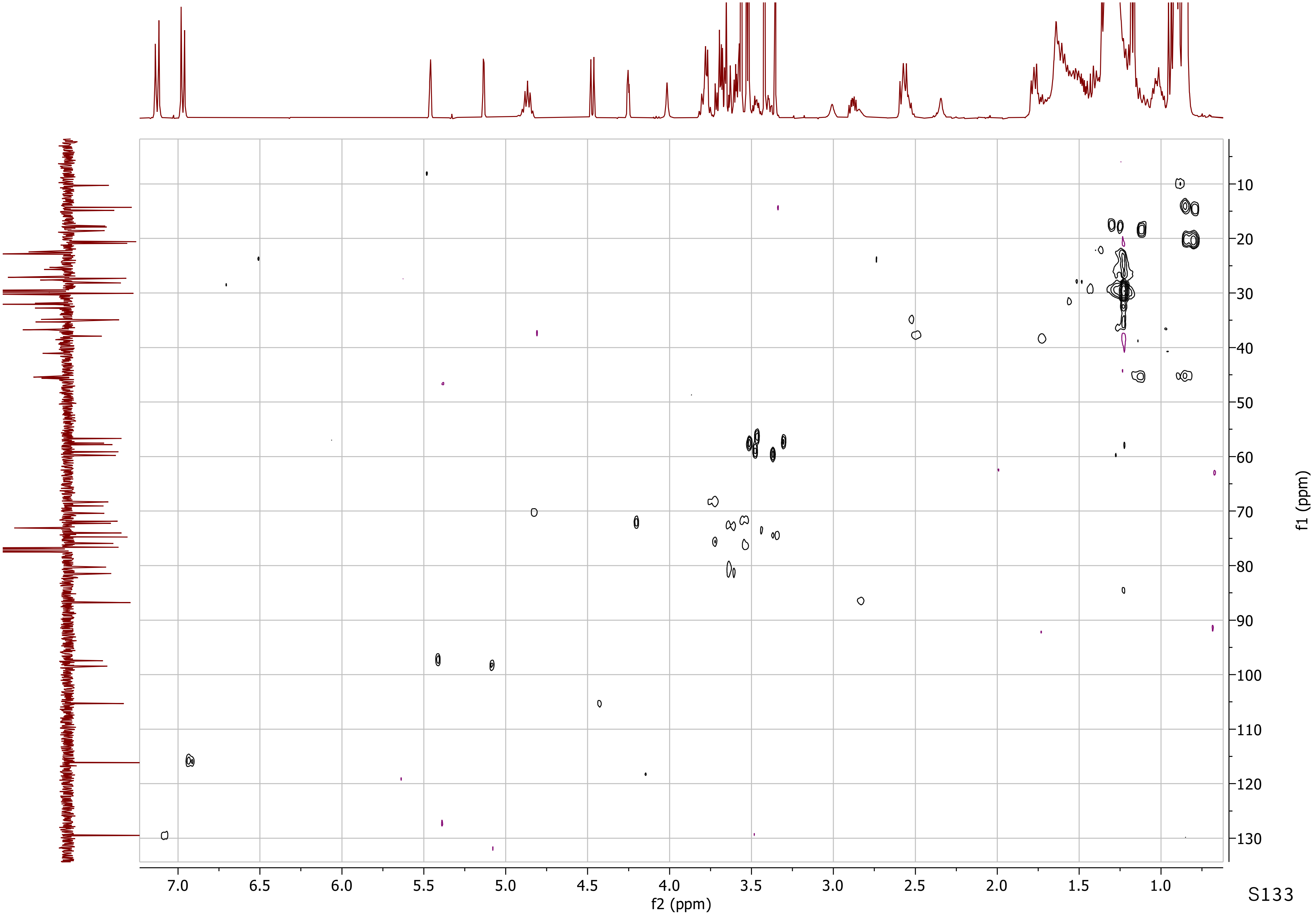

f2 (ppm)
f1 (ppm)

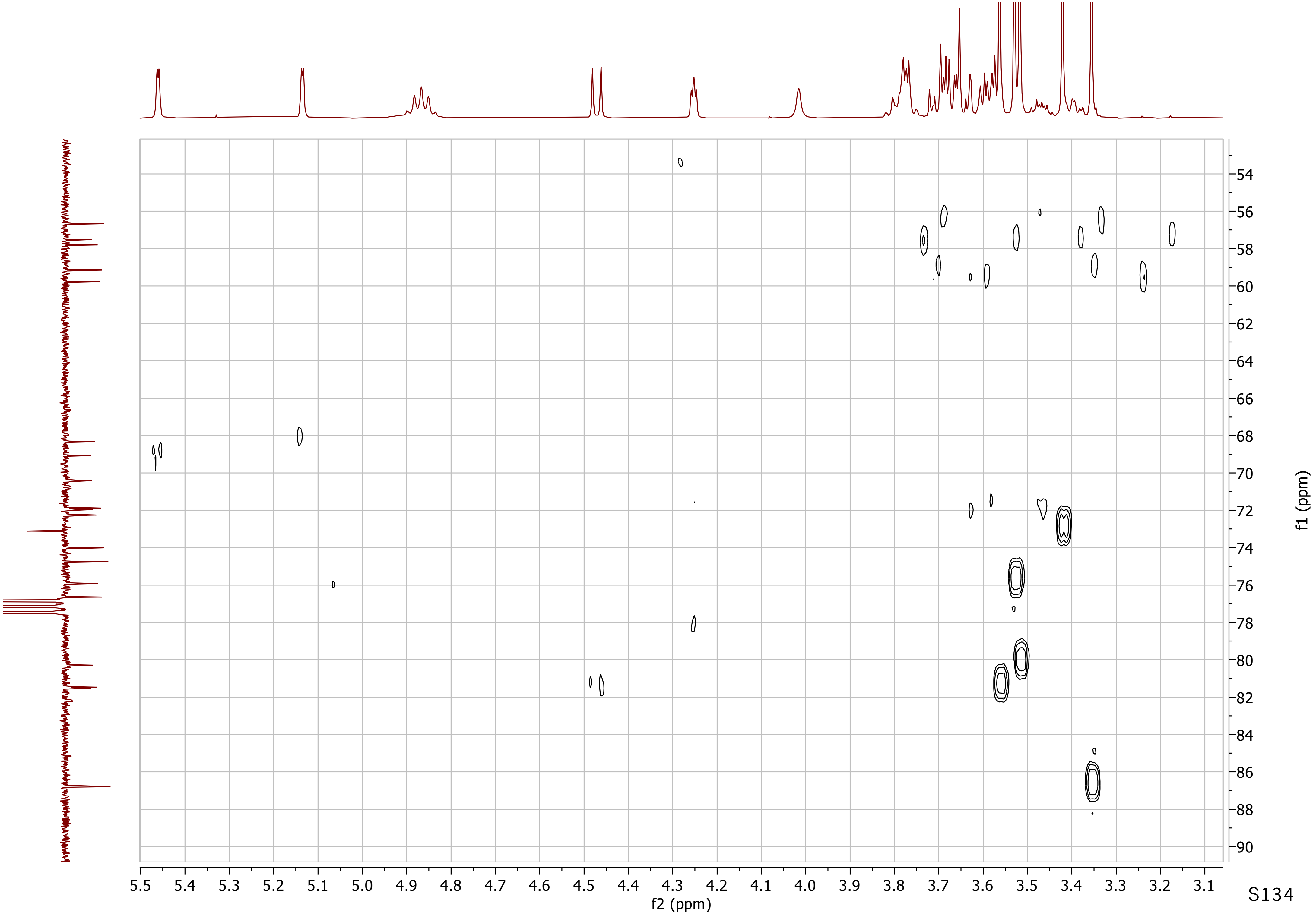
f2 (ppm)
f1 (ppm)

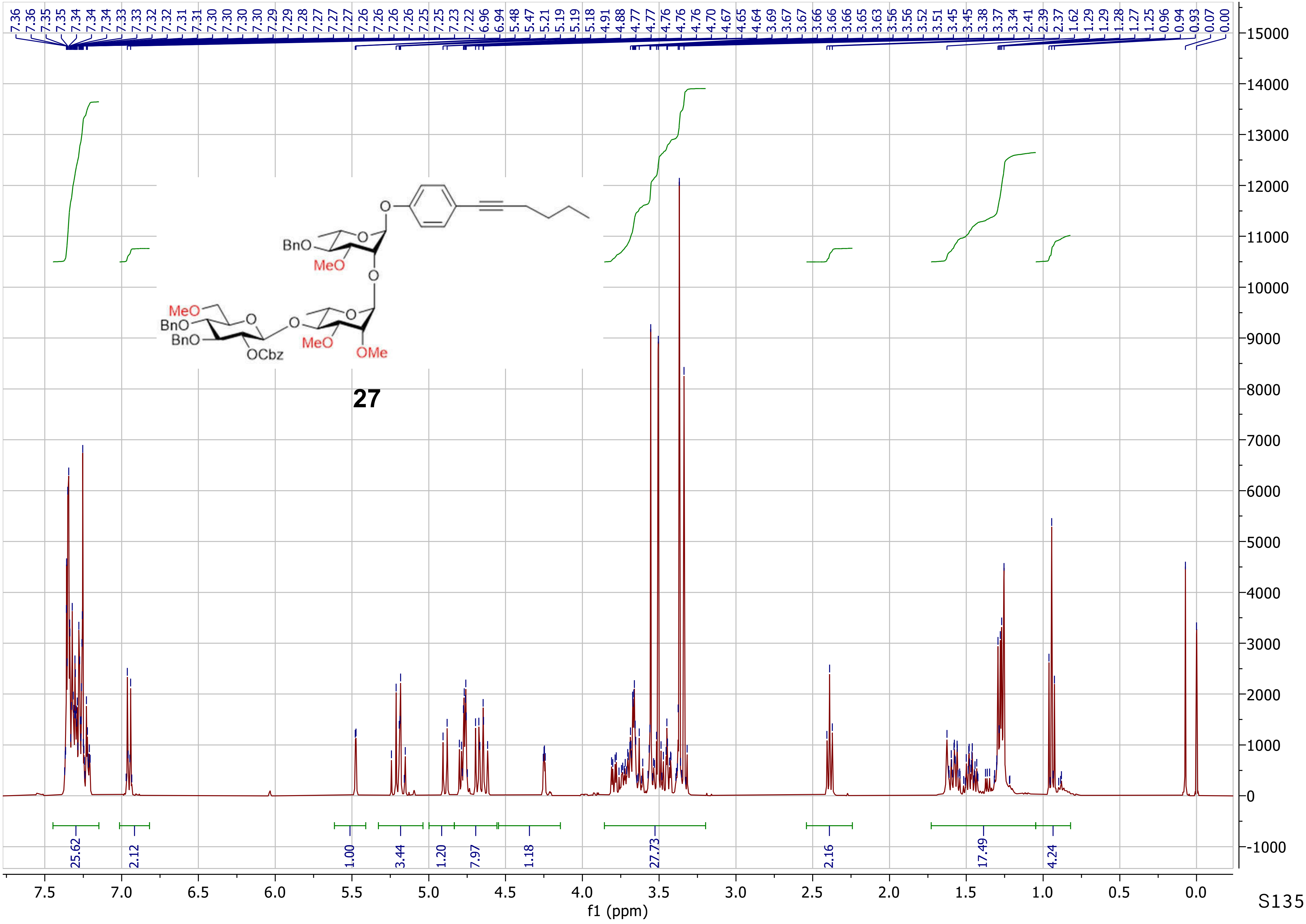

BnO
MeO
MeO
BnO
BnO
OCbz
MeO
OMe
27
25.62
2.12
1.00
3.44
1.20
7.97
1.18
27.73
2.16
17.49
4.24
f1 (ppm)

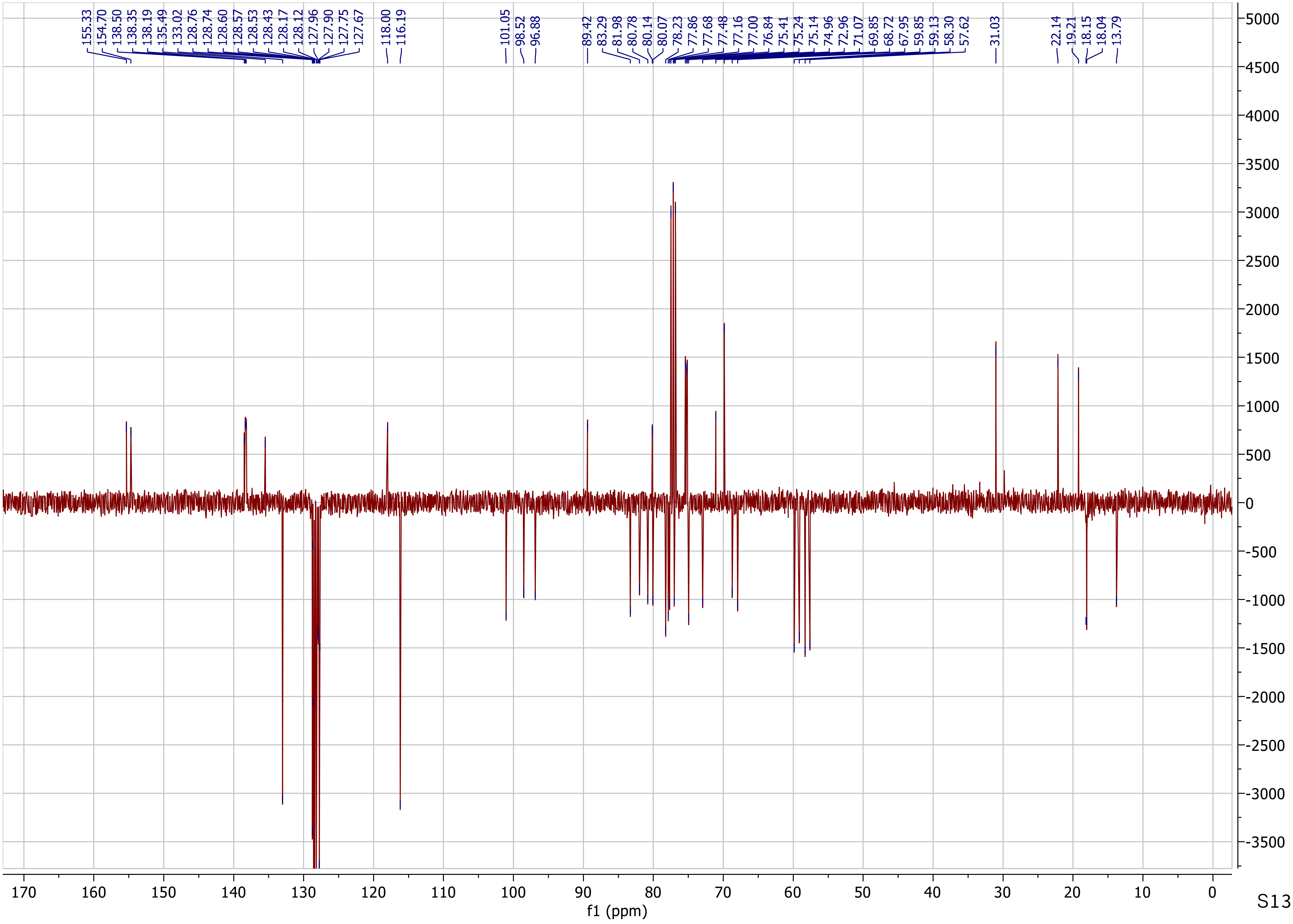
155.33
154.70
138.50
138.35
138.19
135.49
133.02
128.76
128.74
128.60
128.57
128.53
128.43
128.17
128.12
127.96
127.90
127.75
127.67
118.00
116.19
101.05
98.52
96.88
89.42
83.29
81.98
80.78
80.14
80.07
78.23
77.86
77.68
77.48
77.16
77.00
76.84
75.41
75.24
75.14
74.96
72.96
71.07
69.85
68.72
67.95
59.85
59.13
58.30
57.62
31.03
22.14
19.21
18.15
18.04
13.79
f1 (ppm)

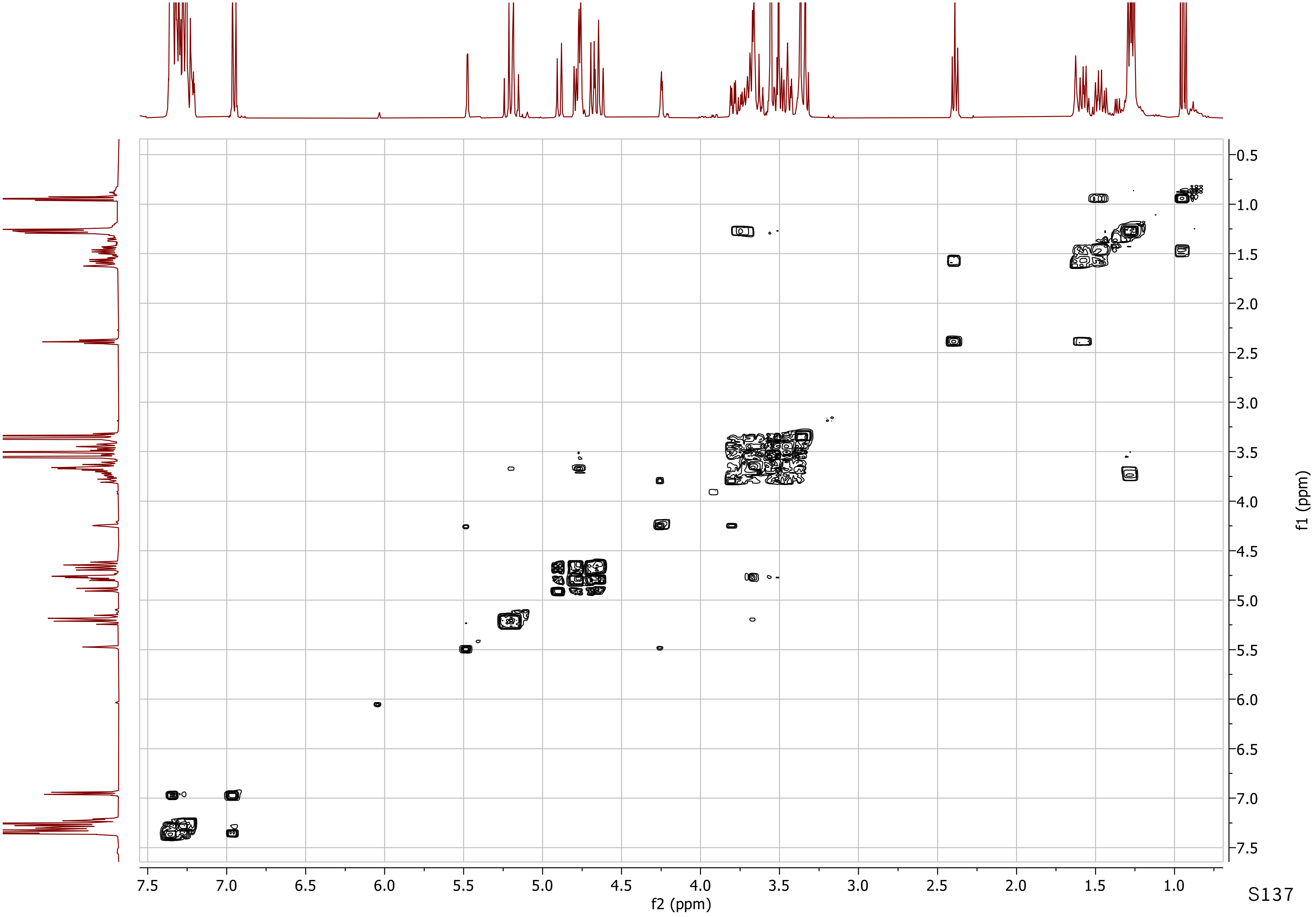

f2 (ppm)
f1 (ppm)

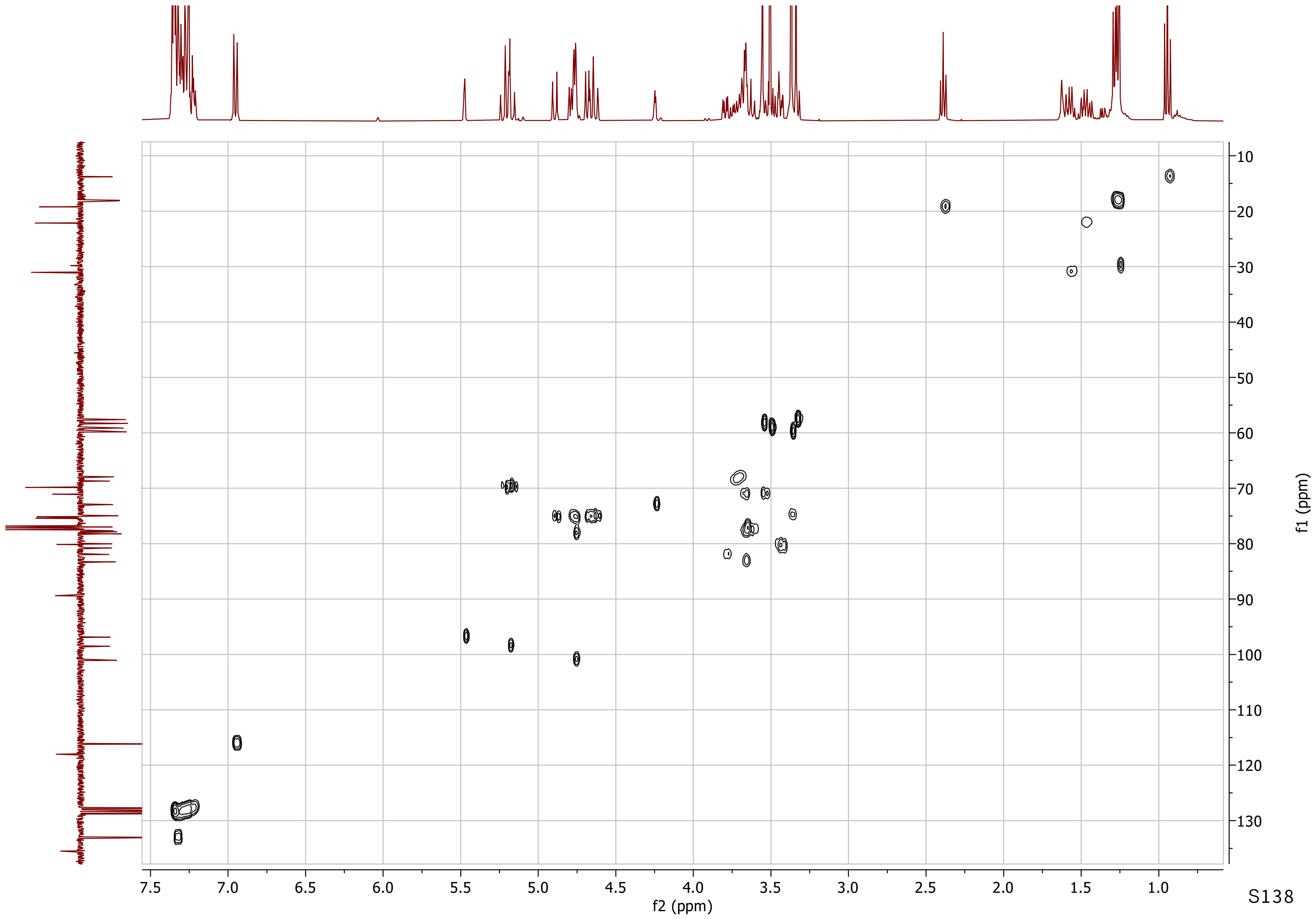
f2 (ppm)
f1 (ppm)

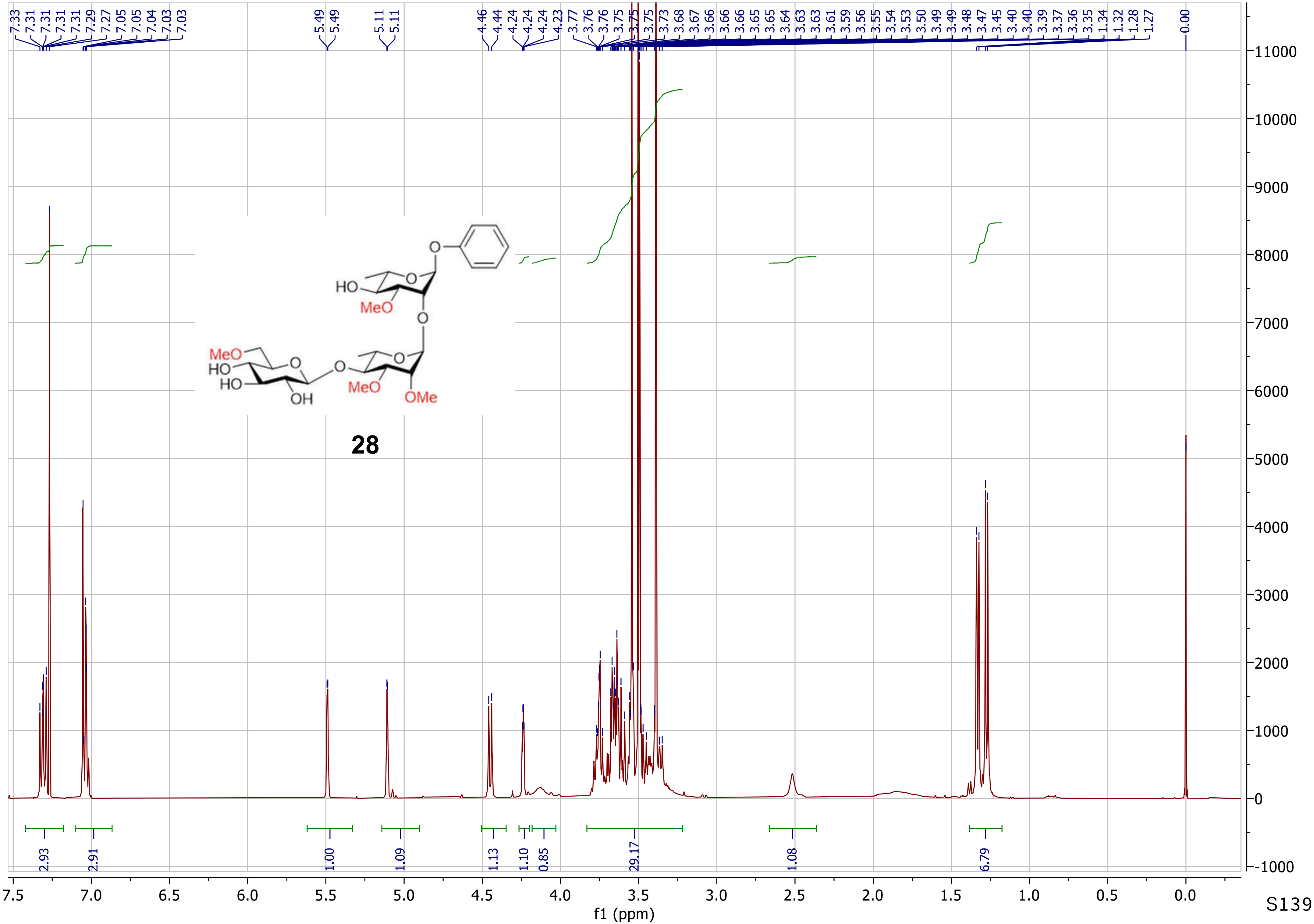
7.33 7.31 7.31 7.31 7.31 7.29 7.27 7.05 7.05 7.04 7.03 7.03
5.49 5.49
5.11 5.11
4.46 4.44 4.24 4.24 4.24 4.23
3.77 3.76 3.76 3.75 3.75 3.75 3.73 3.68 3.67 3.66 3.66 3.65 3.65 3.64 3.63 3.63 3.61 3.59 3.56 3.55 3.54 3.53 3.50 3.49 3.49 3.48 3.47 3.45 3.40 3.40 3.39 3.37 3.36 3.35 1.34 1.32 1.28 1.27
0.00
2.93 2.91 1.00 1.09 1.13 1.10 0.85 29.17 1.08 6.79
HO
MeO
O
MeO
HO
HO
OH
MeO
OMe
28
f1 (ppm)

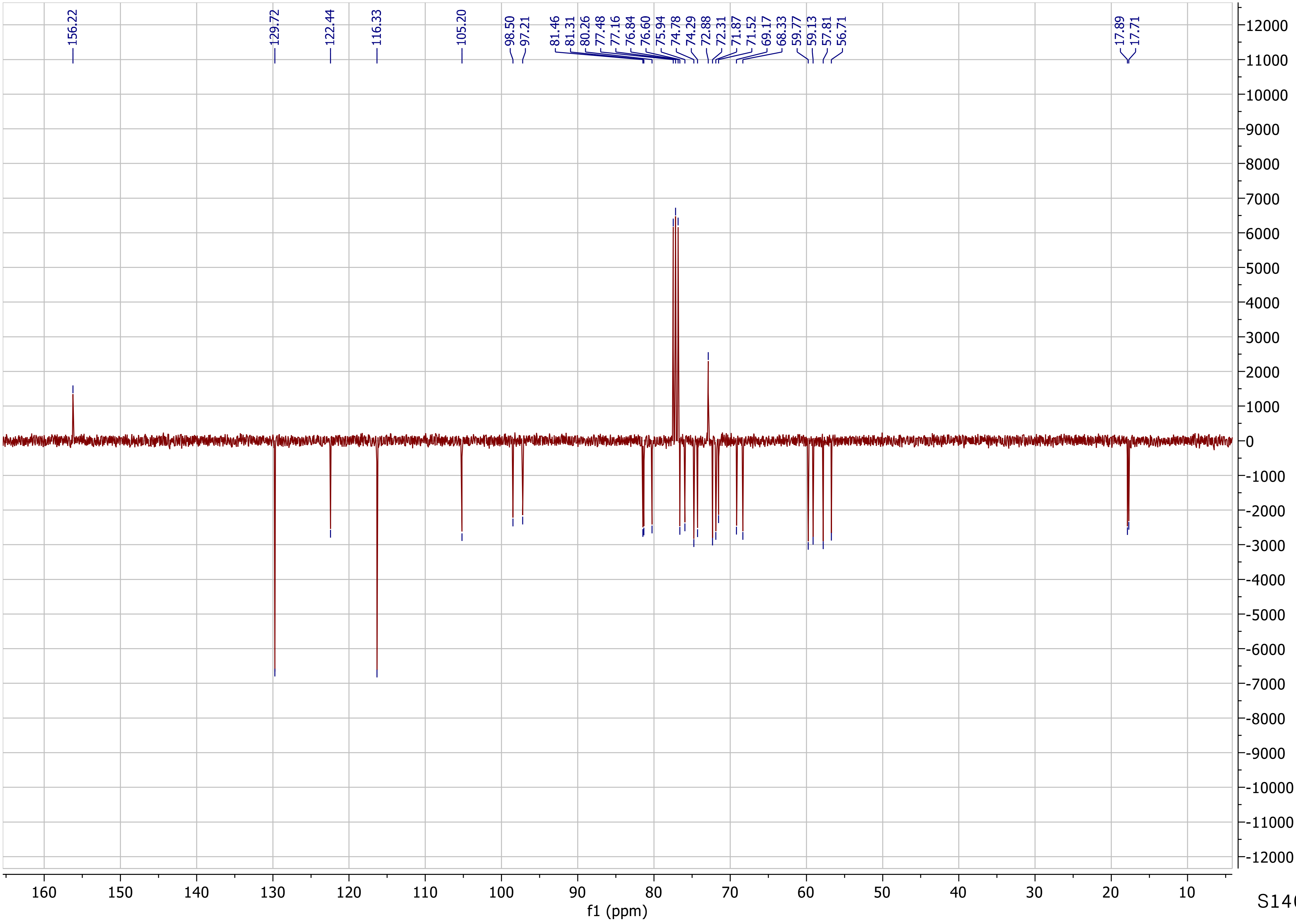
156.22
129.72
122.44
116.33
105.20
98.50
97.21
81.46
81.31
80.26
77.48
77.16
76.84
76.60
75.94
74.78
74.29
72.88
72.31
71.87
71.52
69.17
68.33
59.77
59.13
57.81
56.71
17.89
17.71
f1 (ppm)

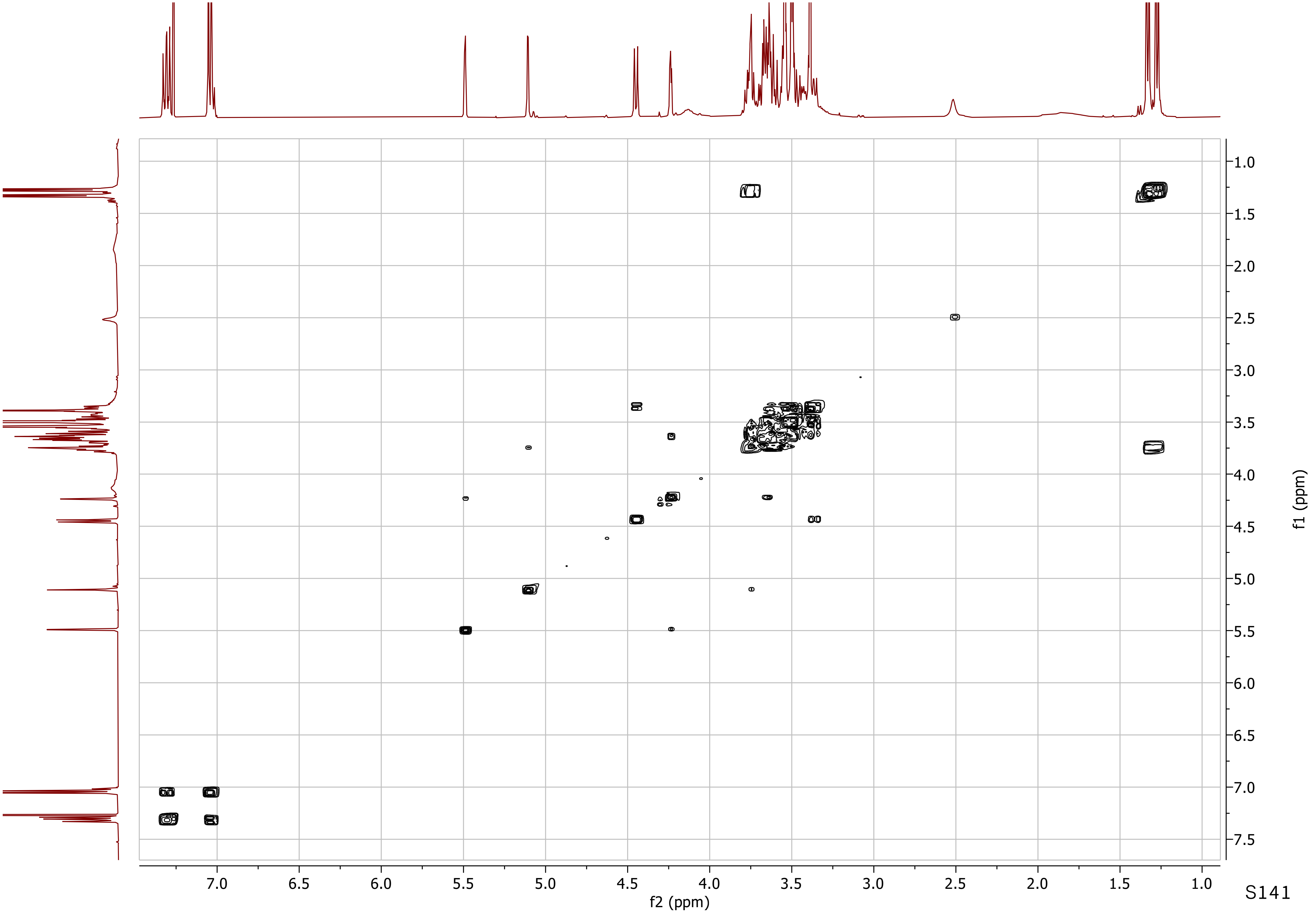

7.0
6.5
6.0
5.5
5.0
4.5
4.0
3.5
3.0
2.5
2.0
1.5
1.0
f2 (ppm)
1.0
1.5
2.0
2.5
3.0
3.5
4.0
4.5
5.0
5.5
6.0
6.5
7.0
7.5
f1 (ppm)

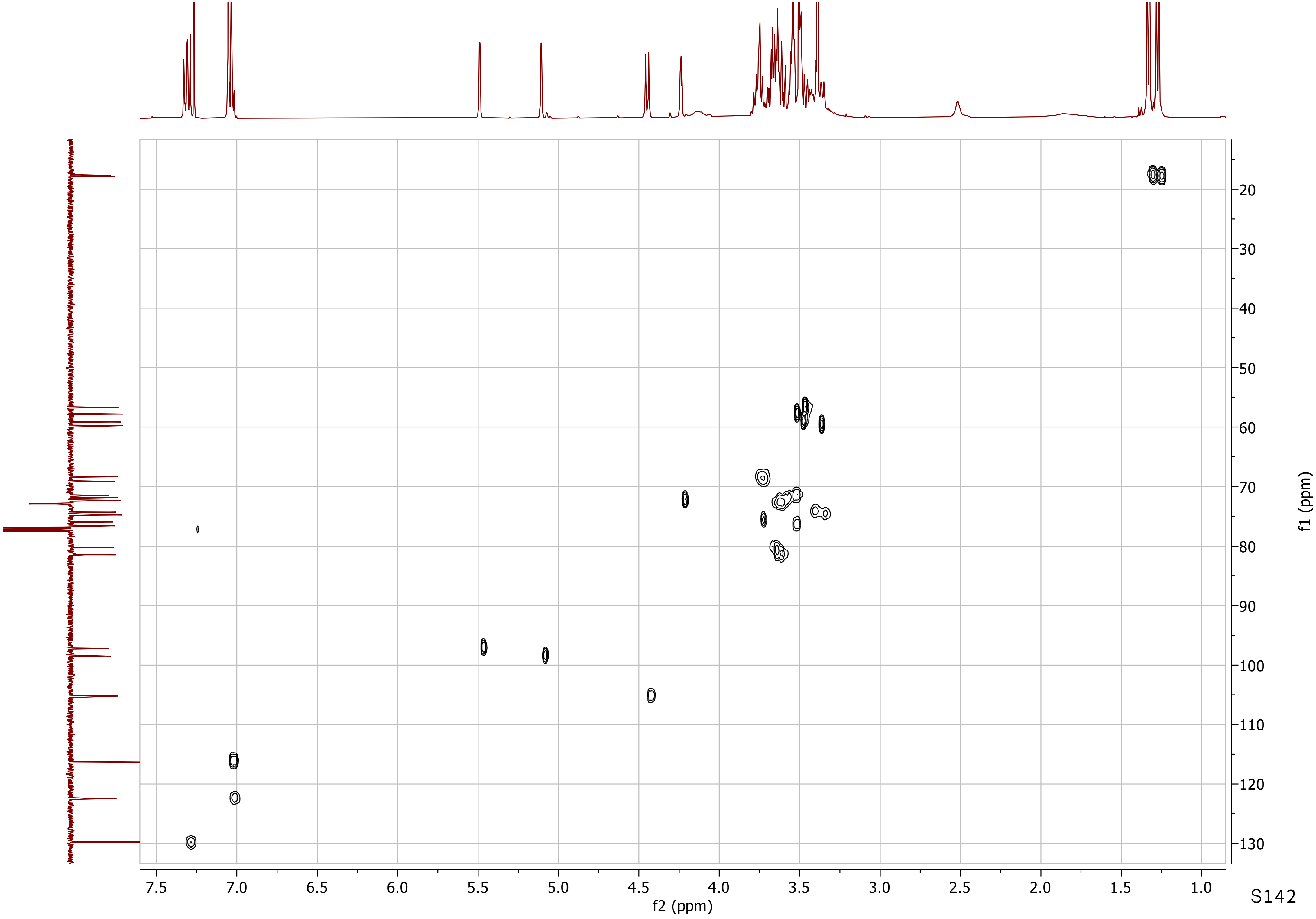

7.5
7.0
6.5
6.0
5.5
5.0
4.5
4.0
3.5
3.0
2.5
2.0
1.5
1.0
f2 (ppm)
20
30
40
50
60
70
80
90
100
110
120
130
f1 (ppm)

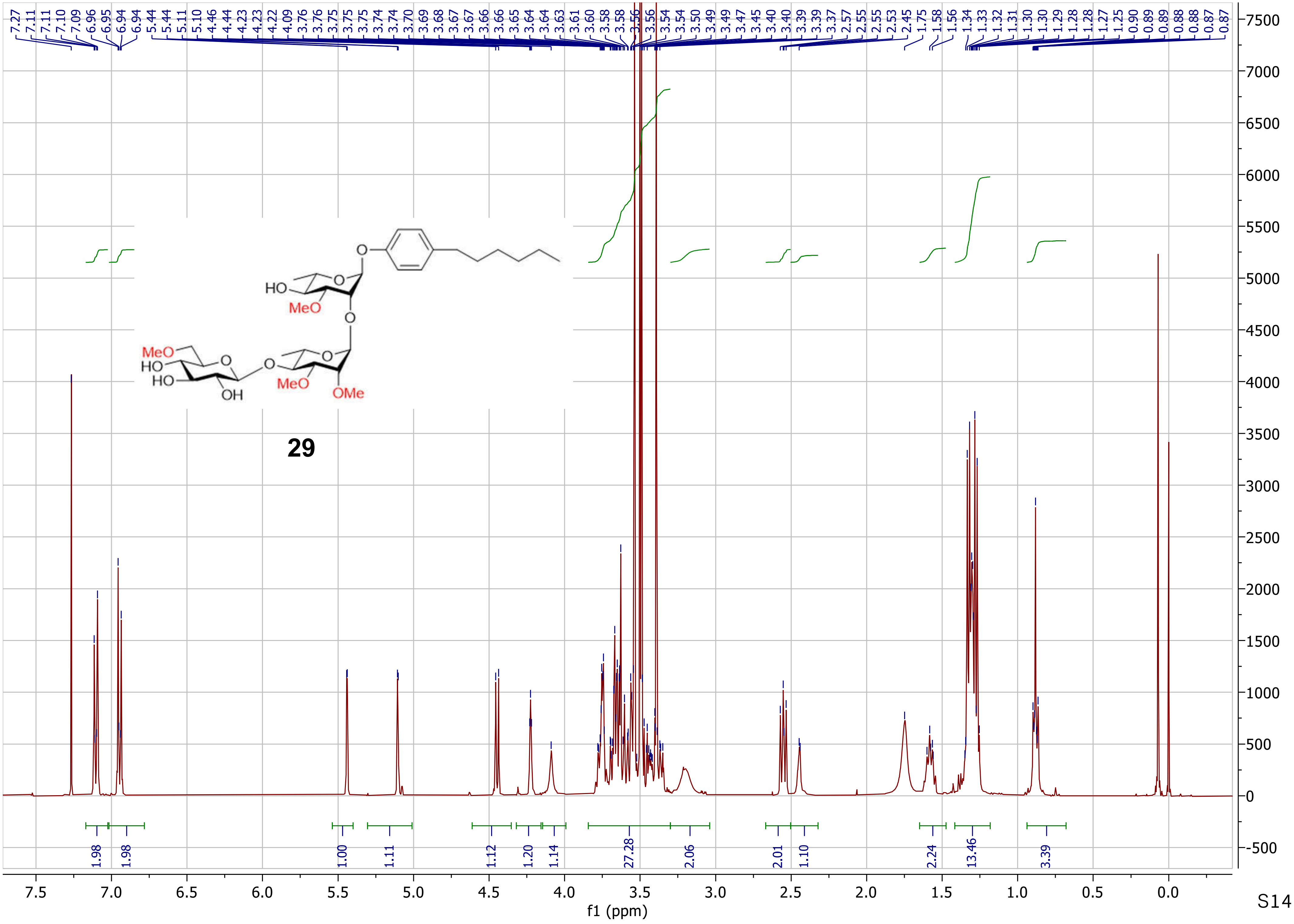
7.27 7.11 7.11 7.10 7.09 6.96 6.95 6.94 6.94 5.44 5.44 5.11 5.10 4.46 4.44 4.23 4.23 4.22 4.09 3.76 3.76 3.75 3.75 3.75 3.74 3.74 3.70 3.69 3.68 3.67 3.67 3.66 3.66 3.65 3.64 3.64 3.63 3.61 3.60 3.58 3.58 3.56 3.56 3.54 3.54 3.50 3.49 3.49 3.47 3.45 3.40 3.40 3.39 3.39 3.37 2.57 2.55 2.55 2.53 2.45 1.75 1.58 1.56 1.34 1.33 1.32 1.31 1.30 1.30 1.29 1.28 1.28 1.27 1.25 0.90 0.89 0.89 0.88 0.88 0.87 0.87
HO
MeO
O
MeO
HO
HO
OH
MeO
OMe
29
1.98 1.98 1.00 1.11 1.12 1.20 1.14 27.28 2.06 2.01 1.10 2.24 13.46 3.39
f1 (ppm)

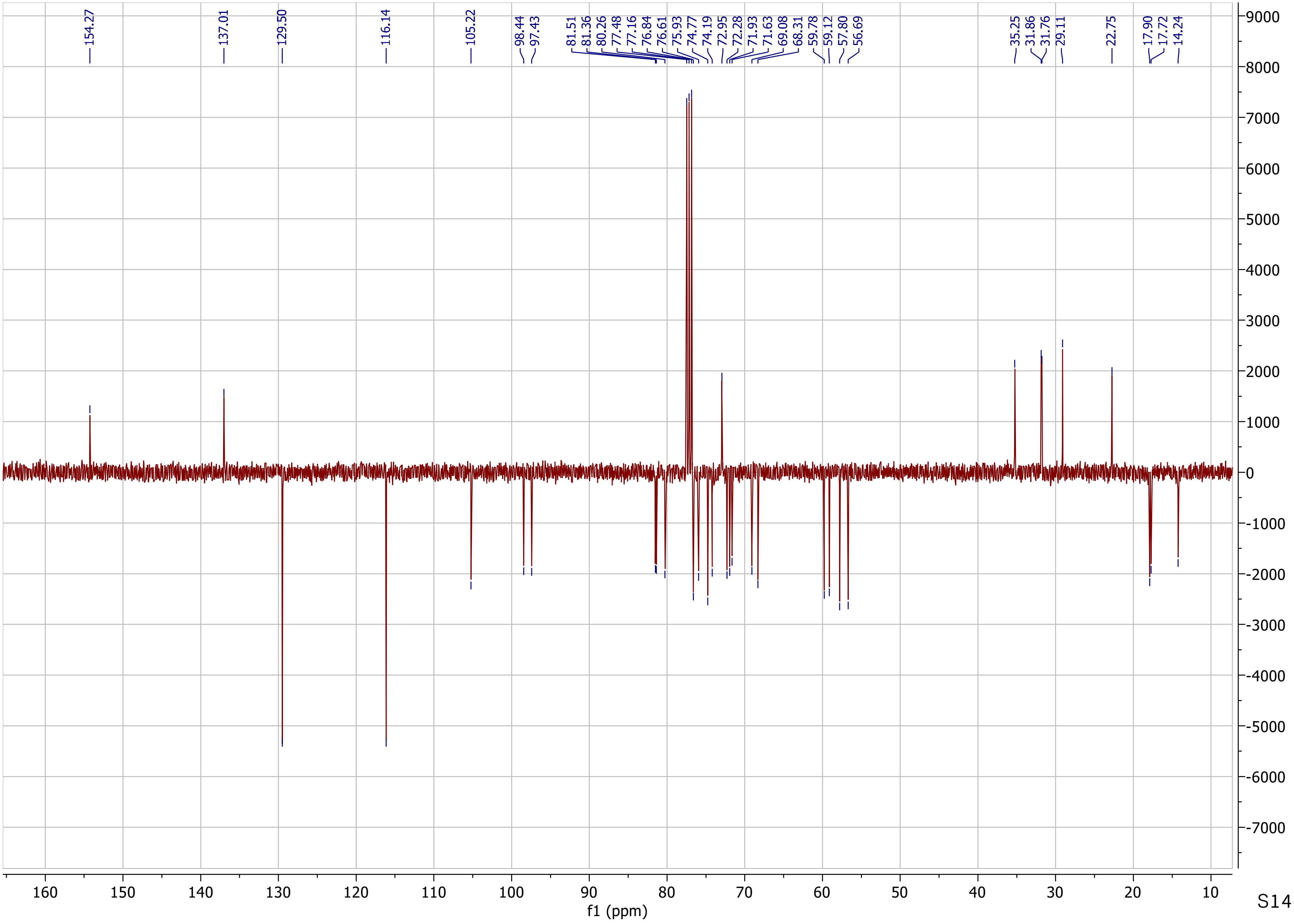
154.27
137.01
129.50
116.14
105.22
98.44
97.43
81.51
81.36
80.26
77.48
77.16
76.84
76.61
75.93
74.77
74.19
72.95
72.28
71.93
71.63
69.08
68.31
59.78
59.12
57.80
56.69
35.25
31.86
31.76
29.11
22.75
17.90
17.72
14.24
f1 (ppm)

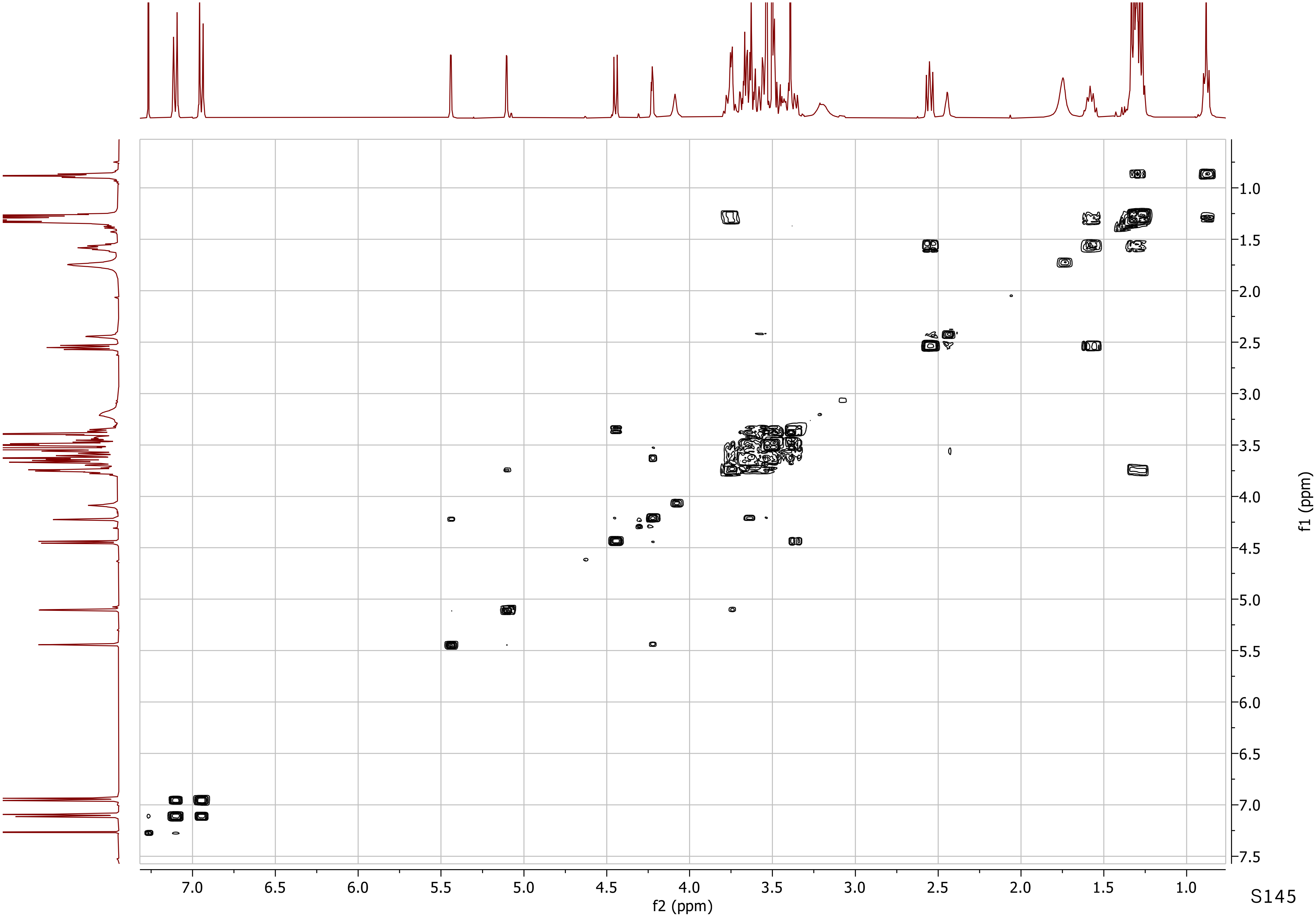

f2 (ppm)
f1 (ppm)
7.0
6.5
6.0
5.5
5.0
4.5
4.0
3.5
3.0
2.5
2.0
1.5
1.0

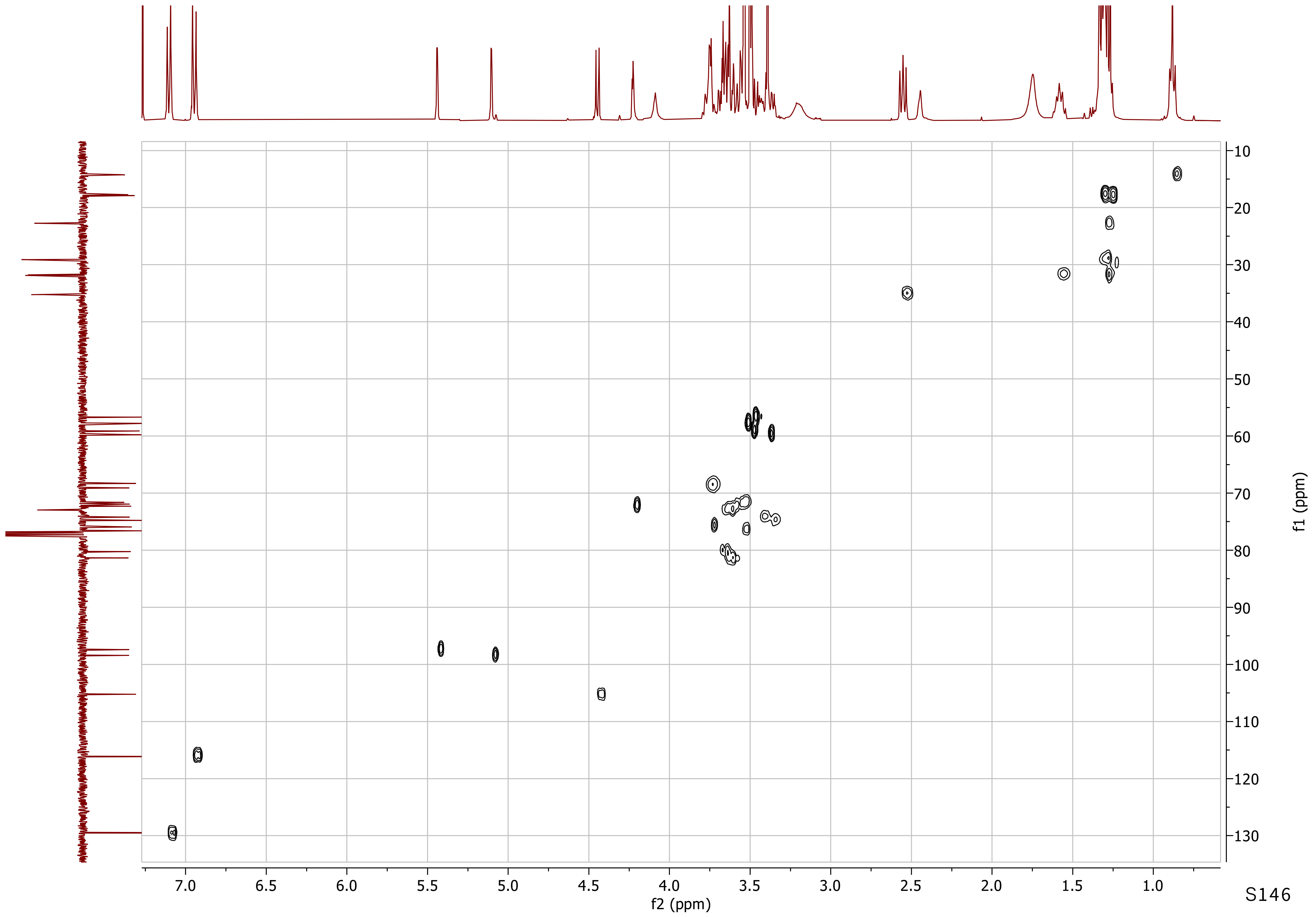

f2 (ppm)
f1 (ppm)

Spot C
HH-COSY

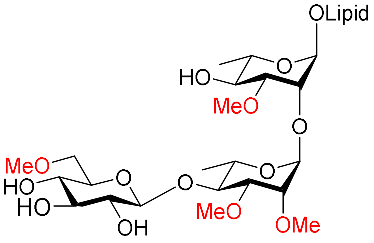

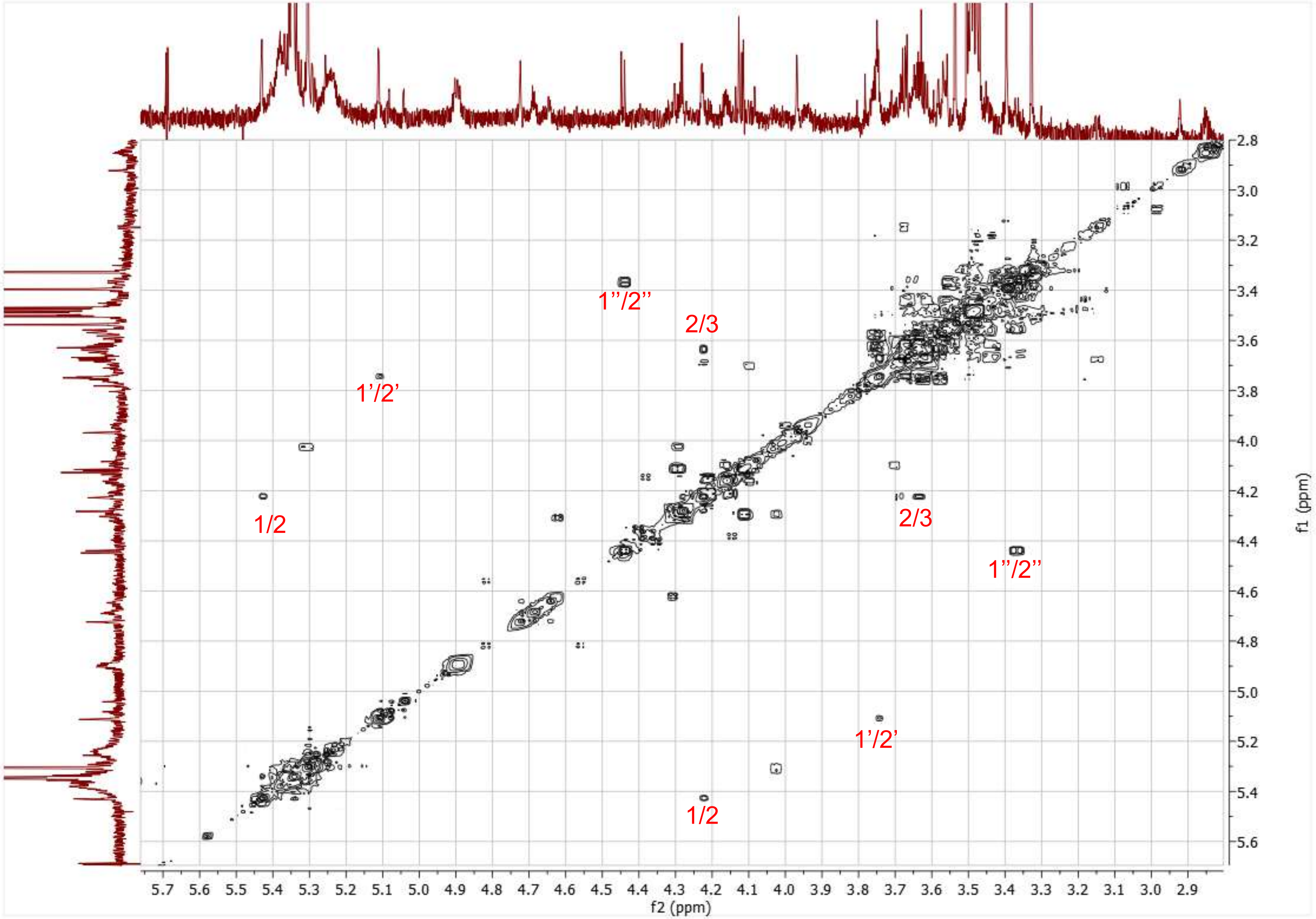

Spot C
HH-COSY

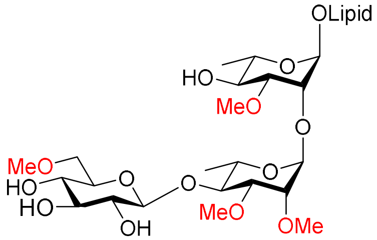

Synthetic PGL-III
HH-COSY

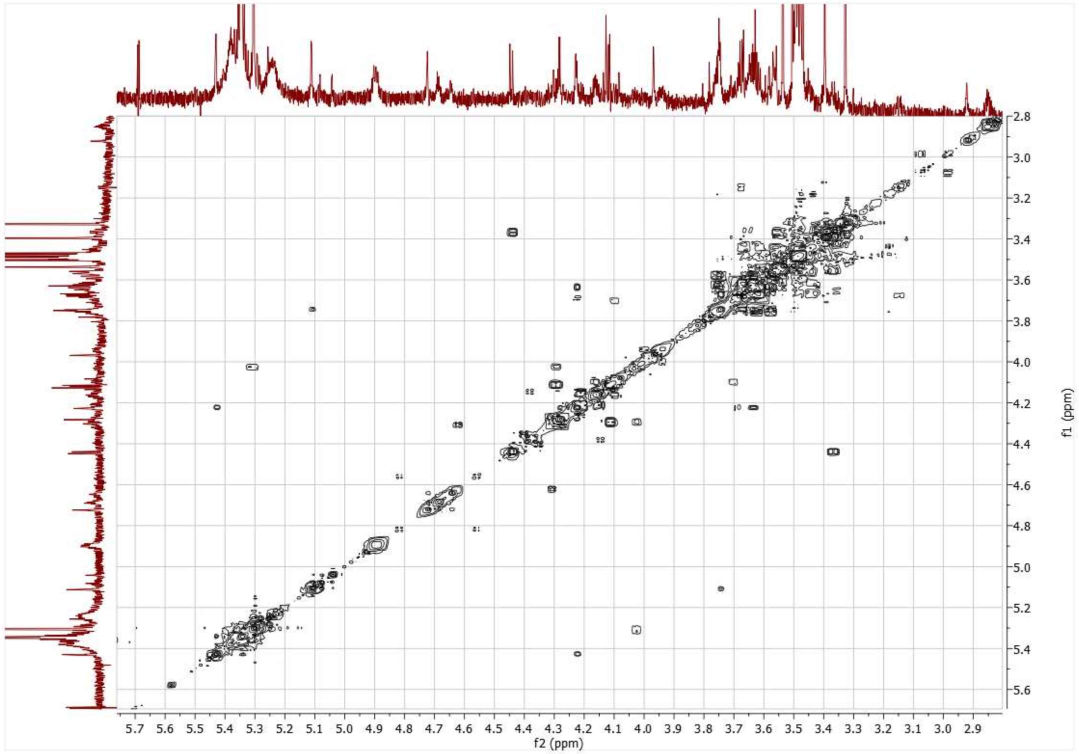

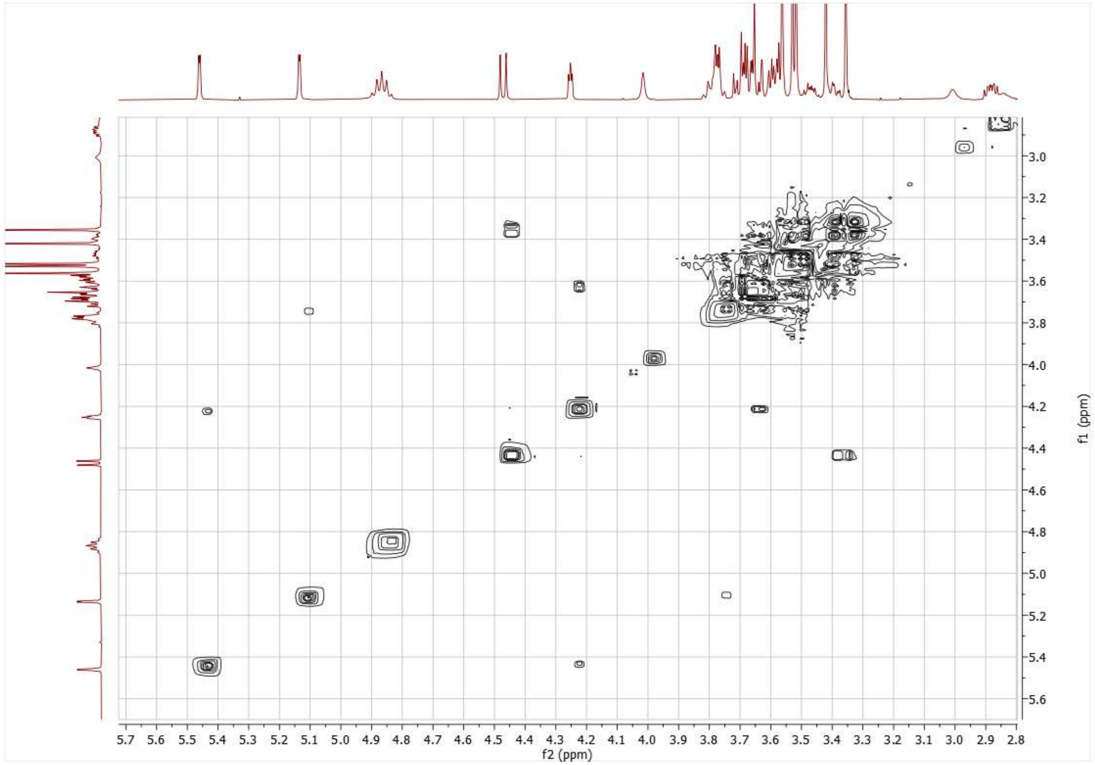

Spot C
HSQC

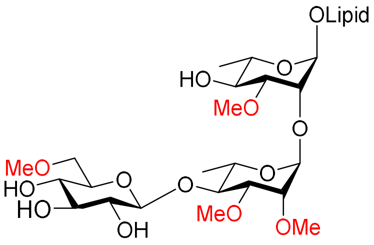

OLipid
HO
MeO
O
MeO
HO
HO
OH
MeO
OMe

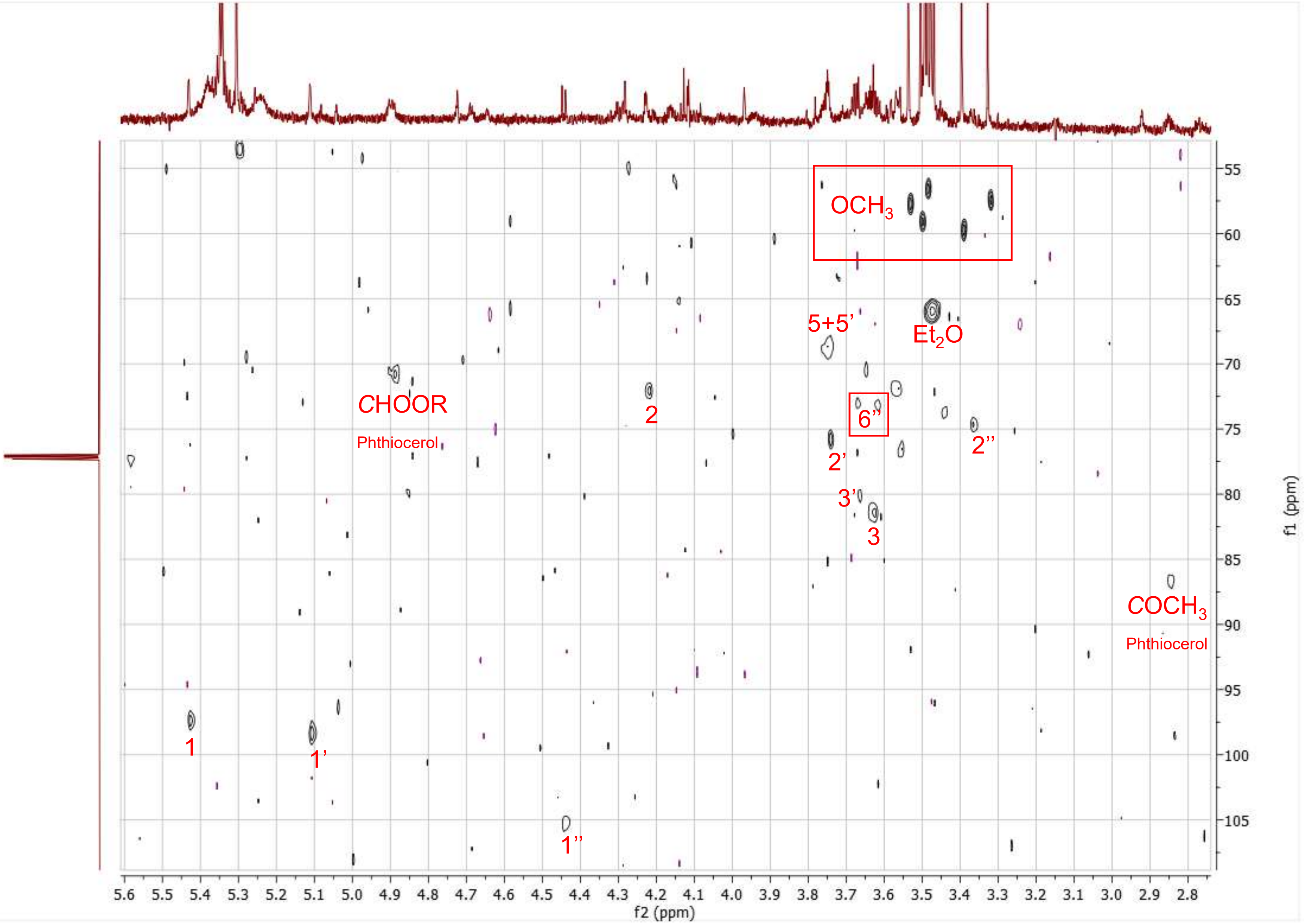

OCH3
5+5'
Et2O
CHOOR
Phthiocerol
2
6''
2'
2''
3'
3
COCH3
Phthiocerol
1
1'
1''
f2 (ppm)
f1 (ppm)

Spot C
HSQC

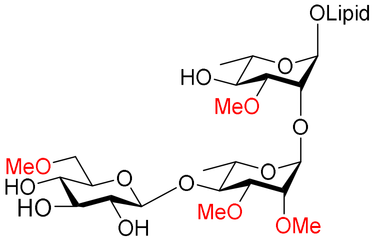

Synthetic PGL-III
HSQC

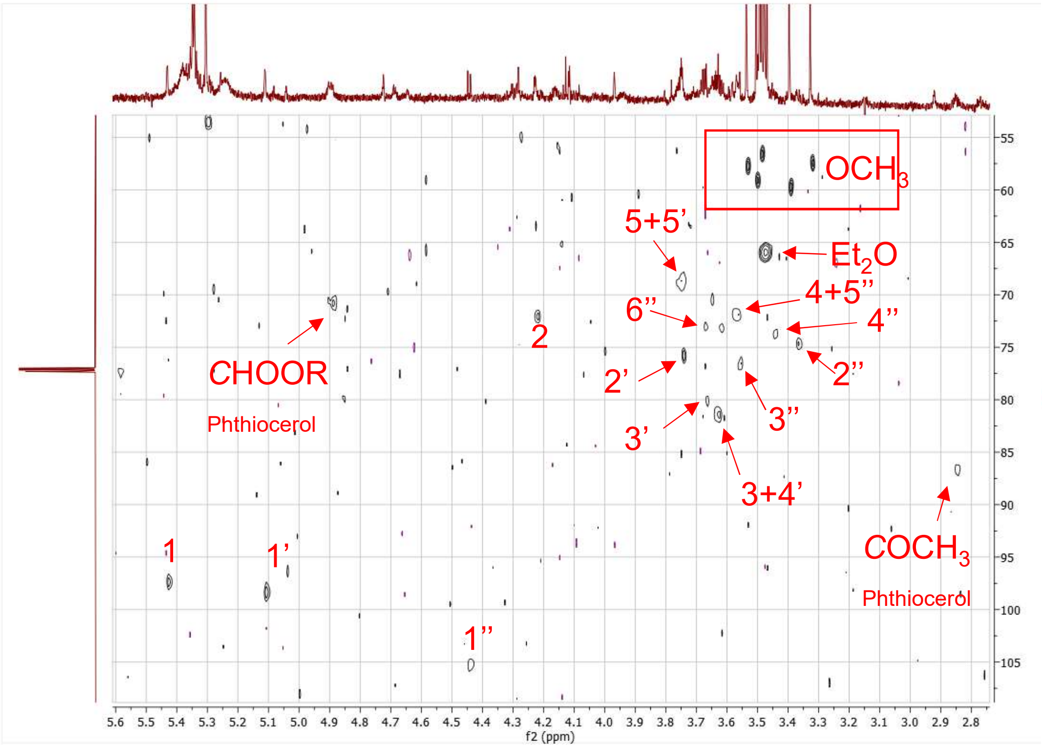

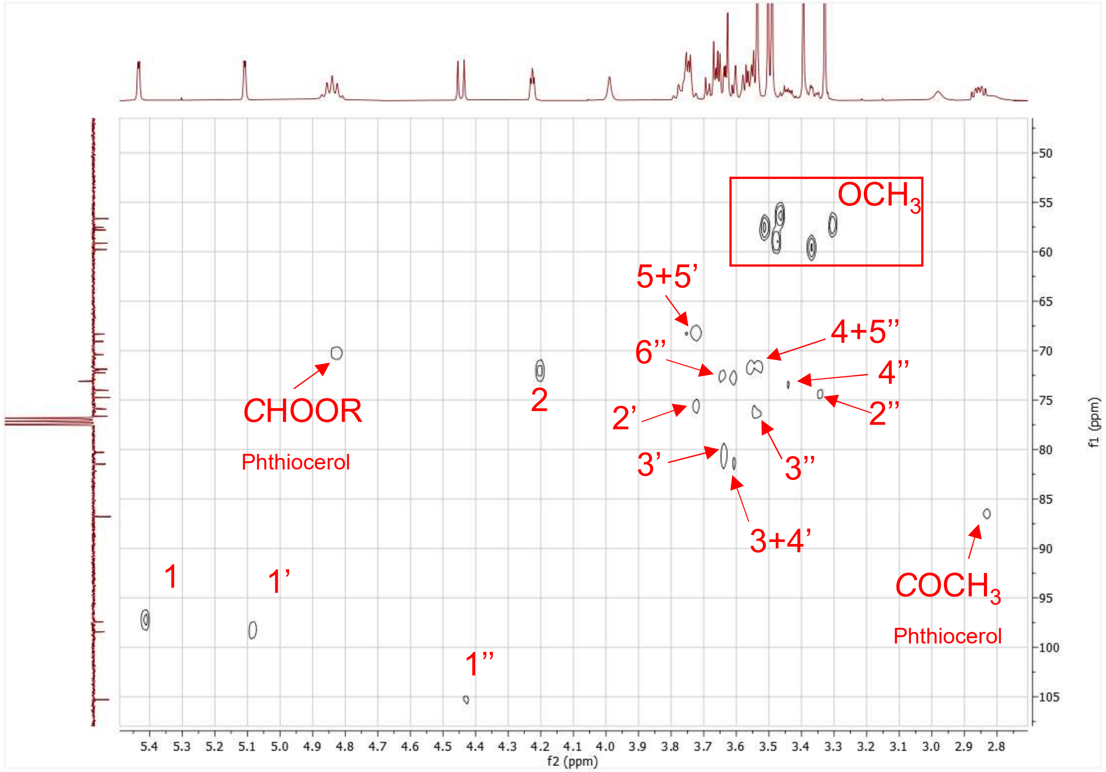

Spot C
HMBC

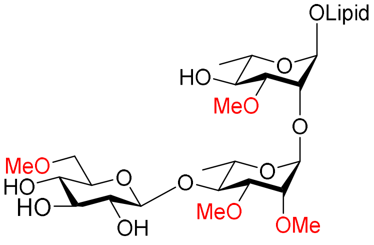
OLipid
HO
MeO
O
MeO
HO
HO
OH
MeO
OMe

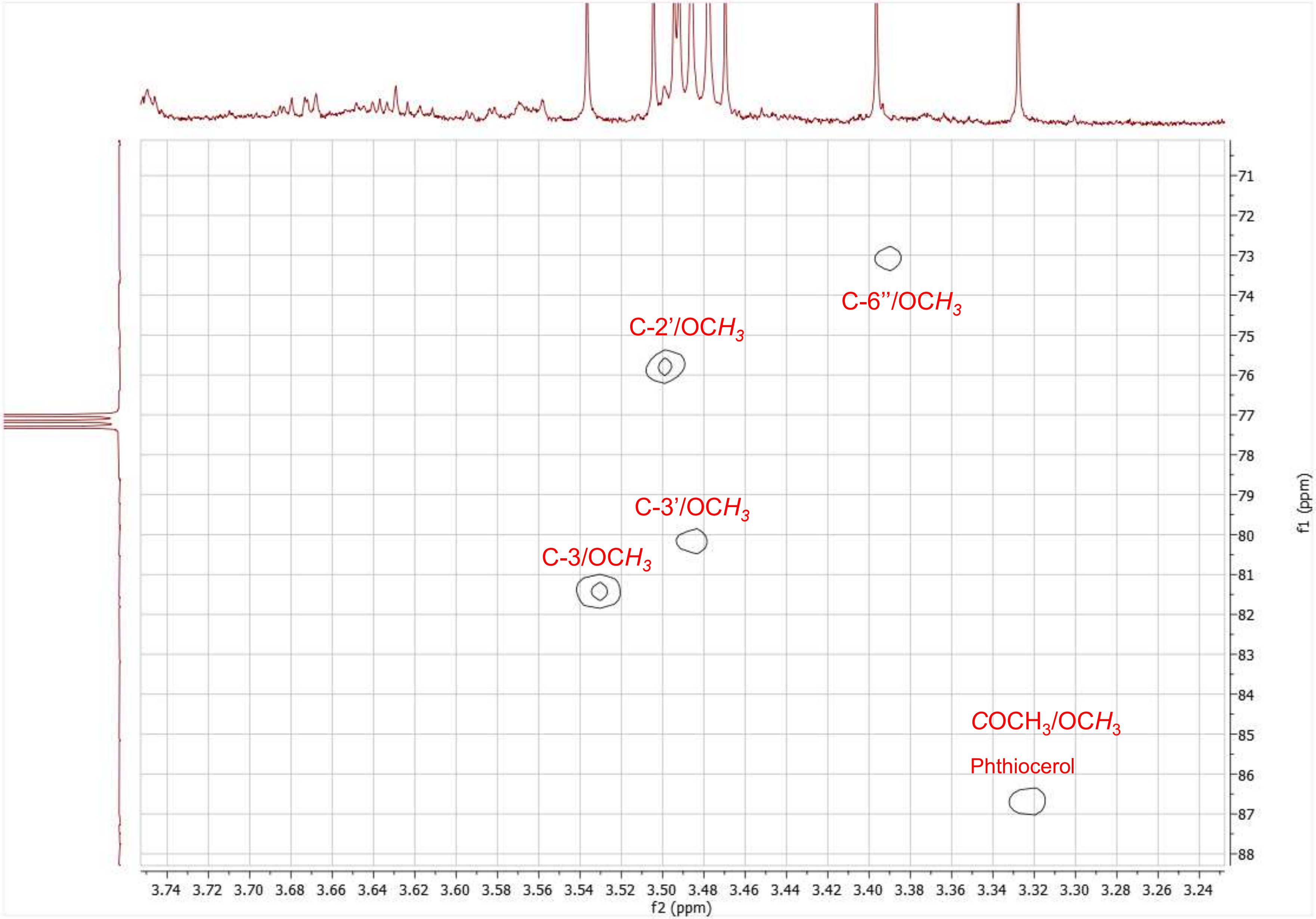
C-6''/OCH3
C-2'/OCH3
C-3'/OCH3
C-3/OCH3
COCH3/OCH3
Phthiocerol
f2 (ppm)
f1 (ppm)

Spot C
HMBC

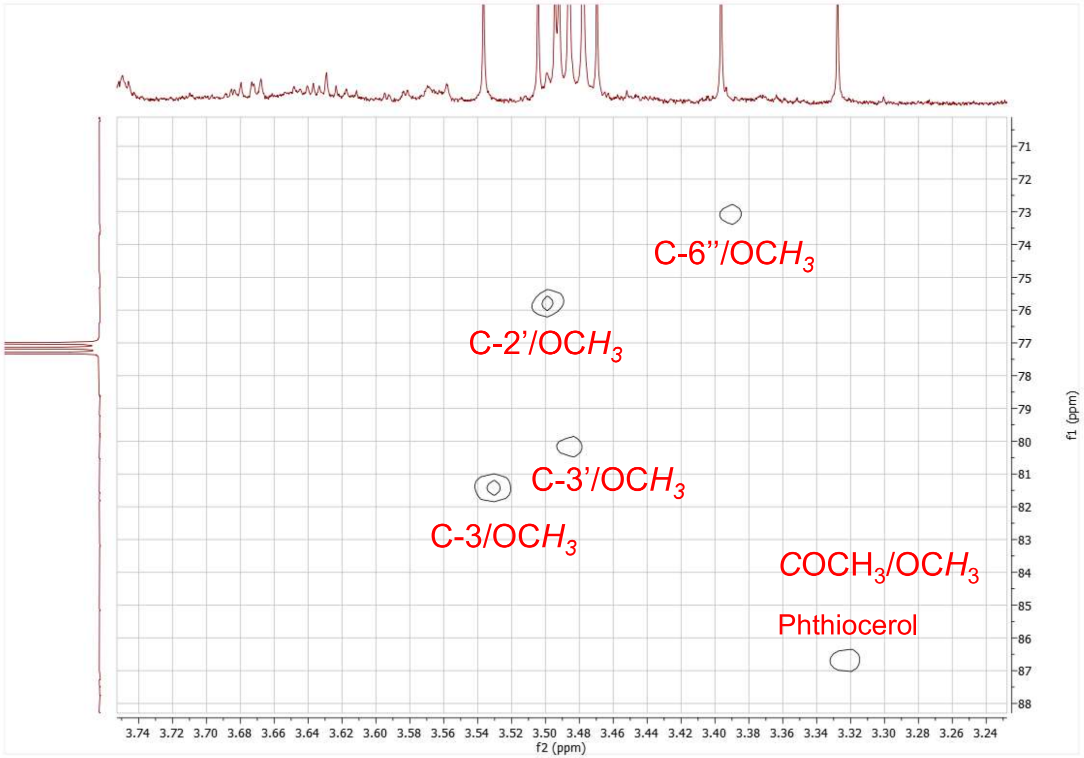

Synthetic PGL-III
HMBC

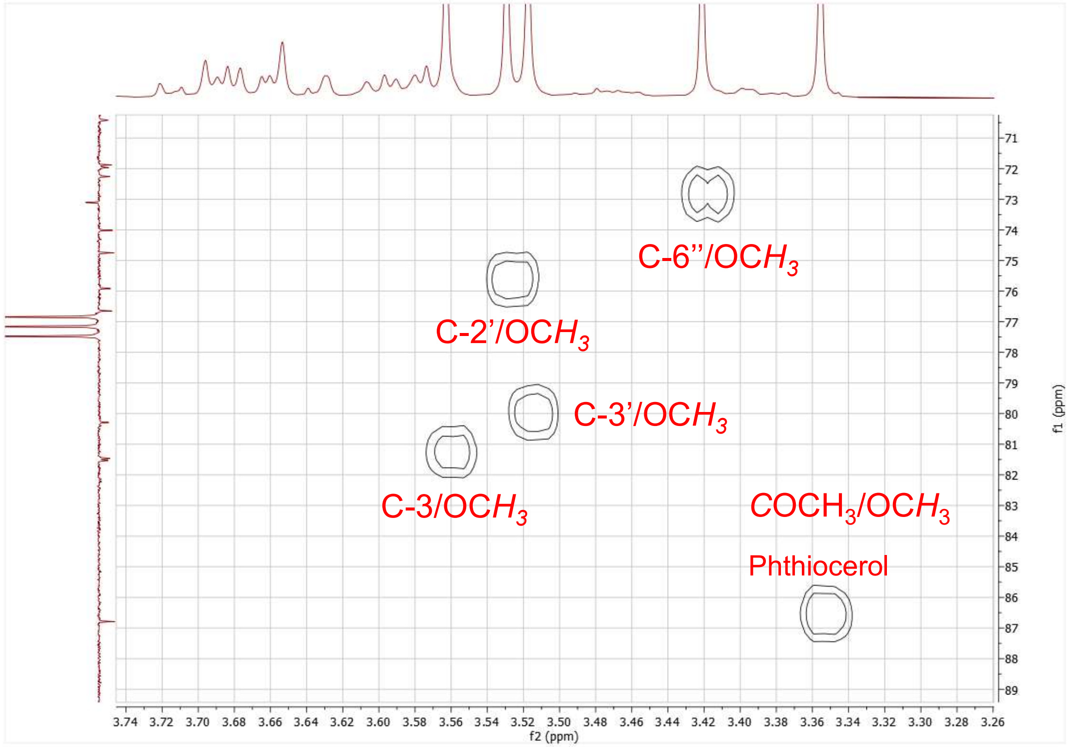

Supplement: Supplementary file 1 — oc3c00040_si_001.pdf [file oc3c00040_si_001.pdf]
